# Association of Inherited Genetic Factors With Drug-Induced Hepatic Damage Among Children With Acute Lymphoblastic Leukemia

**DOI:** 10.1001/jamanetworkopen.2022.48803

**Published:** 2022-12-29

**Authors:** Wenjian Yang, Seth E. Karol, Keito Hoshitsuki, Shawn Lee, Eric C. Larsen, Naomi Winick, William L. Carroll, Mignon L. Loh, Elizabeth A. Raetz, Stephen P. Hunger, Stuart S. Winter, Kimberly P. Dunsmore, Meenakshi Devidas, Mary V. Relling, Jun J. Yang

**Affiliations:** 1 Department of Pharmacy and Pharmaceutical Sciences, St Jude Children’s Research Hospital, Memphis, Tennessee; 2 Department of Oncology, St Jude Children’s Research Hospital, Memphis, Tennessee; 3 Maine Children’s Cancer Program, Scarborough; 4 Department of Pediatrics, The University of Texas Southwestern Medical Center, Dallas; 5 Department of Pediatrics, New York University Grossman School of Medicine, New York; 6 Seattle Children’s Hospital, the Ben Town Center for Childhood Cancer Research, University of Washington, Seattle; 7 Department of Pediatrics, Children’s Hospital of Philadelphia, Perelman School of Medicine, University of Pennsylvania, Philadelphia; 8 Cancer and Blood Disorders Program, Children’s Minnesota, Minneapolis; 9 Department of Pediatrics, University of Virginia, Charlottesville; 10 Department of Global Pediatric Medicine, St Jude Children’s Research Hospital, Memphis, Tennessee

## Abstract

**Importance:**

Acute lymphoblastic leukemia (ALL) is the most common childhood cancer. Hepatotoxic effects, including hyperbilirubinemia and elevated alanine aminotransferase (ALT) and aspartate aminotransferase (AST) levels, are common during all phases of therapy and are linked to several chemotherapeutic agents, including asparaginase, mercaptopurine, and methotrexate.

**Objective:**

To determine which genetic variants were associated with hyperbilirubinemia and elevated ALT and AST levels in children, adolescents, and young adults treated for ALL.

**Design, Setting, and Participants:**

This retrospective analysis of a multiethnic genome-wide association study was conducted between January 1, 2019, and April 15, 2022, including patients treated as part of Children’s Oncology Group (COG) trials with centers in the United States, Canada, and Australia, which accrued data from December 29, 2003, to January 21, 2011 (AALL0232), and from January 22, 2007, to July 24, 2014 (AALL0434). Germline genotypes were interrogated using genome-wide arrays and imputed using a National Institutes of Health TOPMed Imputation server. Mixed-effects logistic regressions were used to account for multiple episodes for an individual patient. Genotype × treatment phase interaction was tested to uncover phase-specific genetic risk factors.

**Exposures:**

Total duration of multiagent protocol chemotherapy ranging from 2.5 to 3.5 years.

**Main Outcomes and Measures:**

The primary outcomes were National Cancer Institute Common Terminology Criteria for Adverse Events (version 4) hyperbilirubinemia of grade 3 or higher and elevated liver ALT and AST levels.

**Results:**

A total of 3557 participants were included in the analysis (2179 [61.3%] male; median age, 11.1 [range, 1-30] years). Among 576 known variants associated with these liver function test results in the general population, *UGT1A1* variant rs887829 and *PNPLA3* variant rs738409 were associated with increased risk of hyperbilirubinemia (odds ratio [OR], 2.18 [95% CI, 1.89-2.53]; *P* = 6.7 × 10^−27^) and ALT and AST levels (OR, 1.27 [95% CI, 1.15-1.40]; *P* = 3.7 × 10^−7^), respectively, during treatment for ALL. Corresponding polygenic risk scores were associated with hepatotoxic effects across all therapy phases and were largely driven by *UGT1A1* and *PNPLA3* variants. Genome-wide association analysis revealed an age-specific variant near the *CPT1A* gene that was only associated with elevated ALT and AST levels among patients younger than 10 years (OR, 1.28 [95% CI, 1.18-1.39]; *P* = 8.7 × 10^−10^).

**Conclusions and Relevance:**

These results suggest a strong genetic basis for interpatient variability in hyperbilirubinemia and aminotransferase level elevations during leukemia chemotherapy.

## Key Points

**Question:**

What are the genetic drivers of interpatient variability in the occurrence of hepatotoxic effects during treatment for acute lymphoblastic leukemia (ALL)?

**Findings:**

In this genetic association study of 3557 children, adolescents, and young adults receiving ALL therapy, variants in *UGT1A1* and *PNPLA3* were associated with hyperbilirubinemia and elevated alanine aminotransferase and aspartate aminotransferase levels, respectively. A polygenic risk score–based analysis demonstrated that the *UGT1A1* variant was the primary driver of elevated bilirubin levels, while other genetic variants contributed to aminotransferase levels even after adjusting for *PNPLA3*.

**Meaning:**

These findings suggest that there is an association between genetic factors and interpatient variability in the occurrence of hepatotoxic effects during treatment for ALL.

## Teaser

This genetic association study replicated previously identified genetic factors and assessed novel genetic factors associated with hepatic toxic effects during treatment in pediatric patients with acute lymphoblastic leukemia (ALL).

## Introduction

Childhood acute lymphoblastic leukemia (ALL) is the most common pediatric cancer. Cure rates now exceed 90%,^1^ and decreasing treatment-related toxic effects and improving patients’ quality of life have become increasingly important to further improve overall patient outcomes. ALL therapy is prolonged for up to 3 years and is characterized by several phases of intensive chemotherapy.^2^ Patients can experience a plethora of adverse effects, including hepatotoxic effects, as evidenced by hyperbilirubinemia and elevated levels of alanine aminotransferase (ALT) and aspartate aminotransferase (AST). Hepatotoxic effects can be caused by chemotherapies such as methotrexate, asparaginase, and mercaptopurine, but can also be caused by infections, blood transfusions, and drugs used in supportive care. Even though these toxic effects usually resolve after withholding therapy, their long-term implications remain unclear. Uncovering genetic and nongenetic factors associated with elevated bilirubin, ALT, and AST levels can help identify patients who are likely to develop severe hepatotoxic effects and facilitate treatment individualization. Elucidating the underlying biological mechanisms could provide insights into the development of rational interventions.

Previously, using a candidate gene approach, Kishi et al^3^ identified the *UGT1A1* promoter TA repeat variant (rs8175347) as associated with hyperbilirubinemia during all treatment phases in a single-institution study at St Jude Children’s Research Hospital (Total XIIIB protocol). In a 2017 genome-wide analysis using modern Total XV and Total XVI study cohorts,^4^ the *PNPLA3* I148M variant (rs738409) was shown to be associated with elevated ALT levels after induction therapy. In addition to associations with ALL treatment-related hepatoxicity, *UGT1A1* variants are associated with bilirubin level, and *PNPLA3* variants are associated with ALT and AST levels within the general population at baseline.^5,6,7^ These findings suggest that genetic factors that influence healthy liver function can also have effects on drug-induced changes in liver function in patients with ALL. Pharmacogenomics studies often are limited by the number of cases of the drug-induced phenotype of interest and therefore underpowered. One possible remedial approach is, when a phenotype is shared between a drug effect and a disease, prioritizing known disease-associated variants may help overcome this limitation. Herein we demonstrate the feasibility of this approach.

The purpose of this study was to replicate previously identified genetic factors and discover novel genetic factors associated with hepatotoxic effects during pediatric ALL treatment. We first identified candidate variants by mining an available public large-scale genome-wide association study (GWAS) repository^8^ and tested their associations with hepatotoxic effects in 2 large multi-institutional Children’s Oncology Group (COG) trials, AALL0232 (n = 2283)^9^ and AALL0434 (n = 1274).^10^ Subsequently, we undertook a complementary gene-agnostic approach by performing a GWAS in our cohorts to discover novel genetic variant associations.

## Methods

### Patients

Patients with newly diagnosed ALL were enrolled in the COG AALL0232^9^ and AALL0434^10^ protocols, which accrued data from December 29, 2003, to January 21, 2011 (AALL0232), and from January 22, 2007, to July 24, 2014 (AALL0434), and both of which used augmented Berlin-Frankfurt-Münster treatment regiments.^11,12^ Written informed consent from the parents or guardians and assent from the patients, as appropriate, were obtained with oversight by the local institutional review boards, all of which approved the study. This study followed the Strengthening the Reporting of Genetic Association Studies (STREGA) reporting guideline, an extension of the Strengthening the Reporting of Observational Studies in Epidemiology (STROBE) reporting guideline.

Data were analyzed between January 1, 2019, and April 15, 2022. Toxic effects data were retrieved from protocol databases on May 29, 2019. Toxic effects were graded according to the National Cancer Institute Common Terminology Criteria for Adverse Events, version 4. Only patients with toxic effects of grade 3 or above were considered as cases. Hyperbilirubinemia of grade 3 or above was defined as the bilirubin level above 3.0 times the upper limit of normal. Levels of ALT or AST of grade 3 or above were defined as greater than 5.0 times the upper limit of normal.

Toxic effects were reported within treatment phases (eTable 1 in Supplement 1), including induction, consolidation, interim maintenance, delayed intensification, and maintenance. For those patients who did not have toxic effects reported during the phase, if the patient’s last follow-up time was later than the day that at least 80% toxicity events were reported during the treatment phase, then they were assumed to not have had any toxicity events for the whole phase and were considered a control; otherwise, the patient was treated as missing a value for the phase and subsequent treatment phases. Only the first delayed intensification or interim maintenance phase was considered. Total duration of multiagent protocol chemotherapy ranged from 2.5 to 3.5 years.

### Genotyping

At the end of the induction phase, DNA were extracted and genotyped using a mapping array (Affymetrix SNP6; Thermo Fisher Scientific) for the AALL0232 cohort and an exome array (Infinium 2.5 Omni-exome array; Illumina) for the AALL0434 cohort.^13,14^ Variants were filtered by Hardy-Weinberg equilibrium (*P* > .0001) and a call rate of greater than 95%; variants with minor allele frequency of at least 3% were used for association analysis. Imputation was performed using a National Institutes of Health TOPMed Imputation server. Genetic ancestry was included as a covariate in the genetic association analysis to reduce false discovery attributable to population stratification. Genetic ancestry assignments are provided in the eMethods in Supplement 1.

### Candidate Single-Nucleotide Variants and Polygenetic Risk Scores 

The GWAS catalog was surveyed on September 30, 2021, using the terms *bilirubin*, *liver enzyme*, *alanine aminotransferase (ALT)*, *aspartate aminotransferase (AST)*, and *liver injuries* (eTable 2 in Supplement 1). Single-nucleotide variants (SNVs; formerly single-nucleotide polymorphisms [SNPs]) with *P* values reaching genome-wide significance (*P* = 5 × 10^−8^) were selected as candidate SNVs (n = 576). Note that in this article, *SNV* is used to describe variants occurring in at least 3% of the population and does not include somatic events.

Clinical Pharmacogenetics Implementation Consortium (CPIC) level A/B genes (ie, clinically actionable genes) that could have implications for drugs used in the treatment of ALL were selected, including *CYP3A5* (OMIM 605325), *CYP2B6* (OMIM 123930), *CYP2C9* (OMIM 601130), *CYP2C19* (OMIM 124020), *CYP2D6* (OMIM 124030), *DPYD* (OMIM 612779), *G6PD* (OMIM 305900), *SLCO1B1* (OMIM 604843), *TPMT* (OMIM 187680), and *NUDT15* (OMIM 615792). The list of variants in the CPIC genes were extracted from the CPIC website^15^ on September 30, 2021, and a total of 122 SNVs were tested in our cohorts.^16^ To assess the additive effect of multiallele contribution to the phenotype and evaluate performance, we calculated polygenic risk scores (PRS)^7^ as described in the eMethods in Supplement 1.

### Statistical Analysis

Both hyperbilirubinemia and elevated ALT and AST levels were treated as repeated binary phenotypes. Incidences of toxic effects were estimated using a Kaplan-Meier curve with the first event as time of onset. Patients with relapse or other outcome events were treated as censored at time of events. To account for subgroup effects between the 2 treatment protocol cohorts, models were fitted in the 2 cohorts separately for all genetic association analyses, and the statistics were combined in a meta-analysis using the Stouffer method.^17^ Details of statistical testing techniques and controls for multiple testing are described in the eMethods in Supplement 1. *P* < .05 indicated statistical significance; all *P* values are 2 sided unless indicated otherwise.

## Results

Between the AAL0232 (n = 2283) and AAL0434 (n = 1274) cohorts, 3557 participants were included in the study with an overall median age of 11.1 (range, 1-30) years; 1378 participants (38.7%) were female and 2179 (61.3%) were male. The patient population included 1474 participants (41.4%) of non-European genetic ancestry, including 284 of African ancestry (8.0%), 781 of admixed American and Latino ancestry (22.0%), 142 of Asian ancestry (4.0%), and 267 other ancestry, including patients who could not be clustered with aforementioned ancestry groups (7.5%).

### Incidence of Hepatotoxic Effects and Clinical Risk Factors

**Figure 1.**
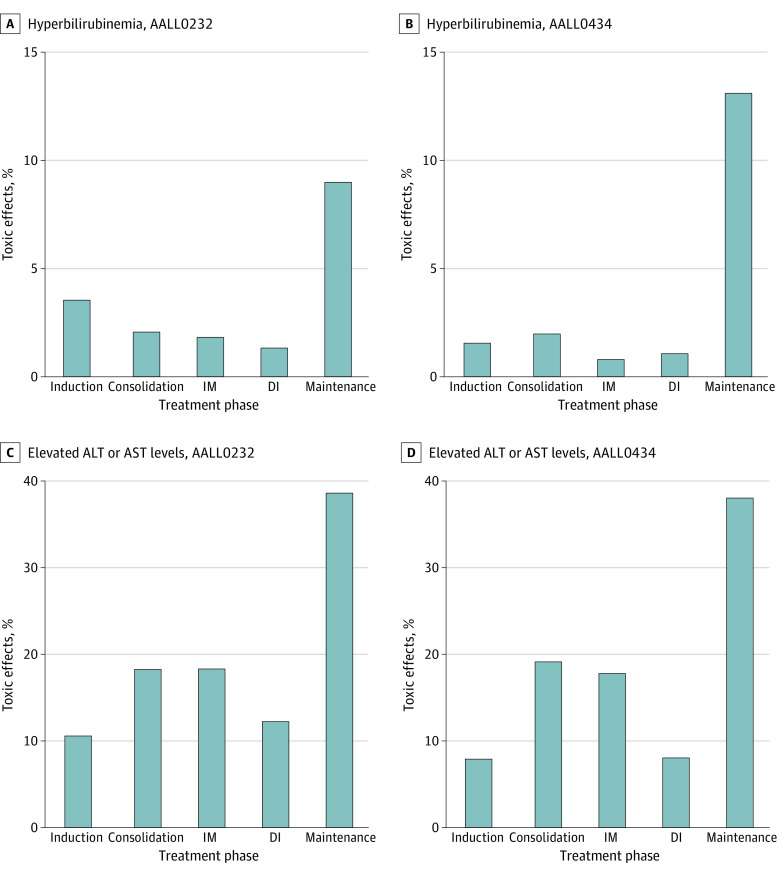
Proportion of Patients With Hepatotoxic Effects by Protocol and Treatment Phases Protocols include the AALL0232 and AALL0434 cohorts. ALT indicates alanine aminotransferase; AST, aspartate aminotransferase; DI, delayed intensification; and IM, interim maintenance.

The cumulative incidence of hyperbilirubinemia within the first 2 years of therapy was 11.4% (range, 10.4%-12.4%) in the AALL0232 and 10.1% (range, 8.6%-11.4%) in AALL0434 cohorts (eFigure 1 in Supplement 1). The percentage of patients who experienced hyperbilirubinemia was the highest during maintenance therapy in both cohorts (157 of 1757 [9.0%] in AALL0232 and 98 of 749 [13.1%] in AALL0434) (Figure 1 and eFigure 2 in Supplement 1). Because transfusions and severe infections are rarer during maintenance therapy, this finding suggests that these elevations are more likely chemotherapy related.

The cumulative incidence of elevated ALT and AST levels within the first 2 years of therapy was estimated to be 48.4% (range, 46.8%-49.9%) in the AALL0232 cohort and 45.7% (range, 43.3%-47.9%) in the AALL0434 cohort (eFigure 1 in Supplement 1). The percentage of patients experiencing elevated ALT and AST levels was the highest during maintenance therapy in both cohorts (689 of 1785 [38.6%] in the AALL0232 cohort and 289 of 770 [37.5%] in the AALL0434 cohort) (Figure 1). Consolidation (374 of 2187 [17.1%] in the AAL0232 protocol and 186 of 973 [19.1%] in the AALL0434 protocol) and interim-maintenance (374 of 2044 [18.3%] in the AAL0232 protocol and 158 of 889 [17.8%] in the AALL0434 protocol) phases were the next 2 treatment phases with the highest ALT and AST levels.

In the AALL0232 cohort, older age was associated with both hyperbilirubinemia (odds ratio [OR], 4.91 [95% CI, 3.37-7.14]; *P* < 1 × 10^−16^) and elevated ALT and AST levels (OR, 1.41 [95% CI, 1.18-1.68]; *P* = .0002) (Table 1). Patients with American Indian ancestry (ie, admixed American and Latino ancestry, mostly corresponding to self-reported Hispanic ancestry) had higher bilirubin (OR, 1.61 [95% CI, 1.19-2.16]; *P* = .002) and ALT and AST (OR, 1.57 [95% CI, 1.29-1.91]; *P* = 6.9 × 10^−6^) levels compared with patients of European ancestry, while African ancestry was associated with lower incidence of elevated ALT and AST levels (OR, 0.34 [95% CI, 0.22-0.54]; *P* = 6.4 × 10^−6^) and nominally lower incidence of hyperbilirubinemia (OR, 0.41 [95% CI, 0.17-0.93]; *P* = .03).

**Table 1.** Hepatotoxic Effects by Demographic Characteristics and Treatment Groups in the AALL0232 and AALL0434 Cohorts^a^

Characteristic	No. of patients	Hyperbilirubinemia		Elevated ALT and AST levels	
		OR (95% CI)	*P* value	OR (95% CI)	*P* value
**AALL0232 cohort**					
No. of patients	2283	NA	NA	NA	NA
Age at diagnosis, y					
<10	817	1 [Reference]	NA	1 [Reference]	NA
≥10	1466	4.91 (3.37-7.14)	<1 × 10^−16^	1.41 (1.18-1.68)	.0002
Sex					
Male	1244	1 [Reference]	NA	1 [Reference]	NA
Female	1039	0.99 (0.92-1.07)	.67	0.86 (0.73-1.01)	.07
Genetic ancestry					
European	1293	1 [Reference]	NA	1 [Reference]	NA
African	127	0.41 (0.17-0.93)	.03	0.34 (0.22-0.54)	6.4 × 10^−6^
Admixed American and Latino	594	1.61 (1.19-2.16)	.002	1.57 (1.29-1.91)	6.9 × 10^−6^
Asian	72	1.80 (0.92-3.47)	.088	1.58 (0.98-2.54)	.06
Other^b^	197	0.84 (0.51-1.40)	.51	1.11 (0.84-1.47)	.49
Treatment randomization					
Dexamethasone and Capizzi methotrexate	413	1 [Reference]	NA	1 [Reference]	NA
Dexamethasone and high-dose methotrexate	459	1.04 (0.68-1.59)	.86	1.07 (0.82-1.39)	.64
Prednisone and Capizzi methotrexate	701	0.81 (0.56-1.18)	.18	0.97 (0.76-1.23)	.79
Prednisone and high-dose methotrexate	710	0.67 (0.43-1.04)	.07	1.08 (0.85-1.38)	.53
**AALL0434 cohort**					
No. of patients	1274	NA	NA	NA	NA
Age at diagnosis, y					
<10	682	1 [Reference]	NA	1 [Reference]	NA
≥10	592	1.95 (1.34-2.84)	.0005	0.94 (0.74-1.20)	.62
Sex					
Male	935	1 [Reference]	NA	1 [Reference]	NA
Female	339	0.96 (0.64-1.45)	.86	0.98 (0.74-1.30)	.90
Genetic ancestry					
European	790	1 [Reference]	NA	1 [Reference]	NA
African	157	0.69 (0.36-1.32)	.26	0.40 (0.26-0.63)	5.5 × 10^−5^
Admixed American and Latino	187	1.30 (0.79-2.13)	.30	1.19 (0.84-1.67)	.33
Asian	70	1.75 (0.88-3.48)	.11	1.43 (0.85-2.43)	.18
Other^b^	70	1.12 (0.48-2.58)	.80	1.71 (0.99-2.93)	.05
Treatment randomization					
Capizzi methotrexate and nelarabine	125	1 [Reference]	NA	1 [Reference]	NA
Capizzi methotrexate	313	1.19 (0.66-2.13)	.57	1.11 (0.74-1.66)	.63
High-dose methotrexate and nelarabine	192	0.97 (0.62-1.53)	.90	1.07 (0.78-1.45)	.68
High-dose methotrexate	387	0.95 (0.54-1.67)	.85	1.26 (0.86-1.85)	.24
Induction only	257	4.66 (1.77-12.30)	.002	2.64 (1.53-4.55)	.0005

Abbreviations: NA, not applicable; OR, odds ratio.

^a^ Odds ratio and *P* values were based on mixed-effects logistic regression. Numbers of patients used in the mixed-effects model are shown.

^b^ Indicates admixed ancestry category for which the genetic ancestry did not fit into the specified categories. The method to determine ancestry and cutoffs is described in the eMethods in Supplement 1.

In the AALL0434 cohort, older age was associated with hyperbilirubinemia (OR, 1.95 [95% CI, 1.34-2.84]; *P* = .0005) (Table 1), but not with ALT and AST levels (OR, 0.94 [95% CI, 0.74-1.20]; *P* = .62). As in the AALL0232 cohort, African ancestry was associated with lower ALT and AST levels compared with European ancestry (OR, 0.40 [95% CI, 0.26-0.63]; *P* = 5.5 × 10^−5^). Admixed American and Latino ancestry was not associated with ALT, AST, or bilirubin levels. Randomized treatment group and sex were not associated with hepatotoxic effects in either cohort (Table 1).

### Association Between Hepatotoxic Effects and Known Genetic Variants

**Figure 2.**
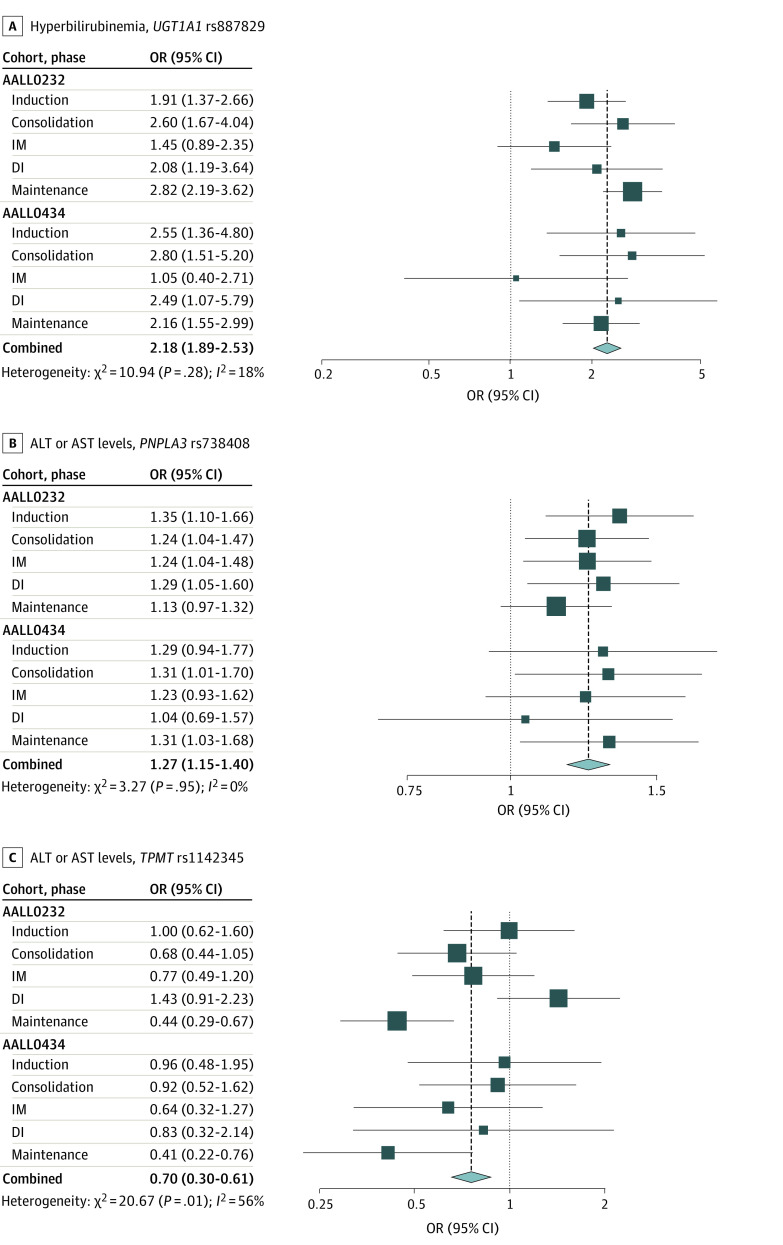
Top Single-Nucleotide Variants (SNVs) From Genome-Wide Association Study Catalog and Clinical Pharmacogenetics Implementation Consortium Protocols include the AALL0232 and AALL0434 cohorts. ALT indicates alanine aminotransferase; AST, aspartate aminotransferase; DI, delayed intensification; and IM, interim maintenance.

Between reported variants associated with hepatic function from the GWAS catalog and CPIC level A/B genes, we tested 698 variants. Hyperbilirubinemia was associated with *UGT1A1* rs887829 after adjusting for multiple testing (OR, 2.18 [95% CI, 1.89-2.53]; meta-analysis *P* = 6.7 × 10^−27^; adjusted *P* < .002) (Table 2) based on a mixed-effects logistic model adjusting for genetic ancestry and age. Variant rs887829 is in high linkage disequilibrium (LD) with the *UGT1A1* promoter (TA)n variant (*28; rs8175347) across populations. The rs887829–T allele, which is in LD with (TA)7/8, was associated with higher risk of hyperbilirubinemia in the AALL0232 cohort (OR, 2.18 [95% CI, 1.83-2.59]; *P* = 3.1 × 10^−18^) and the AALL0434 cohort (OR, 2.20 [95% CI, 1.67-2.89]; *P* = 1.43 × 10^−8^). No significant interaction between rs887829 and treatment phase was observed (adjusted *P* = .25). Patients with the rs887829–T allele were more likely to have hyperbilirubinemia across all phases in both cohorts (Figure 2A). No other candidate variants tested were statistically significant after multiple testing adjustment for either the main effect or genotype × treatment phase interaction.

**Table 2.** Top SNVs Associated With Hepatic Toxic Effects^a^

Toxic effect	Gene	SNV ID (dbSNV)	Effect allele	Allele frequency by ancestry^b^	Treatment phase	AALL0232 cohort		AALL0434 cohort		Combined cohort			
						OR (95% CI)^c^	*P* value	OR (95% CI)^c^	*P* value				
										Meta-analysis *P* value^d^	Adjusted *P* value^e^	*P* value for genotype by phase interaction	Adjusted *P* value^e^
Hyperbilirubinemia	*UGT1A1*	chr2:233759924:C:T (rs887829)	T	0.379, 0.493, 0.130, 0.298, 0.437	Nonspecific	2.18 (1.89-2.53)	3.1 × 10^−18^	2.20 (1.67-2.89)	1.43 × 10^−8^	6.7 × 10^−27^	<.002	.02	.25
Elevated ALT and AST	*PNPLA3*	chr22:43928847:C:G (rs738409)	G	0.484, 0.118, 0.350, 0.226, 0.246	Nonspecific	1.26 (1.12-1.41)	.0001	1.29 (1.07-1.55)	.007	3.72 × 10^−7^	<.002	.39	.78
Elevated ALT and AST	*TPMT*	chr6:18130687:T:C (rs1142345)	C	0.058, 0.067, 0.022, 0.029, 0.017	Nonspecific	0.72 (0.54-0.97)	.029	0.67 (0.45-1.00)	.051	.003	.16	2.38 × 10^−5^	.02
					Maintenance	0.44 (0.29-0.67)	.0001	0.41 (0.22-0.76)	.005	2.2 × 10^−6^	<.002	NA	NA
Elevated ALT and AST^f^	Near *IGHMBP2*, *CPT1A*	chr11:68993081:C:T (rs12283870)	T	0.499, 0.258, 0.122, 0.270, 0.226	Nonspecific	1.29 (1.16-1.44)	5.5 × 10^−6^	1.28 (1.14-1.44)	4.7 × 10^−5^	8.7 × 10^−10^		NA	NA

Abbreviations: ALT, alanine aminotransferase; AST, aspartate aminotransferase; NA, not applicable; OR, odds ratio; SNV, single-nucleotide variant.

^a^ Logistic random-effects models were used for estimating the SNV effect across the whole treatment, including multiple treatment phases.

^b^ Allele frequencies are based on 1000 Genomes Project. Ancestry includes admixed American and Latino, African, East Asian, European, South Asian.

^c^ Odds ratio is associated with an additional copy of effect allele.

^d^ Meta-analysis *P* values were generated using the Stouffer method.^17^

^e^ Computed based on permutation test (n = 500), considering 617 SNVs were tested.

^f^ Includes patients younger than 10 years only.

*PNPLA3* rs738409 (I148M) was associated with elevated ALT and AST levels after adjusting for multiple testing (OR, 1.27 [95% CI, 1.15-1.40]; meta-analysis *P* = 3.72 × 10^−7^; adjusted *P* < .002). The G allele of rs738409 was associated with higher ALT and AST levels in both the AALL0232 (OR, 1.26; [95% CI, 1.12-1.41]; *P* = .0001) and AALL0434 (OR, 1.29 [95% CI, 1.07-1.55]; *P* = .007) cohorts. No interaction between *PNPLA3* genotype and treatment phase was observed (adjusted *P* = .78) (Figure 2B). A significant interaction between *TPMT* rs1142345 (Y240C) and treatment phase was observed (meta-analysis *P* = 2.38 × 10^−5^; adjusted *P* = .02). The C allele represents *TPMT* *3C and was only associated with lower incidence of elevated ALT and AST levels during maintenance phase in the AALL0232 (OR, 0.44 [95% CI, 0.29-0.67]; *P* = .0001) and AALL0434 (OR, 0.41 [95% CI, 0.22-0.76]; *P* = .005) cohorts (OR, 0.43 [95% CI, 0.30-0.61]; meta-analysis *P* = 2.2 × 10^−6^), but not in other treatment phases (*P* > .05) (Figure 2C). TPMT rs1800460 (*3B) is in strong LD with rs1142345 and also was associated with elevated ALT and AST levels, but not after multiple testing adjustment (eTable 2 in Supplement 1). No other additional candidate variants reached significance for ALT and AST toxic effects after adjusting for *PNPLA3* and *TPMT* variants.

We generated PRS of 46 blood and urine biomarkers based on UK Biobank data.^5^ Polygenic risk scores for direct bilirubin and ALT levels were associated with hyperbilirubinemia (OR, 1.11 [95% CI, 1.09-1.14]; meta-analysis *P* = 5.07 × 10^−23^) and elevated ALT and AST levels (OR, 1.08 [95% CI, 1.05-1.11]; meta-analysis *P* = 1.2 × 10^−5^) in our cohorts) (eFigure 3 in Supplement 1). After adjusting for *UGT1A1* rs887829, the direct bilirubin PRS was no longer significant (*P* = .41). Interestingly, the ALT PRS remained associated with ALT and AST levels even after adjusting for the *PNPLA3* rs738409 variant (OR, 1.06 [95% CI, 1.02-1.10]; meta-analysis *P* = .001) (eTables 3 and 4 in Supplement 1). Because the UK Biobank data set is composed primarily of participants of European ancestry, we also performed a PRS analysis in only patients of European ancestry, and the above associations remained, with similar effect size estimates (eTables 3 and 4 in Supplement 1).

### Genome-Wide Association Analysis of Hepatotoxic Effects

**Figure 3.**
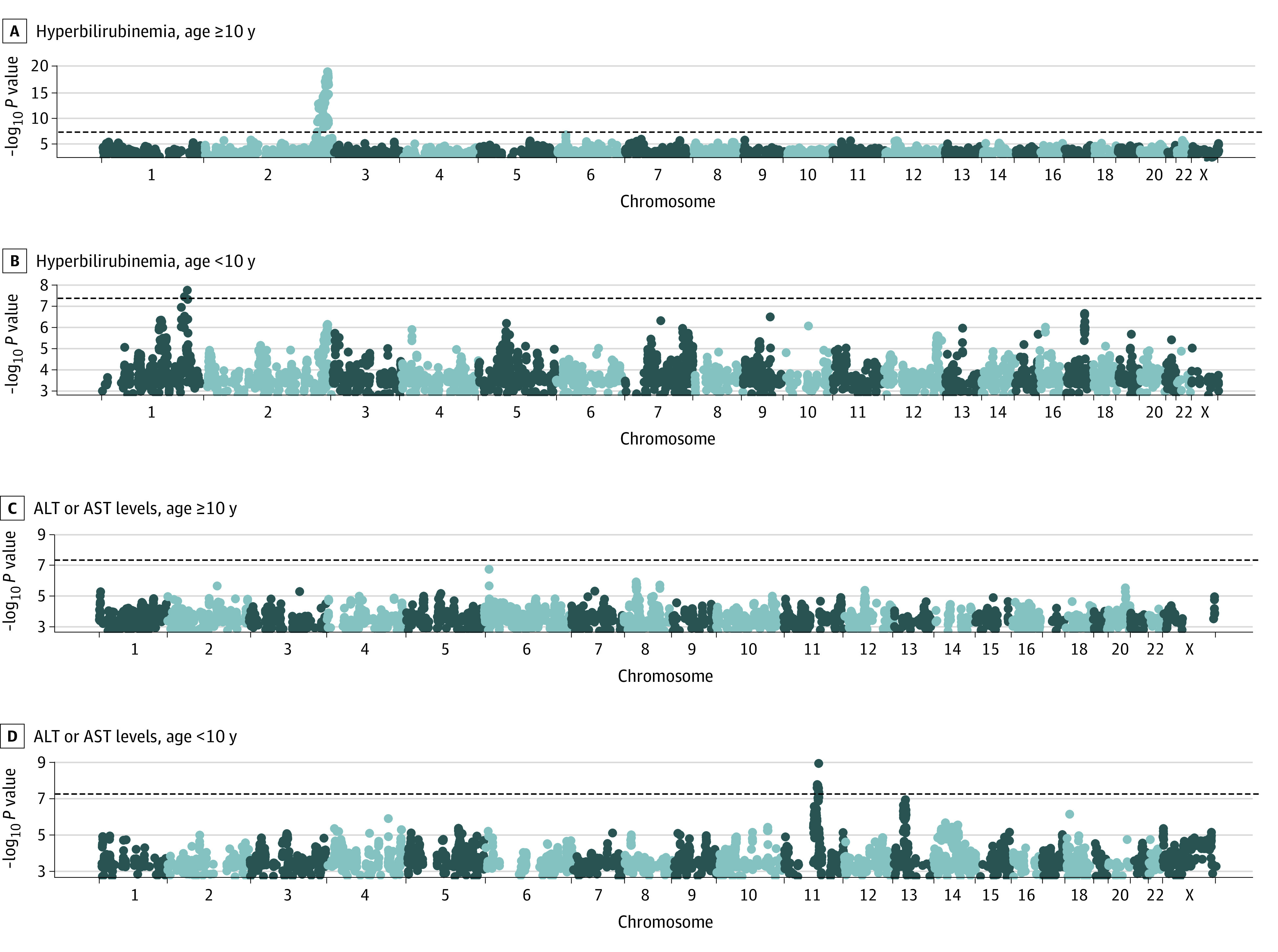
Genome-Wide Association Study Analyses of Hyperbilirubinemia and Elevated Alanine Aminotransferase (ALT) and Aspartate Aminotransferase (AST) Levels Stratified by Age

To discover novel genetic associations with hepatotoxic effects, we performed genome-wide analyses within each cohort adjusting for age, treatment group, and genetic ancestry, and then performed meta-analysis using the Stouffer method.^17^ *UGT1A1* was the only locus reaching genome-wide significance for hyperbilirubinemia (eFigure 4 in Supplement 1), with the top variant being rs887829. We also performed conditional analysis adjusting for *UGT1A1* rs887829, and the locus zoom plot showed it was the only significant signal at this locus (eFigure 5 in Supplement 1). Because age was associated with hyperbilirubinemia, we performed genome-wide analysis within subcohorts of patients younger than 10 years or 10 years and older (Figure 3A). *UGT1A1* rs887829 continued to be significant in the age-stratified analysis for patients younger than 10 years (OR, 1.10 [95% CI, 1.06-1.14]; meta-analysis *P* = 3.64 × 10^−7^) vs patients 10 years or older (OR, 1.27 [95% CI, 1.20-1.34]; meta-analysis *P* = 4.4 × 10^−19^). No additional variants reached genome-wide significance in the age-stratified analysis.

For elevated ALT and AST levels, adjusting for age and genetic ancestry, no variants reached genome-wide significance (eFigure 4 in Supplement 1) in the full cohorts. When analyzed among patients younger than 10 years, a variant rs12283870 on chromosome 11 reached genome-wide significance (OR, 1.28 [95% CI, 1.18-1.39]; meta-analysis *P* = 8.7 × 10^−10^) (Table 2 and Figure 3D). The T allele of rs12283870 was associated with higher risk of ALT and AST toxicity among younger patients in the AALL0232 (OR, 1.29 [95% CI, 1.16-1.44]; *P* = 5.5 × 10^−6^) and AALL0434 (OR, 1.28 [95% CI, 1.14-1.44]; *P* = 4.7 × 10^−5^) cohorts (eFigure 6 in Supplement 1). No genome-wide significant variants were observed among patients 10 years or older, including rs12283870 in the AALL0232 (OR, 0.93 [95% CI, 0.85-1.02]; *P* = .11) and AALL0234 (OR, 1.07 [95% CI, 0.94-1.22]; *P* = .33) cohorts (eFigure 6 in Supplement 1).

## Discussion

In this genome-wide association study, hepatic toxic effects were common in both pediatric B-cell lineage (AALL0232) and T-cell lineage (AALL0434) ALL, and over 40% of patients experienced elevated ALT and AST levels over the first 2 years of treatment. Age was associated with hyperbilirubinemia in both cohorts and with elevated ALT and AST levels in the AALL0232 cohort. The incidence of both hepatotoxic effects also differed among ancestry groups, suggesting potential underlying genetic factors. By leveraging the power from existing common disease GWAS, we were able to apply a less stringent significance threshold and improve our power for discovering biologically relevant variants that were associated with phenotypes, despite our limited sample size. For example, the *PNPLA3* rs738409 variant was not significant genome-wide in our cohort (eFigure 7 in Supplement 1), but it could be discovered even after multiple testing adjustments by focusing on candidate SNVs from the GWAS catalog of relevant phenotypes. This replicated the association between rs738409 and hepatotoxic effects reported in a new independent cohort.^4^ Our findings indicate the benefit of leveraging common disease findings in future genetic association discovery studies of drug toxicities in ALL.

We observed a genome-wide significant SNV that was associated with elevated ALT and AST levels in younger patients. The SNV rs12283870 is located 89 kilobase (kb) downstream of *IGHMBP2* and 148.6 kb upstream of the *CPT1A* gene. From GTEx, version 8, rs12283870 is an expression quantitative trait locus for *IGHMBP2* and a splice quantitative trait locus for *CPT1A* in multiple tissues (eFigure 8 in Supplement 1). *CPT1A* encodes carnitine palmitoyltransferase 1, a key enzyme in transferring the acyl group of long-chain fatty acid–coenzyme A conjugates onto carnitine. It has been proposed that l-carnitine be incorporated into ALL treatment to reduce hepatotoxic effects in adult ALL.^18,19,20^ However, in a mouse model, it did not reduce asparaginase-associated hepatotoxic effects.^21^ *IGHMBP2* was initially discovered to bind to the immunoglobulin mu chain switch (S mu) region,^22^ but its function related to IgM has not been well established. There is conflicting evidence whether *IGHMBP2* is associated with IgA nephropathy.^23,24^ Polyethylene glycol conjugated (PEGylated) preparations of asparaginase (pegasparagase) were used in both AALL0232 and AALL0434,^25^ and presence of anti-PEG IgM has been shown to accelerate blood clearance of the PEGylated products.^26,27^ It is possible that rs12283870 influences hepatic toxicity by modifying exposure to asparaginase.

By interrogating the interaction between genotypes and treatment phase, the likely drugs implicated with these genetic variants may be identified. We showed that the *TPMT* genotype was only associated with elevated ALT and AST levels during the maintenance phase, a period when mercaptopurine exposure is continuous for more than 1 year. TPMT intermediate and poor metabolizers were reported to have lower ALT levels, which could be due to lower concentrations of hepatotoxic methylated thiopurine metabolites.^28,29^

Patients of African ancestry had a lower incidence of hyperbilirubinemia, even though they had a higher risk allele frequency for *UGT1A1* rs887829 and a lower frequency of the *PNPLA3* risk allele; thus, we were unable to identify a genetic basis for their lower hyperbilirubinemia. Hispanic patients had a higher frequency of the *PNPLA3* rs738409 risk allele, which likely contributes to their higher ALT and AST levels. Furthermore, in the multivariable analysis with both ancestry and genotype, Hispanic patients continued to a have higher risk of elevated ALT and AST levels in the AALL0232 cohort (eTable 5 in Supplement 1). These findings suggest a genetic basis for racial and ethnic differences in hepatotoxic effects and further contribute to a growing body of evidence for racial and ethnic disparities in childhood ALL.^30^

### Strengths and Limitations

By using both a candidate gene and genome-wide approach in an ancestrally diverse study population, we validated previous associations found in other cohorts^3,4^ and identified novel loci associated with hepatotoxic effects during pediatric ALL treatment. Our novel discovery of an association of rs12283870 on chromosome 11 with elevated ALT and AST levels in younger patients needs to be replicated in additional cohorts. We also discovered a new interaction between the maintenance phase and *TPMT* *3C protective effect on ALT and AST levels that has biological plausibility.

This study also has some limitations. Because patients were enrolled in prospective interventional clinical trials that use contemporary multimodal ALL therapy, definitive analysis of individual drug effects was not possible, and therefore treatment phase was used as a surrogate for differential drug effects. As ALL therapy evolves, future studies may elucidate drug-specific effects. Although we found that genetic risk factors for elevated bilirubin, ALT, and AST levels were also risk factors for treatment-associated elevations in these biomarkers, conflicting findings were reported for nonalcoholic fatty liver disease–associated SNVs and drug-induced liver injury.^31^ The PRS used in our study was derived from older adults and therefore may have performance limitations; however, it was still associated with elevated bilirubin, ALT, and AST levels in this pediatric cohort. Finally, we did not have access to nonalcoholic fatty liver disease data that may partially explain the association between ALT and AST levels and *PNPLA3* rs738409.

## Conclusions

The findings of this genetic association study suggest that *UGT1A1* and *PNPLA3* were associated with increased risk of hyperbilirubinemia and elevated ALT and AST levels in independent multinational and ancestrally diverse cohorts treated for ALL. We also identified novel associations and interactions among age, treatment phase, and genotype and found that disease-risk alleles can inform candidate SNVs for treatment-related phenotypes. Our results suggest a strong genetic basis for interpatient variability in bilirubin, ALT, and AST levels during ALL therapy.

## Notes

Supplement 1. **eMethods.** Data Analysis **eTable 1.** Treatment Phase and Protocol **eTable 2.** Association Between GWAS Catalog or CPIC Level A/B Variants With Hyperbilirubinemia or Elevated ALT and AST **eTable 3.** Top PRS From PRS Catalog **eTable 4.** Association Between Liver Function–Related PRS With Hyperbilirubinemia or Elevated ALT and AST in Combined Cohort or European Ancestry–Only Cohort **eTable 5.** Multivariable Analysis Including Both Ancestral Group and Genotypes **eFigure 1.** Cumulative Incidence of Hepatotoxic Effects in AALL0232 and AALL0434 **eFigure 2.** Proportion With Toxic Effects by Age **eFigure 3.** Forest Plot of the Association Between Top PRS With Hyperbilirubinemia (A) and Elevated ALT and AST (B) **eFigure 4.** GWAS of Hyperbilirubinemia and Elevated ALT and AST **eFigure 5.** Locus Zoom for *UGT1A1* and Hyperbilirubinemia Based on Results From Meta-analysis Combining Both AALL0232 and AALL0434 **eFigure 6.** Association of rs12283870 Genotypes and Elevated ALT and AST by Age and Treatment Phase **eFigure 7.** Locus Zoom for *PNPLA3* and Elevated ALT and AST Based on Results From Meta-analysis Combining Both AALL0232 and AALL0434 **eFigure 8.** Locus Zoom for rs12283870 and Elevated ALT and AST (Age < 10 y) Based on Results From Meta-analysis Combining Both AALL0232 and AALL0434
eReferences

Supplement 2.
Data Sharing Statement
