# Supplementary material for: Association of Inherited Genetic Factors With Drug-Induced Hepatic Damage Among Children With Acute Lymphoblastic Leukemia
Source: JAMA Netw Open. 2022 Dec 29;5(12):e2248803. doi: 10.1001/jamanetworkopen.2022.48803 (PMC9857512; doi:10.1001/jamanetworkopen.2022.48803)
Supplement: Supplement 1. — eMethods. Data Analysis eTable 1. Treatment Phase and Protocol eTable 2. Association Between GWAS Catalog or CPIC Level A/B Variants With Hyperbilirubinemia or Elevated ALT and AST eTable 3. Top PRS From PRS Catalog eTable 4. Association Between Liver Function–Related PRS With Hyperbilirubinemia or Elevated ALT and AST in Combined Cohort or European Ancestry–Only Cohort eTable 5. Multivariable Analysis Including Both Ancestral Group and Genotypes eFigure 1. Cumulative Incidence of Hepatotoxic Effects in AALL0232 and AALL0434 eFigure 2. Proportion With Toxic Effects by Age eFigure 3. Forest Plot of the Association Between Top PRS With Hyperbilirubinemia (A) and Elevated ALT and AST (B) eFigure 4. GWAS of Hyperbilirubinemia and Elevated ALT and AST eFigure 5. Locus Zoom for UGT1A1 and Hyperbilirubinemia Based on Results From Meta-analysis Combining Both AALL0232 and AALL0434 eFigure 6. Association of rs12283870 Genotypes and Elevated ALT and AST by Age and Treatment Phase eFigure 7. Locus Zoom for PNPLA3 and Elevated ALT and AST Based on Results From Meta-analysis Combining Both AALL0232 and AALL0434 eFigure 8. Locus Zoom for rs12283870 and Elevated ALT and AST (Age < 10 y) Based on Results From Meta-analysis Combining Both AALL0232 and AALL0434 eReferences [file jamanetwopen-e2248803-s001.pdf]

Supplementary Online Content

Yang W, Karol SE, Hoshitsuki K, et al. Association of inherited genetic factors with drug-induced hepatic damage among children with acute lymphoblastic leukemia. *JAMA Netw Open*. 2022;5(12):e2248803. doi:10.1001/jamanetworkopen.2022.48803

eMethods. Data Analysis
eTable 1. Treatment Phase and Protocol
eTable 2. Association Between GWAS Catalog or CPIC Level A/B Variants With Hyperbilirubinemia or Elevated ALT and AST
eTable 3. Top PRS From PRS Catalog
eTable 4. Association Between Liver Function–Related PRS With Hyperbilirubinemia or Elevated ALT and AST in Combined Cohort or European Ancestry–Only Cohort
eTable 5. Multivariable Analysis Including Both Ancestral Group and Genotypes
eFigure 1. Cumulative Incidence of Hepatotoxic Effects in AALL0232 and AALL0434
eFigure 2. Proportion With Toxic Effects by Age
eFigure 3. Forest Plot of the Association Between Top PRS With Hyperbilirubinemia (A) and Elevated ALT and AST (B)
eFigure 4. GWAS of Hyperbilirubinemia and Elevated ALT and AST
eFigure 5. Locus Zoom for *UGT1A1* and Hyperbilirubinemia Based on Results From Meta-analysis Combining Both AALL0232 and AALL0434
eFigure 6. Association of rs12283870 Genotypes and Elevated ALT and AST by Age and Treatment Phase
eFigure 7. Locus Zoom for *PNPLA3* and Elevated ALT and AST Based on Results From Meta-analysis Combining Both AALL0232 and AALL0434
eFigure 8. Locus Zoom for rs12283870 and Elevated ALT and AST (Age < 10 y) Based on Results From Meta-analysis Combining Both AALL0232 and AALL0434
eReferences

This supplementary material has been provided by the authors to give readers additional information about their work.

© 2022 Yang W et al. *JAMA Network Open*.

eMethods

Genome-wide genotypes were imputed using TOPMED imputation server using the TOPMED population as reference.[1] Genetic ancestries for individuals were estimated by iAdmix using 1000genome population as reference.[2,3] For reporting ancestry-specific hazard ratios, patients were grouped into European (%European > 90%), African (%African > 60%), Asian (%EAS+%SAS > 90%), Admixed American/Latino (%American Indian> 10%) and other Admixed population. Principal components were generated using genome-wide SNPs and used to control for population stratification in the association studies.

To evaluate the association between single nucleotide polymorphism (SNP) genotypes and toxicity, a mixed-effects logistic regression model was fitted within each cohort (i.e., AALL0232 and AALL0434) including age, principal components for genetic ancestry, treatment randomization, and treatment phase as fixed covariates. The mixed-effects model took into consideration that we observed multiple related toxicity phenotypes from each patient over the multiple treatment phases. We also included the interaction term between genotype and treatment phase to examine the heterogenous effect of SNP on toxicity at different treatment phases. Analysis of deviance was used to generate p-values comparing models without SNP versus the model including the SNP genotypes with or without its interaction with treatment phase.

For the analysis using candidate SNPs and PRSs, we used permutation to control for multiple testing. For each permutation, we randomly assigned genotypes and associated principal components to each patient and recalculated the p-value as described above. The adjusted p-values were computed as the frequency of permutations with p-value less or equal to the observed p-value. A total of 500 permutations were performed. Adjusted p-values < 0.05 were considered statistically significant. For genome-wide screening, we used 5e-8 as the threshold of statistical significance. Data analysis was performed using R version 4.02 and PLINK v2.00a3LM. All p-values are two-sided unless specified otherwise.

A PRS provides a single value estimate of an individual's risk for the phenotype by weighting the effect size from GWAS data. To compute PRS, 46 PRS of blood and urine biomarkers defined in catalog PGP000128 based on UK biobank were downloaded from PRS catalog (http://www.pgscatalog.org/publication/PGP000128/) in September 2021.[4] PRSIce2 were used to calculate PRS based on the weights provided from PGS catalog.[5]

© 2022 Yang W et al. *JAMA Network Open*.

eTable 1. Treatment phase and protocol

	AALL0232		AALL0434	
Treatment Phase	Protocol duration	Drugs	Protocol duration	Drugs
Induction (INDU)	35 days w/wo 2 weeks for extended induction	Prednisone or Dexamethasone, PEG-asparaginase, Vincristine, Daunorubicin Cytarabine((IT), Methotrexate(IT)	35 days	Prednisone, PEG-asparaginase, Vincristine, Daunorubicin, Cytarabine(IT), Methotrexate(IT)
Consolidation (CONS)	56 days	Cyclophosphamide, Cytarabine, Mercaptopurine, Vincristine, PEG-asparaginase, Methotrexate (IT)	56 days or 77 days (Nelarabine arms)	Cyclophosphamide, Cytarabine, Mercaptopurine, Vincristine, PEG-asparaginase, Methotrexate(IT), Nelarabine[b]
Interim Maintenance (IM)[a]	56 days	Vincristine, HD-Methotrexate or Capizzi Methotrexate, PEG-Asparaginase (Capizzi arms), Mercaptopurine (HDMTX arms), Methotrexate(IT)	56 days	Vincristine, HD-Methotrexate or Capizzi Methotrexate, PEG-Asparaginase (Capizzi arms), Mercaptopurine (HDMTX arms), Methotrexate(IT)
Delayed Intensification (DI)[a]	56 days	Vincristine, Dexamethasone, Doxorubicin, PEG-asparaginase, Cyclophosphamide, Cytarabine, Methotrexate(IT), Thioguanine	63 days	Vincristine, Dexamethasone, Doxorubicin, PEG-asparaginase, Cyclophosphamide, Cytarabine, Methotrexate(IT), Thioguanine, Nelarabine[b]
Maintenance (MNT)	>=2years	Mercaptopurine, Methotrexate, Prednisone, Vincristine, Methotrexate(IT)	>=2years	Mercaptopurine, Methotrexate, Prednisone, Vincristine, Methotrexate(IT), Nelarabine[b]

[a]only first IM and DI were included; [b]only for patients randomized to Nelarabine arm.

© 2022 Yang W et al. *JAMA Network Open*.

eTable 2. Association between GWAS Catalog or CPIC level A/B variants with hyperbilirubinemia or elevated ALT/AST

Patient Phenotype	SNP	gwascat.min.pvalue	gwascat.allele	gwascat.trait	REF	ALT	EST_AALL0232	OR_AALL0232	PVALUE_AALL0232	EST_AALL0434	OR_AALL0434	PVALUE_AALL0434	meta.p	adj_meta.p	interaction_p	adj_interaction.p
Hyperbilirubinemia	chr2:233759924:C:T	1E-69	rs887829-T	bilirubin levels;total bilirubin levels in hiv-1 infection;bilirubin levels in tenofovir-treated hiv infection	C	T	0.777	2.176	3.10E-18	0.788	2.198	1.4E-08	6.7E-27	< 0.002	0.015027	0.989744
Hyperbilirubinemia	chr2:233759924:C:T	0.000000001	nearGene-5	CPIC:UGT1A1	C	T	0.777	2.176	3.10E-18	0.788	2.198	1.4E-08	6.7E-27	< 0.002	0.015027	0.989744
Hyperbilirubinemia	chr2:233750415:A:C	0.000000002	rs2885296-?	bilirubin levels in tenofovir-treated hiv infection	A	C	0.791	2.206	6.17E-17	0.729	2.074	4.2E-08	3.6E-26	< 0.002	0.240667	1
Hyperbilirubinemia	chr2:233744071:C:T	0.000000005	rs17864701-?	bilirubin levels in tenofovir-treated hiv infection	C	T	0.799	2.224	3.34E-17	0.712	2.039	1.0E-07	4.3E-26	< 0.002	0.259402	1
Hyperbilirubinemia	chr2:233745803:A:G	0.000000003	rs11695484-?	bilirubin levels in tenofovir-treated hiv infection	A	G	0.798	2.221	3.73E-17	0.709	2.032	1.1E-07	5.1E-26	< 0.002	0.260601	1
Hyperbilirubinemia	chr2:233764663:C:T	5E-93	rs4148325-?	bilirubin levels in extreme obesity;bilirubin levels;total bilirubin levels in hiv-1 infection;serum bilirubin levels in metabolic syndrome;bilirubin levels in tenofovir-treated hiv infection	C	T	0.757	2.131	1.69E-15	0.787	2.197	3.7E-09	7.2E-26	< 0.002	0.019973	1
Hyperbilirubinemia	chr2:233757136:G:A	3E-64	rs10929302-A	bilirubin levels;bilirubin levels in tenofovir-treated hiv infection	G	A	0.781	2.184	1.37E-16	0.741	2.097	3.2E-08	7.9E-26	< 0.002	0.266573	1

© 2022 Yang W et al. *JAMA Network Open*.

Hyperbilirubinemia	chr2:233756119:T:C	0.000000002	rs6714634-?	bilirubin levels in tenofovir-treated hiv infection	T	C	0.781	2.184	1.37E-16	0.738	2.091	3.4E-08	8.1E-26	< 0.002	0.268136	1
Hyperbilirubinemia	chr2:233755708:G:A	0.000000002	rs6747843-?	bilirubin levels in tenofovir-treated hiv infection	G	A	0.781	2.185	1.29E-16	0.736	2.088	4.0E-08	9.7E-26	< 0.002	0.272642	1
Hyperbilirubinemia	chr2:233764076:T:G	9E-11	rs4148324-?	bilirubin levels in tenofovir-treated hiv infection	T	G	0.753	2.123	2.37E-15	0.781	2.183	4.5E-09	1.2E-25	< 0.002	0.024718	1
Hyperbilirubinemia	chr2:233741916:T:C	3E-10	rs34352510-?	bilirubin levels in tenofovir-treated hiv infection	T	C	0.792	2.207	6.99E-17	0.699	2.012	1.4E-07	2.0E-25	< 0.002	0.205679	1
Hyperbilirubinemia	chr2:233740656:G:A	0.000000006	rs17862875-?	bilirubin levels in tenofovir-treated hiv infection	G	A	0.796	2.216	5.44E-17	0.690	1.995	2.9E-07	2.4E-25	< 0.002	0.30364	1
Hyperbilirubinemia	chr2:233764593:G:A	1E-10	rs3771341-?	bilirubin levels in tenofovir-treated hiv infection	G	A	0.753	2.123	2.63E-15	0.762	2.143	1.2E-08	8.2E-25	< 0.002	0.208548	1
Hyperbilirubinemia	chr2:233747994:T:C	0.000000006	rs11888459-?	bilirubin levels in tenofovir-treated hiv infection	T	C	0.694	2.003	1.24E-13	0.755	2.128	1.4E-08	3.7E-23	< 0.002	0.381411	1
Hyperbilirubinemia	chr2:233749231:T:A	0.000000006	rs10178992-?	bilirubin levels in tenofovir-treated hiv infection	T	A	0.694	2.003	1.24E-13	0.755	2.128	1.4E-08	3.7E-23	< 0.002	0.381411	1
Hyperbilirubinemia	chr2:233749470:C:T	0.000000006	rs7604115-?	bilirubin levels in tenofovir-treated hiv infection	C	T	0.694	2.003	1.24E-13	0.755	2.128	1.4E-08	3.7E-23	< 0.002	0.381411	1
Hyperbilirubinemia	chr2:233755414:G:T	0.000000004	rs11673726-?	bilirubin levels in tenofovir-treated hiv infection	G	T	0.691	1.995	1.59E-13	0.768	2.155	9.5E-09	4.2E-23	< 0.002	0.411747	1
Hyperbilirubinemia	chr2:233736540:G:T	9E-18	rs7564935-G	bilirubin levels	G	T	0.691	1.995	1.83E-13	0.731	2.076	4.3E-08	1.9E-22	< 0.002	0.4468	1
Hyperbilirubinemia	chr2:233735091:A:T	5E-16	rs4477910-?	total bilirubin levels in hiv-1 infection	A	T	0.690	1.993	3.88E-13	0.755	2.127	2.7E-08	2.1E-22	< 0.002	0.062325	1
Hyperbilirubinemia	chr2:233730664:G:A	1E-41	rs11891311-?	bilirubin levels	G	A	0.683	1.979	4.83E-13	0.692	1.999	2.6E-07	4.0E-21	< 0.002	0.535922	1
Hyperbilirubinemia	chr2:233720033:T:G	0	rs13401281-?	total bilirubin levels	T	G	0.685	1.984	3.87E-13	0.678	1.969	3.8E-07	4.9E-21	< 0.002	0.483129	1

© 2022 Yang W et al. *JAMA Network Open*.

Hyperbilirubinemia	chr2:233765830:A:G	3E-24	rs929596-?	total bilirubin levels in hiv-1 infection;serum bilirubin levels in metabolic syndrome;bilirubin levels in tenofovir-treated hiv infection	A	G	0.719	2.052	6.27E-14	0.651	1.917	2.0E-06	7.0E-21	< 0.002	0.259954	1
Hyperbilirubinemia	chr2:233702448:T:G	1E-22	rs17863787-?	total bilirubin levels in hiv-1 infection;serum bilirubin levels in metabolic syndrome	T	G	0.693	2.000	1.61E-12	0.682	1.978	6.6E-07	4.3E-20	< 0.002	0.019451	1
Hyperbilirubinemia	chr2:233716976:C:T	7E-12	rs1875263-?	total bilirubin levels in hiv-1 infection	C	T	0.635	1.887	2.27E-11	0.659	1.933	1.2E-06	1.5E-18	< 0.002	0.412175	1
Hyperbilirubinemia	chr2:233755003:C:G	0.000000007	rs10929301-?	bilirubin levels in tenofovir-treated hiv infection	C	G	0.611	1.842	8.66E-11	0.612	1.845	8.9E-06	1.3E-17	< 0.002	0.242258	1
Hyperbilirubinemia	chr2:233749337:T:C	0.000000001	rs10179091-?	bilirubin levels in tenofovir-treated hiv infection	T	C	0.615	1.849	4.94E-11	0.571	1.769	2.0E-05	1.4E-17	< 0.002	0.35756	1
Hyperbilirubinemia	chr2:233758936:A:C	9E-20	rs3755319-?	total bilirubin levels in hiv-1 infection	A	C	0.596	1.815	2.46E-10	0.627	1.871	5.8E-06	2.8E-17	< 0.002	0.134132	1
Hyperbilirubinemia	chr2:233688675:A:T	5E-16	rs10173355-?	total bilirubin levels in hiv-1 infection	A	T	0.601	1.824	4.25E-09	0.736	2.088	8.9E-08	2.7E-16	< 0.002	0.003191	0.671795
Hyperbilirubinemia	chr2:233693545:A:G	2E-14	rs2070959-G	serum bilirubin levels in metabolic syndrome	A	G	0.601	1.824	4.32E-09	0.738	2.091	9.8E-08	3.0E-16	< 0.002	0.003545	0.738462
Hyperbilirubinemia	chr2:233746667:C:A	4E-16	rs4663969-?	total bilirubin levels in hiv-1 infection	C	A	0.587	1.799	2.10E-10	0.506	1.659	1.4E-04	9.4E-16	< 0.002	0.889235	1
Hyperbilirubinemia	chr2:233600317:C:T	0.000000002	rs2741012-?	total bilirubin levels in hiv-1 infection	C	T	0.432	1.541	5.88E-05	0.482	1.620	6.6E-04	4.0E-08	< 0.002	0.221501	1
Hyperbilirubinemia	chr2:233609365:G:A	0.000000001	rs2741027-A	serum bilirubin levels in metabolic syndrome	G	A	0.439	1.551	4.36E-05	0.441	1.554	2.3E-03	8.1E-08	< 0.002	0.21467	1
Hyperbilirubinemia	chr2:233762816:G:T	0	rs28946889-?	total bilirubin levels	G	T	-0.443	0.642	1.07E-04	-0.255	0.775	1.1E-01	1.4E-04	0.005	0.566731	1

© 2022 Yang W et al. *JAMA Network Open*.

Hyperbilirubinemia	chr11:78416868:A:G	2E-23	rs869202-A	aspartate aminotransferase levels	A	G	-0.290	0.748	1.69E-02	-0.336	0.715	5.7E-02	3.1E-04	0.47	0.046424	1
Hyperbilirubinemia	chr2:233790144:T:C	7E-23	rs2361502-?	bilirubin levels;serum bilirubin levels in metabolic syndrome;total bilirubin levels in hiv-1 infection	T	C	0.397	1.487	9.95E-05	0.239	1.269	9.9E-02	3.4E-04	0.01	0.777016	1
Hyperbilirubinemia	chr2:233698748:A:C	0.000000005	rs12988520-C	serum bilirubin levels in metabolic syndrome	A	C	0.299	1.348	1.69E-03	0.350	1.419	8.3E-03	4.1E-04	0.015	0.428224	1
Hyperbilirubinemia	chr2:233772999:G:C	4E-20	rs8330-?	total bilirubin levels	G	C	0.306	1.359	8.07E-03	0.399	1.490	1.7E-02	3.7E-03	0.075	0.535365	1
Hyperbilirubinemia	chr2:233760498:G:A	0.000000001	G71R	CPIC:UGT1A1	G	A	1.102	3.011	1.24E-03	0.484	1.623	4.1E-01	4.0E-03	0.595	0.458486	1
Hyperbilirubinemia	chr2:233760498:G:A	3E-139	rs4148323-?	bilirubin levels	G	A	1.102	3.011	1.24E-03	0.484	1.623	4.1E-01	4.0E-03	0.595	0.458486	1
Hyperbilirubinemia	chr2:233759379:A:G	0.00000005	rs6723506-G	bilirubin levels	A	G	0.790	2.203	1.66E-03	0.734	2.083	1.2E-01	4.7E-03	0.65	0.56	1
Hyperbilirubinemia	chr6:18130687:T:C	0.000000001	Y240C;Y240S	CPIC:TPMT	T	C	-0.529	0.589	5.51E-02	-0.883	0.413	4.6E-02	9.6E-03	0.865	0.296684	1
Hyperbilirubinemia	chr22:28333779:A:T	0.00000002	rs134489-A	aspartate aminotransferase levels	A	T	-0.055	0.946	6.21E-01	-0.417	0.659	1.4E-02	1.0E-02	1	0.395234	1
Hyperbilirubinemia	chr11:108566127:A:G	0.00000001	rs2846407-?	alanine aminotransferase levels	A	G	-0.225	0.798	5.76E-02	-0.169	0.845	2.8E-01	1.1E-02	1	0.021066	1
Hyperbilirubinemia	chr2:135839297:G:A	0.00000002	rs6742283-G	aspartate aminotransferase levels	G	A	0.186	1.205	5.62E-02	0.153	1.165	2.7E-01	1.3E-02	1	0.08259	1
Hyperbilirubinemia	chr11:78229029:G:A	5E-10	rs2450128-G	alanine aminotransferase levels	G	A	-0.111	0.895	3.41E-01	-0.202	0.817	2.2E-01	1.5E-02	1	0.106229	1
Hyperbilirubinemia	chr22:28712241:A:G	7E-18	rs5752776-G	alanine aminotransferase levels	A	G	0.075	1.078	4.66E-01	0.265	1.303	8.0E-02	1.7E-02	1	0.665966	1

© 2022 Yang W et al. *JAMA Network Open*.

Hyperbilirubinemia	chr22:17168884:T:C	0.00000004	rs5748926-T	non-alcoholic fatty liver disease activity score in non-alcoholic fatty liver disease	T	C	-0.036	0.965	7.27E-01	-0.292	0.747	4.2E-02	1.7E-02	1	0.618988	1
Hyperbilirubinemia	chr3:17791337:C:T	0.00000001	rs6577596-C	aspartate aminotransferase levels	C	T	0.012	1.012	9.09E-01	0.433	1.542	5.4E-03	1.8E-02	1	0.968128	1
Hyperbilirubinemia	chr6:32411959:G:A	1E-10	rs9405098-A	hepatitis c induced liver cirrhosis	G	A	0.548	1.729	1.94E-02	0.000	1.000	1.0E+00	1.8E-02	1	0.909637	1
Hyperbilirubinemia	chr10:63361805:C:A	5E-10	rs12355784-A	liver enzyme levels	C	A	0.105	1.111	2.79E-01	0.127	1.135	3.5E-01	2.0E-02	1	0.016485	1
Hyperbilirubinemia	chr10:63374062:A:G	6E-23	rs7923609-G	liver enzyme levels (alkaline phosphatase)	A	G	0.115	1.122	2.36E-01	0.127	1.135	3.5E-01	2.1E-02	1	0.017772	1
Hyperbilirubinemia	chr1:97082391:T:A	0.000000001	p.D949V	CPIC:DPYD	T	A	0.343	1.410	6.23E-01	1.784	5.955	4.4E-03	2.2E-02	1	NA	NA
Hyperbilirubinemia	chr6:13512277:G:A	4E-18	rs2841532-A	aspartate aminotransferase levels	G	A	-0.016	0.985	8.79E-01	0.329	1.389	3.4E-02	2.4E-02	1	0.000475	0.138462
Hyperbilirubinemia	chr15:40038428:C:T	1E-17	rs8041057-C	alanine aminotransferase levels;aspartate aminotransferase levels	C	T	-0.187	0.830	5.83E-02	-0.140	0.869	3.0E-01	2.5E-02	1	0.403614	1
Hyperbilirubinemia	chr7:99652770:T:TA	0.000000001	346Frameshift	CPIC:CYP3A5	T	TA	0.033	1.034	9.63E-01	0.869	2.384	3.9E-02	2.7E-02	1	0.144072	1
Hyperbilirubinemia	chr11:94180526:T:C	6E-18	rs74913549-?	alanine transaminase levels;aspartate aminotransferase levels	T	C	0.067	1.070	6.70E-01	0.611	1.842	2.1E-03	3.0E-02	1	0.14227	1
Hyperbilirubinemia	chr12:121102728:G:A	6E-12	rs1794898-G	alanine aminotransferase levels	G	A	-0.081	0.923	5.60E-01	-0.160	0.852	3.7E-01	3.0E-02	1	0.014967	0.989744
Hyperbilirubinemia	chr18:66962261:A:G	0.000000007	rs116561224-	drug-induced liver injury (statins)	A	G	-0.090	0.914	7.44E-01	-0.872	0.418	3.5E-02	3.2E-02	1	0.365711	1
Hyperbilirubinemia	chr17:31312307:G:C	1E-13	rs4638642-G	aspartate aminotransferase levels	G	C	0.007	1.007	9.42E-01	-0.187	0.830	1.7E-01	3.5E-02	1	0.906473	1

© 2022 Yang W et al. *JAMA Network Open*.

Hyperbilirubinemia	chr10:94981224:C:T	0.000000001	p.R335W	CPIC:CYP2C9	C	T	0.160	1.174	8.19E-01	0.899	2.456	2.2E-01	4.1E-02	1	NA	NA
Hyperbilirubinemia	chr8:71507263:T:G	4E-10	rs10096191-G	aspartate aminotransferase levels;alanine aminotransferase levels	T	G	-0.453	0.636	3.40E-02	-0.627	0.534	6.4E-02	4.5E-02	1	0.248063	1
Hyperbilirubinemia	chr6:18138997:C:T	0.000000001	A154T	CPIC:TPMT	C	T	-0.553	0.575	6.42E-02	-0.533	0.587	2.8E-01	4.5E-02	1	0.3029	1
Hyperbilirubinemia	chr4:99527667:C:T	0.00000002	rs10007975-C	alanine aminotransferase levels	C	T	0.074	1.077	4.85E-01	0.107	1.113	4.7E-01	4.5E-02	1	0.048016	1
Hyperbilirubinemia	chr14:24402720:C:T	6E-13	rs11621792-T	alanine aminotransferase levels	C	T	0.235	1.265	1.91E-02	0.187	1.206	1.9E-01	4.5E-02	1	0.605168	1
Hyperbilirubinemia	chr20:25317451:A:G	7E-10	rs7267979-G	liver enzyme levels (alkaline phosphatase)	A	G	-0.113	0.893	2.39E-01	-0.081	0.922	5.5E-01	4.6E-02	1	0.086268	1
Hyperbilirubinemia	chr14:34715117:T:C	9E-13	rs10141892-C	alanine aminotransferase levels;aspartate aminotransferase levels	T	C	0.227	1.255	1.64E-02	-0.043	0.958	7.5E-01	4.7E-02	1	0.707031	1
Hyperbilirubinemia	chr7:35515178:C:T	0.00000003	rs343064-A	non-alcoholic fatty liver disease histology (other)	C	T	0.152	1.165	1.12E-01	0.165	1.179	2.3E-01	4.8E-02	1	0.154546	1
Hyperbilirubinemia	chr10:63405424:G:A	3E-11	rs10822168-?	total bilirubin levels	G	A	0.082	1.085	4.01E-01	0.098	1.103	4.7E-01	4.9E-02	1	0.044618	1
Hyperbilirubinemia	chr1:155154472:T:C	2E-15	rs10908458-T	liver enzyme levels (gamma-glutamyl transferase)	T	C	-0.317	0.728	1.40E-03	-0.131	0.878	3.3E-01	5.0E-02	1	0.672592	1
Hyperbilirubinemia	chr2:100962027:G:A	0.000000005	rs6747874-A	aspartate aminotransferase levels	G	A	-0.134	0.875	2.15E-01	-0.238	0.788	1.2E-01	5.1E-02	1	0.83313	1

© 2022 Yang W et al. *JAMA Network Open*.

Hyperbilirubinemia	chr22:43928847:C:G	1E-300	rs738409-G	aminotransferase levels;alanine aminotransferase levels;alanine transaminase levels in high alcohol intake;aspartate transaminase levels in high alcohol intake;liver fat content (mri proton density fat fraction measure);aspartate aminotransferase platelet ratio index in high alcohol intake;alanine transaminase levels;nonalcoholic fatty liver disease;liver enzyme levels (alanine transaminase);liver fibrosis and steatohepatitis severity (mri ct1 measure);percent liver fat;alanine aminotransferase (alt)	C	G	0.356	1.428	4.28E-04	0.046	1.048	7.6E-01	5.2E-02	1	0.243795	1

© 2022 Yang W et al. *JAMA Network Open*.

Hyperbilirubinemia	chr22:43928850:C:T	8E-82	rs738408-?	alanine transaminase levels in high alcohol intake;aspartate transaminase levels in high alcohol intake;aspartate aminotransferase levels;aspartate aminotransferase platelet ratio index in high alcohol intake;alanine aminotransferase (alt) levels after remission induction therapy in actute lymphoblastic leukemia (all)	C	T	0.356	1.428	4.28E-04	0.046	1.048	7.6E-01	5.2E-02	1	0.243795	1
Hyperbilirubinemia	chr11:125582185:C:T	0.00000002	rs11220136-T	alanine aminotransferase levels	C	T	0.196	1.217	2.82E-01	0.279	1.322	2.7E-01	5.3E-02	1	0.499101	1
Hyperbilirubinemia	chr22:42128181:A:T	0.000000001	p.M279K	CPIC:CYP2D6	A	T	1.119	3.063	3.95E-01	1.575	4.832	2.9E-01	5.4E-02	1	NA	NA
Hyperbilirubinemia	chr1:198665496:T:C	0.000000003	rs1052238-T	aspartate aminotransferase levels	T	C	0.127	1.135	1.78E-01	-0.025	0.975	8.5E-01	5.6E-02	1	0.883409	1
Hyperbilirubinemia	chr8:144729622:T:C	5E-25	rs2467663-T	alanine aminotransferase levels	T	C	-0.009	0.991	9.31E-01	-0.173	0.841	2.2E-01	5.7E-02	1	0.926647	1
Hyperbilirubinemia	chr19:46718300:C:G	6E-13	rs313839-G	aspartate aminotransferase levels	C	G	0.102	1.107	4.21E-01	0.144	1.154	4.3E-01	5.8E-02	1	0.362142	1
Hyperbilirubinemia	chr9:4774830:G:C	9E-15	rs35954307-?	aspartate aminotransferase platelet ratio index in high alcohol intake	G	C	-0.447	0.640	7.27E-04	-0.008	0.993	9.7E-01	5.8E-02	1	0.312502	1

© 2022 Yang W et al. *JAMA Network Open*.

Hyperbilirubinemia	chr6:152721900:G:A	0.00000001	rs17710008-A	alanine aminotransferase levels	G	A	0.017	1.017	8.85E-01	0.341	1.407	4.4E-02	5.9E-02	1	0.956731	1

Hyperbilirubinemia	chr17:72110287:T:C	1E-12	rs1477066-C	alanine aminotransferase levels	T	C	0.062	1.064	5.16E-01	0.189	1.208	1.7E-01	5.9E-02	1	0.718553	1
Hyperbilirubinemia	chr10:63515167:A:G	7E-10	rs10761779-G	liver enzyme levels	A	G	0.089	1.093	3.61E-01	0.078	1.081	5.6E-01	6.0E-02	1	0.028063	1
Hyperbilirubinemia	chr15:73685996:C:T	0.000000001	rs8038465-T	liver enzyme levels (gamma-glutamyl transferase)	C	T	-0.088	0.916	3.84E-01	-0.225	0.798	1.2E-01	6.0E-02	1	0.045174	1
Hyperbilirubinemia	chr22:43928975:G:A	4E-82	rs3747207-?	alanine transaminase levels in high alcohol intake;aspartate transaminase levels in high alcohol intake;aspartate aminotransferase platelet ratio index in high alcohol intake;alanine aminotransferase levels in excessive alcohol consumption;aspartate aminotransferase levels in excessive alcohol consumption	G	A	0.366	1.442	2.96E-04	0.023	1.023	8.8E-01	6.2E-02	1	0.232617	1

© 2022 Yang W et al. *JAMA Network Open*.

Hyperbilirubinemia	chr14:94378610:C:T	1E-17	rs28929474-?	alanine aminotransferase levels;alanine transaminase levels;alanine transaminase levels in high alcohol intake;aspartate transaminase levels in high alcohol intake	C	T	0.707	2.027	3.64E-02	0.705	2.024	1.7E-01	6.4E-02	1	0.67851	1
Hyperbilirubinemia	chr22:42130715:C:T	0.000000001	p.R26H	CPIC:CYP2D6	C	T	1.180	3.255	1.42E-01	0.471	1.601	6.9E-01	6.5E-02	1	0.389016	1
Hyperbilirubinemia	chr11:94198621:T:C	1E-24	rs1857682-?	alanine aminotransferase levels	T	C	0.211	1.235	4.78E-02	0.222	1.248	1.4E-01	6.9E-02	1	0.757547	1

Hyperbilirubinemia	chr2:88124547:T:C	0.000000004	rs2241883-T	alanine aminotransferase levels	T	C	0.136	1.146	1.68E-01	0.058	1.059	7.0E-01	7.1E-02	1	0.817822	1
Hyperbilirubinemia	chr7:99652613:G:T	0.000000001	T398N	CPIC:CYP3A5	G	T	0.850	2.341	2.59E-01	0.654	1.923	4.6E-01	7.1E-02	1	NA	NA
Hyperbilirubinemia	chr8:18415371:G:A	7E-11	rs1495741-A	liver injury in anti-tuberculosis drug treatment	G	A	-0.167	0.846	9.96E-02	-0.217	0.805	1.4E-01	7.1E-02	1	0.147024	1
Hyperbilirubinemia	chr12:52891302:T:G	2E-10	rs11170319-G	aspartate aminotransferase levels	T	G	0.228	1.256	1.88E-02	-0.118	0.889	3.9E-01	7.3E-02	1	0.399459	1
Hyperbilirubinemia	chr6:32400310:T:C	0.00000001	rs3817963-A	hepatitis c induced liver cirrhosis	T	C	0.075	1.078	4.56E-01	0.158	1.172	2.6E-01	7.3E-02	1	0.819041	1
Hyperbilirubinemia	chr2:187346716:C:T	7E-10	rs10931283-T	aspartate aminotransferase levels	C	T	0.173	1.188	7.82E-02	0.058	1.060	6.7E-01	7.3E-02	1	0.135895	1
Hyperbilirubinemia	chr9:33117967:C:T	6E-14	rs7865362-?	aspartate aminotransferase levels	C	T	0.080	1.084	4.20E-01	0.148	1.160	2.9E-01	7.5E-02	1	2.95E-05	0.051282
Hyperbilirubinemia	chr2:71335373:T:C	2E-10	rs17743415-C	aspartate aminotransferase levels	T	C	0.136	1.145	1.63E-01	-0.139	0.870	3.2E-01	7.5E-02	1	0.375435	1

© 2022 Yang W et al. *JAMA Network Open*.

Hyperbilirubinemia	chr7:116777794:G:C	0.000000001	rs11770163-C	alanine aminotransferase levels	G	C	-0.124	0.883	2.33E-01	-0.037	0.964	7.9E-01	7.8E-02	1	0.136706	1
Hyperbilirubinemia	chr6:32421871:A:G	1E-10	rs3135363-C	hepatitis c induced liver cirrhosis	A	G	-0.074	0.928	4.97E-01	-0.295	0.745	7.7E-02	8.0E-02	1	0.167154	1
Hyperbilirubinemia	chr4:87292656:A:G	5E-69	rs6834314-A	alanine aminotransferase levels;aspartate aminotransferase levels;liver enzyme levels (alanine transaminase)	A	G	-0.266	0.767	2.66E-02	-0.084	0.919	6.0E-01	8.1E-02	1	0.600092	1
Hyperbilirubinemia	chr6:27332531:A:G	8E-30	rs13212562-A	aspartate aminotransferase levels	A	G	0.084	1.088	5.73E-01	0.114	1.121	5.9E-01	8.2E-02	1	0.977994	1
Hyperbilirubinemia	chr15:73692882:T:C	2E-19	rs2127015-C	alanine aminotransferase levels	T	C	-0.054	0.948	5.69E-01	-0.253	0.777	5.5E-02	8.4E-02	1	0.060667	1
Hyperbilirubinemia	chr22:42129180:A:T	0.000000001	p.F120I	CPIC:CYP2D6	A	T	1.796	6.023	9.73E-02	-10.937	0.000	9.7E-01	8.6E-02	1	0.458486	1
Hyperbilirubinemia	chr19:41004377:A:G	0.000000001	K139E	CPIC:CYP2B6	A	G	0.608	1.836	4.56E-01	0.725	2.064	4.2E-01	8.7E-02	1	NA	NA
Hyperbilirubinemia	chr1:155134221:G:A	1E-30	rs12904-G	alanine aminotransferase levels;aspartate aminotransferase levels	G	A	-0.303	0.739	2.15E-03	-0.081	0.923	5.5E-01	8.8E-02	1	0.796776	1
Hyperbilirubinemia	chr6:127113961:A:T	0.00000003	rs6916318-T	aspartate aminotransferase levels	A	T	-0.049	0.952	6.02E-01	0.009	1.009	9.4E-01	8.9E-02	1	0.042635	1
Hyperbilirubinemia	chr1:21439960:A:C	2E-50	rs1976403-C	liver enzyme levels (alkaline phosphatase)	A	C	-0.282	0.754	7.22E-03	-0.250	0.779	8.1E-02	9.0E-02	1	0.389016	1
Hyperbilirubinemia	chr10:77900367:T:C	5E-27	rs10430531-C	alanine aminotransferase levels	T	C	0.182	1.200	9.25E-02	0.051	1.052	7.4E-01	9.1E-02	1	0.377166	1

© 2022 Yang W et al. *JAMA Network Open*.

Hyperbilirubinemia	chr6:135106021:T:C	5E-14	rs9389269-?	aspartate aminotransferase platelet ratio index in high alcohol intake	T	C	0.213	1.237	4.66E-02	-0.076	0.927	6.4E-01	9.1E-02	1	0.299941	1
Hyperbilirubinemia	chr22:36131919:A:G	0.00000001	rs5756080-?	alanine transaminase levels in high alcohol intake	A	G	0.039	1.040	7.15E-01	-0.299	0.741	4.7E-02	9.4E-02	1	0.005051	0.85641
Hyperbilirubinemia	chr11:116778201:G:C	2E-19	rs964184-G	aspartate aminotransferase levels	G	C	-0.012	0.988	9.19E-01	-0.185	0.831	2.6E-01	9.5E-02	1	0.173341	1
Hyperbilirubinemia	chr5:132461855:A:G	1E-12	rs6894249-A	aspartate aminotransferase levels	A	G	0.154	1.166	1.12E-01	0.002	1.002	9.9E-01	9.6E-02	1	0.414281	1
Hyperbilirubinemia	chr6:135098493:A:G	0.000000004	rs9389268-?	aspartate aminotransferase levels	A	G	0.210	1.233	5.49E-02	-0.102	0.903	5.3E-01	9.9E-02	1	0.649636	1
Hyperbilirubinemia	chr12:124824136:G:A	4E-11	rs10846742-?	total bilirubin levels	G	A	0.153	1.166	1.80E-01	0.185	1.203	2.6E-01	1.0E-01	1	0.387874	1

Hyperbilirubinemia	chr8:52506268:T:A	0.00000002	rs7820212-A	immunoglobulin light chain (al) amyloidosis (liver involvement)	T	A	-0.048	0.953	6.20E-01	-0.013	0.987	9.2E-01	1.0E-01	1	0.069481	1
Hyperbilirubinemia	chr17:48065680:T:C	9E-10	rs7209484-C	aspartate aminotransferase levels	T	C	-0.023	0.977	8.26E-01	-0.406	0.666	1.5E-02	1.0E-01	1	0.690505	1
Hyperbilirubinemia	chr17:72102020:G:C	0.000000001	rs9913711-C	liver enzyme levels (gamma-glutamyl transferase)	G	C	-0.033	0.968	7.45E-01	0.263	1.301	7.7E-02	1.0E-01	1	0.611139	1
Hyperbilirubinemia	chr19:49509317:G:A	6E-10	rs34010237-?	aspartate aminotransferase levels	G	A	-0.162	0.850	2.63E-01	-0.141	0.868	4.6E-01	1.0E-01	1	0.349256	1
Hyperbilirubinemia	chr16:58730951:C:A	2E-12	rs73550818-?	aspartate aminotransferase levels	C	A	-0.209	0.812	1.75E-01	0.028	1.029	8.9E-01	1.1E-01	1	0.166063	1
Hyperbilirubinemia	chr17:46006582:T:C	2E-12	rs8067056-T	aspartate aminotransferase levels	T	C	0.048	1.049	6.28E-01	0.026	1.026	8.5E-01	1.1E-01	1	0.527126	1

© 2022 Yang W et al. *JAMA Network Open*.

Hyperbilirubinemia	chr5:73110201:A:G	3E-11	rs554327-?	aspartate aminotransferase levels	A	G	0.125	1.133	1.85E-01	-0.186	0.830	1.7E-01	1.1E-01	1	0.4611	1
Hyperbilirubinemia	chr9:19469848:G:T	0.000000009	rs11790131-?	liver fibrosis severity in hiv/hepatitis c co-infection	G	T	0.016	1.017	8.91E-01	-0.334	0.716	6.7E-02	1.1E-01	1	0.500063	1
Hyperbilirubinemia	chr15:89802996:A:G	2E-10	rs7168849-A	alanine aminotransferase levels	A	G	0.164	1.178	1.81E-01	0.025	1.025	8.8E-01	1.1E-01	1	0.581066	1
Hyperbilirubinemia	chr15:60591082:C:A	7E-20	rs339969-A	liver enzyme levels (gamma-glutamyl transferase);alanine aminotransferase levels;aspartate aminotransferase levels	C	A	0.046	1.047	6.45E-01	-0.160	0.852	2.3E-01	1.1E-01	1	0.245395	1
Hyperbilirubinemia	chr1:109279521:G:A	0.000000003	rs1277930-A	alanine aminotransferase levels	G	A	0.012	1.013	9.12E-01	-0.174	0.840	2.5E-01	1.2E-01	1	0.040492	1
Hyperbilirubinemia	chr19:49495592:G:C	0.00000002	rs34750569-?	aspartate aminotransferase levels	G	C	-0.162	0.851	2.59E-01	-0.204	0.815	2.9E-01	1.2E-01	1	0.361711	1
Hyperbilirubinemia	chr19:15864286:G:T	0.00000003	rs11086005-G	aspartate aminotransferase levels	G	T	0.007	1.007	9.39E-01	-0.292	0.747	3.3E-02	1.2E-01	1	0.431551	1
Hyperbilirubinemia	chr3:37042249:A:G	0.00000002	rs9876116-A	alanine aminotransferase levels	A	G	0.062	1.064	5.31E-01	0.118	1.125	3.8E-01	1.2E-01	1	0.027611	1
Hyperbilirubinemia	chr1:150507425:C:T	1E-12	rs1815544-C	alanine aminotransferase levels	C	T	-0.046	0.955	6.57E-01	0.392	1.480	4.5E-03	1.2E-01	1	0.737449	1
Hyperbilirubinemia	chr6:43789345:T:A	4E-14	rs4711750-A	alanine aminotransferase levels	T	A	0.238	1.269	1.11E-02	0.017	1.017	9.0E-01	1.3E-01	1	0.845233	1

© 2022 Yang W et al. *JAMA Network Open*.

Hyperbilirubinemia	chr6:135097901:C:A	1E-14	rs56293029-?	aspartate aminotransferase platelet ratio index in high alcohol intake	C	A	0.213	1.238	5.04E-02	-0.118	0.889	4.7E-01	1.3E-01	1	0.633048	1
Hyperbilirubinemia	chr4:3442204:A:G	3E-12	rs13108218-A	alanine aminotransferase levels;aspartate aminotransferase levels	A	G	0.061	1.063	5.11E-01	-0.147	0.863	2.5E-01	1.3E-01	1	0.20784	1
Hyperbilirubinemia	chr16:68626662:C:T	7E-10	rs6499186-C	liver fibrosis in non-alcoholic fatty acid liver disease	C	T	0.184	1.202	1.11E-01	0.181	1.199	2.9E-01	1.3E-01	1	0.506433	1
Hyperbilirubinemia	chr12:21205999:G:C	0.000000001	G488A	CPIC:SLCO1B1	G	C	0.527	1.694	5.51E-01	1.053	2.867	1.6E-01	1.3E-01	1	NA	NA
Hyperbilirubinemia	chr11:2921363:G:A	5E-11	rs1661052-?	total bilirubin levels	G	A	-0.111	0.895	4.28E-01	0.130	1.138	5.6E-01	1.3E-01	1	0.02308	1
Hyperbilirubinemia	chr15:84626943:T:C	0.000000001	rs61394864-C	aspartate aminotransferase levels	T	C	-0.094	0.911	3.13E-01	0.273	1.314	5.0E-02	1.3E-01	1	0.008138	0.958974
Hyperbilirubinemia	chr13:27865252:T:C	4E-12	rs1970619-T	alanine aminotransferase levels	T	C	-0.020	0.981	8.36E-01	-0.143	0.867	2.9E-01	1.3E-01	1	0.09779	1
Hyperbilirubinemia	chr6:135097778:A:G	6E-20	rs7776054-A	aspartate aminotransferase levels	A	G	0.212	1.236	5.25E-02	-0.118	0.889	4.7E-01	1.3E-01	1	0.632896	1
Hyperbilirubinemia	chr5:52897294:A:G	3E-13	rs4074793-G	alanine aminotransferase levels;liver enzyme levels (gamma-glutamyl transferase)	A	G	0.073	1.076	6.61E-01	0.567	1.763	1.4E-02	1.4E-01	1	0.261492	1
Hyperbilirubinemia	chr14:103393869:G:A	1E-10	rs12889639-A	aspartate aminotransferase levels	G	A	0.100	1.105	3.22E-01	-0.017	0.983	9.1E-01	1.4E-01	1	0.037006	1
Hyperbilirubinemia	chr10:93050042:A:T	0.000000001	rs835278-?	total bilirubin levels	A	T	0.088	1.092	3.63E-01	0.076	1.079	5.7E-01	1.4E-01	1	0.804757	1
Hyperbilirubinemia	chr19:41012693:T:A	0.000000001	I391N	CPIC:CYP2B6	T	A	0.794	2.212	1.62E-01	0.302	1.352	7.2E-01	1.4E-01	1	NA	NA
Hyperbilirubinemia	chr19:40832467:C:T	8E-14	rs3852868-?	aspartate aminotransferase levels	C	T	0.183	1.200	5.03E-02	-0.080	0.923	5.5E-01	1.5E-01	1	0.131097	1

© 2022 Yang W et al. *JAMA Network Open*.

Hyperbilirubinemia	chr7:56005148:C:T	0.00000003	rs4948100-?	alanine aminotransferase levels	C	T	0.118	1.125	2.62E-01	0.124	1.132	4.0E-01	1.6E-01	1	0.890559	1
Hyperbilirubinemia	chr5:161257724:T:C	0.000000003	rs114811931-	drug-induced liver injury (diclofenac)	T	C	-0.042	0.959	8.59E-01	-0.512	0.599	3.1E-01	1.6E-01	1	0.525377	1
Hyperbilirubinemia	chr19:44908684:T:C	5E-35	rs429358-T	alanine aminotransferase levels;liver fat content (mri proton density fat fraction measure)	T	C	-0.100	0.905	4.77E-01	-0.352	0.703	8.3E-02	1.6E-01	1	0.572293	1
Hyperbilirubinemia	chr2:201196097:C:T	0.00000001	rs3731714-C	alanine aminotransferase levels	C	T	-0.090	0.914	4.18E-01	0.031	1.032	8.4E-01	1.6E-01	1	0.122038	1
Hyperbilirubinemia	chr1:220796686:A:G	1E-31	rs2642438-G	alanine aminotransferase levels;alanine transaminase levels in high alcohol intake;nonalcoholic fatty liver disease	A	G	-0.225	0.799	3.30E-02	0.105	1.111	5.0E-01	1.6E-01	1	0.416058	1
Hyperbilirubinemia	chr1:109928142:G:A	2E-64	rs333947-G	aspartate aminotransferase levels	G	A	-0.071	0.932	5.55E-01	0.517	1.677	2.0E-03	1.6E-01	1	0.401833	1
Hyperbilirubinemia	chr22:42128308:C:A	0.000000001	p.A237S	CPIC:CYP2D6	C	A	-15.059	0.000	9.76E-01	-0.877	0.416	4.1E-01	1.6E-01	1	0.900544	1
Hyperbilirubinemia	chr6:24441518:G:A	1E-11	rs9467160-A	liver enzyme levels	G	A	-0.061	0.941	5.78E-01	0.048	1.049	7.5E-01	1.6E-01	1	0.043219	1
Hyperbilirubinemia	chr10:98480617:C:T	3E-10	rs72838936-?	aspartate aminotransferase levels	C	T	0.188	1.207	1.64E-01	-0.188	0.829	3.9E-01	1.6E-01	1	0.015497	0.989744
Hyperbilirubinemia	chr5:157326276:T:C	5E-15	rs2289850-T	alanine aminotransferase levels	T	C	-0.083	0.920	6.79E-01	-0.309	0.735	2.1E-01	1.6E-01	1	0.003449	0.717949
Hyperbilirubinemia	chr18:72540105:A:G	0.000000003	rs658995-A	serum bilirubin levels in metabolic syndrome	A	G	0.442	1.556	5.19E-02	-0.092	0.912	7.1E-01	1.6E-01	1	0.00432	0.8

© 2022 Yang W et al. *JAMA Network Open*.

Hyperbilirubinemia	chr7:56022948:T:A	3E-12	rs6973917-T	alanine aminotransferase levels	T	A	0.120	1.127	2.74E-01	0.171	1.186	3.0E-01	1.6E-01	1	0.386566	1
Hyperbilirubinemia	chr10:45443298:C:T	0.00000001	rs1051713-C	alanine aminotransferase levels	C	T	0.141	1.152	2.48E-01	-0.524	0.592	1.2E-02	1.6E-01	1	0.363551	1
Hyperbilirubinemia	chr1:97450068:A:G	0.000000001	p.F632F	CPIC:DPYD	A	G	0.117	1.124	5.31E-01	0.205	1.227	4.5E-01	1.7E-01	1	0.863208	1
Hyperbilirubinemia	chr6:43790159:C:A	1E-16	rs998584-A	aspartate aminotransferase levels	C	A	0.207	1.229	2.74E-02	0.015	1.015	9.1E-01	1.7E-01	1	0.928543	1
Hyperbilirubinemia	chr10:99397681:G:C	9E-10	rs76850691-C	aspartate aminotransferase levels;liver enzyme levels (aspartate transaminase)	G	C	-14.383	0.000	9.66E-01	-13.189	0.000	9.7E-01	1.7E-01	1	1	1
Hyperbilirubinemia	chr2:164700808:C:T	3E-17	rs6712203-C	alanine aminotransferase levels	C	T	-0.170	0.844	1.10E-01	0.109	1.115	4.4E-01	1.7E-01	1	0.278416	1
Hyperbilirubinemia	chr1:16178825:A:G	1E-54	rs1497406-G	alanine aminotransferase levels;liver enzyme levels (gamma-glutamyl transferase)	A	G	0.021	1.021	8.22E-01	-0.015	0.985	9.1E-01	1.8E-01	1	0.927584	1
Hyperbilirubinemia	chr7:106687688:G:T	0.00000003	rs342221-?	aspartate aminotransferase platelet ratio index in high alcohol intake	G	T	0.052	1.053	5.87E-01	0.085	1.088	5.4E-01	1.8E-01	1	0.899472	1
Hyperbilirubinemia	chr3:149494481:G:A	3E-15	rs62269283-?	alanine aminotransferase levels;alanine transaminase levels	G	A	0.142	1.153	7.87E-01	0.405	1.500	5.2E-01	1.8E-01	1	0.369208	1
Hyperbilirubinemia	chr20:14691531:T:C	0.000000005	rs6034011-C	alanine aminotransferase levels	T	C	-0.082	0.921	3.99E-01	0.394	1.483	5.0E-03	1.8E-01	1	0.340936	1

© 2022 Yang W et al. *JAMA Network Open*.

Hyperbilirubinemia	chr11:95450772:T:C	0.00000001	rs12799680-T	aspartate aminotransferase levels	T	C	0.103	1.108	5.11E-01	0.228	1.256	2.7E-01	1.8E-01	1	0.211656	1
Hyperbilirubinemia	chr11:22250324:A:T	8E-26	rs7481951-T	aspartate aminotransferase levels;alanine aminotransferase levels	A	T	-0.003	0.997	9.75E-01	-0.364	0.695	1.1E-02	1.8E-01	1	0.196063	1
Hyperbilirubinemia	chr2:11641492:T:C	0.00000002	rs149940960-	alanine aminotransferase (alt) levels after remission induction therapy in actute lymphoblastic leukemia (all)	T	C	0.722	2.058	3.16E-01	-12.584	0.000	9.8E-01	1.9E-01	1	NA	NA
Hyperbilirubinemia	chr2:226248038:C:T	2E-20	rs2943654-T	alanine aminotransferase levels;aspartate aminotransferase levels	C	T	0.143	1.154	1.92E-01	0.231	1.260	1.2E-01	1.9E-01	1	0.540158	1
Hyperbilirubinemia	chr1:97573881:C:T	0.000000001	p.M406I	CPIC:DPYD	C	T	0.699	2.011	2.32E-01	0.233	1.263	7.4E-01	1.9E-01	1	0.046157	1
Hyperbilirubinemia	chr8:10736964:C:A	2E-20	rs4841436-C	aspartate aminotransferase levels	C	A	-0.184	0.832	5.43E-02	0.079	1.082	5.7E-01	1.9E-01	1	0.759649	1
Hyperbilirubinemia	chr11:1758975:A:G	9E-13	rs11555039-G	aspartate aminotransferase levels	A	G	-0.248	0.780	2.00E-01	-0.268	0.765	3.0E-01	1.9E-01	1	0.669149	1
Hyperbilirubinemia	chr11:126355981:C:G	2E-14	rs112771035-	aspartate aminotransferase levels	C	G	0.304	1.356	6.74E-02	0.155	1.168	5.2E-01	2.0E-01	1	0.426011	1
Hyperbilirubinemia	chr12:12362395:A:G	0.00000002	rs11054848-G	aspartate aminotransferase levels	A	G	0.018	1.018	8.52E-01	0.204	1.226	1.5E-01	2.0E-01	1	0.400808	1
Hyperbilirubinemia	chr19:41012316:T:C	0.000000001	I328T	CPIC:CYP2B6	T	C	0.240	1.272	7.17E-01	0.566	1.761	3.4E-01	2.0E-01	1	0.037472	1

© 2022 Yang W et al. *JAMA Network Open*.

Hyperbilirubinemia	chr8:9327636:T:C	5E-34	rs2126259-T	alanine aminotransferase levels	T	C	0.114	1.121	4.04E-01	-0.283	0.753	1.3E-01	2.0E-01	1	0.716317	1
Hyperbilirubinemia	chr10:77814320:G:A	1E-16	rs11002301-A	aspartate aminotransferase levels	G	A	0.207	1.230	3.63E-02	-0.053	0.948	7.1E-01	2.0E-01	1	0.466287	1
Hyperbilirubinemia	chr17:37713312:C:A	2E-15	rs17138478-C	alanine aminotransferase levels;aspartate aminotransferase levels	C	A	0.143	1.154	3.06E-01	0.093	1.098	6.3E-01	2.0E-01	1	0.510079	1
Hyperbilirubinemia	chr1:88681169:A:G	1E-20	rs1002436-G	aspartate aminotransferase levels;alanine aminotransferase levels	A	G	-0.076	0.927	4.30E-01	-0.064	0.938	6.3E-01	2.0E-01	1	0.520103	1
Hyperbilirubinemia	chr1:150770224:T:C	2E-18	rs11204725-C	aspartate aminotransferase levels	T	C	0.106	1.111	2.55E-01	0.188	1.207	1.7E-01	2.0E-01	1	0.550939	1
Hyperbilirubinemia	chr6:69829361:G:A	0.000000003	rs77249491-?	percent liver fat;nonalcoholic fatty liver disease	G	A	0.400	1.491	6.45E-01	1.404	4.073	3.0E-02	2.0E-01	1	NA	NA
Hyperbilirubinemia	chr11:72786417:C:T	5E-12	rs481206-T	alanine aminotransferase levels	C	T	0.088	1.092	3.75E-01	-0.221	0.802	1.5E-01	2.1E-01	1	0.793286	1
Hyperbilirubinemia	chr4:87255231:T:C	4E-15	rs28664118-?	aspartate aminotransferase levels	T	C	-0.178	0.837	1.50E-01	-0.179	0.836	3.0E-01	2.1E-01	1	0.53687	1
Hyperbilirubinemia	chr1:21495264:A:G	7E-15	rs1780324-T	liver enzyme levels	A	G	0.187	1.205	5.19E-02	0.164	1.179	2.2E-01	2.1E-01	1	0.750208	1
Hyperbilirubinemia	chr13:48045719:C:T	0.000000001	R139C	CPIC:NUDT15	C	T	-0.269	0.764	4.69E-01	-1.231	0.292	1.2E-01	2.1E-01	1	0.4942	1
Hyperbilirubinemia	chr9:91218276:T:A	2E-12	rs56049491-?	total bilirubin levels	T	A	-0.245	0.782	5.90E-02	0.097	1.102	5.9E-01	2.1E-01	1	0.800473	1
Hyperbilirubinemia	chr9:33109998:G:T	8E-33	rs62544382-T	aspartate aminotransferase levels	G	T	-0.049	0.952	6.13E-01	-0.101	0.904	4.6E-01	2.1E-01	1	0.001242	0.338462

© 2022 Yang W et al. *JAMA Network Open*.

Hyperbilirubinemia	chr2:178480456:C:T	6E-16	rs71423566-T	aspartate aminotransferase levels;alanine aminotransferase levels	C	T	-0.217	0.805	7.15E-02	0.003	1.003	9.8E-01	2.2E-01	1	0.725156	1
Hyperbilirubinemia	chr8:125495147:C:A	4E-20	rs2954038-?	alanine transaminase levels in high alcohol intake	C	A	0.052	1.054	6.10E-01	-0.124	0.884	4.2E-01	2.2E-01	1	0.493305	1
Hyperbilirubinemia	chr14:24103723:G:A	3E-11	rs111723834-	liver fibrosis and steatohepatitis severity (mri ct1 measure)	G	A	-0.300	0.741	5.43E-01	-13.851	0.000	9.7E-01	2.2E-01	1	0.864042	1
Hyperbilirubinemia	chr22:50656498:C:T	2E-14	rs9616810-T	aspartate aminotransferase levels	C	T	0.164	1.179	1.32E-01	-0.087	0.917	6.0E-01	2.2E-01	1	0.6884	1
Hyperbilirubinemia	chr3:141744456:A:C	0.000000009	rs16851720-C	hepatitis c induced liver fibrosis	A	C	0.074	1.077	5.50E-01	-0.510	0.600	8.2E-03	2.2E-01	1	0.248425	1
Hyperbilirubinemia	chr12:21239042:A:C	0.000000001	L643F	CPIC:SLCO1B1	A	C	0.364	1.438	7.29E-02	0.290	1.336	3.2E-01	2.2E-01	1	0.153847	1
Hyperbilirubinemia	chr16:68526282:A:G	3E-11	rs698718-A	liver fibrosis in non-alcoholic fatty acid liver disease	A	G	0.106	1.112	3.27E-01	0.221	1.248	1.5E-01	2.3E-01	1	0.943691	1
Hyperbilirubinemia	chr7:80545045:T:C	3E-14	rs139761834-	aspartate aminotransferase levels	T	C	2.075	7.966	4.69E-02	-11.757	0.000	9.7E-01	2.3E-01	1	NA	NA
Hyperbilirubinemia	chr22:42129174:C:A	0.000000001	p.A122S	CPIC:CYP2D6	C	A	0.786	2.195	5.35E-01	-11.074	0.000	9.8E-01	2.3E-01	1	NA	NA
Hyperbilirubinemia	chr2:233385215:C:T	0.00000003	rs4663580-T	bilirubin levels	C	T	-0.036	0.964	8.65E-01	0.237	1.267	5.7E-01	2.3E-01	1	0.187417	1
Hyperbilirubinemia	chr2:233570731:G:A	2E-105	rs12993249-G	bilirubin levels	G	A	0.029	1.030	7.54E-01	0.264	1.302	5.2E-02	2.3E-01	1	0.109878	1
Hyperbilirubinemia	chr12:21178957:A:G	0.000000001	I222V	CPIC:SLCO1B1	A	G	-11.220	0.000	9.82E-01	1.090	2.975	2.4E-01	2.3E-01	1	NA	NA
Hyperbilirubinemia	chr1:88680551:C:A	4E-11	rs12145922-A	liver enzyme levels (gamma-glutamyl transferase)	C	A	-0.063	0.939	5.08E-01	-0.067	0.935	6.2E-01	2.3E-01	1	0.467224	1
Hyperbilirubinemia	chr8:143560999:C:T	3E-10	rs1545536-T	alanine aminotransferase levels	C	T	-0.134	0.874	2.27E-01	0.081	1.085	5.9E-01	2.4E-01	1	0.54628	1
Hyperbilirubinemia	chr12:52901297:T:G	0.00000001	rs4531558-G	alanine aminotransferase levels	T	G	0.216	1.241	2.42E-02	-0.170	0.844	2.1E-01	2.4E-01	1	0.371157	1

© 2022 Yang W et al. *JAMA Network Open*.

Hyperbilirubinemia	chr22:29381515:C:T	5E-15	rs11704551-C	aspartate aminotransferase levels	C	T	0.037	1.038	7.54E-01	0.187	1.205	2.3E-01	2.4E-01	1	0.20362	1
Hyperbilirubinemia	chr2:233732958:C:G	0	rs75520741-?	total bilirubin levels	C	G	0.091	1.095	6.69E-01	0.162	1.176	5.6E-01	2.4E-01	1	0.034578	1
Hyperbilirubinemia	chr5:65395057:T:C	0.000000005	rs76622665-?	aspartate aminotransferase levels in low alcohol consumption	T	C	-0.267	0.765	5.57E-01	0.632	1.882	1.6E-01	2.4E-01	1	0.343054	1
Hyperbilirubinemia	chr15:78944951:C:T	4E-11	rs2289702-T	aspartate aminotransferase levels	C	T	-0.168	0.845	3.81E-01	0.113	1.119	6.3E-01	2.4E-01	1	0.550891	1
Hyperbilirubinemia	chr6:135097880:T:C	0.000000001	rs9399137-?	aspartate aminotransferase levels	T	C	0.225	1.252	4.31E-02	-0.192	0.825	2.6E-01	2.4E-01	1	0.721785	1
Hyperbilirubinemia	chr11:65408899:C:T	3E-13	rs12799896-C	alanine aminotransferase levels	C	T	0.004	1.004	9.71E-01	-0.212	0.809	2.5E-01	2.4E-01	1	0.001658	0.45641
Hyperbilirubinemia	chr10:77920676:A:T	6E-10	rs754466-T	liver enzyme levels (gamma-glutamyl transferase)	A	T	0.133	1.142	2.23E-01	0.019	1.019	9.0E-01	2.4E-01	1	0.731801	1
Hyperbilirubinemia	chr4:87255885:C:A	3E-17	rs11935592-?	alanine aminotransferase levels	C	A	-0.177	0.838	1.53E-01	-0.156	0.855	3.6E-01	2.5E-01	1	0.511334	1
Hyperbilirubinemia	chr10:77920756:C:A	0.000000002	rs754465-?	alanine aminotransferase levels;alanine transaminase levels	C	A	0.130	1.139	1.82E-01	0.004	1.004	9.8E-01	2.5E-01	1	0.052245	1
Hyperbilirubinemia	chr11:122648314:T:G	1E-11	rs6589941-G	aspartate aminotransferase levels	T	G	-0.018	0.982	8.51E-01	-0.217	0.805	1.2E-01	2.6E-01	1	0.173024	1
Hyperbilirubinemia	chr12:112379979:T:A	2E-11	rs11066280-?	aspartate aminotransferase levels;alanine aminotransferase levels	T	A	0.101	1.107	8.75E-01	-1.400	0.247	1.0E-01	2.6E-01	1	0.409551	1

© 2022 Yang W et al. *JAMA Network Open*.

Hyperbilirubinemia	chr17:80390047:G:A	5E-10	rs3185057-G	aspartate aminotransferase levels	G	A	0.053	1.054	7.66E-01	0.069	1.072	7.8E-01	2.6E-01	1	0.041162	1
Hyperbilirubinemia	chr7:50218883:A:T	0.00000003	rs4598207-?	aspartate aminotransferase levels	A	T	-0.052	0.949	5.98E-01	-0.081	0.922	5.8E-01	2.6E-01	1	0.691689	1
Hyperbilirubinemia	chr14:94318281:C:T	5E-12	rs61980636-T	alanine aminotransferase levels	C	T	-0.006	0.994	9.55E-01	0.008	1.008	9.6E-01	2.6E-01	1	0.707573	1
Hyperbilirubinemia	chr10:17219763:G:A	3E-21	rs12413013-G	aspartate aminotransferase levels	G	A	-0.007	0.993	9.44E-01	0.035	1.036	8.1E-01	2.6E-01	1	0.371909	1
Hyperbilirubinemia	chr6:136024992:C:T	0.00000003	rs17835714-?	aspartate aminotransferase levels	C	T	-0.756	0.470	2.44E-02	0.253	1.288	4.2E-01	2.6E-01	1	0.823748	1
Hyperbilirubinemia	chr2:168984064:T:G	0.000000002	rs16856332-T	liver enzyme levels (alkaline phosphatase)	T	G	0.069	1.071	7.80E-01	-0.400	0.670	3.4E-01	2.6E-01	1	0.737048	1
Hyperbilirubinemia	chr6:29588001:A:G	2E-10	rs2235698-G	alanine aminotransferase levels	A	G	-0.187	0.829	9.46E-02	0.050	1.051	7.5E-01	2.7E-01	1	0.553603	1
Hyperbilirubinemia	chr2:232648465:C:A	0.000000001	rs2140773-A	liver enzyme levels (gamma-glutamyl transferase)	C	A	-0.009	0.991	9.25E-01	-0.244	0.783	6.2E-02	2.7E-01	1	0.054911	1
Hyperbilirubinemia	chr8:6719619:G:T	0.000000008	rs2911980-T	alanine aminotransferase levels	G	T	-0.009	0.991	9.36E-01	-0.319	0.727	2.8E-02	2.7E-01	1	0.389141	1
Hyperbilirubinemia	chr3:58437771:A:C	7E-10	rs6794695-?	aspartate transaminase levels in high alcohol intake;aspartate aminotransferase platelet ratio index in high alcohol intake	A	C	-0.219	0.803	1.01E-01	-0.060	0.942	7.5E-01	2.7E-01	1	0.839374	1
Hyperbilirubinemia	chr10:94775507:G:A	0.000000001	R150H	CPIC:CYP2C19	G	A	-13.946	0.000	9.77E-01	-13.150	0.000	9.7E-01	2.7E-01	1	NA	NA

© 2022 Yang W et al. *JAMA Network Open*.

Hyperbilirubinemia	chr13:21776998:T:C	6E-11	rs500802-T	aspartate aminotransferase levels	T	C	-0.004	0.996	9.70E-01	0.137	1.146	3.4E-01	2.7E-01	1	0.508847	1
Hyperbilirubinemia	chr6:32338202:G:T	7E-25	rs3129900-?	lumiracoxib-related liver injury	G	T	-0.047	0.954	7.20E-01	0.198	1.219	3.2E-01	2.7E-01	1	0.32122	1

Hyperbilirubinemia	chr4:183283075:T:C	0.00000001	rs3814420-T	aspartate aminotransferase levels	T	C	0.336	1.400	9.08E-02	0.310	1.363	2.3E-01	2.7E-01	1	0.196118	1
Hyperbilirubinemia	chr10:94949217:A:G	0.000000001	p.H251R	CPIC:CYP2C9	A	G	-0.353	0.702	6.62E-01	-1.349	0.260	2.0E-01	2.8E-01	1	0.497799	1
Hyperbilirubinemia	chr17:17280660:A:C	0.00000002	rs117743490-	alanine aminotransferase levels	A	C	-0.274	0.761	3.14E-01	0.048	1.049	8.8E-01	2.8E-01	1	0.572248	1
Hyperbilirubinemia	chr22:42129075:C:T	0.000000001	p.E155K	CPIC:CYP2D6	C	T	-0.057	0.944	9.60E-01	1.299	3.664	4.1E-02	2.8E-01	1	NA	NA
Hyperbilirubinemia	chr6:32433302:A:G	0.000000001	rs3129860-A	hepatitis c induced liver cirrhosis	A	G	-0.106	0.900	4.70E-01	0.099	1.104	6.6E-01	2.9E-01	1	0.197637	1
Hyperbilirubinemia	chr6:18133847:C:A	0.000000001	Q179H	CPIC:TPMT	C	A	1.800	6.048	4.93E-02	-12.792	0.000	9.7E-01	2.9E-01	1	0.735841	1
Hyperbilirubinemia	chr12:111803962:G:A	5E-13	rs671-A	aspartate aminotransferase levels	G	A	0.153	1.165	8.15E-01	-1.281	0.278	1.3E-01	2.9E-01	1	0.410563	1
Hyperbilirubinemia	chr1:219494910:C:A	1E-11	rs1538742-A	alanine aminotransferase levels	C	A	-0.109	0.897	2.66E-01	0.145	1.157	3.0E-01	2.9E-01	1	0.080835	1
Hyperbilirubinemia	chr2:160010548:G:A	2E-13	rs2667011-?	bilirubin levels	G	A	0.170	1.186	1.87E-01	-0.282	0.754	8.4E-02	2.9E-01	1	0.608729	1
Hyperbilirubinemia	chr12:112136812:C:T	3E-13	rs12231737-T	aspartate aminotransferase levels;alanine aminotransferase levels	C	T	0.128	1.137	8.43E-01	-1.281	0.278	1.3E-01	2.9E-01	1	0.401585	1
Hyperbilirubinemia	chr22:23924539:T:C	0.000000002	rs5751775-T	alanine aminotransferase levels	T	C	0.200	1.222	3.94E-02	0.047	1.048	7.4E-01	3.0E-01	1	0.304601	1
Hyperbilirubinemia	chr8:23016020:G:A	3E-12	rs876435-G	aspartate aminotransferase levels	G	A	-0.038	0.963	6.93E-01	-0.108	0.898	4.4E-01	3.0E-01	1	0.648994	1

© 2022 Yang W et al. *JAMA Network Open*.

Hyperbilirubinemia	chr6:26116754:C:T	0.00000002	rs12206204-T	protein quantitative trait loci (liver);bilirubin levels	C	T	-0.218	0.804	6.10E-01	-0.381	0.683	5.5E-01	3.0E-01	1	NA	NA
Hyperbilirubinemia	chr22:24594246:A:G	4E-11	rs4820599-G	liver enzyme levels	A	G	0.135	1.145	1.79E-01	-0.204	0.815	1.5E-01	3.0E-01	1	0.392353	1
Hyperbilirubinemia	chr4:150759175:G:A	0.000000005	rs28521457-?	drug-induced hepatocellular liver injury	G	A	0.225	1.252	1.96E-01	0.085	1.089	7.2E-01	3.0E-01	1	0.469652	1

Hyperbilirubinemia	chr11:61806212:T:C	0.000000006	rs174551-?	alanine transaminase levels	T	C	0.066	1.068	5.05E-01	0.007	1.007	9.6E-01	3.0E-01	1	0.128941	1
Hyperbilirubinemia	chr7:117509089:C:T	0.000000001	R74W	CPIC:CFTR	C	T	1.089	2.971	4.36E-01	-11.361	0.000	9.7E-01	3.1E-01	1	NA	NA
Hyperbilirubinemia	chr7:117642528:G:A	0.000000001	D1270N	CPIC:CFTR	G	A	0.812	2.252	5.43E-01	-11.361	0.000	9.7E-01	3.1E-01	1	NA	NA
Hyperbilirubinemia	chr12:11638751:A:G	8E-13	rs12824533-G	alanine aminotransferase levels	A	G	0.082	1.085	4.09E-01	0.128	1.137	3.6E-01	3.1E-01	1	0.18532	1
Hyperbilirubinemia	chr19:41012465:C:T	0.000000001	R378X	CPIC:CYP2B6	C	T	1.416	4.120	4.20E-01	-10.635	0.000	9.8E-01	3.1E-01	1	NA	NA
Hyperbilirubinemia	chr12:21178691:C:T	0.000000001	F199=	CPIC:SLCO1B1	C	T	0.283	1.327	2.98E-03	0.140	1.151	3.0E-01	3.2E-01	1	0.165404	1
Hyperbilirubinemia	chr4:102268971:A:G	2E-12	rs151401-G	aspartate aminotransferase levels	A	G	-0.193	0.824	1.17E-01	0.190	1.209	2.4E-01	3.2E-01	1	0.288676	1
Hyperbilirubinemia	chr7:99672916:T:C	0.000000001	Splicing defect	CPIC:CYP3A5	T	C	-0.005	0.995	9.73E-01	-0.224	0.799	2.2E-01	3.2E-01	1	0.679244	1
Hyperbilirubinemia	chr12:21178615:T:C	0.000000001	V174A	CPIC:SLCO1B1	T	C	0.259	1.295	4.57E-02	0.159	1.172	4.0E-01	3.2E-01	1	0.825234	1
Hyperbilirubinemia	chr12:21178615:T:C	7E-13	rs4149056-C	bilirubin levels	T	C	0.259	1.295	4.57E-02	0.159	1.172	4.0E-01	3.2E-01	1	0.825234	1
Hyperbilirubinemia	chr3:8764738:G:A	0.000000001	rs73132848-?	liver fibrosis severity in hiv/hepatitis c co-infection	G	A	0.044	1.045	8.38E-01	0.403	1.497	1.2E-01	3.2E-01	1	0.025072	1
Hyperbilirubinemia	chr18:63178651:T:C	5E-13	rs12454712-T	alanine aminotransferase levels	T	C	0.079	1.082	4.18E-01	-0.035	0.966	8.0E-01	3.2E-01	1	0.594596	1
Hyperbilirubinemia	chr15:73689995:C:T	2E-16	rs8041181-T	aspartate aminotransferase levels	C	T	-0.001	0.999	9.90E-01	-0.214	0.808	1.2E-01	3.2E-01	1	0.02622	1
Hyperbilirubinemia	chr19:54255708:C:T	3E-11	rs380731-T	aspartate aminotransferase levels	C	T	0.098	1.103	3.80E-01	0.123	1.131	4.2E-01	3.3E-01	1	0.164432	1
Hyperbilirubinemia	chr4:154468095:T:C	6E-11	rs17031671-?	bilirubin levels	T	C	0.292	1.339	6.64E-01	0.321	1.379	6.3E-01	3.3E-01	1	0.198908	1

© 2022 Yang W et al. *JAMA Network Open*.

Hyperbilirubinemia	chr9:114386047:T:C	1E-16	rs10739409-?	aspartate aminotransferase levels	T	C	-0.068	0.934	5.14E-01	0.077	1.081	6.2E-01	3.3E-01	1	0.656312	1
Hyperbilirubinemia	chr4:99144358:T:G	2E-28	rs1800759-T	protein quantitative trait loci (liver);aspartate aminotransferase levels	T	G	0.001	1.001	9.90E-01	-0.149	0.861	2.8E-01	3.3E-01	1	NA	NA
Hyperbilirubinemia	chr3:11354347:C:G	0.00000002	rs2594973-C	aspartate aminotransferase levels	C	G	0.020	1.020	8.36E-01	0.011	1.011	9.4E-01	3.3E-01	1	0.799258	1
Hyperbilirubinemia	chr12:20871928:T:G	8E-106	rs73233620-?	total bilirubin levels;bilirubin levels	T	G	-0.211	0.810	3.97E-01	0.096	1.100	7.4E-01	3.3E-01	1	0.446958	1
Hyperbilirubinemia	chr19:5831829:C:T	8E-24	rs17855739-C	aspartate aminotransferase levels	C	T	0.233	1.262	2.12E-01	0.210	1.234	3.9E-01	3.4E-01	1	0.759506	1
Hyperbilirubinemia	chr10:99052093:C:G	1E-12	rs7097660-G	aspartate aminotransferase levels	C	G	-0.056	0.945	5.85E-01	0.239	1.270	1.4E-01	3.4E-01	1	0.301577	1
Hyperbilirubinemia	chr19:48703346:C:T	1E-15	rs281377-T	liver enzyme levels (alkaline phosphatase)	C	T	0.070	1.072	4.64E-01	0.052	1.054	6.9E-01	3.4E-01	1	0.78145	1
Hyperbilirubinemia	chr3:12355089:G:A	6E-22	rs4135247-G	alanine aminotransferase levels;aspartate aminotransferase levels	G	A	-0.130	0.878	1.65E-01	-0.151	0.860	2.5E-01	3.4E-01	1	0.208242	1
Hyperbilirubinemia	chr1:89517625:G:A	0.00000002	rs359935-A	serum bilirubin levels in metabolic syndrome	G	A	-0.045	0.956	9.25E-01	0.481	1.618	3.6E-01	3.4E-01	1	0.139967	1
Hyperbilirubinemia	chr7:87450286:G:C	1E-19	rs4148821-?	alanine aminotransferase levels	G	C	-0.172	0.842	1.62E-01	-0.052	0.949	7.5E-01	3.4E-01	1	0.891456	1
Hyperbilirubinemia	chr10:94988925:A:G	0.000000001	p.N457S	CPIC:CYP2C9	A	G	-11.669	0.000	9.75E-01	2.153	8.607	1.9E-01	3.4E-01	1	NA	NA

© 2022 Yang W et al. *JAMA Network Open*.

Hyperbilirubinemia	chr3:125208361:G:A	0.000000005	rs6762589-A	alanine aminotransferase levels	G	A	0.148	1.159	1.19E-01	-0.075	0.928	5.7E-01	3.5E-01	1	0.808109	1
Hyperbilirubinemia	chr6:29860883:A:G	2E-10	rs2523822-?	drug-induced liver injury (amoxicillin-clavulanate)	A	G	-0.110	0.896	2.92E-01	0.260	1.297	6.4E-02	3.5E-01	1	0.528622	1
Hyperbilirubinemia	chr4:87254878:C:A	1E-16	rs10001545-?	alanine transaminase levels;aspartate aminotransferase levels	C	A	-0.188	0.828	1.40E-01	-0.124	0.884	4.9E-01	3.5E-01	1	0.534025	1
Hyperbilirubinemia	chr21:44789511:C:G	3E-11	rs11088963-C	alanine aminotransferase levels	C	G	-0.100	0.905	3.14E-01	-0.065	0.937	6.4E-01	3.5E-01	1	0.149575	1
Hyperbilirubinemia	chr12:50827344:A:G	1E-10	rs864899-G	alanine aminotransferase levels	A	G	0.078	1.081	4.10E-01	0.039	1.040	7.7E-01	3.5E-01	1	0.974069	1
Hyperbilirubinemia	chr22:42129132:C:T	0.000000001	p.V136M	CPIC:CYP2D6	C	T	-0.007	0.993	9.92E-01	-0.653	0.521	4.0E-01	3.5E-01	1	0.296662	1
Hyperbilirubinemia	chr17:70379805:T:C	4E-10	rs4485410-T	aspartate aminotransferase levels	T	C	0.089	1.093	4.14E-01	0.303	1.354	5.0E-02	3.5E-01	1	0.489962	1
Hyperbilirubinemia	chr10:98367794:G:A	3E-14	rs17109512-?	aspartate aminotransferase levels	G	A	-0.002	0.998	9.91E-01	-0.225	0.799	3.7E-01	3.6E-01	1	0.149079	1
Hyperbilirubinemia	chr11:61930432:G:A	0.000000002	rs6591673-G	aspartate aminotransferase levels	G	A	-0.089	0.914	3.49E-01	-0.036	0.965	7.9E-01	3.6E-01	1	0.536096	1
Hyperbilirubinemia	chr5:75054108:G:A	2E-15	rs76722284-A	aspartate aminotransferase levels	G	A	0.119	1.127	4.02E-01	-0.148	0.862	5.4E-01	3.6E-01	1	0.141357	1
Hyperbilirubinemia	chrX:154536002:C:T	0.000000001	p.V68M	CPIC:G6PD	C	T	-0.496	0.609	4.43E-01	0.786	2.194	5.8E-03	3.6E-01	1	0.169677	1
Hyperbilirubinemia	chr19:18111154:A:C	9E-23	rs273507-C	alanine aminotransferase levels	A	C	0.049	1.050	6.56E-01	-0.166	0.847	2.8E-01	3.6E-01	1	0.39092	1
Hyperbilirubinemia	chr13:48045720:G:A	0.000000001	R139H	CPIC:NUDT15	G	A	0.136	1.145	8.74E-01	1.145	3.143	2.5E-01	3.6E-01	1	NA	NA

© 2022 Yang W et al. *JAMA Network Open*.

Hyperbilirubinemia	chr2:232655544:A:T	4E-50	rs13395911-T	alanine aminotransferase levels;aspartate aminotransferase levels;alanine transaminase levels;alanine transaminase levels in high alcohol intake	A	T	-0.019	0.981	8.44E-01	-0.191	0.826	1.5E-01	3.6E-01	1	0.066218	1
Hyperbilirubinemia	chr1:111194561:C:T	0.00000002	rs660173-C	aspartate aminotransferase levels	C	T	-0.130	0.878	2.24E-01	0.035	1.036	8.2E-01	3.6E-01	1	0.621617	1
Hyperbilirubinemia	chr5:39555201:G:T	0.000000003	rs2939328-?	aspartate aminotransferase levels	G	T	0.032	1.032	7.42E-01	0.090	1.094	5.0E-01	3.6E-01	1	0.955981	1
Hyperbilirubinemia	chr2:27518370:T:C	0.00000002	rs780094-T	nonalcoholic fatty liver disease	T	C	-0.074	0.929	4.43E-01	0.178	1.194	2.0E-01	3.6E-01	1	0.606367	1
Hyperbilirubinemia	chr9:130276442:A:G	0.00000003	rs9657643-A	aspartate aminotransferase levels	A	G	-0.082	0.921	4.60E-01	-0.005	0.995	9.7E-01	3.7E-01	1	0.825808	1
Hyperbilirubinemia	chr22:17957192:C:T	1E-16	rs1076540-C	liver enzyme levels (gamma-glutamyl transferase)	C	T	-0.093	0.911	4.43E-01	0.120	1.127	4.6E-01	3.7E-01	1	0.305138	1
Hyperbilirubinemia	chr22:41830391:C:T	0.00000001	rs5758487-C	alanine aminotransferase levels	C	T	0.091	1.095	3.62E-01	-0.190	0.827	1.5E-01	3.7E-01	1	0.027286	1
Hyperbilirubinemia	chr1:247559286:G:A	1E-10	rs74227709-?	aspartate aminotransferase platelet ratio index in high alcohol intake	G	A	-0.061	0.941	7.73E-01	-0.078	0.925	7.8E-01	3.7E-01	1	0.263431	1
Hyperbilirubinemia	chr7:87450090:T:A	2E-18	rs2109505-?	alanine transaminase levels	T	A	-0.160	0.852	1.92E-01	-0.067	0.935	6.8E-01	3.7E-01	1	0.903597	1
Hyperbilirubinemia	chr2:113999632:G:C	1E-13	rs17046767-G	alanine aminotransferase levels	G	C	0.062	1.064	7.23E-01	0.184	1.202	4.3E-01	3.7E-01	1	0.311369	1

© 2022 Yang W et al. *JAMA Network Open*.

Hyperbilirubinemia	chr8:144506797:G:C	3E-38	rs147998249-	alanine aminotransferase levels	G	C	-11.657	0.000	9.76E-01	1.536	4.644	9.6E-02	3.8E-01	1	NA	NA
Hyperbilirubinemia	chr3:160455812:A:C	8E-14	rs17236494-C	alanine aminotransferase levels;aspartate aminotransferase levels	A	C	-0.016	0.984	8.81E-01	-0.069	0.933	6.7E-01	3.8E-01	1	0.63111	1
Hyperbilirubinemia	chr3:172550013:T:C	2E-20	rs13074711-T	aspartate aminotransferase levels	T	C	0.234	1.263	9.03E-02	0.018	1.018	9.3E-01	3.8E-01	1	0.542247	1
Hyperbilirubinemia	chr6:20404189:T:C	7E-10	rs73382439-C	alanine aminotransferase levels	T	C	0.168	1.183	1.66E-01	0.021	1.022	9.0E-01	3.9E-01	1	0.990681	1

Hyperbilirubinemia	chr8:119175470:A:G	0.000000006	rs2468175-G	aspartate aminotransferase levels	A	G	-0.083	0.920	3.98E-01	-0.050	0.951	7.1E-01	3.9E-01	1	0.460023	1
Hyperbilirubinemia	chr3:33056563:G:A	8E-12	rs11706136-A	aspartate aminotransferase levels	G	A	0.222	1.248	2.76E-02	-0.128	0.880	3.4E-01	3.9E-01	1	0.400102	1
Hyperbilirubinemia	chr12:25255530:A:G	0.00000003	rs12320328-A	alanine aminotransferase levels	A	G	-0.068	0.935	7.21E-01	-0.281	0.755	2.8E-01	4.0E-01	1	0.908671	1
Hyperbilirubinemia	chr10:94775423:A:C	0.000000001	E122A	CPIC:CYP2C19	A	C	-10.444	0.000	9.79E-01	0.698	2.010	5.7E-01	4.0E-01	1	NA	NA
Hyperbilirubinemia	chr19:41012339:C:T	0.000000001	R336C	CPIC:CYP2B6	C	T	-9.359	0.000	9.80E-01	-10.452	0.000	9.8E-01	4.0E-01	1	NA	NA
Hyperbilirubinemia	chr1:150320113:C:T	0.00000002	rs11581793-?	alanine aminotransferase levels	C	T	-1.687	0.185	1.05E-01	-0.173	0.841	8.3E-01	4.0E-01	1	0.731596	1
Hyperbilirubinemia	chr16:80463704:C:A	0.000000003	rs4581712-A	liver enzyme levels (gamma-glutamyl transferase)	C	A	-0.003	0.997	9.79E-01	-0.082	0.921	5.9E-01	4.0E-01	1	0.495644	1

© 2022 Yang W et al. *JAMA Network Open*.

Hyperbilirubinemia	chr4:76495474:A:G	1E-22	rs12500824-A	alanine aminotransferase levels;aspartate aminotransferase levels;alanine transaminase levels	A	G	0.061	1.063	5.34E-01	-0.041	0.960	7.7E-01	4.0E-01	1	0.21926	1
Hyperbilirubinemia	chr10:94981296:A:C	0.000000001	p.I359L	CPIC:CYP2C9	A	C	-0.080	0.924	6.92E-01	0.326	1.386	2.2E-01	4.1E-01	1	0.68771	1
Hyperbilirubinemia	chr14:93998863:G:A	4E-10	rs10145626-A	aspartate aminotransferase levels	G	A	-0.121	0.886	4.05E-01	0.238	1.269	2.4E-01	4.1E-01	1	0.751263	1
Hyperbilirubinemia	chr12:120987058:A:G	7E-45	rs7310409-G	liver enzyme levels (gamma-glutamyl transferase)	A	G	0.041	1.042	6.70E-01	0.140	1.150	3.1E-01	4.1E-01	1	0.953101	1
Hyperbilirubinemia	chr9:34107507:C:T	0.000000003	rs11557154-T	alanine aminotransferase levels	C	T	0.136	1.146	2.67E-01	-0.008	0.992	9.6E-01	4.2E-01	1	0.538744	1
Hyperbilirubinemia	chr12:21130388:G:A	0.000000001	NA	CPIC:SLCO1B1	G	A	0.094	1.098	6.62E-01	0.247	1.280	3.3E-01	4.2E-01	1	0.867927	1
Hyperbilirubinemia	chr2:233260305:C:T	0.00000003	rs6758317-T	bilirubin levels	C	T	-0.226	0.798	5.57E-02	0.004	1.004	9.8E-01	4.2E-01	1	0.101442	1

© 2022 Yang W et al. *JAMA Network Open*.

Hyperbilirubinemia	chr22:36149089:A:T	6E-34	rs132642-T	alanine aminotransferase levels;aspartate aminotransferase levels	A	T	0.271	1.311	9.58E-02	0.051	1.053	8.1E-01	4.2E-01	1	0.057588	1
Hyperbilirubinemia	chr11:47893747:A:G	2E-15	rs34467936-A	aspartate aminotransferase levels	A	G	-0.087	0.917	3.80E-01	0.066	1.068	6.3E-01	4.2E-01	1	0.023042	1
Hyperbilirubinemia	chr16:54381615:G:A	2E-36	rs74367983-A	aspartate aminotransferase levels	G	A	-0.412	0.663	5.60E-02	0.134	1.144	6.5E-01	4.2E-01	1	0.465043	1
Hyperbilirubinemia	chr19:41004381:G:A	0.000000001	R140Q	CPIC:CYP2B6	G	A	0.180	1.197	8.11E-01	0.627	1.872	6.3E-01	4.2E-01	1	NA	NA
Hyperbilirubinemia	chr9:114377802:T:C	1E-40	rs4979372-?	alanine aminotransferase levels;alanine transaminase levels	T	C	-0.036	0.964	6.99E-01	0.124	1.132	3.7E-01	4.2E-01	1	0.124821	1
Hyperbilirubinemia	chr14:72545177:A:G	0.00000002	rs2239222-A	aspartate aminotransferase levels	A	G	0.158	1.171	1.03E-01	-0.158	0.854	2.5E-01	4.3E-01	1	0.544583	1
Hyperbilirubinemia	chr11:119225051:T:G	0.000000007	rs11217192-?	aspartate aminotransferase platelet ratio index in high alcohol intake	T	G	0.036	1.036	7.44E-01	-0.293	0.746	6.5E-02	4.3E-01	1	0.629947	1
Hyperbilirubinemia	chr9:114382224:C:T	1E-51	rs4979373-C	aspartate aminotransferase levels	C	T	-0.048	0.953	6.07E-01	0.107	1.113	4.4E-01	4.4E-01	1	0.128453	1
Hyperbilirubinemia	chr12:26321934:G:A	0.00000002	rs10842708-G	alanine aminotransferase levels	G	A	-0.032	0.968	7.57E-01	0.011	1.011	9.4E-01	4.4E-01	1	0.934778	1
Hyperbilirubinemia	chr2:5092045:A:G	0.00000001	rs72631567-?	drug-induced liver injury;drug-induced cholestatic/mixed liver injury	A	G	0.001	1.001	9.97E-01	0.332	1.393	2.2E-01	4.4E-01	1	0.411337	1
Hyperbilirubinemia	chr8:9320758:A:T	2E-10	rs6984305-A	liver enzyme levels (alkaline phosphatase)	A	T	0.005	1.005	9.72E-01	-0.126	0.882	5.3E-01	4.4E-01	1	0.681155	1

© 2022 Yang W et al. *JAMA Network Open*.

Hyperbilirubinemia	chr9:114371911:C:T	2E-18	rs7043196-?	aspartate aminotransferase levels	C	T	-0.051	0.950	5.80E-01	0.086	1.090	5.2E-01	4.4E-01	1	0.106609	1
Hyperbilirubinemia	chr15:72282873:A:T	0.00000005	rs8027647-T	alanine aminotransferase levels	A	T	-0.033	0.968	7.49E-01	-0.114	0.892	4.1E-01	4.4E-01	1	NA	NA
Hyperbilirubinemia	chr9:114383763:C:G	8E-124	rs7041363-C	alanine aminotransferase levels	C	G	-0.030	0.971	7.50E-01	0.124	1.132	3.7E-01	4.4E-01	1	0.142735	1
Hyperbilirubinemia	chr19:41009350:C:T	0.000000001	S259R	CPIC:CYP2B6	C	T	-10.860	0.000	9.84E-01	-11.071	0.000	9.8E-01	4.5E-01	1	NA	NA
Hyperbilirubinemia	chr8:58553547:T:C	1E-11	rs113895159-	aspartate aminotransferase levels	T	C	0.066	1.068	5.14E-01	0.036	1.036	7.9E-01	4.5E-01	1	0.488895	1
Hyperbilirubinemia	chr11:89491285:A:C	0.000000002	rs2289125-C	aspartate aminotransferase levels	A	C	0.021	1.021	8.46E-01	0.039	1.040	8.0E-01	4.5E-01	1	0.087379	1
Hyperbilirubinemia	chr2:27508073:T:C	4E-13	rs1260326-T	liver enzyme levels (gamma-glutamyl transferase);nonalcoholic fatty liver disease;liver fat content (mri proton density fat fraction measure)	T	C	-0.064	0.938	5.03E-01	0.154	1.166	2.7E-01	4.5E-01	1	0.621429	1
Hyperbilirubinemia	chr5:39424526:A:C	0.000000007	rs1818782-?	aspartate aminotransferase levels	A	C	0.017	1.017	8.63E-01	-0.154	0.857	2.6E-01	4.5E-01	1	0.116672	1
Hyperbilirubinemia	chr8:10713981:C:A	2E-19	rs4484649-C	alanine aminotransferase levels	C	A	-0.188	0.828	4.58E-02	0.104	1.110	4.3E-01	4.5E-01	1	0.735841	1
Hyperbilirubinemia	chr2:233768740:G:A	3E-81	rs6431630-?	total bilirubin levels	G	A	-0.141	0.869	3.96E-01	-0.064	0.938	7.7E-01	4.5E-01	1	0.3065	1
Hyperbilirubinemia	chr16:86063872:C:T	0.00000001	rs56360131-T	aspartate aminotransferase levels	C	T	0.174	1.190	1.49E-01	-0.038	0.963	8.2E-01	4.6E-01	1	0.555689	1
Hyperbilirubinemia	chr8:8804171:C:G	1E-23	rs12544992-G	aspartate aminotransferase levels	C	G	0.033	1.033	7.46E-01	-0.096	0.909	4.8E-01	4.6E-01	1	0.601772	1

© 2022 Yang W et al. *JAMA Network Open*.

Hyperbilirubinemia	chr10:94781859:G:A	0.000000001	splicing defect	CPIC:CYP2C19	G	A	-0.139	0.870	3.09E-01	0.119	1.126	4.9E-01	4.6E-01	1	0.154074	1
Hyperbilirubinemia	chr19:40991441:A:G	0.000000001	M46V	CPIC:CYP2B6	A	G	-0.909	0.403	3.92E-01	-13.197	0.000	9.8E-01	4.6E-01	1	NA	NA
Hyperbilirubinemia	chr19:41006936:G:T	0.000000001	Q172H	CPIC:CYP2B6	G	T	-0.220	0.802	4.13E-02	0.000	1.000	1.0E+00	4.6E-01	1	0.94876	1
Hyperbilirubinemia	chr22:42127608:C:T	0.000000001	p.V338M	CPIC:CYP2D6	C	T	0.124	1.132	8.60E-01	-0.608	0.544	4.3E-01	4.6E-01	1	0.291027	1
Hyperbilirubinemia	chr10:18221519:G:A	3E-14	rs2489201-?	aspartate aminotransferase levels	G	A	-0.135	0.874	1.89E-01	0.053	1.055	7.1E-01	4.7E-01	1	0.892021	1
Hyperbilirubinemia	chr14:77051443:G:A	0.00000003	rs75004998-G	alanine aminotransferase levels	G	A	0.031	1.032	7.49E-01	-0.001	0.999	9.9E-01	4.7E-01	1	0.289659	1
Hyperbilirubinemia	chr22:43995806:G:A	2E-11	rs2143571-A	nonalcoholic fatty liver disease	G	A	-0.022	0.978	8.42E-01	-0.293	0.746	8.2E-02	4.7E-01	1	0.787553	1
Hyperbilirubinemia	chr19:48702915:C:T	8E-10	rs516246-T	liver enzyme levels (gamma-glutamyl transferase)	C	T	0.026	1.027	7.92E-01	-0.116	0.891	4.1E-01	4.7E-01	1	0.227585	1
Hyperbilirubinemia	chr12:111280427:C:A	1E-30	rs79105258-?	alanine transaminase levels;alanine aminotransferase levels;aspartate aminotransferase levels	C	A	0.118	1.126	8.55E-01	-0.763	0.466	2.9E-01	4.7E-01	1	0.435376	1
Hyperbilirubinemia	chr8:125473052:T:A	3E-23	rs2954027-T	aspartate aminotransferase levels	T	A	0.037	1.038	6.86E-01	-0.075	0.928	5.7E-01	4.7E-01	1	0.336871	1
Hyperbilirubinemia	chr2:27512105:G:A	4E-24	rs6547692-G	aspartate aminotransferase levels	G	A	-0.075	0.928	4.27E-01	0.150	1.162	2.7E-01	4.8E-01	1	0.478324	1
Hyperbilirubinemia	chr1:97595149:T:C	0.000000001	p.K290E	CPIC:DPYD	T	C	-10.281	0.000	9.81E-01	-10.293	0.000	9.8E-01	4.8E-01	1	NA	NA
Hyperbilirubinemia	chr1:28245806:G:C	2E-13	rs12129745-G	aspartate aminotransferase levels	G	C	0.035	1.035	7.37E-01	-0.090	0.914	5.4E-01	4.8E-01	1	0.629604	1
Hyperbilirubinemia	chr5:53980471:G:A	6E-10	rs1664781-A	alanine aminotransferase levels	G	A	-0.194	0.824	7.01E-02	0.137	1.146	3.7E-01	4.8E-01	1	0.439822	1
Hyperbilirubinemia	chrX:154326058:C:G	0.000000009	rs766420-G	bilirubin levels	C	G	-0.126	0.882	1.87E-01	-0.032	0.969	7.9E-01	4.8E-01	1	0.367275	1

© 2022 Yang W et al. *JAMA Network Open*.

Hyperbilirubinemia	chr1:183833125:T:C	1E-34	rs2491441-C	aspartate aminotransferase levels	T	C	0.003	1.003	9.83E-01	-0.016	0.984	9.2E-01	4.9E-01	1	0.220525	1
Hyperbilirubinemia	chr12:55991020:G:A	8E-11	rs705699-G	aspartate aminotransferase levels	G	A	-0.083	0.921	4.13E-01	0.289	1.336	3.2E-02	4.9E-01	1	0.05669	1
Hyperbilirubinemia	chr19:39248147:C:T	0.000000001	unknown (ups	CPIC:IFNL3	C	T	0.120	1.127	2.39E-01	-0.186	0.830	1.9E-01	4.9E-01	1	0.726949	1
Hyperbilirubinemia	chr5:31020414:A:G	0.000000001	rs6888304-A	liver enzyme levels (gamma-glutamyl transferase)	A	G	0.070	1.073	5.34E-01	-0.303	0.739	5.7E-02	4.9E-01	1	0.948602	1
Hyperbilirubinemia	chr13:73550539:G:A	1E-10	rs12876036-G	aspartate aminotransferase levels	G	A	-0.027	0.973	7.76E-01	-0.010	0.990	9.4E-01	4.9E-01	1	0.563516	1
Hyperbilirubinemia	chr1:205075211:A:G	1E-17	rs11240351-G	aspartate aminotransferase levels	A	G	-0.123	0.884	2.14E-01	-0.098	0.906	4.8E-01	4.9E-01	1	0.835054	1
Hyperbilirubinemia	chr14:103106478:A:G	6E-29	rs944002-G	liver enzyme levels (gamma-glutamyl transferase)	A	G	0.121	1.128	2.70E-01	-0.169	0.844	2.9E-01	4.9E-01	1	0.476748	1
Hyperbilirubinemia	chr6:116003979:C:T	6E-12	rs3756772-T	alanine aminotransferase levels	C	T	0.013	1.013	8.95E-01	-0.104	0.901	4.4E-01	4.9E-01	1	0.387004	1
Hyperbilirubinemia	chr7:7233809:G:A	2E-11	rs1008897-G	aspartate aminotransferase levels	G	A	0.002	1.002	9.87E-01	-0.143	0.867	3.4E-01	5.0E-01	1	0.042174	1
Hyperbilirubinemia	chr12:109631385:G:T	0.00000003	rs11067592-?	aspartate aminotransferase levels	G	T	-0.121	0.886	9.19E-01	-0.290	0.748	7.5E-01	5.0E-01	1	NA	NA
Hyperbilirubinemia	chr11:5679844:C:A	7E-24	rs11601507-A	alanine aminotransferase levels;aspartate aminotransferase levels	C	A	0.097	1.102	5.70E-01	0.019	1.019	9.4E-01	5.0E-01	1	0.716153	1

© 2022 Yang W et al. *JAMA Network Open*.

Hyperbilirubinemia	chr9:4763176:T:C	2E-13	rs385893-?	aspartate aminotransferase platelet ratio index in high alcohol intake	T	C	-0.067	0.935	4.90E-01	0.027	1.028	8.4E-01	5.0E-01	1	0.584468	1
Hyperbilirubinemia	chr2:233729143:A:G	1E-10	rs28898617-?	total bilirubin levels	A	G	0.375	1.454	7.67E-01	-0.006	0.994	9.9E-01	5.0E-01	1	NA	NA
Hyperbilirubinemia	chr12:21008244:A:G	3E-18	rs76300959-?	total bilirubin levels	A	G	-0.118	0.888	2.52E-01	-0.069	0.933	6.4E-01	5.0E-01	1	0.005288	0.866667
Hyperbilirubinemia	chr2:233527423:C:T	7E-12	rs36075906-T	bilirubin levels	C	T	0.134	1.143	3.86E-01	-0.189	0.828	4.6E-01	5.1E-01	1	0.254873	1
Hyperbilirubinemia	chr9:133458632:A:G	8E-21	rs4962153-A	liver enzyme levels	A	G	-0.052	0.949	6.91E-01	0.239	1.270	2.0E-01	5.1E-01	1	0.160215	1

Hyperbilirubinemia	chr3:56831417:T:C	1E-14	rs11925835-?	aspartate aminotransferase platelet ratio index in high alcohol intake	T	C	-0.183	0.833	6.41E-02	0.142	1.153	2.9E-01	5.1E-01	1	0.945874	1
Hyperbilirubinemia	chr22:42129809:T:C	0.000000001	p.H94R	CPIC:CYP2D6	T	C	0.001	1.001	9.94E-01	-0.157	0.855	4.6E-01	5.1E-01	1	0.024697	1
Hyperbilirubinemia	chr16:58037060:T:A	1E-10	rs12928392-T	alanine aminotransferase levels	T	A	0.018	1.018	8.67E-01	-0.111	0.895	4.7E-01	5.1E-01	1	0.2559	1
Hyperbilirubinemia	chr10:88047609:T:C	0.000000006	rs10887777-C	alanine aminotransferase levels	T	C	-0.102	0.903	3.52E-01	-0.043	0.958	7.8E-01	5.1E-01	1	0.658472	1
Hyperbilirubinemia	chr22:42130710:G:A	0.000000001	p.R28C	CPIC:CYP2D6	G	A	1.000	2.718	1.73E-01	-13.173	0.000	9.8E-01	5.2E-01	1	NA	NA
Hyperbilirubinemia	chr21:28760989:T:C	0.000000002	rs2832059-?	alanine aminotransferase levels	T	C	0.197	1.218	2.39E-01	-0.181	0.834	4.3E-01	5.2E-01	1	0.825222	1
Hyperbilirubinemia	chr1:2244114:C:T	0.000000002	rs142451335-	alanine aminotransferase levels	C	T	-0.066	0.936	7.44E-01	0.244	1.276	3.4E-01	5.2E-01	1	0.030271	1
Hyperbilirubinemia	chr1:16184399:C:T	1E-39	rs36086195-T	aspartate aminotransferase levels;alanine aminotransferase levels	C	T	0.002	1.002	9.84E-01	0.072	1.075	6.0E-01	5.2E-01	1	0.966129	1
Hyperbilirubinemia	chr1:94202414:T:C	2E-13	rs1541098-T	aspartate aminotransferase levels	T	C	-0.006	0.994	9.55E-01	0.015	1.015	9.2E-01	5.2E-01	1	0.648238	1

© 2022 Yang W et al. *JAMA Network Open*.

Hyperbilirubinemia	chr18:58463061:A:C	9E-15	rs7243073-A	aspartate aminotransferase levels	A	C	0.130	1.139	2.31E-01	-0.077	0.926	6.0E-01	5.2E-01	1	0.359908	1
Hyperbilirubinemia	chr5:56508725:A:C	2E-14	rs40270-C	alanine aminotransferase levels	A	C	-0.138	0.871	1.98E-01	-0.088	0.916	5.5E-01	5.2E-01	1	0.852558	1
Hyperbilirubinemia	chr6:35316181:T:C	1E-11	rs13197551-T	alanine aminotransferase levels	T	C	0.004	1.004	9.72E-01	-0.168	0.845	2.8E-01	5.3E-01	1	0.377927	1

© 2022 Yang W et al. *JAMA Network Open*.

Hyperbilirubinemia	chr9:6665010:C:T	2E-11	rs1658972-C	alanine aminotransferase levels;alanine transaminase levels	C	T	-0.028	0.972	8.42E-01	-0.024	0.976	9.0E-01	5.3E-01	1	0.990618	1
Hyperbilirubinemia	chr10:119711739:C:G	4E-22	rs196210-G	aspartate aminotransferase levels	C	G	-0.125	0.883	2.12E-01	-0.083	0.920	5.5E-01	5.3E-01	1	0.445594	1
Hyperbilirubinemia	chr21:39183635:T:C	6E-11	rs117143374-	alanine aminotransferase levels	T	C	0.100	1.105	4.91E-01	-0.282	0.755	1.9E-01	5.3E-01	1	0.122276	1
Hyperbilirubinemia	chr2:233257131:G:A	5E-37	rs2119503-A	bilirubin levels	G	A	-0.156	0.856	2.24E-01	-0.107	0.899	5.5E-01	5.3E-01	1	0.100665	1
Hyperbilirubinemia	chr12:56639569:C:T	4E-12	rs2950388-C	alanine aminotransferase levels	C	T	0.061	1.063	5.68E-01	-0.067	0.935	6.6E-01	5.3E-01	1	0.794399	1
Hyperbilirubinemia	chr21:29349061:T:C	8E-11	rs412662-C	aspartate aminotransferase levels	T	C	-0.035	0.966	7.13E-01	0.139	1.149	3.0E-01	5.3E-01	1	0.862136	1
Hyperbilirubinemia	chr22:42130692:G:A	0.000000001	p.P34S	CPIC:CYP2D6	G	A	0.004	1.004	9.72E-01	-0.162	0.850	3.6E-01	5.4E-01	1	0.153341	1
Hyperbilirubinemia	chr17:75771356:A:G	2E-11	rs4485425-G	alanine aminotransferase levels	A	G	0.101	1.106	3.27E-01	0.214	1.239	1.4E-01	5.4E-01	1	0.003722	0.764103
Hyperbilirubinemia	chr2:43839108:G:C	5E-11	rs11887534-C	alanine aminotransferase levels	G	C	0.184	1.202	3.03E-01	-0.517	0.596	1.2E-01	5.4E-01	1	0.201862	1
Hyperbilirubinemia	chr17:7282460:C:A	2E-12	rs117643180-	alanine aminotransferase levels;aspartate aminotransferase levels	C	A	0.015	1.015	9.71E-01	0.462	1.587	2.7E-01	5.4E-01	1	0.769693	1
Hyperbilirubinemia	chr20:39913927:C:T	0.00000001	rs17803745-C	aspartate aminotransferase levels	C	T	0.015	1.016	8.75E-01	0.075	1.078	6.0E-01	5.4E-01	1	0.593497	1
Hyperbilirubinemia	chr4:23924364:G:A	2E-10	rs11932940-A	aspartate aminotransferase levels	G	A	0.089	1.093	3.88E-01	0.014	1.014	9.2E-01	5.4E-01	1	0.639581	1
Hyperbilirubinemia	chr19:15897578:A:C	0.000000001	W12G	CPIC:CYP4F2	A	C	-0.033	0.967	8.04E-01	0.005	1.005	9.8E-01	5.4E-01	1	0.654546	1

© 2022 Yang W et al. *JAMA Network Open*.

Hyperbilirubinemia	chr2:191252512:C:T	1E-11	rs13030978-T	liver enzyme levels (gamma-glutamyl transferase)	C	T	-0.183	0.833	8.80E-02	-0.059	0.942	6.8E-01	5.4E-01	1	0.861267	1
Hyperbilirubinemia	chr10:94981301:C:G	0.000000001	p.D360E	CPIC:CYP2C9	C	G	-10.493	0.000	9.81E-01	-11.311	0.000	9.7E-01	5.4E-01	1	NA	NA
Hyperbilirubinemia	chr6:18139710:G:A	0.000000001	S125L	CPIC:TPMT	G	A	-10.237	0.000	9.78E-01	-11.950	0.000	9.8E-01	5.4E-01	1	NA	NA
Hyperbilirubinemia	chr14:77043529:G:A	0.000000003	rs11159247-G	aspartate aminotransferase levels	G	A	0.021	1.021	8.34E-01	0.027	1.027	8.5E-01	5.4E-01	1	0.3302	1
Hyperbilirubinemia	chr2:102130853:A:G	0.00000001	rs1024794-G	alanine aminotransferase levels	A	G	-0.101	0.904	3.09E-01	-0.061	0.941	6.8E-01	5.5E-01	1	0.250909	1
Hyperbilirubinemia	chr22:42129819:G:T	0.000000001	p.L91M	CPIC:CYP2D6	G	T	0.003	1.003	9.84E-01	-0.142	0.868	5.0E-01	5.5E-01	1	0.025323	1
Hyperbilirubinemia	chr10:100035604:T:A	0.000000004	rs10883437-T	liver enzyme levels (alanine transaminase)	T	A	-0.004	0.996	9.65E-01	0.071	1.074	6.0E-01	5.5E-01	1	0.55572	1
Hyperbilirubinemia	chr17:70468676:T:A	1E-10	rs717118-T	alanine aminotransferase levels	T	A	-0.066	0.937	4.82E-01	0.125	1.133	3.6E-01	5.5E-01	1	0.307526	1
Hyperbilirubinemia	chr10:99372525:A:C	1E-25	rs10509735-?	aspartate aminotransferase levels	A	C	-0.028	0.973	7.81E-01	-0.076	0.927	5.9E-01	5.6E-01	1	0.913582	1
Hyperbilirubinemia	chr12:121004867:T:C	2E-10	rs1169313-C	liver enzyme levels	T	C	-0.047	0.954	6.33E-01	-0.093	0.911	5.1E-01	5.6E-01	1	0.426834	1
Hyperbilirubinemia	chr2:233760973:C:A	0.000000001	P229Q	CPIC:UGT1A1	C	A	0.154	1.167	9.00E-01	-12.054	0.000	9.7E-01	5.6E-01	1	NA	NA
Hyperbilirubinemia	chr1:97515865:C:T	0.000000001	p.S534N	CPIC:DPYD	C	T	-0.218	0.804	5.94E-01	0.344	1.411	4.4E-01	5.6E-01	1	0.480253	1
Hyperbilirubinemia	chr6:130024960:G:A	6E-12	rs7740188-A	alanine aminotransferase levels;aspartate aminotransferase levels	G	A	0.135	1.145	2.08E-01	-0.221	0.801	1.4E-01	5.6E-01	1	0.622389	1
Hyperbilirubinemia	chr3:157080986:T:C	6E-13	rs900400-C	alanine aminotransferase levels	T	C	-0.062	0.940	5.09E-01	-0.107	0.899	4.2E-01	5.7E-01	1	0.401435	1
Hyperbilirubinemia	chr19:15879621:C:T	0.000000001	V433M	CPIC:CYP4F2	C	T	-0.019	0.981	8.58E-01	-0.058	0.943	6.9E-01	5.7E-01	1	0.18198	1
Hyperbilirubinemia	chr15:51201770:G:A	2E-13	rs11070841-A	aspartate aminotransferase levels	G	A	-0.064	0.938	5.98E-01	0.021	1.022	8.9E-01	5.7E-01	1	0.971845	1

© 2022 Yang W et al. *JAMA Network Open*.

Hyperbilirubinemia	chr22:37073551:G:A	0.000000002	rs4820268-G	liver fibrosis and steatohepatitis severity (mri ct1 measure)	G	A	0.001	1.001	9.93E-01	0.196	1.217	1.4E-01	5.7E-01	1	0.495119	1
Hyperbilirubinemia	chr1:97573919:G:A	0.000000001	p.R394W	CPIC:DPYD	G	A	-9.901	0.000	9.79E-01	-11.411	0.000	9.7E-01	5.8E-01	1	NA	NA
Hyperbilirubinemia	chr7:87445648:T:C	4E-42	rs1468615-T	alanine aminotransferase levels	T	C	-0.114	0.892	3.60E-01	0.012	1.012	9.4E-01	5.8E-01	1	0.531516	1
Hyperbilirubinemia	chr18:57655270:C:T	9E-10	rs12968116-C	liver enzyme levels (gamma-glutamyl transferase)	C	T	0.043	1.044	8.00E-01	-0.128	0.880	5.9E-01	5.8E-01	1	0.870544	1
Hyperbilirubinemia	chr19:41004125:G:A	0.000000001	G99E	CPIC:CYP2B6	G	A	-11.031	0.000	9.77E-01	-10.800	0.000	9.8E-01	5.8E-01	1	NA	NA
Hyperbilirubinemia	chr14:54771591:G:A	4E-16	rs7155922-G	aspartate aminotransferase levels	G	A	0.079	1.083	4.02E-01	0.127	1.135	3.4E-01	5.8E-01	1	0.122166	1
Hyperbilirubinemia	chr5:39533216:T:A	1E-29	rs2910953-A	aspartate aminotransferase levels	T	A	0.032	1.032	7.69E-01	-0.058	0.944	7.1E-01	5.9E-01	1	0.313514	1
Hyperbilirubinemia	chr16:80464197:T:C	2E-15	rs7189522-T	aspartate aminotransferase levels	T	C	0.003	1.003	9.76E-01	0.051	1.052	7.3E-01	5.9E-01	1	0.831525	1
Hyperbilirubinemia	chr11:16227964:A:G	6E-10	rs10832570-A	alanine aminotransferase levels	A	G	0.072	1.075	4.47E-01	-0.071	0.932	6.0E-01	5.9E-01	1	0.252241	1
Hyperbilirubinemia	chr11:16228637:C:T	1E-10	rs1401454-C	aspartate aminotransferase levels	C	T	0.072	1.075	4.47E-01	-0.071	0.932	6.0E-01	5.9E-01	1	0.252241	1
Hyperbilirubinemia	chr6:95634240:T:C	0.000000003	rs35064159-C	aspartate aminotransferase levels	T	C	0.182	1.199	3.13E-01	-0.211	0.810	4.7E-01	5.9E-01	1	0.162342	1
Hyperbilirubinemia	chr8:102650624:T:C	6E-17	rs13275089-T	alanine aminotransferase levels	T	C	-0.066	0.936	5.20E-01	-0.153	0.858	2.9E-01	5.9E-01	1	0.176052	1
Hyperbilirubinemia	chr10:94761900:C:T	0.000000001	5' region	CPIC:CYP2C19	C	T	0.180	1.198	1.41E-01	-0.086	0.918	6.3E-01	6.0E-01	1	0.107197	1
Hyperbilirubinemia	chr9:133261662:G:A	5E-25	rs687621-G	alanine aminotransferase levels	G	A	0.019	1.019	8.51E-01	0.009	1.009	9.5E-01	6.0E-01	1	0.380274	1

© 2022 Yang W et al. *JAMA Network Open*.

Hyperbilirubinemia	chr10:94762706:A:G	0.000000001	M1V	CPIC:CYP2C19	A	G	0.189	1.208	8.32E-01	-13.065	0.000	9.8E-01	6.0E-01	1	NA	NA

Hyperbilirubinemia	chr9:95450326:C:T	0.00000002	rs2282043-C	alanine aminotransferase levels	C	T	-0.015	0.985	9.36E-01	0.098	1.103	7.1E-01	6.0E-01	1	0.760412	1
Hyperbilirubinemia	chr8:125478730:A:T	2E-16	rs2954029-?	alanine transaminase levels	A	T	0.059	1.061	5.29E-01	-0.092	0.912	4.9E-01	6.0E-01	1	0.306332	1
Hyperbilirubinemia	chr5:150064431:C:T	0.000000001	rs216141-C	aspartate aminotransferase levels	C	T	-0.075	0.928	4.58E-01	-0.060	0.942	6.7E-01	6.0E-01	1	0.114553	1
Hyperbilirubinemia	chr19:40991224:T:C	0.000000001	5' region	CPIC:CYP2B6	T	C	0.410	1.506	3.26E-01	-0.638	0.528	4.1E-01	6.0E-01	1	0.310542	1
Hyperbilirubinemia	chr12:3231491:C:T	0.00000004	rs7976853-T	aspartate aminotransferase levels	C	T	-0.077	0.926	6.43E-01	0.313	1.368	1.5E-01	6.0E-01	1	0.502818	1
Hyperbilirubinemia	chr11:2987174:G:A	2E-11	rs12806061-G	aspartate aminotransferase levels	G	A	-0.088	0.916	3.84E-01	0.053	1.055	7.2E-01	6.0E-01	1	0.075548	1
Hyperbilirubinemia	chr5:73055235:C:G	9E-37	rs1200503-C	aspartate aminotransferase levels	C	G	-0.099	0.906	2.96E-01	0.251	1.286	6.5E-02	6.0E-01	1	0.458874	1
Hyperbilirubinemia	chr22:37066896:A:G	0.00000005	rs855791-?	total bilirubin levels	A	G	0.024	1.024	8.00E-01	0.192	1.212	1.6E-01	6.0E-01	1	NA	NA
Hyperbilirubinemia	chr10:94842866:A:G	0.000000001	I331V	CPIC:CYP2C19	A	G	0.101	1.106	6.23E-01	-0.239	0.787	3.8E-01	6.0E-01	1	0.802955	1
Hyperbilirubinemia	chr11:62432704:C:T	8E-12	rs17145884-C	alanine aminotransferase levels	C	T	0.075	1.078	5.11E-01	-0.189	0.827	2.9E-01	6.0E-01	1	0.031047	1
Hyperbilirubinemia	chr12:120878449:A:G	1E-15	rs656933-A	aspartate aminotransferase levels	A	G	-0.116	0.890	2.71E-01	0.046	1.047	7.5E-01	6.0E-01	1	0.507945	1
Hyperbilirubinemia	chr1:209942767:G:A	0.00000002	rs2205986-?	drug-induced liver injury in interferon-beta-treated multiple sclerosis	G	A	-0.084	0.919	6.33E-01	0.056	1.058	8.3E-01	6.1E-01	1	0.070381	1
Hyperbilirubinemia	chr5:31021251:A:G	6E-25	rs10075805-A	alanine aminotransferase levels;aspartate aminotransferase levels	A	G	0.053	1.054	6.54E-01	-0.337	0.714	4.3E-02	6.1E-01	1	0.959326	1

© 2022 Yang W et al. *JAMA Network Open*.

Hyperbilirubinemia	chr16:252162:A:G	2E-12	rs151330263-	bilirubin levels	A	G	0.210	1.234	8.56E-01	0.377	1.457	6.6E-01	6.1E-01	1	NA	NA
Hyperbilirubinemia	chr12:21202555:C:G	0.000000001	F400L	CPIC:SLCO1B1	C	G	-0.318	0.727	7.82E-01	-12.627	0.000	9.7E-01	6.2E-01	1	NA	NA

Hyperbilirubinemia	chr3:12288912:G:A	0.00000002	rs17036170-?	drug-induced liver injury	G	A	-0.134	0.874	7.79E-01	-0.151	0.859	8.2E-01	6.2E-01	1	0.349727	1
Hyperbilirubinemia	chr5:140695889:C:T	0.00000002	rs17119056-T	aspartate aminotransferase levels	C	T	0.156	1.168	3.49E-01	-0.200	0.819	4.4E-01	6.2E-01	1	0.810216	1
Hyperbilirubinemia	chr16:72186474:G:C	0.000000005	rs7186908-C	liver enzyme levels (alkaline phosphatase)	G	C	-0.053	0.949	6.49E-01	0.199	1.220	1.9E-01	6.3E-01	1	0.201899	1
Hyperbilirubinemia	chr1:220801203:T:C	3E-10	rs10863565-C	aspartate aminotransferase levels	T	C	-0.161	0.852	1.72E-01	0.141	1.152	4.3E-01	6.3E-01	1	0.827771	1
Hyperbilirubinemia	chr12:111433337:A:T	1E-26	rs4766462-A	alanine aminotransferase levels	A	T	-0.018	0.982	8.68E-01	-0.032	0.969	8.3E-01	6.3E-01	1	0.398318	1
Hyperbilirubinemia	chr1:97305364:C:T	0.000000001	p.V732I	CPIC:DPYD	C	T	0.004	1.004	9.87E-01	0.545	1.724	6.0E-02	6.4E-01	1	0.234509	1
Hyperbilirubinemia	chr6:18130762:C:T	0.000000001	R215H	CPIC:TPMT	C	T	0.442	1.555	6.98E-01	-12.646	0.000	9.8E-01	6.4E-01	1	NA	NA
Hyperbilirubinemia	chr17:81513660:T:C	7E-25	rs11657440-C	alanine aminotransferase levels	T	C	-0.062	0.940	5.38E-01	-0.061	0.941	6.8E-01	6.4E-01	1	0.263732	1
Hyperbilirubinemia	chr13:113849020:T:C	7E-16	rs6602909-C	aspartate aminotransferase levels	T	C	0.094	1.099	3.68E-01	-0.257	0.773	7.7E-02	6.4E-01	1	0.498931	1
Hyperbilirubinemia	chr1:97549726:G:C	0.000000001	p.P453R	CPIC:DPYD	G	C	-10.471	0.000	9.81E-01	-11.411	0.000	9.7E-01	6.4E-01	1	NA	NA
Hyperbilirubinemia	chr16:11186056:G:A	8E-10	rs193759-A	aspartate aminotransferase levels	G	A	-0.010	0.990	9.21E-01	0.061	1.063	6.8E-01	6.4E-01	1	0.948426	1
Hyperbilirubinemia	chr17:66212167:C:G	8E-17	rs1801690-G	aspartate aminotransferase levels	C	G	-0.071	0.932	7.68E-01	-0.420	0.657	2.8E-01	6.4E-01	1	0.910032	1
Hyperbilirubinemia	chr17:75397378:T:A	2E-10	rs2053158-A	aspartate aminotransferase levels	T	A	0.051	1.052	6.88E-01	-0.075	0.927	6.6E-01	6.5E-01	1	0.294721	1

© 2022 Yang W et al. *JAMA Network Open*.

Hyperbilirubinemia	chr9:100569123:T:C	4E-12	rs2800290-C	aspartate aminotransferase levels	T	C	0.063	1.065	5.12E-01	-0.041	0.960	7.7E-01	6.5E-01	1	0.804427	1
Hyperbilirubinemia	chr1:112574591:A:G	6E-10	rs12406530-A	aspartate aminotransferase levels	A	G	-0.062	0.940	5.74E-01	-0.230	0.795	1.7E-01	6.5E-01	1	0.87852	1

Hyperbilirubinemia	chr9:133266456:T:C	5E-44	rs2519093-C	aspartate aminotransferase levels	T	C	-0.054	0.947	6.61E-01	0.081	1.085	6.4E-01	6.5E-01	1	0.594257	1
Hyperbilirubinemia	chr6:32223562:C:T	3E-16	rs115695709-	aspartate aminotransferase levels	C	T	-0.170	0.844	2.80E-01	-0.042	0.959	8.4E-01	6.5E-01	1	0.317358	1
Hyperbilirubinemia	chr10:94775367:A:G	0.000000001	splicing defect	CPIC:CYP2C19	A	G	-0.153	0.858	2.60E-01	0.163	1.177	3.3E-01	6.5E-01	1	0.111293	1
Hyperbilirubinemia	chr11:61836038:C:A	0.000000001	rs174576-?	alanine aminotransferase levels	C	A	0.022	1.022	8.21E-01	0.037	1.038	7.9E-01	6.5E-01	1	0.182589	1
Hyperbilirubinemia	chr3:46130331:C:A	0.000000003	rs75182702-A	aspartate aminotransferase levels	C	A	0.314	1.368	6.64E-02	0.128	1.137	5.9E-01	6.5E-01	1	0.325175	1
Hyperbilirubinemia	chr20:3925431:A:G	0.00000002	rs241597-G	aspartate aminotransferase levels	A	G	-0.065	0.937	6.74E-01	0.020	1.020	9.2E-01	6.6E-01	1	0.865586	1
Hyperbilirubinemia	chr1:159886639:C:T	2E-10	rs2501324-?	bilirubin levels	C	T	0.378	1.460	6.15E-01	-0.890	0.411	4.1E-01	6.6E-01	1	NA	NA
Hyperbilirubinemia	chr8:133543494:T:C	7E-10	rs58504358-T	aspartate aminotransferase levels	T	C	0.005	1.005	9.67E-01	-0.138	0.871	4.2E-01	6.6E-01	1	0.851565	1
Hyperbilirubinemia	chr6:18143724:C:G	0.000000001	A80P	CPIC:TPMT	C	G	0.346	1.414	7.18E-01	-12.046	0.000	9.8E-01	6.6E-01	1	NA	NA
Hyperbilirubinemia	chr3:132479151:T:G	3E-13	rs62292950-T	aspartate aminotransferase levels	T	G	0.136	1.145	2.76E-01	-0.100	0.904	5.7E-01	6.6E-01	1	0.493016	1
Hyperbilirubinemia	chr13:110367115:A:C	0.000000002	rs4773169-A	alanine aminotransferase levels	A	C	0.019	1.019	8.48E-01	0.007	1.007	9.6E-01	6.6E-01	1	0.963442	1
Hyperbilirubinemia	chr4:145873469:T:C	3E-27	rs4547811-C	liver enzyme levels (gamma-glutamyl transferase)	T	C	-0.034	0.967	7.60E-01	0.060	1.061	7.1E-01	6.6E-01	1	0.866879	1

© 2022 Yang W et al. *JAMA Network Open*.

Hyperbilirubinemia	chr19:41009350:C:A	0.000000001	S259R	CPIC:CYP2B6	C	A	0.259	1.296	7.80E-01	-13.187	0.000	9.7E-01	6.7E-01	1	NA	NA
Hyperbilirubinemia	chr8:141219046:A:G	5E-26	rs3739235-G	aspartate aminotransferase levels	A	G	0.066	1.068	5.00E-01	-0.032	0.968	8.2E-01	6.7E-01	1	0.943649	1
Hyperbilirubinemia	chr10:94938683:A:G	0.000000001	p.M1V	CPIC:CYP2C9	A	G	-11.424	0.000	9.76E-01	-11.758	0.000	9.8E-01	6.7E-01	1	NA	NA
Hyperbilirubinemia	chr10:94344908:T:G	0.00000002	rs3758526-G	aspartate aminotransferase levels	T	G	0.030	1.031	8.33E-01	0.061	1.063	7.5E-01	6.7E-01	1	0.226896	1
Hyperbilirubinemia	chr11:126413890:C:T	0.000000002	rs2236653-T	liver enzyme levels (alkaline phosphatase)	C	T	-0.128	0.880	1.83E-01	0.030	1.031	8.2E-01	6.8E-01	1	0.507433	1
Hyperbilirubinemia	chr19:19268740:C:T	1E-71	rs58542926-T	alanine aminotransferase levels;aspartate aminotransferase levels;liver fat content (mri proton density fat fraction measure);liver fibrosis and steatohepatitis severity (mri ct1 measure);nonalcoholic fatty liver disease	C	T	0.005	1.005	9.78E-01	-0.046	0.955	8.5E-01	6.8E-01	1	0.670984	1
Hyperbilirubinemia	chr1:196729333:A:G	4E-10	rs6689009-A	aspartate aminotransferase levels	A	G	0.037	1.038	7.72E-01	0.205	1.228	2.2E-01	6.8E-01	1	0.582838	1
Hyperbilirubinemia	chr1:97450058:C:T	0.000000001	NA	CPIC:DPYD	C	T	-12.703	0.000	9.70E-01	0.435	1.545	7.0E-01	6.8E-01	1	NA	NA
Hyperbilirubinemia	chr2:233934849:C:T	1E-10	rs10490012-T	bilirubin levels	C	T	0.006	1.006	9.55E-01	0.023	1.023	8.8E-01	6.8E-01	1	0.925678	1
Hyperbilirubinemia	chr2:169013785:C:A	9E-31	rs2241339-?	alanine aminotransferase levels;alanine transaminase levels;aspartate aminotransferase levels	C	A	0.052	1.054	7.37E-01	0.077	1.080	7.1E-01	6.8E-01	1	0.867322	1

© 2022 Yang W et al. *JAMA Network Open*.

Hyperbilirubinemia	chr1:97573918:C:A	0.000000001	p.R394L	CPIC:DPYD	C	A	-12.101	0.000	9.75E-01	-10.398	0.000	9.8E-01	6.9E-01	1	NA	NA
Hyperbilirubinemia	chr12:122131417:G:T	5E-25	rs11061602-T	alanine aminotransferase levels	G	T	-0.101	0.904	2.87E-01	-0.026	0.975	8.5E-01	6.9E-01	1	0.182351	1
Hyperbilirubinemia	chr4:102789773:G:A	0.000000006	rs223454-G	alanine aminotransferase levels	G	A	-0.099	0.906	2.90E-01	-0.029	0.972	8.2E-01	6.9E-01	1	0.033858	1
Hyperbilirubinemia	chr3:172541716:C:A	5E-16	rs140822617-	alanine aminotransferase levels	C	A	0.071	1.074	6.06E-01	0.142	1.152	4.3E-01	6.9E-01	1	0.583448	1
Hyperbilirubinemia	chr11:61824890:A:G	0.00000002	rs174566-?	aspartate aminotransferase platelet ratio index in high alcohol intake	A	G	0.055	1.057	5.76E-01	-0.015	0.985	9.2E-01	6.9E-01	1	0.145584	1
Hyperbilirubinemia	chr3:155844283:C:T	0.000000008	rs6441017-C	alanine aminotransferase levels	C	T	0.008	1.008	9.34E-01	0.266	1.305	6.3E-02	6.9E-01	1	0.663164	1
Hyperbilirubinemia	chr7:99665212:C:T	0.000000001	Splicing defect	CPIC:CYP3A5	C	T	-0.468	0.626	5.02E-01	0.484	1.622	3.6E-01	7.0E-01	1	0.176051	1
Hyperbilirubinemia	chr2:112013193:A:G	0.000000001	rs4374383-A	hepatitis c induced liver fibrosis	A	G	0.082	1.085	3.99E-01	-0.067	0.935	6.2E-01	7.0E-01	1	0.258543	1
Hyperbilirubinemia	chr7:73612048:C:T	0.000000003	rs17145750-C	liver enzyme levels (gamma-glutamyl transferase)	C	T	-0.211	0.810	1.55E-01	0.062	1.064	7.3E-01	7.0E-01	1	0.023246	1
Hyperbilirubinemia	chr10:112189906:T:C	1E-30	rs10787429-T	alanine aminotransferase levels;aspartate aminotransferase levels;alanine transaminase levels;alanine transaminase levels in high alcohol intake	T	C	-0.228	0.796	2.69E-02	0.238	1.269	1.4E-01	7.0E-01	1	0.57095	1
Hyperbilirubinemia	chr16:83946924:C:G	1E-19	rs4782568-C	alanine aminotransferase levels	C	G	-0.103	0.902	2.87E-01	0.008	1.008	9.5E-01	7.0E-01	1	0.330997	1

© 2022 Yang W et al. *JAMA Network Open*.

Hyperbilirubinemia	chr4:17878793:A:C	0.000000003	rs7700107-C	alanine aminotransferase levels	A	C	-0.075	0.928	5.82E-01	0.329	1.390	5.8E-02	7.0E-01	1	0.985563	1
Hyperbilirubinemia	chr22:43958231:C:T	1E-18	rs738491-T	nonalcoholic fatty liver disease	C	T	-0.031	0.969	7.54E-01	-0.128	0.880	3.7E-01	7.1E-01	1	0.879411	1
Hyperbilirubinemia	chr1:97679170:T:C	0.000000001	p.K259E	CPIC:DPYD	T	C	0.438	1.549	4.92E-01	-0.162	0.850	8.8E-01	7.1E-01	1	NA	NA
Hyperbilirubinemia	chr12:21176879:C:A	0.000000001	P155T	CPIC:SLCO1B1	C	A	0.061	1.063	6.56E-01	0.001	1.001	1.0E+00	7.1E-01	1	0.070774	1
Hyperbilirubinemia	chr21:29236564:A:G	1E-16	rs2832277-G	alanine aminotransferase levels	A	G	-0.096	0.909	4.24E-01	0.155	1.168	4.1E-01	7.1E-01	1	0.048175	1

© 2022 Yang W et al. *JAMA Network Open*.

Hyperbilirubinemia	chr5:56792072:C:T	1E-10	rs144983009-	alanine aminotransferase levels;alanine transaminase levels	C	T	0.017	1.017	9.28E-01	-0.318	0.727	2.7E-01	7.1E-01	1	0.163129	1
Hyperbilirubinemia	chr9:114367105:C:G	7E-10	rs2787352-?	alanine transaminase levels in high alcohol intake	C	G	-0.040	0.961	6.70E-01	0.153	1.166	2.5E-01	7.1E-01	1	0.023572	1
Hyperbilirubinemia	chr2:168977860:G:A	9E-27	rs72623176-A	alanine aminotransferase levels	G	A	0.030	1.030	8.70E-01	0.029	1.030	9.0E-01	7.1E-01	1	0.950727	1
Hyperbilirubinemia	chr5:149404204:T:G	0.00000003	rs6887452-G	serum bilirubin levels x mediterranean diet adherence interaction in metabolic syndrome	T	G	0.032	1.033	7.47E-01	0.147	1.158	2.7E-01	7.2E-01	1	0.328187	1
Hyperbilirubinemia	chr7:28152756:C:T	3E-20	rs12154248-T	alanine aminotransferase levels	C	T	0.123	1.131	3.64E-01	0.036	1.036	8.6E-01	7.2E-01	1	0.833233	1
Hyperbilirubinemia	chr10:94762760:A:C	0.000000001	I19L	CPIC:CYP2C19	A	C	-12.557	0.000	9.77E-01	0.698	2.010	5.7E-01	7.2E-01	1	NA	NA
Hyperbilirubinemia	chr2:111932403:A:G	0.00000003	rs6755970-A	alanine aminotransferase levels	A	G	0.020	1.020	8.42E-01	-0.066	0.936	6.3E-01	7.2E-01	1	0.113821	1
Hyperbilirubinemia	chr7:117614699:G:C	0.000000001	D1152H	CPIC:CFTR	G	C	-11.948	0.000	9.73E-01	-10.677	0.000	9.8E-01	7.2E-01	1	NA	NA
Hyperbilirubinemia	chr7:92779056:C:T	6E-14	rs445-T	alanine aminotransferase levels	C	T	0.067	1.069	6.11E-01	-0.472	0.624	3.7E-02	7.2E-01	1	0.895803	1
Hyperbilirubinemia	chr3:171007753:G:A	6E-11	rs10513686-A	liver enzyme levels (gamma-glutamyl transferase)	G	A	0.084	1.088	5.29E-01	0.070	1.072	7.1E-01	7.2E-01	1	0.492081	1
Hyperbilirubinemia	chr22:42129130:C:G	0.000000001	p.V136=	CPIC:CYP2D6	C	G	0.052	1.053	5.85E-01	-0.228	0.796	9.0E-02	7.3E-01	1	0.13591	1
Hyperbilirubinemia	chr20:34962143:C:T	8E-15	rs11698868-T	aspartate aminotransferase levels	C	T	0.054	1.056	6.56E-01	-0.049	0.952	7.7E-01	7.3E-01	1	0.692526	1
Hyperbilirubinemia	chr19:33420799:T:C	2E-16	rs57457691-T	aspartate aminotransferase levels	T	C	0.127	1.136	1.85E-01	-0.122	0.885	3.6E-01	7.3E-01	1	0.424539	1

© 2022 Yang W et al. *JAMA Network Open*.

Hyperbilirubinemia	chr18:63181406:T:C	0.00000002	rs4940576-C	aspartate aminotransferase levels	T	C	-0.060	0.942	5.67E-01	-0.071	0.932	6.3E-01	7.3E-01	1	0.74275	1
Hyperbilirubinemia	chr12:20921188:C:T	3E-14	rs2117032-C	bilirubin levels	C	T	-0.124	0.884	2.04E-01	-0.066	0.936	6.4E-01	7.3E-01	1	0.649398	1
Hyperbilirubinemia	chr12:21176804:A:G	0.000000001	N130D	CPIC:SLCO1B1	A	G	0.178	1.194	6.25E-02	0.041	1.042	7.7E-01	7.3E-01	1	0.058157	1
Hyperbilirubinemia	chr8:80890620:G:A	0.00000002	rs7840329-A	aspartate aminotransferase levels	G	A	0.039	1.040	6.94E-01	-0.133	0.875	3.5E-01	7.3E-01	1	0.609155	1
Hyperbilirubinemia	chr7:28143829:T:C	0.00000002	rs4722763-?	alanine aminotransferase levels	T	C	0.123	1.131	3.65E-01	0.036	1.036	8.6E-01	7.3E-01	1	0.833269	1
Hyperbilirubinemia	chr9:129804387:G:A	0.00000002	rs7029757-G	alanine aminotransferase levels	G	A	0.156	1.169	3.74E-01	-0.147	0.863	5.6E-01	7.3E-01	1	0.216664	1
Hyperbilirubinemia	chr7:21480514:G:A	0.000000006	rs10272006-G	aspartate aminotransferase levels in non-alcoholic fatty liver disease	G	A	0.037	1.038	7.22E-01	-0.121	0.886	4.2E-01	7.4E-01	1	0.100235	1
Hyperbilirubinemia	chr6:21939097:G:A	2E-11	rs6938097-G	alanine aminotransferase levels;aspartate aminotransferase levels	G	A	-0.107	0.898	3.35E-01	0.097	1.102	5.4E-01	7.4E-01	1	0.244574	1
Hyperbilirubinemia	chr22:42126611:C:G	0.000000001	p.S486T	CPIC:CYP2D6	C	G	0.046	1.047	6.30E-01	-0.195	0.823	1.5E-01	7.4E-01	1	0.218574	1
Hyperbilirubinemia	chr2:36863090:T:C	0.00000003	rs2110944-C	alanine aminotransferase levels	T	C	-0.062	0.940	5.14E-01	-0.112	0.894	4.2E-01	7.4E-01	1	0.215187	1
Hyperbilirubinemia	chr19:40991367:A:T	0.000000001	Q21L	CPIC:CYP2B6	A	T	-0.700	0.497	5.30E-01	-0.079	0.924	9.5E-01	7.4E-01	1	NA	NA
Hyperbilirubinemia	chr16:80463444:G:C	7E-22	rs28650012-G	alanine aminotransferase levels	G	C	-0.008	0.992	9.43E-01	0.031	1.032	8.3E-01	7.5E-01	1	0.808272	1
Hyperbilirubinemia	chr12:6384185:A:G	0.000000001	rs10849448-A	aspartate aminotransferase levels	A	G	0.091	1.095	4.41E-01	-0.362	0.696	1.9E-02	7.5E-01	1	0.371144	1

© 2022 Yang W et al. *JAMA Network Open*.

Hyperbilirubinemia	chr6:24491247:A:C	6E-26	rs1883415-C	liver enzyme levels (alkaline phosphatase)	A	C	-0.041	0.960	6.91E-01	0.163	1.177	2.5E-01	7.5E-01	1	0.512605	1
Hyperbilirubinemia	chr14:50390914:A:G	3E-13	rs10134101-A	aspartate aminotransferase levels	A	G	-0.194	0.824	3.04E-01	0.097	1.101	7.2E-01	7.5E-01	1	0.511343	1
Hyperbilirubinemia	chr6:32347950:T:C	9E-11	rs910049-A	hepatitis c induced liver cirrhosis	T	C	-0.061	0.941	5.76E-01	0.051	1.053	7.5E-01	7.5E-01	1	0.39062	1
Hyperbilirubinemia	chr12:21196951:A:G	0.000000001	I245V	CPIC:SLCO1B1	A	G	-0.367	0.693	7.42E-01	-13.781	0.000	9.8E-01	7.5E-01	1	NA	NA
Hyperbilirubinemia	chr9:93130444:T:C	1E-22	rs7018885-T	aspartate aminotransferase levels;alanine aminotransferase levels	T	C	-0.037	0.964	7.40E-01	-0.120	0.887	4.4E-01	7.5E-01	1	0.025706	1
Hyperbilirubinemia	chr22:38203357:G:A	6E-12	rs4821764-A	alanine aminotransferase levels	G	A	0.117	1.124	2.18E-01	-0.197	0.821	1.3E-01	7.5E-01	1	0.966015	1
Hyperbilirubinemia	chr18:57461443:G:A	4E-45	rs503581-G	aspartate aminotransferase levels	G	A	-0.182	0.833	2.26E-01	0.006	1.006	9.8E-01	7.6E-01	1	0.573143	1
Hyperbilirubinemia	chr11:61855668:C:T	0.000000003	rs174601-T	liver enzyme levels (alkaline phosphatase)	C	T	0.036	1.037	7.14E-01	-0.025	0.975	8.5E-01	7.6E-01	1	0.606231	1
Hyperbilirubinemia	chr2:233356202:C:G	3E-19	rs1550532-?	bilirubin levels	C	G	0.005	1.005	9.58E-01	0.267	1.306	8.0E-02	7.6E-01	1	0.105633	1
Hyperbilirubinemia	chr12:123409363:T:C	2E-15	rs28642812-C	alanine aminotransferase levels	T	C	-0.105	0.900	2.97E-01	0.038	1.039	8.0E-01	7.6E-01	1	0.342392	1
Hyperbilirubinemia	chr16:31096368:C:T	0.000000001	5' region	CPIC:VKORC1	C	T	-0.053	0.949	5.82E-01	-0.022	0.978	8.7E-01	7.6E-01	1	0.935859	1
Hyperbilirubinemia	chr10:17839303:A:C	2E-42	rs377536650-	aspartate aminotransferase levels	A	C	-0.014	0.986	8.86E-01	-0.002	0.998	9.9E-01	7.6E-01	1	0.756458	1
Hyperbilirubinemia	chr19:38268463:T:C	0.00000001	rs3786876-C	alanine aminotransferase levels	T	C	0.089	1.093	4.94E-01	-0.164	0.849	4.2E-01	7.6E-01	1	0.696263	1

© 2022 Yang W et al. *JAMA Network Open*.

Hyperbilirubinemia	chr20:34545380:A:C	1E-18	rs6088521-C	alanine aminotransferase levels	A	C	-0.078	0.925	4.24E-01	-0.094	0.910	5.0E-01	7.6E-01	1	0.499584	1
Hyperbilirubinemia	chr12:21176827:G:A	0.000000001	S137=	CPIC:SLCO1B1	G	A	0.057	1.059	6.78E-01	0.000	1.000	1.0E+00	7.6E-01	1	0.093723	1
Hyperbilirubinemia	chr2:85188306:A:T	1E-13	rs2568207-T	aspartate aminotransferase levels	A	T	-0.033	0.968	7.31E-01	0.088	1.092	5.1E-01	7.7E-01	1	0.701251	1
Hyperbilirubinemia	chr10:17933413:T:C	2E-19	rs10827785-?	aspartate aminotransferase levels	T	C	0.025	1.026	8.07E-01	0.159	1.173	2.7E-01	7.7E-01	1	0.120453	1
Hyperbilirubinemia	chr2:113083453:A:G	5E-16	rs6734238-A	aspartate aminotransferase levels	A	G	0.052	1.053	5.94E-01	0.075	1.078	5.9E-01	7.7E-01	1	0.127226	1
Hyperbilirubinemia	chr6:36679903:T:C	1E-10	rs4135240-T	aspartate aminotransferase levels	T	C	-0.144	0.866	1.54E-01	0.072	1.074	6.1E-01	7.7E-01	1	0.023312	1
Hyperbilirubinemia	chr6:32615700:C:T	7E-38	rs28383223-T	alanine aminotransferase levels	C	T	-0.013	0.987	8.90E-01	0.033	1.034	8.1E-01	7.7E-01	1	0.562407	1
Hyperbilirubinemia	chr9:133263862:A:C	2E-30	rs657152-T	liver enzyme levels	A	C	0.040	1.041	6.93E-01	0.004	1.004	9.8E-01	7.7E-01	1	0.404569	1
Hyperbilirubinemia	chr10:94775489:G:A	0.000000001	R144H	CPIC:CYP2C19	G	A	-11.324	0.000	9.74E-01	0.109	1.116	9.2E-01	7.7E-01	1	NA	NA
Hyperbilirubinemia	chr1:97078987:G:T	0.000000001	p.P1023T	CPIC:DPYD	G	T	0.446	1.562	7.03E-01	-13.630	0.000	9.8E-01	7.7E-01	1	NA	NA
Hyperbilirubinemia	chr2:36922355:T:C	0.00000004	rs11124554-?	alanine transaminase levels	T	C	0.062	1.064	5.10E-01	0.184	1.202	1.9E-01	7.7E-01	1	0.260962	1
Hyperbilirubinemia	chr7:135197671:T:A	6E-14	rs292580-A	aspartate aminotransferase levels	T	A	-0.009	0.991	9.35E-01	-0.026	0.974	8.6E-01	7.7E-01	1	0.294784	1
Hyperbilirubinemia	chr13:110647653:T:C	3E-11	rs421491-T	aspartate aminotransferase levels	T	C	-0.110	0.895	5.59E-01	0.073	1.076	7.6E-01	7.7E-01	1	0.94682	1
Hyperbilirubinemia	chr7:73586691:C:T	7E-16	rs35173225-T	alanine aminotransferase levels	C	T	-0.282	0.755	1.05E-01	0.025	1.025	9.1E-01	7.7E-01	1	0.02813	1
Hyperbilirubinemia	chr12:21130019:T:G	4E-14	rs4149014-G	bilirubin levels	T	G	-0.083	0.920	7.97E-01	-0.036	0.964	9.3E-01	7.8E-01	1	0.775703	1
Hyperbilirubinemia	chr10:100101678:G:A	0.00000002	rs11597390-A	liver enzyme levels	G	A	-0.044	0.957	6.59E-01	-0.018	0.982	9.0E-01	7.8E-01	1	0.178021	1

© 2022 Yang W et al. *JAMA Network Open*.

Hyperbilirubinemia	chr18:58419588:C:T	2E-38	rs9959832-C	alanine aminotransferase levels	C	T	0.099	1.104	4.11E-01	0.054	1.056	7.4E-01	7.8E-01	1	0.176875	1
Hyperbilirubinemia	chr6:139522446:T:C	0.000000008	rs636202-T	alanine aminotransferase levels	T	C	0.043	1.043	6.55E-01	0.006	1.006	9.7E-01	7.8E-01	1	0.036259	1
Hyperbilirubinemia	chr10:94942309:G:A	0.000000001	p.R150H; p.R1	CPIC:CYP2C9	G	A	-0.294	0.745	7.95E-01	0.160	1.173	8.9E-01	7.9E-01	1	NA	NA
Hyperbilirubinemia	chr21:28754160:A:G	8E-11	rs73186036-?	alanine transaminase levels	A	G	0.077	1.080	5.50E-01	-0.107	0.898	5.5E-01	7.9E-01	1	0.564327	1
Hyperbilirubinemia	chr1:161531185:A:G	8E-11	rs6700241-A	alanine aminotransferase levels	A	G	0.053	1.055	6.52E-01	-0.244	0.784	1.5E-01	7.9E-01	1	0.968486	1
Hyperbilirubinemia	chr6:36680587:T:C	0.00000001	rs3176334-T	alanine aminotransferase levels	T	C	-0.122	0.886	2.31E-01	0.044	1.045	7.6E-01	7.9E-01	1	0.032937	1
Hyperbilirubinemia	chr15:58431280:T:C	2E-10	rs1077834-T	alanine aminotransferase levels	T	C	0.039	1.040	7.16E-01	-0.038	0.963	8.0E-01	7.9E-01	1	0.413605	1
Hyperbilirubinemia	chr18:58416822:T:C	3E-12	rs4503880-T	liver enzyme levels (gamma-glutamyl transferase)	T	C	0.061	1.063	6.11E-01	-0.021	0.980	9.0E-01	7.9E-01	1	0.144494	1
Hyperbilirubinemia	chr1:247438055:G:A	2E-10	rs12143966-G	aspartate aminotransferase levels	G	A	0.043	1.044	6.44E-01	-0.148	0.863	2.8E-01	7.9E-01	1	0.495705	1
Hyperbilirubinemia	chr15:58431740:G:A	1E-11	rs2070895-G	aspartate aminotransferase levels	G	A	0.039	1.040	7.14E-01	-0.038	0.963	8.0E-01	7.9E-01	1	0.413386	1
Hyperbilirubinemia	chr6:33570484:C:T	8E-14	rs9380371-?	aspartate aminotransferase platelet ratio index in high alcohol intake	C	T	0.089	1.093	3.70E-01	-0.189	0.828	2.3E-01	7.9E-01	1	0.258526	1
Hyperbilirubinemia	chr2:31241000:G:C	0.000000002	rs632964-?	aspartate aminotransferase platelet ratio index in high alcohol intake	G	C	0.065	1.068	5.58E-01	0.000	1.000	1.0E+00	8.0E-01	1	0.730016	1

© 2022 Yang W et al. *JAMA Network Open*.

Hyperbilirubinemia	chr6:32433162:G:C	7E-19	rs3129859-C	acute-on-chronic liver failure in hepatitis b	G	C	-0.049	0.952	6.42E-01	0.032	1.033	8.2E-01	8.0E-01	1	0.665197	1
Hyperbilirubinemia	chr5:77438259:C:T	0.00000003	rs33204-C	aspartate aminotransferase levels	C	T	-0.021	0.979	8.21E-01	-0.076	0.927	5.7E-01	8.0E-01	1	0.903272	1
Hyperbilirubinemia	chr1:247439666:A:G	2E-14	rs4925546-A	alanine aminotransferase levels	A	G	0.065	1.067	5.02E-01	-0.010	0.990	9.4E-01	8.0E-01	1	0.65634	1
Hyperbilirubinemia	chr10:94849995:C:T	0.000000001	R410C	CPIC:CYP2C19	C	T	-12.471	0.000	9.79E-01	-11.705	0.000	9.8E-01	8.0E-01	1	NA	NA

Hyperbilirubinemia	chr19:19296909:T:C	1E-18	rs10401969-?	alanine transaminase levels in high alcohol intake;aspartate transaminase levels in high alcohol intake;aspartate aminotransferase platelet ratio index in high alcohol intake	T	C	-0.092	0.912	5.97E-01	0.078	1.081	7.3E-01	8.0E-01	1	0.339234	1
Hyperbilirubinemia	chr19:806256:A:G	0.00000003	rs351978-G	alanine aminotransferase levels	A	G	0.027	1.027	7.80E-01	-0.007	0.993	9.6E-01	8.0E-01	1	0.848403	1
Hyperbilirubinemia	chr3:171014810:G:A	0.000000002	rs7356034-A	alanine aminotransferase levels	G	A	0.025	1.025	8.10E-01	0.019	1.019	8.9E-01	8.0E-01	1	0.85338	1
Hyperbilirubinemia	chr12:20864941:T:C	7E-19	rs2417940-?	bilirubin levels	T	C	0.138	1.148	3.20E-01	0.237	1.268	2.2E-01	8.0E-01	1	0.230068	1
Hyperbilirubinemia	chr1:97699535:T:C	0.000000001	p.M166V	CPIC:DPYD	T	C	0.135	1.144	4.40E-01	-0.083	0.921	7.5E-01	8.0E-01	1	0.734537	1
Hyperbilirubinemia	chr10:94780653:G:A	0.000000001	W212X	CPIC:CYP2C19	G	A	0.096	1.101	9.19E-01	0.112	1.119	8.9E-01	8.1E-01	1	NA	NA
Hyperbilirubinemia	chr3:136207780:G:T	3E-29	rs645040-T	alanine aminotransferase levels;alanine transaminase levels	G	T	-0.010	0.990	9.33E-01	0.018	1.018	9.1E-01	8.1E-01	1	0.951207	1
Hyperbilirubinemia	chr22:42127941:G:A	0.000000001	p.R296C	CPIC:CYP2D6	G	A	0.046	1.048	6.41E-01	-0.139	0.870	3.3E-01	8.1E-01	1	0.548617	1
Hyperbilirubinemia	chr17:43058379:A:C	1E-12	rs8176279-C	aspartate aminotransferase levels	A	C	-0.005	0.995	9.63E-01	-0.024	0.976	8.6E-01	8.1E-01	1	0.309112	1

© 2022 Yang W et al. *JAMA Network Open*.

Hyperbilirubinemia	chr19:48722509:A:T	3E-14	rs11882796-T	aspartate aminotransferase levels	A	T	0.162	1.176	1.03E-01	-0.098	0.907	4.9E-01	8.1E-01	1	0.180653	1
Hyperbilirubinemia	chr19:44911194:T:C	1E-10	rs439401-C	aspartate aminotransferase levels	T	C	0.018	1.019	8.53E-01	-0.100	0.905	4.7E-01	8.1E-01	1	0.042822	1
Hyperbilirubinemia	chr1:201047168:G:A	1E-10	rs3850625-G	aspartate aminotransferase levels	G	A	-0.120	0.887	4.53E-01	0.155	1.167	4.6E-01	8.1E-01	1	0.64093	1
Hyperbilirubinemia	chr12:111286895:T:C	2E-18	rs4766568-T	aspartate aminotransferase levels	T	C	-0.142	0.868	2.79E-01	0.214	1.239	1.9E-01	8.1E-01	1	0.455978	1
Hyperbilirubinemia	chr7:17872129:G:T	4E-25	rs1917368-G	aspartate aminotransferase levels	G	T	-0.117	0.890	2.23E-01	0.195	1.215	1.5E-01	8.2E-01	1	0.965105	1
Hyperbilirubinemia	chr10:17833858:T:G	6E-49	rs56278466-?	aspartate transaminase levels in high alcohol intake;aspartate aminotransferase platelet ratio index in high alcohol intake	T	G	-0.057	0.944	5.57E-01	0.117	1.124	4.1E-01	8.2E-01	1	0.169549	1
Hyperbilirubinemia	chr15:49544670:G:T	0.000000009	rs12593917-T	aspartate aminotransferase levels	G	T	-0.005	0.995	9.65E-01	-0.029	0.972	8.5E-01	8.2E-01	1	0.683617	1
Hyperbilirubinemia	chr10:100080295:T:C	0.00000003	rs7900372-?	alanine transaminase levels in high alcohol intake	T	C	-0.040	0.961	6.83E-01	0.109	1.116	4.3E-01	8.2E-01	1	0.425299	1
Hyperbilirubinemia	chr10:94775416:T:C	0.000000001	W120R	CPIC:CYP2C19	T	C	-12.855	0.000	9.71E-01	1.176	3.241	3.3E-01	8.3E-01	1	NA	NA
Hyperbilirubinemia	chr12:70719307:T:C	4E-27	rs2137537-T	aspartate aminotransferase levels	T	C	0.050	1.051	5.99E-01	-0.053	0.949	6.9E-01	8.3E-01	1	0.744856	1
Hyperbilirubinemia	chr6:115996281:T:C	0.000000002	rs3798232-C	aspartate aminotransferase levels	T	C	0.033	1.033	7.33E-01	-0.086	0.917	5.3E-01	8.3E-01	1	0.333889	1

© 2022 Yang W et al. *JAMA Network Open*.

Hyperbilirubinemia	chr3:58394738:A:T	4E-40	rs11714574-A	aspartate aminotransferase levels	A	T	-0.085	0.919	3.73E-01	-0.255	0.775	6.1E-02	8.3E-01	1	0.181526	1
Hyperbilirubinemia	chr15:72211718:G:A	0.000000004	rs8023703-A	aspartate aminotransferase levels	G	A	-0.002	0.998	9.82E-01	-0.081	0.922	5.6E-01	8.3E-01	1	0.006209	0.882051
Hyperbilirubinemia	chr19:19308262:G:A	1E-18	rs739846-?	alanine transaminase levels in high alcohol intake;aspartate transaminase levels in high alcohol intake;aspartate aminotransferase platelet ratio index in high alcohol intake	G	A	-0.092	0.912	5.97E-01	0.068	1.070	7.6E-01	8.3E-01	1	0.344428	1
Hyperbilirubinemia	chr5:73099362:G:A	8E-10	rs628401-?	aspartate aminotransferase levels	G	A	0.111	1.117	2.41E-01	-0.227	0.797	9.3E-02	8.3E-01	1	0.645994	1
Hyperbilirubinemia	chr17:78406035:G:A	7E-13	rs2376584-A	aspartate aminotransferase levels	G	A	-0.040	0.961	6.70E-01	-0.046	0.955	7.5E-01	8.3E-01	1	0.692588	1
Hyperbilirubinemia	chr22:42126914:C:T	0.000000001	p.E418K	CPIC:CYP2D6	C	T	-12.527	0.000	9.80E-01	-11.296	0.000	9.8E-01	8.3E-01	1	NA	NA
Hyperbilirubinemia	chr22:42129087:G:C	0.000000001	p.Q151E	CPIC:CYP2D6	G	C	-0.431	0.650	6.13E-01	-0.604	0.547	5.9E-01	8.3E-01	1	NA	NA
Hyperbilirubinemia	chr22:42130773:C:T	0.000000001	p.V7M	CPIC:CYP2D6	C	T	-0.431	0.650	6.13E-01	-0.604	0.547	5.9E-01	8.3E-01	1	NA	NA
Hyperbilirubinemia	chr19:19434290:G:A	0.00000002	rs4808199-A	nonalcoholic fatty liver disease	G	A	-0.013	0.987	9.04E-01	0.045	1.046	7.7E-01	8.3E-01	1	0.672225	1
Hyperbilirubinemia	chr2:31251621:A:C	7E-10	rs609316-?	aspartate aminotransferase platelet ratio index in high alcohol intake	A	C	0.077	1.080	4.71E-01	-0.077	0.926	6.2E-01	8.3E-01	1	0.539935	1
Hyperbilirubinemia	chr1:97721542:T:C	0.000000001	p.N151D	CPIC:DPYD	T	C	0.137	1.147	8.76E-01	-0.705	0.494	5.6E-01	8.3E-01	1	NA	NA
Hyperbilirubinemia	chr2:168854097:G:A	5E-18	rs11678685-A	aspartate aminotransferase levels	G	A	0.058	1.060	5.82E-01	-0.041	0.960	7.9E-01	8.4E-01	1	0.760598	1

© 2022 Yang W et al. *JAMA Network Open*.

Hyperbilirubinemia	chr16:72120610:C:T	3E-16	rs4788460-T	alanine aminotransferase levels	C	T	0.083	1.086	4.59E-01	-0.139	0.871	3.9E-01	8.4E-01	1	0.133628	1
Hyperbilirubinemia	chr1:97549713:G:A	0.000000001	p.N457N	CPIC:DPYD	G	A	-0.020	0.981	9.87E-01	-13.686	0.000	9.8E-01	8.4E-01	1	NA	NA
Hyperbilirubinemia	chr12:89147879:G:C	0.00000001	rs10858828-G	aspartate aminotransferase levels	G	C	-0.133	0.875	2.46E-01	0.198	1.219	1.8E-01	8.4E-01	1	0.962914	1
Hyperbilirubinemia	chr16:72072066:T:C	7E-17	rs217184-T	aspartate aminotransferase levels	T	C	-0.133	0.875	2.28E-01	0.082	1.085	5.8E-01	8.5E-01	1	0.16253	1
Hyperbilirubinemia	chr5:107543091:G:C	6E-11	rs6879279-G	alanine aminotransferase levels	G	C	0.032	1.032	8.10E-01	0.138	1.148	4.4E-01	8.5E-01	1	0.112991	1
Hyperbilirubinemia	chr4:48041928:G:A	0.00000001	rs13145218-A	aspartate aminotransferase levels	G	A	0.018	1.018	8.78E-01	0.037	1.038	8.2E-01	8.5E-01	1	0.856149	1
Hyperbilirubinemia	chr1:111141654:A:G	0.000000007	rs1335645-A	liver enzyme levels (gamma-glutamyl transferase)	A	G	0.100	1.105	4.29E-01	0.099	1.104	6.2E-01	8.5E-01	1	0.43342	1
Hyperbilirubinemia	chr6:117180825:T:C	4E-14	rs9372475-T	aspartate aminotransferase levels	T	C	-0.106	0.899	2.92E-01	-0.021	0.979	8.8E-01	8.5E-01	1	0.336717	1
Hyperbilirubinemia	chrX:154535277:T:C	0.000000001	p.N126D	CPIC:G6PD	T	C	-0.367	0.693	3.23E-01	0.477	1.611	6.3E-02	8.5E-01	1	0.035246	1
Hyperbilirubinemia	chr2:233800178:T:G	0.00000005	rs55686299-G	bilirubin levels	T	G	-0.079	0.924	5.49E-01	-0.126	0.882	5.0E-01	8.5E-01	1	NA	NA
Hyperbilirubinemia	chr10:73021160:A:G	0.000000002	rs79188145-A	alanine aminotransferase levels	A	G	-0.139	0.870	4.00E-01	0.028	1.028	8.9E-01	8.5E-01	1	0.00404	0.774359
Hyperbilirubinemia	chr10:94989020:C:T	0.000000001	p.P489S	CPIC:CYP2C9	C	T	-0.301	0.740	7.92E-01	-13.284	0.000	9.8E-01	8.6E-01	1	NA	NA
Hyperbilirubinemia	chr12:21215788:T:C	0.00000005	rs4363657-?	bilirubin levels	T	C	0.221	1.247	6.55E-02	0.006	1.006	9.7E-01	8.6E-01	1	NA	NA
Hyperbilirubinemia	chrX:154532439:A:G	0.000000001	p.Y437Y	CPIC:G6PD	A	G	-0.162	0.850	1.09E-01	0.162	1.176	3.0E-01	8.6E-01	1	0.705099	1

© 2022 Yang W et al. *JAMA Network Open*.

Hyperbilirubinemia	chr8:125469835:A:G	2E-75	rs2954021-A	alanine aminotransferase levels;liver enzyme levels (alkaline phosphatase);liver enzyme levels (alanine transaminase)	A	G	0.016	1.016	8.66E-01	0.017	1.017	9.0E-01	8.6E-01	1	0.439662	1
Hyperbilirubinemia	chr10:94775106:C:T	0.000000001	R73C	CPIC:CYP2C19	C	T	-11.933	0.000	9.73E-01	-11.354	0.000	9.7E-01	8.6E-01	1	NA	NA
Hyperbilirubinemia	chr22:43932850:G:A	3E-10	rs4823173-?	aspartate aminotransferase levels;alanine aminotransferase levels	G	A	0.185	1.203	8.50E-02	-0.163	0.850	3.4E-01	8.6E-01	1	0.485009	1
Hyperbilirubinemia	chr10:100152437:C:T	1E-16	rs1408579-?	alanine aminotransferase levels	C	T	0.101	1.106	3.18E-01	-0.149	0.862	3.1E-01	8.6E-01	1	0.401457	1
Hyperbilirubinemia	chr4:102267552:C:T	1E-133	rs13107325-T	liver fibrosis and steatohepatitis severity (mri ct1 measure)	C	T	-0.185	0.831	3.49E-01	0.118	1.125	6.9E-01	8.6E-01	1	0.388865	1
Hyperbilirubinemia	chr19:33398687:A:G	5E-16	rs7256564-A	alanine aminotransferase levels	A	G	0.117	1.124	2.43E-01	-0.100	0.904	4.7E-01	8.7E-01	1	0.984941	1

© 2022 Yang W et al. *JAMA Network Open*.

Hyperbilirubinemia	chr10:100152307:T:C	3E-114	rs2862954-T	alanine aminotransferase levels;aspartate aminotransferase levels	T	C	0.101	1.106	3.18E-01	-0.151	0.860	3.1E-01	8.7E-01	1	0.401056	1
Hyperbilirubinemia	chr22:42128945:C:T	0.000000001	Splicing defect	CPIC:CYP2D6	C	T	0.017	1.017	8.99E-01	-0.109	0.896	5.9E-01	8.8E-01	1	0.167577	1
Hyperbilirubinemia	chr5:96898508:G:A	8E-12	rs3096168-G	aspartate aminotransferase levels	G	A	0.018	1.018	8.51E-01	-0.050	0.951	7.1E-01	8.8E-01	1	0.888535	1
Hyperbilirubinemia	chr2:210675783:C:A	6E-18	rs1047891-?	alanine transaminase levels;alanine aminotransferase levels;protein quantitative trait loci (liver)	C	A	0.026	1.026	7.99E-01	-0.011	0.989	9.4E-01	8.8E-01	1	NA	NA
Hyperbilirubinemia	chr10:96706525:T:A	1E-14	rs61856806-A	alanine aminotransferase levels	T	A	0.083	1.087	5.25E-01	-0.221	0.802	2.5E-01	8.8E-01	1	0.128282	1
Hyperbilirubinemia	chr22:42127852:C:T	0.000000001	Splicing defect	CPIC:CYP2D6	C	T	-1.002	0.367	3.67E-01	0.244	1.276	8.3E-01	8.8E-01	1	NA	NA
Hyperbilirubinemia	chr19:40827379:T:C	7E-20	rs11878604-?	alanine transaminase levels;alanine aminotransferase levels;aspartate aminotransferase levels	T	C	-0.027	0.974	8.48E-01	-0.067	0.936	7.5E-01	8.8E-01	1	0.695483	1
Hyperbilirubinemia	chr17:7188331:T:C	8E-12	rs314253-C	liver enzyme levels (alkaline phosphatase)	T	C	-0.122	0.885	2.16E-01	0.149	1.161	2.7E-01	8.8E-01	1	0.698934	1
Hyperbilirubinemia	chr12:21028292:C:A	3E-34	rs1604542-?	total bilirubin levels	C	A	-0.174	0.840	7.48E-02	0.037	1.037	8.0E-01	8.8E-01	1	0.228873	1
Hyperbilirubinemia	chr19:55312964:T:G	0.00000003	rs7246479-G	aspartate aminotransferase levels	T	G	-0.097	0.907	3.19E-01	-0.025	0.976	8.6E-01	8.9E-01	1	0.580192	1
Hyperbilirubinemia	chr19:7827201:A:G	0.00000005	rs12979658-G	alanine aminotransferase levels	A	G	0.036	1.036	7.38E-01	-0.010	0.990	9.5E-01	8.9E-01	1	NA	NA
Hyperbilirubinemia	chr22:43936690:G:A	8E-16	rs2281135-T	liver enzyme levels	G	A	0.171	1.186	1.09E-01	-0.174	0.840	3.0E-01	8.9E-01	1	0.46736	1

© 2022 Yang W et al. *JAMA Network Open*.

Hyperbilirubinemia	chr7:28139639:C:A	0.000000001	rs13247499-?	alanine transaminase levels	C	A	0.124	1.131	3.59E-01	0.037	1.038	8.5E-01	8.9E-01	1	0.540001	1
Hyperbilirubinemia	chr9:101460951:C:G	0.000000001	rs10819937-C	liver enzyme levels (alkaline phosphatase)	C	G	-0.114	0.892	3.00E-01	-0.115	0.892	4.7E-01	8.9E-01	1	0.442965	1
Hyperbilirubinemia	chr4:145900258:C:A	2E-62	rs4835265-A	alanine aminotransferase levels;aspartate aminotransferase levels;alanine transaminase levels	C	A	-0.019	0.981	8.65E-01	-0.012	0.988	9.5E-01	8.9E-01	1	0.741357	1
Hyperbilirubinemia	chr8:144467535:G:A	0.000000003	rs35968570-?	alanine transaminase levels in high alcohol intake	G	A	-14.853	0.000	9.78E-01	1.047	2.848	1.2E-01	8.9E-01	1	0.498914	1
Hyperbilirubinemia	chr7:87430383:C:T	3E-12	rs31672-T	aspartate aminotransferase levels	C	T	0.094	1.098	4.15E-01	-0.003	0.997	9.9E-01	8.9E-01	1	0.357769	1
Hyperbilirubinemia	chr19:40991381:A:T	0.000000001	T26S	CPIC:CYP2B6	A	T	0.300	1.350	7.98E-01	-12.760	0.000	9.7E-01	9.0E-01	1	NA	NA
Hyperbilirubinemia	chr19:40991388:A:G	0.000000001	D28G	CPIC:CYP2B6	A	G	0.300	1.350	7.98E-01	-12.760	0.000	9.7E-01	9.0E-01	1	NA	NA
Hyperbilirubinemia	chr19:40991390:C:A	0.000000001	p.R29S	CPIC:CYP2B6	C	A	0.300	1.350	7.98E-01	-12.760	0.000	9.7E-01	9.0E-01	1	NA	NA
Hyperbilirubinemia	chr19:40991391:G:C	0.000000001	p.R29P	CPIC:CYP2B6	G	C	0.300	1.350	7.98E-01	-12.760	0.000	9.7E-01	9.0E-01	1	NA	NA
Hyperbilirubinemia	chr2:232639279:G:A	0.000000006	rs10165093-?	aspartate aminotransferase levels	G	A	-0.030	0.970	7.53E-01	-0.122	0.885	3.6E-01	9.0E-01	1	0.078268	1
Hyperbilirubinemia	chr8:22059711:T:C	3E-12	rs73545546-C	alanine aminotransferase levels	T	C	0.115	1.122	3.34E-01	-0.102	0.903	5.7E-01	9.0E-01	1	0.055509	1
Hyperbilirubinemia	chr12:21178665:T:C	0.000000001	L191=	CPIC:SLCO1B1	T	C	-0.063	0.939	5.12E-01	0.017	1.017	9.0E-01	9.0E-01	1	0.591475	1
Hyperbilirubinemia	chr11:94137172:C:T	7E-55	rs7117339-C	alanine aminotransferase levels	C	T	0.009	1.009	9.48E-01	-0.004	0.996	9.8E-01	9.0E-01	1	0.920802	1
Hyperbilirubinemia	chr20:12992873:A:T	2E-14	rs686548-A	alanine aminotransferase levels	A	T	0.058	1.060	5.58E-01	-0.010	0.990	9.4E-01	9.0E-01	1	0.399752	1

© 2022 Yang W et al. *JAMA Network Open*.

Hyperbilirubinemia	chr12:20426458:G:C	0.00000001	rs7488780-G	aspartate aminotransferase levels	G	C	-0.126	0.882	3.00E-01	0.110	1.116	5.2E-01	9.1E-01	1	0.092802	1
Hyperbilirubinemia	chr1:97573863:C:T	0.000000001	p.E412E	CPIC:DPYD	C	T	0.093	1.098	8.15E-01	-0.648	0.523	5.5E-01	9.1E-01	1	0.615175	1
Hyperbilirubinemia	chr1:97579893:G:C	0.000000001	NA	CPIC:DPYD	G	C	0.093	1.098	8.15E-01	-0.648	0.523	5.5E-01	9.1E-01	1	0.615175	1

Hyperbilirubinemia	chr19:35547488:A:G	6E-12	rs7599-A	alanine aminotransferase levels	A	G	0.147	1.158	1.36E-01	0.098	1.103	4.7E-01	9.1E-01	1	0.000915	0.261538
Hyperbilirubinemia	chr22:43937814:T:G	2E-31	rs2896019-G	nonalcoholic fatty liver disease;pediatric non-alcoholic fatty liver disease activity score	T	G	0.166	1.180	1.20E-01	-0.172	0.842	3.0E-01	9.2E-01	1	0.490846	1
Hyperbilirubinemia	chr10:69225511:G:C	8E-27	rs2394529-C	alanine aminotransferase levels;aspartate aminotransferase levels	G	C	-0.150	0.861	1.27E-01	0.109	1.115	4.2E-01	9.2E-01	1	0.200319	1
Hyperbilirubinemia	chr16:58804727:G:A	0.00000002	rs35317979-?	aspartate transaminase levels in high alcohol intake;aspartate aminotransferase platelet ratio index in high alcohol intake	G	A	0.042	1.042	8.37E-01	-0.184	0.832	5.3E-01	9.2E-01	1	0.528426	1
Hyperbilirubinemia	chr14:20470092:A:C	1E-11	rs1760940-C	aspartate aminotransferase levels;alanine aminotransferase levels	A	C	0.133	1.142	2.65E-01	-0.121	0.886	4.8E-01	9.2E-01	1	0.330145	1
Hyperbilirubinemia	chr6:31464003:T:G	6E-97	rs2395029-?	drug-induced liver injury (flucloxacillin)	T	G	-0.020	0.980	9.46E-01	-0.213	0.808	6.1E-01	9.2E-01	1	0.902468	1
Hyperbilirubinemia	chr16:88631079:G:A	1E-10	rs2291160-G	aspartate aminotransferase levels	G	A	0.002	1.002	9.80E-01	-0.071	0.931	6.0E-01	9.2E-01	1	0.2796	1

© 2022 Yang W et al. *JAMA Network Open*.

Hyperbilirubinemia	chr2:112957399:T:G	0.000000006	rs12617864-G	alanine aminotransferase levels	T	G	-0.206	0.814	4.60E-02	0.198	1.218	1.8E-01	9.3E-01	1	0.144537	1
Hyperbilirubinemia	chr22:42127803:C:T	0.000000001	Splicing defect	CPIC:CYP2D6	C	T	0.188	1.207	2.36E-01	-0.256	0.774	3.4E-01	9.3E-01	1	0.036549	1
Hyperbilirubinemia	chr16:58736993:T:G	9E-41	rs11643959-T	aspartate aminotransferase levels	T	G	-0.023	0.977	9.09E-01	-0.074	0.929	7.9E-01	9.3E-01	1	0.536053	1

Hyperbilirubinemia	chr6:54038354:G:A	4E-10	rs9637973-G	alanine aminotransferase levels	G	A	0.032	1.032	7.54E-01	-0.070	0.933	6.3E-01	9.3E-01	1	0.507824	1
Hyperbilirubinemia	chr1:97883329:A:G	0.000000001	p.C29R	CPIC:DPYD	A	G	0.126	1.134	2.59E-01	-0.090	0.914	5.9E-01	9.3E-01	1	0.211131	1
Hyperbilirubinemia	chr19:41307470:T:C	7E-10	rs15052-?	aspartate aminotransferase platelet ratio index in high alcohol intake	T	C	-0.087	0.916	5.56E-01	-0.080	0.923	6.9E-01	9.3E-01	1	0.780276	1
Hyperbilirubinemia	chr17:63844742:G:C	0.000000005	rs2727324-C	alanine aminotransferase levels	G	C	0.050	1.051	6.05E-01	-0.087	0.917	5.2E-01	9.3E-01	1	0.759116	1
Hyperbilirubinemia	chr6:35312920:C:T	5E-17	rs6938946-C	aspartate aminotransferase levels	C	T	0.010	1.010	9.36E-01	-0.014	0.986	9.3E-01	9.3E-01	1	0.600122	1
Hyperbilirubinemia	chr13:48045806:G:A	0.000000001	3' UTR	CPIC:NUDT15	G	A	-0.165	0.848	3.43E-01	0.343	1.409	1.5E-01	9.3E-01	1	0.128675	1
Hyperbilirubinemia	chr19:7766742:G:A	2E-21	rs2277998-G	aspartate aminotransferase levels	G	A	0.036	1.037	7.22E-01	-0.153	0.858	3.2E-01	9.3E-01	1	0.193426	1
Hyperbilirubinemia	chr3:136301699:T:C	2E-16	rs1711171-?	total bilirubin levels	T	C	-0.059	0.943	6.01E-01	0.081	1.084	6.2E-01	9.3E-01	1	0.616107	1
Hyperbilirubinemia	chr10:94942290:C:T	0.000000001	p.R144C	CPIC:CYP2C9	C	T	0.026	1.026	8.71E-01	-0.359	0.699	1.7E-01	9.3E-01	1	0.852537	1
Hyperbilirubinemia	chr7:55869748:T:A	0.000000004	rs7803882-?	alanine transaminase levels	T	A	-0.082	0.921	3.81E-01	0.026	1.026	8.5E-01	9.4E-01	1	0.202578	1
Hyperbilirubinemia	chr1:97515839:T:C	0.000000001	p.I543V	CPIC:DPYD	T	C	-0.132	0.877	2.63E-01	0.259	1.295	8.4E-02	9.4E-01	1	0.887914	1
Hyperbilirubinemia	chr3:142940519:A:AT	3E-11	rs6440123-A	alanine aminotransferase levels	A	AT	-12.324	0.000	9.81E-01	-11.953	0.000	9.7E-01	9.4E-01	1	1	1
Hyperbilirubinemia	chr12:57445390:G:T	6E-25	rs61352607-T	aspartate aminotransferase levels	G	T	0.050	1.051	6.39E-01	0.057	1.058	7.3E-01	9.4E-01	1	0.084562	1

© 2022 Yang W et al. *JAMA Network Open*.

Hyperbilirubinemia	chr1:59214574:T:G	2E-10	rs6678642-T	aspartate aminotransferase levels	T	G	0.091	1.095	3.92E-01	-0.160	0.852	3.0E-01	9.4E-01	1	0.937302	1
Hyperbilirubinemia	chr4:78698045:C:A	0.00000002	rs75759936-?	aspartate aminotransferase levels	C	A	0.287	1.332	4.00E-01	0.115	1.121	8.9E-01	9.4E-01	1	0.32917	1

© 2022 Yang W et al. *JAMA Network Open*.

Hyperbilirubinemia	chr4:87265357:G:A	2E-16	rs7694379-?	alanine transaminase levels in high alcohol intake;protein quantitative trait loci (liver);aspartate aminotransferase platelet ratio index in high alcohol intake;aspartate transaminase levels in high alcohol intake	G	A	-0.066	0.936	5.17E-01	0.046	1.047	7.4E-01	9.4E-01	1	NA	NA
Hyperbilirubinemia	chr3:170355551:A:C	0.000000001	rs12489967-C	aspartate aminotransferase levels	A	C	-0.009	0.992	9.42E-01	0.124	1.131	4.8E-01	9.4E-01	1	0.212713	1
Hyperbilirubinemia	chr19:41016810:C:T	0.000000001	R487C; R487S	CPIC:CYP2B6	C	T	0.092	1.096	5.80E-01	0.018	1.019	9.3E-01	9.4E-01	1	0.979003	1
Hyperbilirubinemia	chr9:109315250:T:C	1E-10	rs68082079-T	alanine aminotransferase levels	T	C	0.244	1.277	2.09E-02	-0.189	0.828	2.1E-01	9.5E-01	1	0.007073	0.938462
Hyperbilirubinemia	chr4:145893489:G:A	8E-13	rs7682289-?	alanine aminotransferase levels	G	A	-0.015	0.985	8.93E-01	0.006	1.006	9.7E-01	9.5E-01	1	0.881391	1
Hyperbilirubinemia	chr11:94131227:C:G	9E-38	rs56175344-C	aspartate aminotransferase levels	C	G	0.017	1.017	9.07E-01	-0.032	0.969	8.7E-01	9.5E-01	1	0.946306	1
Hyperbilirubinemia	chr3:186933001:A:G	0.00000001	rs10937275-?	drug-induced liver injury (flucloxacillin)	A	G	-0.209	0.811	1.79E-01	0.299	1.349	2.2E-01	9.5E-01	1	0.975897	1
Hyperbilirubinemia	chr3:149470375:A:G	7E-23	rs78900599-A	alanine aminotransferase levels	A	G	-0.121	0.886	4.96E-01	-0.234	0.792	3.6E-01	9.5E-01	1	0.774563	1
Hyperbilirubinemia	chr6:32606583:C:T	1E-13	rs2647074-?	aspartate aminotransferase levels;total bilirubin levels	C	T	-0.016	0.984	8.76E-01	0.022	1.023	8.8E-01	9.5E-01	1	0.092326	1
Hyperbilirubinemia	chr5:179863845:T:G	0.00000004	rs30386-G	aspartate aminotransferase levels	T	G	0.135	1.145	1.73E-01	-0.009	0.991	9.5E-01	9.6E-01	1	0.339289	1

© 2022 Yang W et al. *JAMA Network Open*.

Hyperbilirubinemia	chr1:93392732:G:A	2E-10	rs1365298-A	alanine aminotransferase levels	G	A	-0.148	0.863	1.84E-01	0.045	1.046	7.7E-01	9.6E-01	1	0.323741	1
Hyperbilirubinemia	chr19:10222312:C:T	1E-16	rs10409243-C	alanine aminotransferase levels	C	T	0.058	1.060	5.55E-01	-0.058	0.944	6.8E-01	9.6E-01	1	0.231662	1
Hyperbilirubinemia	chr16:53772541:A:G	0.000000002	rs56094641-G	alanine aminotransferase levels	A	G	-0.129	0.879	2.27E-01	0.089	1.093	5.4E-01	9.6E-01	1	0.185036	1
Hyperbilirubinemia	chr3:142940519:A:G	3E-11	rs6440123-A	alanine aminotransferase levels	A	G	-0.007	0.993	9.49E-01	-0.095	0.910	5.3E-01	9.7E-01	1	0.423487	1
Hyperbilirubinemia	chr1:26700496:C:G	0.00000003	rs4579782-G	alanine aminotransferase levels	C	G	-0.078	0.925	7.15E-01	-0.188	0.829	5.4E-01	9.7E-01	1	0.605767	1
Hyperbilirubinemia	chr10:114026477:G:A	0.000000004	rs72823014-G	aspartate aminotransferase levels	G	A	0.112	1.118	4.66E-01	0.049	1.051	8.1E-01	9.7E-01	1	0.248192	1
Hyperbilirubinemia	chr19:40991369:C:T	0.000000001	R22C	CPIC:CYP2B6	C	T	0.024	1.024	9.13E-01	0.346	1.414	2.6E-01	9.7E-01	1	0.87712	1
Hyperbilirubinemia	chr6:54059899:T:C	0.000000003	rs9296736-T	liver enzyme levels (gamma-glutamyl transferase)	T	C	0.021	1.021	8.37E-01	-0.065	0.937	6.5E-01	9.7E-01	1	0.487656	1
Hyperbilirubinemia	chr1:97699474:T:C	0.000000001	p.Y186C	CPIC:DPYD	T	C	-12.123	0.000	9.81E-01	-11.646	0.000	9.8E-01	9.7E-01	1	NA	NA
Hyperbilirubinemia	chr9:104903697:C:G	0.000000003	rs1800978-C	aspartate aminotransferase levels	C	G	0.009	1.009	9.44E-01	0.159	1.173	3.6E-01	9.7E-01	1	0.358072	1
Hyperbilirubinemia	chr2:31254972:G:A	0.000000002	rs655029-?	aspartate aminotransferase platelet ratio index in high alcohol intake	G	A	0.018	1.018	8.70E-01	-0.059	0.942	7.2E-01	9.8E-01	1	0.534942	1
Hyperbilirubinemia	chr6:33567103:C:T	6E-14	rs6914422-?	aspartate aminotransferase platelet ratio index in high alcohol intake	C	T	0.083	1.087	3.99E-01	-0.110	0.896	4.8E-01	9.8E-01	1	0.342291	1

© 2022 Yang W et al. *JAMA Network Open*.

Hyperbilirubinemia	chr10:96700824:G:A	2E-11	rs45587331-?	alanine aminotransferase levels;aspartate aminotransferase levels;alanine transaminase levels	G	A	0.088	1.092	4.86E-01	-0.252	0.778	1.8E-01	9.8E-01	1	0.154269	1
Hyperbilirubinemia	chr12:20904347:C:T	7E-13	rs7953767-T	alanine aminotransferase levels	C	T	0.116	1.123	3.74E-01	0.262	1.299	1.2E-01	9.8E-01	1	0.060383	1
Hyperbilirubinemia	chr2:48047453:A:T	5E-12	rs72816437-T	aspartate aminotransferase levels	A	T	0.001	1.001	9.92E-01	-0.110	0.896	4.4E-01	9.8E-01	1	0.800093	1
Hyperbilirubinemia	chr22:42130761:C:T	0.000000001	p.V11M	CPIC:CYP2D6	C	T	-0.069	0.933	7.55E-01	-0.185	0.831	6.2E-01	9.8E-01	1	0.801381	1
Hyperbilirubinemia	chr19:10236408:T:C	2E-10	rs8108722-?	alanine aminotransferase levels;alanine transaminase levels	T	C	0.031	1.031	7.88E-01	-0.167	0.846	2.9E-01	9.8E-01	1	0.188298	1
Hyperbilirubinemia	chr8:60613221:G:A	9E-16	rs671275-A	aspartate aminotransferase levels	G	A	0.076	1.079	4.81E-01	-0.166	0.847	2.7E-01	9.9E-01	1	0.280142	1
Hyperbilirubinemia	chr1:65675819:G:T	9E-13	rs1938500-T	aspartate aminotransferase levels;alanine aminotransferase levels	G	T	0.006	1.006	9.55E-01	-0.060	0.942	7.1E-01	9.9E-01	1	0.176682	1
Hyperbilirubinemia	chr10:17974944:C:T	4E-159	rs508196-C	aspartate aminotransferase levels	C	T	0.110	1.116	2.48E-01	-0.064	0.938	6.4E-01	9.9E-01	1	0.04292	1
Hyperbilirubinemia	chr22:42129770:G:A	0.000000001	p.T107I; p.T10	CPIC:CYP2D6	G	A	-0.195	0.823	6.70E-01	0.383	1.467	4.0E-01	9.9E-01	1	0.596026	1
Hyperbilirubinemia	chr14:50734993:C:T	6E-12	rs10148309-C	alanine aminotransferase levels	C	T	0.060	1.062	6.04E-01	0.187	1.206	2.4E-01	9.9E-01	1	0.032874	1
Hyperbilirubinemia	chr2:112027660:T:C	9E-10	rs6541998-T	aspartate aminotransferase levels	T	C	0.058	1.059	5.54E-01	-0.094	0.910	4.9E-01	9.9E-01	1	0.419684	1

© 2022 Yang W et al. *JAMA Network Open*.

Hyperbilirubinemia	chr9:133278724:C:T	3E-123	rs579459-T	liver enzyme levels (alkaline phosphatase)	C	T	0.017	1.017	8.91E-01	0.063	1.065	7.1E-01	1.0E+00	1	0.256651	1
Hyperbilirubinemia	chr19:3750624:A:C	8E-10	rs1046271-A	alanine aminotransferase levels;aspartate aminotransferase levels	A	C	-0.043	0.958	6.51E-01	0.018	1.019	8.9E-01	1.0E+00	1	0.613134	1
Hyperbilirubinemia	chr3:50080087:A:G	0.000000007	rs2624817-A	alanine aminotransferase levels	A	G	-0.129	0.879	2.98E-01	0.237	1.267	2.5E-01	1.0E+00	1	0.74397	1
Hyperbilirubinemia	chr1:26811902:C:T	0.00000001	rs12748152-C	alanine aminotransferase (alt) levels after remission induction therapy in actute lymphoblastic leukemia (all)	C	T	-0.076	0.927	7.22E-01	-0.194	0.824	5.2E-01	1.0E+00	1	0.610575	1
Hyperbilirubinemia	chr7:135199435:C:T	2E-10	rs292585-T	alanine aminotransferase levels	C	T	0.008	1.008	9.39E-01	0.029	1.029	8.4E-01	1.0E+00	1	0.398495	1
Hyperbilirubinemia	chr4:87063179:A:G	0.00000004	rs28432336-?	total bilirubin levels	A	G	0.000	1.000	9.96E-01	0.171	1.186	2.1E-01	1.0E+00	1	0.940319	1
Hyperbilirubinemia	chr22:42129183:C:T	0.000000001	p.V119M	CPIC:CYP2D6	C	T	3.091	21.997	7.83E-02	-9.841	0.000	9.8E-01	NA	NA	NA	NA
Hyperbilirubinemia	chr1:97305363:A:C	0.000000001	p.V732G	CPIC:DPYD	A	C	2.066	7.896	1.67E-01	-8.677	0.000	9.8E-01	NA	NA	NA	NA
Hyperbilirubinemia	chr10:94972119:C:A	0.000000001	p.P279T	CPIC:CYP2C9	C	A	1.185	3.270	4.12E-01	-9.526	0.000	9.8E-01	NA	NA	NA	NA
Hyperbilirubinemia	chr7:117530975:G:A	0.000000001	R117H	CPIC:CFTR	G	A	-0.248	0.781	7.74E-01	-9.939	0.000	9.8E-01	NA	NA	NA	NA
Hyperbilirubinemia	chrX:154532945:C:G	0.000000001	p.D350H	CPIC:G6PD	C	G	-11.368	0.000	9.77E-01	-4.341	0.013	9.8E-01	NA	NA	NA	NA
Hyperbilirubinemia	chr19:39352092:C:T	2E-12	rs773561743-	alanine aminotransferase levels	C	T	-10.513	0.000	9.78E-01	-9.660	0.000	9.8E-01	NA	NA	NA	NA
Hyperbilirubinemia	chrX:154533025:A:G	0.000000001	p.L323P	CPIC:G6PD	A	G	-11.154	0.000	9.78E-01	-4.615	0.010	9.8E-01	NA	NA	NA	NA
Hyperbilirubinemia	chr1:65675819:G:GT	9E-13	rs1938500-T	aspartate aminotransferase levels;alanine aminotransferase levels	G	GT	-10.047	0.000	9.79E-01	-9.509	0.000	9.8E-01	NA	NA	NA	NA

© 2022 Yang W et al. *JAMA Network Open*.

Hyperbilirubinemia	chr7:117587806:G:A	0.000000001	G551D	CPIC:CFTR	G	A	-9.888	0.000	9.79E-01	15.826	NA	9.6E-01	NA	NA	NA	NA
Hyperbilirubinemia	chr10:94780574:G:C	0.000000001	R186P	CPIC:CYP2C19	G	C	-10.731	0.000	9.82E-01	-8.565	0.000	9.8E-01	NA	NA	NA	NA
Hyperbilirubinemia	chr22:42127611:C:T	0.000000001	p.D337N	CPIC:CYP2D6	C	T	-10.576	0.000	9.84E-01	-9.380	0.000	9.8E-01	NA	NA	NA	NA
Hyperbilirubinemia	chr1:97450168:A:G	0.000000001	p.M599T	CPIC:DPYD	A	G	#N/A	#N/A	#N/A	#N/A	#N/A	#N/A	#N/A	NA	NA	NA
Hyperbilirubinemia	chr1:97549735:G:A	0.000000001	p.A450V	CPIC:DPYD	G	A	#N/A	#N/A	#N/A	#N/A	#N/A	#N/A	#N/A	NA	NA	NA

Hyperbilirubinemia	chr10:94780574:G:A	0.000000001	R186P	CPIC:CYP2C19	G	A	#N/A	#N/A	#N/A	#N/A	#N/A	#N/A	#N/A	NA	NA	NA
Hyperbilirubinemia	chr10:94780579:G:A	0.000000001	D188N	CPIC:CYP2C19	G	A	#N/A	#N/A	#N/A	#N/A	#N/A	#N/A	#N/A	NA	NA	NA
Hyperbilirubinemia	chr10:94781858:C:T	0.000000001	P227L	CPIC:CYP2C19	C	T	#N/A	#N/A	#N/A	#N/A	#N/A	#N/A	#N/A	NA	NA	NA
Hyperbilirubinemia	chr10:94852765:C:T	0.000000001	R442C	CPIC:CYP2C19	C	T	#N/A	#N/A	#N/A	#N/A	#N/A	#N/A	#N/A	NA	NA	NA
Hyperbilirubinemia	chr10:94938771:C:T	0.000000001	p.P30L	CPIC:CYP2C9	C	T	#N/A	#N/A	#N/A	#N/A	#N/A	#N/A	#N/A	NA	NA	NA
Hyperbilirubinemia	chr10:94942231:G:A	0.000000001	p.R124Q	CPIC:CYP2C9	G	A	#N/A	#N/A	#N/A	#N/A	#N/A	#N/A	#N/A	NA	NA	NA
Hyperbilirubinemia	chr10:94981201:T:C	0.000000001	p.I327T	CPIC:CYP2C9	T	C	#N/A	#N/A	#N/A	#N/A	#N/A	#N/A	#N/A	NA	NA	NA
Hyperbilirubinemia	chr12:20845519:T:C	4E-13	rs137858877-	total bilirubin levels	T	C	#N/A	#N/A	#N/A	#N/A	#N/A	#N/A	#N/A	NA	NA	NA
Hyperbilirubinemia	chr19:41010088:C:G	0.000000001	T306S	CPIC:CYP2B6	C	G	#N/A	#N/A	#N/A	#N/A	#N/A	#N/A	#N/A	NA	NA	NA
Hyperbilirubinemia	chr19:46932214:G:A	0.00000002	rs547445211-	aspartate aminotransferase levels	G	A	#N/A	#N/A	#N/A	#N/A	#N/A	#N/A	#N/A	NA	NA	NA
Hyperbilirubinemia	chr22:42126605:G:A	0.000000001	p.S488F	CPIC:CYP2D6	G	A	#N/A	#N/A	#N/A	#N/A	#N/A	#N/A	#N/A	NA	NA	NA
Hyperbilirubinemia	chr22:42126647:C:T	0.000000001	p.R474Q	CPIC:CYP2D6	C	T	#N/A	#N/A	#N/A	#N/A	#N/A	#N/A	#N/A	NA	NA	NA
Hyperbilirubinemia	chr22:42127457:C:G	0.000000001	p.R388H	CPIC:CYP2D6	C	G	#N/A	#N/A	#N/A	#N/A	#N/A	#N/A	#N/A	NA	NA	NA
Hyperbilirubinemia	chr22:42127846:C:T	0.000000001	p.321fs	CPIC:CYP2D6	C	T	#N/A	#N/A	#N/A	#N/A	#N/A	#N/A	#N/A	NA	NA	NA
Hyperbilirubinemia	chr22:42128329:G:A	0.000000001	p.L230F	CPIC:CYP2D6	G	A	#N/A	#N/A	#N/A	#N/A	#N/A	#N/A	#N/A	NA	NA	NA
Hyperbilirubinemia	chr3:142940519:AT:A	3E-11	rs6440123-A	alanine aminotransferase levels	AT	A	#N/A	#N/A	#N/A	#N/A	#N/A	#N/A	#N/A	NA	NA	NA
Hyperbilirubinemia	chr6:18130729:C:T	0.000000001	R226Q	CPIC:TPMT	C	T	#N/A	#N/A	#N/A	#N/A	#N/A	#N/A	#N/A	NA	NA	NA
Hyperbilirubinemia	chr6:18133845:T:A	0.000000001	Y180F	CPIC:TPMT	T	A	#N/A	#N/A	#N/A	#N/A	#N/A	#N/A	#N/A	NA	NA	NA
Hyperbilirubinemia	chr6:18138970:G:A	0.000000001	R163C	CPIC:TPMT	G	A	#N/A	#N/A	#N/A	#N/A	#N/A	#N/A	#N/A	NA	NA	NA
Hyperbilirubinemia	chr6:18147851:G:C	0.000000001	L69V	CPIC:TPMT	G	C	#N/A	#N/A	#N/A	#N/A	#N/A	#N/A	#N/A	NA	NA	NA
Hyperbilirubinemia	chr7:117530974:C:G	0.000000001	R117C	CPIC:CFTR	C	G	#N/A	#N/A	#N/A	#N/A	#N/A	#N/A	#N/A	NA	NA	NA
Hyperbilirubinemia	chr7:117548795:C:T	0.000000001	A455E	CPIC:CFTR	C	T	#N/A	#N/A	#N/A	#N/A	#N/A	#N/A	#N/A	NA	NA	NA
Hyperbilirubinemia	chr7:117611595:T:G	0.000000001	F1052V	CPIC:CFTR	T	G	#N/A	#N/A	#N/A	#N/A	#N/A	#N/A	#N/A	NA	NA	NA
Hyperbilirubinemia	chrX:154533122:C:T	0.000000001	p.V291M	CPIC:G6PD	C	T	#N/A	#N/A	#N/A	#N/A	#N/A	#N/A	#N/A	NA	NA	NA

© 2022 Yang W et al. *JAMA Network Open*.

ALT/AST	chr22:43928850:C:T	8E-82	rs738408-?	alanine transaminase levels in high alcohol intake;aspartate transaminase levels in high alcohol intake;aspartate aminotransferase levels;aspartate aminotransferase platelet ratio index in high alcohol intake;alanine aminotransferase (alt) levels after remission induction therapy in acute lymphoblastic leukemia (all)	C	T	0.228	1.257	0.00012	0.253	1.288	0.00677	3.7248E-07	0	0.38749	1

© 2022 Yang W et al. *JAMA Network Open*.

ALT/AST	chr22:43928847:C:G	1E-300	rs738409-G	aminotransferase levels;alanine aminotransferase levels;alanine transaminase levels in high alcohol intake;aspartate transaminase levels in high alcohol intake;liver fat content (mri proton density fat fraction measure);aspartate aminotransferase platelet ratio index in high alcohol intake;alanine transaminase levels;nonalcoholic fatty liver disease;liver enzyme levels (alanine transaminase);liver fibrosis and steatohepatitis severity (mri ct1 measure);percent liver fat;alanine aminotransferase (alt)	C	G	0.228	1.257	0.00012	0.253	1.288	0.00677	3.7248E-07	0	0.38749	1

© 2022 Yang W et al. *JAMA Network Open*.

ALT/AST	chr22:43928975:G:A	4E-82	rs3747207-?	alanine transaminase levels in high alcohol intake;aspartate transaminase levels in high alcohol intake;aspartate aminotransferase platelet ratio index in high alcohol intake;alanine aminotransferase levels in excessive alcohol consumption;aspartate aminotransferase levels in excessive alcohol consumption	G	A	0.236	1.266	0.000359923	0.238	1.269	0.0170886	3.82E-07	0	0.35964	1
ALT/AST	chr4:87292656:A:G	5E-69	rs6834314-A	alanine aminotransferase levels;aspartate aminotransferase levels;liver enzyme levels (alanine transaminase)	A	G	-0.138	0.871	0.051723436	-0.284	0.752	0.0071238	0.00028938	0.19	0.00782	0.964103
ALT/AST	chr4:87265357:G:A	2E-16	rs7694379-?	alanine transaminase levels in high alcohol intake;protein quantitative trait loci (liver);aspartate aminotransferase platelet ratio index in high alcohol intake;aspartate transaminase levels in high alcohol intake	G	A	-0.130	0.878	0.039961468	-0.236	0.790	0.0118598	0.00029745	0.2	0.00254	0.65

© 2022 Yang W et al. *JAMA Network Open*.

ALT/AST	chr3:172541716:C:A	5E-16	rs140822617-	alanine aminotransferase levels	C	A	0.269	1.309	0.00182668	0.105	1.111	0.3929446	0.0005281	0.285	0.73802	1
ALT/AST	chr22:43932850:G:A	3E-10	rs4823173-?	aspartate aminotransferase levels;alanine aminotransferase levels	G	A	0.167	1.181	0.018031261	0.138	1.148	0.2027492	0.00091798	0.43	0.30043	1
ALT/AST	chr3:172550013:T:C	2E-20	rs13074711-T	aspartate aminotransferase levels	T	C	0.280	1.323	0.0018262	0.075	1.078	0.5710386	0.0009942	0.49	0.42929	1
ALT/AST	chr1:97305364:C:T	0.000000001	p.V732I	CPIC:DPYD	C	T	0.200	1.221	0.187127298	0.597	1.816	0.0032697	0.00142244	0.59	0.92936	1
ALT/AST	chr3:149470375:A:G	7E-23	rs78900599-A	alanine aminotransferase levels	A	G	0.198	1.219	0.066284059	0.333	1.396	0.0278294	0.00146412	0.61	0.02509	1
ALT/AST	chr4:99144358:T:G	2E-28	rs1800759-T	aspartate aminotransferase levels	T	G	-0.186	0.830	0.002742795	-0.041	0.960	0.6561351	0.00150068	0.64	0.33309	1
ALT/AST	chr22:43937814:T:G	2E-31	rs2896019-G	nonalcoholic fatty liver disease;pediatric non-alcoholic fatty liver disease activity score	T	G	0.154	1.166	0.027570914	0.123	1.130	0.2453219	0.00207922	0.75	0.33228	1
ALT/AST	chr10:112189906:T:C	1E-30	rs10787429-T	alanine aminotransferase levels;aspartate aminotransferase levels;alanine transaminase levels;alanine transaminase levels in high alcohol intake	T	C	-0.157	0.855	0.019222757	-0.152	0.859	0.1315146	0.00210002	0.75	0.25485	1
ALT/AST	chr22:43936690:G:A	8E-16	rs2281135-T	liver enzyme levels	G	A	0.157	1.170	0.024152304	0.113	1.119	0.2944679	0.00231605	0.78	0.30213	1

© 2022 Yang W et al. *JAMA Network Open*.

ALT/AST	chr19:44908684:T:C	5E-35	rs429358-T	alanine aminotransferase levels;liver fat content (mri proton density fat fraction measure)	T	C	-0.144	0.866	0.099050237	-0.270	0.763	0.0334391	0.00284857	0.88	0.72106	1

ALT/AST	chr5:149404204:T:G	0.00000003	rs6887452-G	serum bilirubin levels x mediterranean diet adherence interaction in metabolic syndrome	T	G	0.126	1.135	0.044802219	0.169	1.184	0.0615878	0.00334293	0.915	0.38841	1
ALT/AST	chr6:18130687:T:C	0.000000001	Y240C;Y240S	CPIC:TPMT	T	C	-0.334	0.716	0.0287	-0.400	0.670	0.0509	0.0034	0.155	0.00002	0.020769
ALT/AST	chr10:99372525:A:C	1E-25	rs10509735-?	aspartate aminotransferase levels	A	C	0.172	1.188	0.007178	0.047	1.049	0.6193212	0.00564967	0.97	0.02007	1
ALT/AST	chr4:87255231:T:C	4E-15	rs28664118-?	aspartate aminotransferase levels	T	C	-0.044	0.957	0.553589452	-0.371	0.690	0.0010368	0.00761516	0.99	0.02036	1
ALT/AST	chr4:87254878:C:A	1E-16	rs10001545-?	alanine transaminase levels;aspartate aminotransferase levels	C	A	-0.049	0.953	0.524874617	-0.377	0.686	0.0013308	0.00763876	0.99	0.03096	1
ALT/AST	chr11:119225051:T:G	0.000000007	rs11217192-?	aspartate aminotransferase platelet ratio index in high alcohol intake	T	G	0.168	1.184	0.014444916	0.068	1.070	0.4997384	0.00804923	0.99	0.67640	1
ALT/AST	chr4:87255885:C:A	3E-17	rs11935592-?	alanine aminotransferase levels	C	A	-0.044	0.957	0.555583336	-0.365	0.694	0.0012333	0.00831842	0.99	0.02606	1

© 2022 Yang W et al. *JAMA Network Open*.

ALT/AST	chr1:220796686:A:G	1E-31	rs2642438-G	alanine aminotransferase levels;alanine transaminase levels in high alcohol intake;nonalcoholic fatty liver disease	A	G	0.111	1.117	0.106503811	0.182	1.200	0.0844335	0.00946673	0.99	0.47296	1
ALT/AST	chr2:31241000:G:C	0.000000002	rs632964-?	aspartate aminotransferase platelet ratio index in high alcohol intake	G	C	0.142	1.153	0.044447548	0.119	1.126	0.2721557	0.00995369	0.99	0.69277	1
ALT/AST	chr10:94775106:C:T	0.000000001	R73C	CPIC:CYP2C19	C	T	-13.093	0.000	0.95174941	-12.842	0.000	0.972367	0.00997656	0.99	1.00000	1
ALT/AST	chr19:3750624:A:C	8E-10	rs1046271-A	alanine aminotransferase levels;aspartate aminotransferase levels	A	C	-0.066	0.937	0.267342442	-0.226	0.798	0.0118474	0.01010009	0.995	0.60813	1
ALT/AST	chr18:63181406:T:C	0.00000002	rs4940576-C	aspartate aminotransferase levels	T	C	-0.127	0.881	0.055591467	-0.117	0.890	0.2450852	0.01118463	1	0.83338	1
ALT/AST	chr12:20864941:T:C	7E-19	rs2417940-?	bilirubin levels	T	C	0.126	1.135	0.137467803	0.186	1.205	0.1420365	0.01165623	1	0.99764	1
ALT/AST	chr7:99672916:T:C	0.000000001	Splicing defect	CPIC:CYP3A5	T	C	0.168	1.183	0.064759478	0.177	1.194	0.1726203	0.01208154	1	0.08565	1
ALT/AST	chr2:27512105:G:A	4E-24	rs6547692-G	aspartate aminotransferase levels	G	A	0.101	1.106	0.093050532	0.129	1.138	0.1509699	0.01232111	1	0.74447	1
ALT/AST	chr6:32615700:C:T	7E-38	rs28383223-T	alanine aminotransferase levels	C	T	-0.090	0.914	0.141003343	-0.168	0.845	0.0633696	0.01442617	1	0.58261	1
ALT/AST	chr9:95450326:C:T	0.00000002	rs2282043-C	alanine aminotransferase levels	C	T	-0.201	0.818	0.095634757	-0.218	0.804	0.2230364	0.01536345	1	0.70407	1
ALT/AST	chr1:97515865:C:T	0.000000001	p.S534N	CPIC:DPYD	C	T	-0.591	0.554	0.027028359	-0.194	0.823	0.5848959	0.01758674	1	0.77663	1
ALT/AST	chr6:35312920:C:T	5E-17	rs6938946-C	aspartate aminotransferase levels	C	T	-0.125	0.883	0.109965986	-0.166	0.847	0.1398534	0.01802371	1	0.81500	1

© 2022 Yang W et al. *JAMA Network Open*.

ALT/AST	chr16:68626662:C:T	7E-10	rs6499186-C	liver fibrosis in non-alcoholic fatty acid liver disease	C	T	0.175	1.192	0.015329747	0.037	1.037	0.7403267	0.01821661	1	0.19130	1
ALT/AST	chr2:233790144:T:C	7E-23	rs2361502-?	bilirubin levels;serum bilirubin levels in metabolic syndrome;total bilirubin levels in hiv-1 infection	T	C	0.058	1.060	0.395723894	0.237	1.267	0.0157825	0.01904701	1	0.39378	1
ALT/AST	chr17:63844742:G:C	0.000000005	rs2727324-C	alanine aminotransferase levels	G	C	-0.179	0.836	0.00404479	0.038	1.039	0.6742001	0.02222287	1	0.93596	1
ALT/AST	chr3:58437771:A:C	7E-10	rs6794695-?	aspartate transaminase levels in high alcohol intake;aspartate aminotransferase platelet ratio index in high alcohol intake	A	C	-0.105	0.901	0.195974998	-0.188	0.829	0.1336394	0.02380879	1	0.46104	1
ALT/AST	chr19:44911194:T:C	1E-10	rs439401-C	aspartate aminotransferase levels	T	C	-0.101	0.904	0.108587521	-0.091	0.913	0.3322222	0.02447379	1	0.48571	1
ALT/AST	chr6:32433302:A:G	0.000000001	rs3129860-A	hepatitis c induced liver cirrhosis	A	G	-0.206	0.814	0.025859294	-0.055	0.947	0.6942893	0.02468144	1	0.51502	1
ALT/AST	chr8:80890620:G:A	0.00000002	rs7840329-A	aspartate aminotransferase levels	G	A	0.084	1.087	0.181369099	0.106	1.112	0.2644257	0.02532694	1	0.27988	1
ALT/AST	chr17:75771356:A:G	2E-11	rs4485425-G	alanine aminotransferase levels	A	G	0.125	1.133	0.054113575	0.054	1.056	0.5727312	0.02610028	1	0.51248	1
ALT/AST	chr4:183283075:T:C	0.00000001	rs3814420-T	aspartate aminotransferase levels	T	C	0.152	1.164	0.257839765	0.332	1.394	0.0621666	0.02693724	1	0.92219	1

© 2022 Yang W et al. *JAMA Network Open*.

ALT/AST	chr11:122648314:T:G	1E-11	rs6589941-G	aspartate aminotransferase levels	T	G	-0.096	0.909	0.117400384	-0.093	0.911	0.3111015	0.02730035	1	0.85909	1
ALT/AST	chr1:97305363:A:C	0.000000001	p.V732G	CPIC:DPYD	A	C	2.520	12.427	0.034130923	-9.542	0.000	0.9765603	0.02774071	1	0.01723	1
ALT/AST	chr11:89491285:A:C	0.000000002	rs2289125-C	aspartate aminotransferase levels	A	C	0.104	1.110	0.121761681	0.125	1.134	0.2211554	0.02859841	1	0.97307	1
ALT/AST	chr19:41004381:G:A	0.000000001	R140Q	CPIC:CYP2B6	G	A	-0.871	0.418	0.129987605	-1.412	0.244	0.307858	0.02877756	1	0.60932	1
ALT/AST	chr22:43995806:G:A	2E-11	rs2143571-A	nonalcoholic fatty liver disease	G	A	0.104	1.110	0.135239894	0.098	1.103	0.3561476	0.03316755	1	0.78793	1

© 2022 Yang W et al. *JAMA Network Open*.

ALT/AST	chr2:27508073:T:C	4E-13	rs1260326-T	liver enzyme levels (gamma-glutamyl transferase);nonalcoholic fatty liver disease;liver fat content (mri proton density fat fraction measure)	T	C	0.100	1.105	0.102588077	0.081	1.084	0.3806627	0.03574396	1	0.86176	1
ALT/AST	chr10:94981301:C:G	0.000000001	p.D360E	CPIC:CYP2C9	C	G	-12.378	0.000	0.962409441	-12.883	0.000	0.9693605	0.03586228	1	1.00000	1
ALT/AST	chr8:71507263:T:G	4E-10	rs10096191-G	aspartate aminotransferase levels;alanine aminotransferase levels	T	G	-0.190	0.827	0.119809746	-0.179	0.836	0.3530138	0.03632048	1	0.57671	1
ALT/AST	chr6:13512277:G:A	4E-18	rs2841532-A	aspartate aminotransferase levels	G	A	0.084	1.087	0.210633463	0.129	1.138	0.1909775	0.03639702	1	0.43309	1
ALT/AST	chr8:133543494:T:C	7E-10	rs58504358-T	aspartate aminotransferase levels	T	C	-0.232	0.793	0.003143263	0.107	1.113	0.3324864	0.03649966	1	0.74045	1
ALT/AST	chr19:40991441:A:G	0.000000001	M46V	CPIC:CYP2B6	A	G	-0.874	0.417	0.080883601	-0.353	0.703	0.7137101	0.03786796	1	0.40325	1
ALT/AST	chr15:40038428:C:T	1E-17	rs8041057-C	alanine aminotransferase levels;aspartate aminotransferase levels	C	T	-0.155	0.857	0.013407567	0.041	1.042	0.6591009	0.03806064	1	0.02297	1
ALT/AST	chr7:73612048:C:T	0.000000003	rs17145750-C	liver enzyme levels (gamma-glutamyl transferase)	C	T	0.220	1.246	0.009882584	-0.033	0.968	0.7921485	0.0380789	1	0.40385	1
ALT/AST	chr11:126413890:C:T	0.000000002	rs2236653-T	liver enzyme levels (alkaline phosphatase)	C	T	-0.172	0.842	0.004540085	0.110	1.116	0.2273384	0.04113396	1	0.67356	1
ALT/AST	chr13:73550539:G:A	1E-10	rs12876036-G	aspartate aminotransferase levels	G	A	-0.111	0.895	0.067156842	-0.064	0.938	0.4897241	0.04271129	1	0.85334	1

© 2022 Yang W et al. *JAMA Network Open*.

ALT/AST	chr5:150064431:C:T	0.000000001	rs216141-C	aspartate aminotransferase levels	C	T	-0.152	0.859	0.01751067	0.008	1.008	0.9328406	0.04306755	1	0.00666	0.938462
ALT/AST	chr6:18138997:C:T	0.000000001	A154T	CPIC:TPMT	C	T	-0.219	0.804	0.194724845	-0.382	0.683	0.1732051	0.04378963	1	0.00003	0.050513
ALT/AST	chr22:42129174:C:A	0.000000001	p.A122S	CPIC:CYP2D6	C	A	0.868	2.382	0.328099338	3.628	37.620	0.0446909	0.04522544	1	0.85021	1
ALT/AST	chr10:94849995:C:T	0.000000001	R410C	CPIC:CYP2C19	C	T	0.575	1.777	0.526076128	2.160	8.671	0.0265672	0.04676729	1	0.98581	1
ALT/AST	chr7:117509089:C:T	0.000000001	R74W	CPIC:CFTR	C	T	-0.940	0.390	0.516738817	2.807	16.568	0.004516	0.04876142	1	0.99152	1
ALT/AST	chr7:99652770:T:TA	0.000000001	346Frameshift	CPIC:CYP3A5	T	TA	0.132	1.141	0.740199451	0.632	1.881	0.0459762	0.04934666	1	0.58458	1
ALT/AST	chr12:11638751:A:G	8E-13	rs12824533-G	alanine aminotransferase levels	A	G	0.018	1.018	0.77419906	0.221	1.247	0.0178276	0.04994487	1	0.79087	1
ALT/AST	chr14:50734993:C:T	6E-12	rs10148309-C	alanine aminotransferase levels	C	T	-0.067	0.935	0.371731913	-0.229	0.795	0.0456371	0.05030443	1	0.20056	1
ALT/AST	chr2:27518370:T:C	0.00000002	rs780094-T	nonalcoholic fatty liver disease	T	C	0.091	1.095	0.140078903	0.082	1.085	0.3706183	0.05067556	1	0.75921	1
ALT/AST	chr10:94989020:C:T	0.000000001	p.P489S	CPIC:CYP2C9	C	T	-0.421	0.657	0.525182997	-14.798	0.000	0.9731516	0.05372673	1	0.09153	1
ALT/AST	chr12:52891302:T:G	2E-10	rs11170319-G	aspartate aminotransferase levels	T	G	0.054	1.055	0.368064865	0.150	1.162	0.1047889	0.05433982	1	0.64714	1
ALT/AST	chr1:247438055:G:A	2E-10	rs12143966-G	aspartate aminotransferase levels	G	A	-0.101	0.904	0.092113714	-0.070	0.933	0.4531197	0.05450686	1	0.13699	1
ALT/AST	chr3:56831417:T:C	1E-14	rs11925835-?	aspartate aminotransferase platelet ratio index in high alcohol intake	T	C	-0.110	0.896	0.07311523	-0.018	0.982	0.8398319	0.0559726	1	0.81402	1
ALT/AST	chr12:52901297:T:G	0.00000001	rs4531558-G	alanine aminotransferase levels	T	G	0.063	1.065	0.291878384	0.125	1.133	0.1718233	0.05602907	1	0.54351	1
ALT/AST	chr1:97549726:G:C	0.000000001	p.P453R	CPIC:DPYD	G	C	-11.861	0.000	0.96445135	-13.072	0.000	0.9718739	0.05677647	1	1.00000	1
ALT/AST	chr9:133458632:A:G	8E-21	rs4962153-A	liver enzyme levels	A	G	0.096	1.101	0.243970399	0.108	1.114	0.3672764	0.05841666	1	0.03001	1
ALT/AST	chr12:57445390:G:T	6E-25	rs61352607-T	aspartate aminotransferase levels	G	T	0.062	1.064	0.364050806	0.151	1.164	0.1611516	0.06072883	1	0.60693	1
ALT/AST	chr22:24594246:A:G	4E-11	rs4820599-G	liver enzyme levels	A	G	0.127	1.136	0.04636401	-0.013	0.987	0.8873057	0.06159611	1	0.45896	1

© 2022 Yang W et al. *JAMA Network Open*.

ALT/AST	chr2:113999632:G:C	1E-13	rs17046767-G	alanine aminotransferase levels	G	C	-0.212	0.809	0.063015241	-0.018	0.982	0.9130456	0.06326006	1	0.42753	1
ALT/AST	chr3:58394738:A:T	4E-40	rs11714574-A	aspartate aminotransferase levels	A	T	-0.071	0.931	0.23597303	-0.117	0.889	0.1925634	0.06354733	1	0.30721	1
ALT/AST	chr16:53772541:A:G	0.000000002	rs56094641-G	alanine aminotransferase levels	A	G	0.057	1.058	0.390345851	0.172	1.188	0.0751648	0.06367837	1	0.76053	1
ALT/AST	chr1:97573919:G:A	0.000000001	p.R394W	CPIC:DPYD	G	A	-11.635	0.000	0.959368941	-13.072	0.000	0.9718739	0.06438894	1	1.00000	1
ALT/AST	chr2:31251621:A:C	7E-10	rs609316-?	aspartate aminotransferase platelet ratio index in high alcohol intake	A	C	0.112	1.118	0.098395402	0.045	1.046	0.6650047	0.06492564	1	0.67695	1
ALT/AST	chr2:102130853:A:G	0.00000001	rs1024794-G	alanine aminotransferase levels	A	G	0.074	1.077	0.240661685	0.093	1.098	0.3407039	0.06562155	1	0.70899	1
ALT/AST	chr10:99052093:C:G	1E-12	rs7097660-G	aspartate aminotransferase levels	C	G	0.087	1.090	0.191934058	0.126	1.134	0.2345643	0.07057464	1	0.00538	0.912821
ALT/AST	chr7:117642528:G:A	0.000000001	D1270N	CPIC:CFTR	G	A	-1.052	0.349	0.454749984	2.807	16.568	0.004516	0.0706437	1	0.97814	1
ALT/AST	chr10:17933413:T:C	2E-19	rs10827785-?	aspartate aminotransferase levels	T	C	0.051	1.052	0.441891475	0.160	1.173	0.099798	0.07207066	1	0.53693	1
ALT/AST	chr6:27332531:A:G	8E-30	rs13212562-A	aspartate aminotransferase levels	A	G	0.105	1.111	0.273175859	0.176	1.193	0.2139624	0.07237148	1	0.17294	1
ALT/AST	chr19:48722509:A:T	3E-14	rs11882796-T	aspartate aminotransferase levels	A	T	0.079	1.082	0.212610521	0.114	1.120	0.2294891	0.07427147	1	0.06444	1
ALT/AST	chr22:42129087:G:C	0.000000001	p.Q151E	CPIC:CYP2D6	G	C	-0.849	0.428	0.140936049	-0.402	0.669	0.5590482	0.07430666	1	0.67227	1
ALT/AST	chr22:42130773:C:T	0.000000001	p.V7M	CPIC:CYP2D6	C	T	-0.849	0.428	0.140936049	-0.402	0.669	0.5590482	0.07430666	1	0.67227	1
ALT/AST	chr12:21130019:T:G	4E-14	rs4149014-G	bilirubin levels	T	G	-0.191	0.826	0.352219116	-0.409	0.665	0.2042118	0.07762309	1	0.86015	1

© 2022 Yang W et al. *JAMA Network Open*.

ALT/AST	chr19:18111154:A:C	9E-23	rs273507-C	alanine aminotransferase levels	A	C	0.119	1.126	0.080165228	0.048	1.049	0.6320301	0.07958125	1	0.08799	1
ALT/AST	chr12:21239042:A:C	0.000000001	L643F	CPIC:SLCO1B1	A	C	0.054	1.056	0.703728935	0.446	1.563	0.0236933	0.08033399	1	0.81777	1

© 2022 Yang W et al. *JAMA Network Open*.

ALT/AST	chr2:178480456:C:T	6E-16	rs71423566-T	aspartate aminotransferase levels;alanine aminotransferase levels	C	T	0.078	1.082	0.274519331	0.125	1.133	0.2399349	0.08204183	1	0.95207	1
ALT/AST	chr11:94131227:C:G	9E-38	rs56175344-C	aspartate aminotransferase levels	C	G	-0.092	0.913	0.30413575	-0.137	0.872	0.3047486	0.08343734	1	0.16652	1
ALT/AST	chr14:72545177:A:G	0.00000002	rs2239222-A	aspartate aminotransferase levels	A	G	0.133	1.142	0.029930299	-0.037	0.963	0.6803084	0.08631835	1	0.38048	1
ALT/AST	chr1:97078987:G:T	0.000000001	p.P1023T	CPIC:DPYD	G	T	-0.101	0.904	0.891784775	-13.772	0.000	0.9642217	0.08638116	1	0.30087	1
ALT/AST	chr3:157080986:T:C	6E-13	rs900400-C	alanine aminotransferase levels	T	C	0.084	1.087	0.160084673	0.044	1.044	0.6269006	0.08706152	1	0.74751	1
ALT/AST	chr14:20470092:A:C	1E-11	rs1760940-C	aspartate aminotransferase levels;alanine aminotransferase levels	A	C	0.088	1.092	0.241016534	0.110	1.116	0.3054929	0.08733982	1	0.35984	1
ALT/AST	chr2:164700808:C:T	3E-17	rs6712203-C	alanine aminotransferase levels	C	T	-0.087	0.917	0.187479584	-0.056	0.945	0.5598141	0.08769304	1	0.37101	1
ALT/AST	chr14:34715117:T:C	9E-13	rs10141892-C	alanine aminotransferase levels;aspartate aminotransferase levels	T	C	0.088	1.092	0.142930576	0.053	1.055	0.5517986	0.08997541	1	0.30917	1
ALT/AST	chr1:97699474:T:C	0.000000001	p.Y186C	CPIC:DPYD	T	C	1.846	6.332	0.047146053	-0.044	0.957	0.9746169	0.0902041	1	0.03919	1
ALT/AST	chr16:54381615:G:A	2E-36	rs74367983-A	aspartate aminotransferase levels	G	A	-0.142	0.868	0.253094329	-0.193	0.824	0.3662496	0.09279176	1	0.54878	1
ALT/AST	chr1:183833125:T:C	1E-34	rs2491441-C	aspartate aminotransferase levels	T	C	-0.105	0.901	0.190868622	-0.086	0.918	0.4653905	0.09341239	1	0.38532	1
ALT/AST	chr12:20871928:T:G	8E-106	rs73233620-?	total bilirubin levels;bilirubin levels	T	G	-0.144	0.866	0.344338068	-0.205	0.815	0.340623	0.09507398	1	0.18989	1

© 2022 Yang W et al. *JAMA Network Open*.

ALT/AST	chr1:97573918:C:A	0.000000001	p.R394L	CPIC:DPYD	C	A	-12.564	0.000	0.956126001	-12.093	0.000	0.9739796	0.09553286	1	1.00000	1
ALT/AST	chr16:11186056:G:A	8E-10	rs193759-A	aspartate aminotransferase levels	G	A	-0.051	0.950	0.427334133	-0.143	0.867	0.1358466	0.09738144	1	0.23979	1
ALT/AST	chr2:233527423:C:T	7E-12	rs36075906-T	bilirubin levels	C	T	0.152	1.165	0.137604366	-0.058	0.943	0.7354617	0.09785083	1	0.09605	1
ALT/AST	chr11:94137172:C:T	7E-55	rs7117339-C	alanine aminotransferase levels	C	T	-0.084	0.919	0.342155504	-0.135	0.874	0.3102819	0.09838288	1	0.11491	1
ALT/AST	chr1:16178825:A:G	1E-54	rs1497406-G	alanine aminotransferase levels;liver enzyme levels (gamma-glutamyl transferase)	A	G	0.050	1.051	0.405113877	0.142	1.152	0.1225721	0.10046636	1	0.44095	1
ALT/AST	chr3:155844283:C:T	0.000000008	rs6441017-C	alanine aminotransferase levels	C	T	-0.120	0.887	0.063595515	0.011	1.011	0.9068842	0.10263068	1	0.28328	1
ALT/AST	chr19:10222312:C:T	1E-16	rs10409243-C	alanine aminotransferase levels	C	T	0.051	1.052	0.410838932	0.137	1.146	0.1397677	0.10343034	1	0.07145	1
ALT/AST	chr12:21178957:A:G	0.000000001	I222V	CPIC:SLCO1B1	A	G	0.302	1.352	0.848802824	-14.104	0.000	0.9708748	0.10437166	1	0.78776	1
ALT/AST	chr12:121004867:T:C	2E-10	rs1169313-C	liver enzyme levels	T	C	0.066	1.068	0.288739158	0.072	1.075	0.4394651	0.10535298	1	0.57445	1
ALT/AST	chr7:117614699:G:C	0.000000001	D1152H	CPIC:CFTR	G	C	-1.475	0.229	0.293965613	-12.953	0.000	0.9721288	0.10895525	1	0.79995	1
ALT/AST	chr12:12362395:A:G	0.00000002	rs11054848-G	aspartate aminotransferase levels	A	G	0.030	1.030	0.634128578	0.156	1.168	0.1017907	0.11028048	1	0.04302	1
ALT/AST	chr1:109928142:G:A	2E-64	rs333947-G	aspartate aminotransferase levels	G	A	0.069	1.072	0.359851518	0.151	1.163	0.2109632	0.1103696	1	0.00563	0.923077
ALT/AST	chr12:109631385:G:T	0.00000003	rs11067592-?	aspartate aminotransferase levels	G	T	-1.323	0.266	0.173504634	-0.413	0.662	0.5452109	0.11103128	1	0.04203	1
ALT/AST	chr3:186933001:A:G	0.00000001	rs10937275-?	drug-induced liver injury (flucloxacillin)	A	G	-0.093	0.911	0.35468148	-0.166	0.847	0.2384606	0.11126012	1	0.60091	1
ALT/AST	chr14:93998863:G:A	4E-10	rs10145626-A	aspartate aminotransferase levels	G	A	0.064	1.066	0.480455793	0.247	1.280	0.0812282	0.11288267	1	0.88381	1

© 2022 Yang W et al. *JAMA Network Open*.

ALT/AST	chr10:17839303:A:C	2E-42	rs377536650-	aspartate aminotransferase levels	A	C	0.090	1.094	0.138929275	0.027	1.027	0.765899	0.11350668	1	0.13789	1
ALT/AST	chr9:133278724:C:T	3E-123	rs579459-T	liver enzyme levels (alkaline phosphatase)	C	T	0.064	1.066	0.399898785	0.116	1.123	0.2975981	0.11375193	1	0.59189	1
ALT/AST	chr21:29349061:T:C	8E-11	rs412662-C	aspartate aminotransferase levels	T	C	-0.052	0.949	0.38431903	-0.098	0.907	0.2676171	0.11606544	1	0.40629	1
ALT/AST	chr2:233934849:C:T	1E-10	rs10490012-T	bilirubin levels	C	T	0.088	1.092	0.179655981	0.043	1.044	0.6743549	0.1166782	1	0.59322	1
ALT/AST	chr7:28139639:C:A	0.000000001	rs13247499-?	alanine transaminase levels	C	A	0.115	1.122	0.186862628	0.040	1.040	0.7606014	0.11965488	1	0.47659	1
ALT/AST	chr6:127113961:A:T	0.00000003	rs6916318-T	aspartate aminotransferase levels	A	T	-0.082	0.921	0.169789257	-0.031	0.969	0.721181	0.12017482	1	0.13675	1
ALT/AST	chr10:94942309:G:A	0.000000001	p.R150H; p.R1	CPIC:CYP2C9	G	A	0.456	1.579	0.427302835	0.962	2.617	0.1277719	0.12061054	1	0.19922	1
ALT/AST	chr18:58419588:C:T	2E-38	rs9959832-C	alanine aminotransferase levels	C	T	0.112	1.119	0.137627316	0.020	1.020	0.8601925	0.12128051	1	0.50467	1
ALT/AST	chr2:233257131:G:A	5E-37	rs2119503-A	bilirubin levels	G	A	0.142	1.152	0.074716363	-0.049	0.952	0.6783645	0.12202166	1	0.45660	1
ALT/AST	chr15:51201770:G:A	2E-13	rs11070841-A	aspartate aminotransferase levels	G	A	-0.113	0.893	0.136747498	-0.029	0.972	0.7919042	0.12505478	1	0.43909	1
ALT/AST	chr22:36131919:A:G	0.00000001	rs5756080-?	alanine transaminase levels in high alcohol intake	A	G	0.106	1.112	0.120117631	0.004	1.004	0.9673227	0.12744578	1	0.17302	1
ALT/AST	chr10:94981224:C:T	0.000000001	p.R335W	CPIC:CYP2C9	C	T	-0.643	0.526	0.213968373	-0.379	0.685	0.5898944	0.12745594	1	0.53511	1
ALT/AST	chr12:21178665:T:C	0.000000001	L191=	CPIC:SLCO1B1	T	C	0.092	1.097	0.133148911	-0.015	0.985	0.8716753	0.12950206	1	0.11031	1
ALT/AST	chr1:97595149:T:C	0.000000001	p.K290E	CPIC:DPYD	T	C	-11.889	0.000	0.964376931	-11.957	0.000	0.9742714	0.13576854	1	1.00000	1
ALT/AST	chr22:42127611:C:T	0.000000001	p.D337N	CPIC:CYP2D6	C	T	-11.643	0.000	0.9522049	-10.456	0.000	0.9743153	0.13664072	1	1.00000	1
ALT/AST	chr5:75054108:G:A	2E-15	rs76722284-A	aspartate aminotransferase levels	G	A	-0.099	0.906	0.300137819	-0.195	0.823	0.2345498	0.13752578	1	0.14868	1
ALT/AST	chr3:142940519:A:AT	3E-11	rs6440123-A	alanine aminotransferase levels	A	AT	-1.654	0.191	0.216120721	-0.372	0.689	0.8310812	0.14356945	1	0.41973	1

© 2022 Yang W et al. *JAMA Network Open*.

ALT/AST	chr21:28760989:T:C	0.000000002	rs2832059-?	alanine aminotransferase levels	T	C	0.019	1.019	0.868077393	-0.402	0.669	0.0142672	0.14362434	1	0.39948	1

ALT/AST	chr7:73586691:C:T	7E-16	rs35173225-T	alanine aminotransferase levels	C	T	0.186	1.205	0.056919522	-0.040	0.961	0.7810388	0.14483535	1	0.44366	1
ALT/AST	chr2:31254972:G:A	0.000000002	rs655029-?	aspartate aminotransferase platelet ratio index in high alcohol intake	G	A	0.097	1.102	0.171891784	0.025	1.025	0.8190622	0.14676321	1	0.37642	1
ALT/AST	chr1:219494910:C:A	1E-11	rs1538742-A	alanine aminotransferase levels	C	A	0.076	1.078	0.224149621	0.063	1.065	0.5007783	0.14763784	1	0.62392	1
ALT/AST	chr19:19268740:C:T	1E-71	rs58542926-T	alanine aminotransferase levels;aspartate aminotransferase levels;liver fat content (mri proton density fat fraction measure);liver fibrosis and steatohepatitis severity (mri ct1 measure);nonalcoholic fatty liver disease	C	T	-0.057	0.944	0.622947368	-0.304	0.738	0.0956757	0.14829072	1	0.58814	1
ALT/AST	chr10:94775423:A:C	0.000000001	E122A	CPIC:CYP2C19	A	C	1.118	3.057	0.371786802	0.911	2.486	0.3259729	0.14847772	1	0.27735	1
ALT/AST	chr10:94775367:A:G	0.000000001	splicing defect	CPIC:CYP2C19	A	G	-0.116	0.891	0.165179777	-0.035	0.965	0.7665234	0.14992866	1	0.48452	1
ALT/AST	chr14:50390914:A:G	3E-13	rs10134101-A	aspartate aminotransferase levels	A	G	0.054	1.056	0.633877014	0.270	1.310	0.1304159	0.15083246	1	0.62190	1

© 2022 Yang W et al. *JAMA Network Open*.

ALT/AST	chr1:16184399:C:T	1E-39	rs36086195-T	aspartate aminotransferase levels;alanine aminotransferase levels	C	T	0.028	1.029	0.640104711	0.168	1.183	0.0689589	0.15099502	1	0.47433	1
ALT/AST	chr10:98367794:G:A	3E-14	rs17109512-?	aspartate aminotransferase levels	G	A	0.019	1.019	0.855329133	0.291	1.338	0.0611021	0.15271491	1	0.51760	1
ALT/AST	chr11:5679844:C:A	7E-24	rs11601507-A	alanine aminotransferase levels;aspartate aminotransferase levels	C	A	0.085	1.089	0.447627347	0.192	1.212	0.2629203	0.15310797	1	0.06560	1
ALT/AST	chr18:58416822:T:C	3E-12	rs4503880-T	liver enzyme levels (gamma-glutamyl transferase)	T	C	0.088	1.092	0.246448081	0.049	1.050	0.6631388	0.15543039	1	0.70767	1
ALT/AST	chr19:49509317:G:A	6E-10	rs34010237-?	aspartate aminotransferase levels	G	A	0.004	1.004	0.963156271	0.270	1.309	0.0269183	0.15548218	1	0.90244	1
ALT/AST	chr5:107543091:G:C	6E-11	rs6879279-G	alanine aminotransferase levels	G	C	-0.075	0.927	0.372496432	-0.083	0.920	0.492654	0.15763131	1	0.62104	1
ALT/AST	chr1:159886639:C:T	2E-10	rs2501324-?	bilirubin levels	C	T	0.609	1.838	0.17547745	0.173	1.189	0.7319831	0.16091765	1	0.34420	1
ALT/AST	chr3:136207780:G:T	3E-29	rs645040-T	alanine aminotransferase levels;alanine transaminase levels	G	T	-0.071	0.932	0.326405134	-0.089	0.915	0.4040131	0.16141104	1	0.93727	1
ALT/AST	chr7:21480514:G:A	0.000000006	rs10272006-G	aspartate aminotransferase levels in non-alcoholic fatty liver disease	G	A	-0.026	0.975	0.696375169	-0.155	0.856	0.12082	0.16344678	1	0.37887	1
ALT/AST	chr3:125208361:G:A	0.000000005	rs6762589-A	alanine aminotransferase levels	G	A	-0.014	0.986	0.812851436	-0.157	0.855	0.0809617	0.1650951	1	0.96182	1
ALT/AST	chr6:18133847:C:A	0.000000001	Q179H	CPIC:TPMT	C	A	1.131	3.098	0.121241022	0.133	1.142	0.8613546	0.16530602	1	0.65437	1

© 2022 Yang W et al. *JAMA Network Open*.

ALT/AST	chr20:3925431:A:G	0.00000002	rs241597-G	aspartate aminotransferase levels	A	G	-0.031	0.969	0.747098164	0.333	1.395	0.0104766	0.16824972	1	0.66167	1

© 2022 Yang W et al. *JAMA Network Open*.

ALT/AST	chr19:19296909:T:C	1E-18	rs10401969-?	alanine transaminase levels in high alcohol intake;aspartate transaminase levels in high alcohol intake;aspartate aminotransferase platelet ratio index in high alcohol intake	T	C	-0.014	0.986	0.893166202	-0.343	0.709	0.0406164	0.16880899	1	0.38407	1
ALT/AST	chr22:43958231:C:T	1E-18	rs738491-T	nonalcoholic fatty liver disease	C	T	0.002	1.002	0.979398861	0.195	1.215	0.0366421	0.17006176	1	0.41371	1
ALT/AST	chr18:63178651:T:C	5E-13	rs12454712-T	alanine aminotransferase levels	T	C	0.074	1.076	0.237949352	0.041	1.042	0.6529059	0.17010195	1	0.29532	1
ALT/AST	chr1:161531185:A:G	8E-11	rs6700241-A	alanine aminotransferase levels	A	G	0.098	1.103	0.190691889	0.035	1.036	0.743439	0.17274033	1	0.22464	1
ALT/AST	chr14:24103723:G:A	3E-11	rs111723834-	liver fibrosis and steatohepatitis severity (mri ct1 measure)	G	A	0.225	1.252	0.386309809	0.453	1.573	0.3074214	0.17467247	1	0.89988	1
ALT/AST	chr4:102267552:C:T	1E-133	rs13107325-T	liver fibrosis and steatohepatitis severity (mri ct1 measure)	C	T	0.146	1.157	0.227534731	0.049	1.050	0.8036736	0.17663387	1	0.31952	1
ALT/AST	chr14:24402720:C:T	6E-13	rs11621792-T	alanine aminotransferase levels	C	T	-0.057	0.945	0.379627874	-0.074	0.928	0.4366383	0.17977735	1	0.89322	1

© 2022 Yang W et al. *JAMA Network Open*.

ALT/AST	chr14:94378610:C:T	1E-17	rs28929474-?	alanine aminotransferase levels;alanine transaminase levels;alanine transaminase levels in high alcohol intake;aspartate transaminase levels in high alcohol intake	C	T	0.161	1.175	0.51986803	0.509	1.664	0.1954949	0.18035289	1	0.61130	1
ALT/AST	chr16:68526282:A:G	3E-11	rs698718-A	liver fibrosis in non-alcoholic fatty acid liver disease	A	G	0.064	1.066	0.345658306	0.082	1.085	0.4110689	0.18293534	1	0.36284	1
ALT/AST	chr7:87450090:T:A	2E-18	rs2109505-?	alanine transaminase levels	T	A	-0.098	0.907	0.195236418	-0.017	0.984	0.8787721	0.18351378	1	0.71772	1
ALT/AST	chr5:179863845:T:G	0.00000004	rs30386-G	aspartate aminotransferase levels	T	G	0.098	1.102	0.112390686	-0.039	0.962	0.6746529	0.1892811	1	0.10914	1
ALT/AST	chr22:23924539:T:C	0.000000002	rs5751775-T	alanine aminotransferase levels	T	C	0.066	1.068	0.278797545	0.059	1.061	0.5214394	0.18966759	1	0.69158	1
ALT/AST	chr2:43839108:G:C	5E-11	rs11887534-C	alanine aminotransferase levels	G	C	0.192	1.212	0.099109891	-0.119	0.888	0.5255423	0.19021472	1	0.64332	1
ALT/AST	chr2:88124547:T:C	0.000000004	rs2241883-T	alanine aminotransferase levels	T	C	0.027	1.028	0.670131979	0.124	1.132	0.2069815	0.19382904	1	0.84754	1
ALT/AST	chr6:32606583:C:T	1E-13	rs2647074-?	aspartate aminotransferase levels;total bilirubin levels	C	T	-0.027	0.973	0.685084923	-0.128	0.880	0.1974699	0.19460687	1	0.88334	1

© 2022 Yang W et al. *JAMA Network Open*.

ALT/AST	chr19:19308262:G:A	1E-18	rs739846-?	alanine transaminase levels in high alcohol intake;aspartate transaminase levels in high alcohol intake;aspartate aminotransferase platelet ratio index in high alcohol intake	G	A	-0.014	0.986	0.893166202	-0.316	0.729	0.057871	0.19720791	1	0.43227	1
ALT/AST	chr10:94781859:G:A	0.000000001	splicing defect	CPIC:CYP2C19	G	A	-0.102	0.903	0.22473899	-0.035	0.966	0.7709182	0.19777722	1	0.42495	1
ALT/AST	chr22:42129819:G:T	0.000000001	p.L91M	CPIC:CYP2D6	G	T	0.156	1.169	0.070206231	-0.098	0.907	0.4729325	0.19966503	1	0.00598	0.928205
ALT/AST	chr10:94780653:G:A	0.000000001	W212X	CPIC:CYP2C19	G	A	-0.507	0.602	0.431539058	-0.554	0.575	0.412702	0.20042112	1	0.35479	1
ALT/AST	chr22:42129809:T:C	0.000000001	p.H94R	CPIC:CYP2D6	T	C	0.152	1.164	0.076770025	-0.086	0.917	0.5236281	0.20206758	1	0.00436	0.85641
ALT/AST	chr2:233732958:C:G	0	rs75520741-?	total bilirubin levels	C	G	0.139	1.149	0.307669796	0.093	1.097	0.6479626	0.20272069	1	0.90990	1
ALT/AST	chr8:52506268:T:A	0.00000002	rs7820212-A	immunoglobulin light chain (al) amyloidosis (liver involvement)	T	A	-0.059	0.943	0.336537794	-0.068	0.934	0.4563916	0.2035341	1	0.77862	1

ALT/AST	chr10:77814320:G:A	1E-16	rs11002301-A	aspartate aminotransferase levels	G	A	0.061	1.063	0.338392413	0.065	1.068	0.4961829	0.20414312	1	0.45667	1
ALT/AST	chr19:40827379:T:C	7E-20	rs11878604-?	alanine transaminase levels;alanine aminotransferase levels;aspartate aminotransferase levels	T	C	0.074	1.077	0.410939816	0.110	1.117	0.4142134	0.20498462	1	0.87860	1
ALT/AST	chr11:2921363:G:A	5E-11	rs1661052-?	total bilirubin levels	G	A	0.121	1.129	0.194038155	0.043	1.044	0.7653865	0.20504358	1	0.83564	1
ALT/AST	chr7:87450286:G:C	1E-19	rs4148821-?	alanine aminotransferase levels	G	C	-0.093	0.911	0.218525746	-0.016	0.984	0.8830233	0.2069713	1	0.74313	1
ALT/AST	chr18:72540105:A:G	0.000000003	rs658995-A	serum bilirubin levels in metabolic syndrome	A	G	0.146	1.157	0.259075624	0.049	1.050	0.7724231	0.20836223	1	0.96275	1
ALT/AST	chr1:2244114:C:T	0.000000002	rs142451335-	alanine aminotransferase levels	C	T	0.095	1.100	0.431707732	0.181	1.198	0.3209512	0.20952333	1	0.40111	1

© 2022 Yang W et al. *JAMA Network Open*.

ALT/AST	chr19:39248147:C:T	0.000000001	unknown (ups	CPIC:IFNL3	C	T	0.120	1.128	0.064687746	-0.069	0.933	0.461712	0.2096611	1	0.82204	1
ALT/AST	chr7:117530975:G:A	0.000000001	R117H	CPIC:CFTR	G	A	0.461	1.586	0.367542952	1.162	3.196	0.4673356	0.20974942	1	0.78384	1
ALT/AST	chr6:69829361:G:A	0.000000003	rs77249491-?	percent liver fat;nonalcoholic fatty liver disease	G	A	0.915	2.498	0.083314463	-0.120	0.887	0.8578584	0.21380777	1	0.04344	1
ALT/AST	chr9:33117967:C:T	6E-14	rs7865362-?	aspartate aminotransferase levels	C	T	0.084	1.088	0.18506763	0.024	1.024	0.800094	0.21524159	1	0.93436	1
ALT/AST	chr1:97450058:C:T	0.000000001	NA	CPIC:DPYD	C	T	-0.350	0.705	0.610673656	-0.922	0.398	0.3163353	0.21585243	1	0.35623	1
ALT/AST	chr4:87063179:A:G	0.00000004	rs28432336-?	total bilirubin levels	A	G	-0.007	0.993	0.908309278	0.197	1.218	0.0324467	0.21645278	1	0.22784	1
ALT/AST	chr19:7766742:G:A	2E-21	rs2277998-G	aspartate aminotransferase levels	G	A	-0.046	0.955	0.479137009	-0.099	0.906	0.3352219	0.21872933	1	0.17490	1
ALT/AST	chr12:111433337:A:T	1E-26	rs4766462-A	alanine aminotransferase levels	A	T	-0.022	0.978	0.742266381	-0.148	0.863	0.1354783	0.22123846	1	0.66254	1
ALT/AST	chrX:154532945:C:G	0.000000001	p.D350H	CPIC:G6PD	C	G	1.644	5.176	0.139811156	-4.771	0.008	0.9765611	0.22159589	1	0.15155	1
ALT/AST	chr19:41009350:C:T	0.000000001	S259R	CPIC:CYP2B6	C	T	2.428	11.339	0.061784118	-12.802	0.000	0.9778499	0.22329018	1	0.60162	1
ALT/AST	chr6:117180825:T:C	4E-14	rs9372475-T	aspartate aminotransferase levels	T	C	-0.057	0.944	0.375400729	-0.062	0.940	0.5184838	0.22331989	1	0.14413	1
ALT/AST	chr2:5092045:A:G	0.00000001	rs72631567-?	drug-induced liver injury;drug-induced cholestatic/mixed liver injury	A	G	0.167	1.181	0.213463554	0.039	1.040	0.8423669	0.22416017	1	0.28115	1
ALT/AST	chr1:109279521:G:A	0.000000003	rs1277930-A	alanine aminotransferase levels	G	A	0.091	1.095	0.200518232	-0.014	0.986	0.8902428	0.22808163	1	0.94356	1
ALT/AST	chr11:78416868:A:G	2E-23	rs869202-A	aspartate aminotransferase levels	A	G	-0.008	0.992	0.914199239	-0.219	0.803	0.0556105	0.22860381	1	0.17420	1
ALT/AST	chr7:28143829:T:C	0.00000002	rs4722763-?	alanine aminotransferase levels	T	C	0.100	1.105	0.262821917	0.003	1.003	0.9843791	0.22891297	1	0.41842	1

© 2022 Yang W et al. *JAMA Network Open*.

ALT/AST	chr15:84626943:T:C	0.000000001	rs61394864-C	aspartate aminotransferase levels	T	C	-0.044	0.957	0.458177327	-0.085	0.918	0.3484402	0.22940603	1	0.06567	1
ALT/AST	chr9:104903697:C:G	0.000000003	rs1800978-C	aspartate aminotransferase levels	C	G	-0.010	0.990	0.907553972	0.265	1.303	0.0284164	0.23136666	1	0.00093	0.307692
ALT/AST	chr1:220801203:T:C	3E-10	rs10863565-C	aspartate aminotransferase levels	T	C	0.128	1.137	0.09047325	-0.084	0.919	0.4591576	0.23149066	1	0.18729	1
ALT/AST	chr19:49495592:G:C	0.00000002	rs34750569-?	aspartate aminotransferase levels	G	C	-0.004	0.996	0.967590463	0.238	1.269	0.0484649	0.23557651	1	0.92314	1
ALT/AST	chr8:58553547:T:C	1E-11	rs113895159-	aspartate aminotransferase levels	T	C	0.065	1.068	0.307147905	0.056	1.057	0.5443446	0.23666017	1	0.25808	1
ALT/AST	chr3:37042249:A:G	0.00000002	rs9876116-A	alanine aminotransferase levels	A	G	0.038	1.038	0.542792472	0.094	1.098	0.2989062	0.23798057	1	0.05079	1

© 2022 Yang W et al. *JAMA Network Open*.

ALT/AST	chr3:160455812:A:C	8E-14	rs17236494-C	alanine aminotransferase levels;aspartate aminotransferase levels	A	C	-0.037	0.964	0.59447302	-0.123	0.884	0.2570543	0.23840388	1	0.76654	1
ALT/AST	chr7:92779056:C:T	6E-14	rs445-T	alanine aminotransferase levels	C	T	0.066	1.069	0.440335522	0.088	1.092	0.5006852	0.23986242	1	0.01791	1
ALT/AST	chr3:8764738:G:A	0.000000001	rs73132848-?	liver fibrosis severity in hiv/hepatitis c co-infection	G	A	0.077	1.081	0.564992323	0.170	1.185	0.3781152	0.24123559	1	0.18581	1
ALT/AST	chr4:102268971:A:G	2E-12	rs151401-G	aspartate aminotransferase levels	A	G	0.044	1.045	0.556227333	0.102	1.107	0.3552025	0.24221399	1	0.04867	1
ALT/AST	chr6:152721900:G:A	0.00000001	rs17710008-A	alanine aminotransferase levels	G	A	0.057	1.059	0.441602793	0.094	1.098	0.4306318	0.2434049	1	0.93592	1
ALT/AST	chr22:42129183:C:T	0.000000001	p.V119M	CPIC:CYP2D6	C	T	-11.039	0.000	0.961448864	-11.710	0.000	0.9782924	0.24648801	1	1.00000	1
ALT/AST	chr21:29236564:A:G	1E-16	rs2832277-G	alanine aminotransferase levels	A	G	-0.066	0.936	0.391732506	-0.032	0.969	0.7931638	0.2485396	1	0.26548	1
ALT/AST	chr17:46006582:T:C	2E-12	rs8067056-T	aspartate aminotransferase levels	T	C	-0.018	0.982	0.77139719	-0.122	0.885	0.1996468	0.24905335	1	0.78235	1
ALT/AST	chr19:46718300:C:G	6E-13	rs313839-G	aspartate aminotransferase levels	C	G	0.066	1.068	0.424467248	0.089	1.093	0.4846744	0.25347778	1	0.05061	1
ALT/AST	chr22:42129130:C:G	0.000000001	p.V136=	CPIC:CYP2D6	C	G	-0.041	0.960	0.490011348	-0.053	0.948	0.5531868	0.25750262	1	0.01317	1
ALT/AST	chr10:100035604:T:A	0.000000004	rs10883437-T	liver enzyme levels (alanine transaminase)	T	A	-0.041	0.960	0.498474457	-0.072	0.931	0.4352986	0.25836887	1	0.53993	1
ALT/AST	chr8:9327636:T:C	5E-34	rs2126259-T	alanine aminotransferase levels	T	C	0.125	1.133	0.150550206	0.003	1.003	0.9843767	0.25933407	1	0.59433	1
ALT/AST	chr3:17791337:C:T	0.00000001	rs6577596-C	aspartate aminotransferase levels	C	T	0.107	1.113	0.101868666	-0.072	0.930	0.4493826	0.26155249	1	0.20805	1

© 2022 Yang W et al. *JAMA Network Open*.

ALT/AST	chr5:53980471:G:A	6E-10	rs1664781-A	alanine aminotransferase levels	G	A	-0.089	0.915	0.190837427	0.005	1.005	0.9562454	0.26205705	1	0.82237	1
ALT/AST	chr15:60591082:C:A	7E-20	rs339969-A	liver enzyme levels (gamma-glutamyl transferase);alanine aminotransferase levels;aspartate aminotransferase levels	C	A	0.046	1.047	0.46568529	0.075	1.078	0.4166633	0.26341559	1	0.16642	1
ALT/AST	chr7:28152756:C:T	3E-20	rs12154248-T	alanine aminotransferase levels	C	T	0.092	1.096	0.30148972	0.003	1.003	0.9843791	0.26525507	1	0.47512	1
ALT/AST	chr8:10736964:C:A	2E-20	rs4841436-C	aspartate aminotransferase levels	C	A	0.010	1.010	0.875628164	0.152	1.164	0.0967916	0.26583311	1	0.23379	1
ALT/AST	chr6:29588001:A:G	2E-10	rs2235698-G	alanine aminotransferase levels	A	G	0.105	1.111	0.118907966	-0.059	0.942	0.5788527	0.26644563	1	0.81383	1
ALT/AST	chr19:40991224:T:C	0.000000001	5' region	CPIC:CYP2B6	T	C	0.188	1.206	0.526738086	0.307	1.360	0.4322733	0.26835232	1	0.84729	1
ALT/AST	chr20:14691531:T:C	0.000000005	rs6034011-C	alanine aminotransferase levels	T	C	-0.077	0.926	0.20817907	0.000	1.000	0.9992529	0.26848297	1	0.76555	1
ALT/AST	chr3:171007753:G:A	6E-11	rs10513686-A	liver enzyme levels (gamma-glutamyl transferase)	G	A	0.151	1.163	0.07121459	-0.095	0.909	0.4547152	0.26865263	1	0.76523	1
ALT/AST	chr3:136301699:T:C	2E-16	rs1711171-?	total bilirubin levels	T	C	-0.056	0.946	0.433721738	-0.065	0.937	0.5315058	0.26869205	1	0.82189	1
ALT/AST	chr21:28754160:A:G	8E-11	rs73186036-?	alanine transaminase levels	A	G	0.001	1.001	0.991325265	-0.218	0.804	0.0768165	0.26913631	1	0.74364	1
ALT/AST	chr17:31312307:G:C	1E-13	rs4638642-G	aspartate aminotransferase levels	G	C	-0.040	0.961	0.524699885	0.263	1.301	0.0040235	0.27022045	1	0.38575	1
ALT/AST	chr12:50827344:A:G	1E-10	rs864899-G	alanine aminotransferase levels	A	G	0.043	1.044	0.473002531	0.047	1.048	0.6045715	0.27324489	1	0.46718	1

© 2022 Yang W et al. *JAMA Network Open*.

ALT/AST	chr6:35316181:T:C	1E-11	rs13197551-T	alanine aminotransferase levels	T	C	-0.047	0.954	0.51858253	-0.100	0.905	0.3474411	0.27380534	1	0.92490	1
ALT/AST	chr10:100101678:G:A	0.00000002	rs11597390-A	liver enzyme levels	G	A	-0.067	0.936	0.293576214	-0.037	0.963	0.7009097	0.2748401	1	0.20873	1
ALT/AST	chr4:150759175:G:A	0.000000005	rs28521457-?	drug-induced hepatocellular liver injury	G	A	0.101	1.106	0.385766452	0.080	1.083	0.6273046	0.27769884	1	0.33477	1
ALT/AST	chr1:155134221:G:A	1E-30	rs12904-G	alanine aminotransferase levels;aspartate aminotransferase levels	G	A	-0.026	0.974	0.675067315	-0.104	0.901	0.2463629	0.28631683	1	0.90892	1
ALT/AST	chr6:33570484:C:T	8E-14	rs9380371-?	aspartate aminotransferase platelet ratio index in high alcohol intake	C	T	0.026	1.026	0.679090104	0.099	1.104	0.3267164	0.28686382	1	0.29938	1
ALT/AST	chr12:21202555:C:G	0.000000001	F400L	CPIC:SLCO1B1	C	G	0.049	1.050	0.928781954	-1.589	0.204	0.1569521	0.28779721	1	0.80186	1
ALT/AST	chr1:21439960:A:C	2E-50	rs1976403-C	liver enzyme levels (alkaline phosphatase)	A	C	0.070	1.072	0.275953448	0.024	1.025	0.7946619	0.2921024	1	0.49653	1
ALT/AST	chr16:88631079:G:A	1E-10	rs2291160-G	aspartate aminotransferase levels	G	A	0.054	1.056	0.368971639	0.014	1.014	0.8768452	0.29327914	1	0.33890	1
ALT/AST	chr6:33567103:C:T	6E-14	rs6914422-?	aspartate aminotransferase platelet ratio index in high alcohol intake	C	T	0.027	1.027	0.666327846	0.092	1.096	0.3620163	0.29364389	1	0.30743	1
ALT/AST	chr12:55991020:G:A	8E-11	rs705699-G	aspartate aminotransferase levels	G	A	-0.055	0.946	0.384240838	-0.027	0.974	0.7752177	0.29601099	1	0.21216	1
ALT/AST	chr5:39533216:T:A	1E-29	rs2910953-A	aspartate aminotransferase levels	T	A	0.123	1.130	0.07590304	-0.108	0.897	0.2920805	0.29948848	1	0.12237	1
ALT/AST	chr13:27865252:T:C	4E-12	rs1970619-T	alanine aminotransferase levels	T	C	0.070	1.073	0.243630299	-0.025	0.975	0.7831883	0.30032792	1	0.03738	1

© 2022 Yang W et al. *JAMA Network Open*.

ALT/AST	chr10:93050042:A:T	0.000000001	rs835278-?	total bilirubin levels	A	T	0.037	1.037	0.551422936	0.064	1.066	0.4705767	0.30167094	1	0.14629	1
ALT/AST	chr19:40832467:C:T	8E-14	rs3852868-?	aspartate aminotransferase levels	C	T	0.040	1.041	0.504764556	0.082	1.085	0.36372	0.30410295	1	0.42387	1
ALT/AST	chr8:141219046:A:G	5E-26	rs3739235-G	aspartate aminotransferase levels	A	G	0.089	1.093	0.151480694	-0.048	0.954	0.6072977	0.30437595	1	0.19248	1
ALT/AST	chr22:42126611:C:G	0.000000001	p.S486T	CPIC:CYP2D6	C	G	-0.039	0.961	0.506814048	-0.043	0.958	0.6317006	0.30578737	1	0.02369	1
ALT/AST	chr2:233762816:G:T	0	rs28946889-?	total bilirubin levels	G	T	-0.021	0.979	0.755392997	-0.124	0.883	0.2351698	0.30645547	1	0.07307	1
ALT/AST	chr1:247559286:G:A	1E-10	rs74227709-?	aspartate aminotransferase platelet ratio index in high alcohol intake	G	A	0.059	1.061	0.640122361	0.172	1.187	0.331375	0.30914251	1	0.95369	1
ALT/AST	chr22:42129180:A:T	0.000000001	p.F120I	CPIC:CYP2D6	A	T	0.103	1.108	0.92360467	1.789	5.983	0.1818623	0.3097483	1	0.47431	1
ALT/AST	chr20:25317451:A:G	7E-10	rs7267979-G	liver enzyme levels (alkaline phosphatase)	A	G	-0.013	0.987	0.833676695	-0.133	0.875	0.1437996	0.31050548	1	0.08282	1
ALT/AST	chr11:108566127:A:G	0.00000001	rs2846407-?	alanine aminotransferase levels	A	G	-0.102	0.903	0.182690814	0.034	1.035	0.7556504	0.31166089	1	0.59962	1
ALT/AST	chr13:48045720:G:A	0.000000001	R139H	CPIC:NUDT15	G	A	-0.235	0.790	0.668063086	-1.119	0.327	0.3189555	0.31293252	1	0.16348	1
ALT/AST	chr10:77920756:C:A	0.000000002	rs754465-?	alanine aminotransferase levels;alanine transaminase levels	C	A	-0.069	0.933	0.258176832	0.003	1.003	0.9746888	0.31961391	1	0.54107	1
ALT/AST	chr2:233800178:T:G	0.00000005	rs55686299-G	bilirubin levels	T	G	0.079	1.082	0.3250359	0.004	1.004	0.9718164	0.32586268	1	0.75469	1
ALT/AST	chr22:37073551:G:A	0.000000002	rs4820268-G	liver fibrosis and steatohepatitis severity (mri ct1 measure)	G	A	-0.099	0.905	0.095977202	0.084	1.088	0.3513256	0.32740245	1	0.69660	1
ALT/AST	chr19:41004377:A:G	0.000000001	K139E	CPIC:CYP2B6	A	G	-0.296	0.743	0.655122462	-0.697	0.498	0.4220655	0.32758803	1	0.11003	1
ALT/AST	chrX:154533025:A:G	0.000000001	p.L323P	CPIC:G6PD	A	G	-0.592	0.553	0.424288858	-5.387	0.005	0.9762812	0.33279923	1	0.35362	1
ALT/AST	chr9:33109998:G:T	8E-33	rs62544382-T	aspartate aminotransferase levels	G	T	-0.045	0.956	0.467785006	-0.058	0.943	0.5247443	0.33290934	1	0.57318	1

© 2022 Yang W et al. *JAMA Network Open*.

ALT/AST	chr6:136024992:C:T	0.00000003	rs17835714-?	aspartate aminotransferase levels	C	T	-0.331	0.718	0.056769008	0.234	1.264	0.2660203	0.33310887	1	0.57643	1
ALT/AST	chr3:142940519:A:G	3E-11	rs6440123-A	alanine aminotransferase levels	A	G	0.051	1.052	0.442473558	0.048	1.049	0.6257009	0.3335867	1	0.52533	1

ALT/AST	chr5:73055235:C:G	9E-37	rs1200503-C	aspartate aminotransferase levels	C	G	-0.080	0.923	0.180543786	0.069	1.071	0.4470095	0.33693368	1	0.15938	1
ALT/AST	chr16:58730951:C:A	2E-12	rs73550818-?	aspartate aminotransferase levels	C	A	-0.076	0.927	0.424566687	-0.033	0.967	0.8116159	0.33707828	1	0.87741	1
ALT/AST	chr13:110367115:A:C	0.000000002	rs4773169-A	alanine aminotransferase levels	A	C	-0.039	0.962	0.521648487	-0.034	0.966	0.7066383	0.33822679	1	0.59689	1
ALT/AST	chr5:96898508:G:A	8E-12	rs3096168-G	aspartate aminotransferase levels	G	A	0.049	1.050	0.416443793	0.024	1.025	0.7856741	0.33933644	1	0.87219	1
ALT/AST	chr2:112013193:A:G	0.000000001	rs4374383-A	hepatitis c induced liver fibrosis	A	G	-0.071	0.931	0.244721317	0.026	1.027	0.7700579	0.33957807	1	0.04161	1
ALT/AST	chr2:233570731:G:A	2E-105	rs12993249-G	bilirubin levels	G	A	-0.073	0.929	0.217119446	0.042	1.043	0.644235	0.34217498	1	0.92497	1
ALT/AST	chr12:21028292:C:A	3E-34	rs1604542-?	total bilirubin levels	C	A	0.017	1.017	0.784331199	0.104	1.109	0.2734572	0.34547547	1	0.58830	1
ALT/AST	chr22:37066896:A:G	0.00000005	rs855791-?	total bilirubin levels	A	G	-0.110	0.896	0.06759432	0.123	1.131	0.1785958	0.35064852	1	0.72004	1
ALT/AST	chr2:112027660:T:C	9E-10	rs6541998-T	aspartate aminotransferase levels	T	C	-0.071	0.931	0.246873223	0.025	1.025	0.7871294	0.35263041	1	0.03052	1
ALT/AST	chr10:99397681:G:C	9E-10	rs76850691-C	aspartate aminotransferase levels;liver enzyme levels (aspartate transaminase)	G	C	-0.364	0.695	0.339540029	-0.112	0.894	0.8775496	0.35303523	1	0.76739	1
ALT/AST	chr2:233729143:A:G	1E-10	rs28898617-?	total bilirubin levels	A	G	0.156	1.168	0.852057194	-1.010	0.364	0.281451	0.3534768	1	0.16009	1
ALT/AST	chr22:42127941:G:A	0.000000001	p.R296C	CPIC:CYP2D6	G	A	-0.052	0.949	0.41338568	-0.014	0.986	0.879246	0.3540232	1	0.18709	1
ALT/AST	chr3:141744456:A:C	0.000000009	rs16851720-C	hepatitis c induced liver fibrosis	A	C	-0.005	0.995	0.951781078	-0.140	0.870	0.2164938	0.35410052	1	0.44814	1
ALT/AST	chr19:41012693:T:A	0.000000001	I391N	CPIC:CYP2B6	T	A	0.310	1.364	0.46374653	0.182	1.200	0.7650599	0.35446034	1	0.88402	1

© 2022 Yang W et al. *JAMA Network Open*.

ALT/AST	chr21:44789511:C:G	3E-11	rs11088963-C	alanine aminotransferase levels	C	G	-0.045	0.956	0.472074634	-0.033	0.967	0.7137503	0.35555382	1	0.73569	1
ALT/AST	chr2:191252512:C:T	1E-11	rs13030978-T	liver enzyme levels (gamma-glutamyl transferase)	C	T	-0.004	0.996	0.955942622	-0.136	0.872	0.1663376	0.3587446	1	0.00462	0.876923

ALT/AST	chr1:94202414:T:C	2E-13	rs1541098-T	aspartate aminotransferase levels	T	C	0.036	1.036	0.60934942	0.059	1.061	0.569268	0.35920393	1	0.15701	1
ALT/AST	chr18:58463061:A:C	9E-15	rs7243073-A	aspartate aminotransferase levels	A	C	0.072	1.075	0.2886689	-0.019	0.981	0.8463478	0.36001502	1	0.09472	1
ALT/AST	chr9:133266456:T:C	5E-44	rs2519093-C	aspartate aminotransferase levels	T	C	0.014	1.014	0.863286872	0.114	1.121	0.3250241	0.36104081	1	0.87481	1
ALT/AST	chr19:7827201:A:G	0.00000005	rs12979658-G	alanine aminotransferase levels	A	G	0.080	1.084	0.237549446	-0.017	0.984	0.8721118	0.36205122	1	0.10412	1
ALT/AST	chr12:120987058:A:G	7E-45	rs7310409-G	liver enzyme levels (gamma-glutamyl transferase)	A	G	-0.028	0.973	0.649368465	-0.061	0.941	0.5067675	0.36399123	1	0.64140	1
ALT/AST	chr1:205075211:A:G	1E-17	rs11240351-G	aspartate aminotransferase levels	A	G	-0.047	0.955	0.455908108	-0.054	0.948	0.5581529	0.36468738	1	0.27164	1
ALT/AST	chr11:72786417:C:T	5E-12	rs481206-T	alanine aminotransferase levels	C	T	0.015	1.015	0.810387588	0.118	1.125	0.2301865	0.36547162	1	0.77120	1
ALT/AST	chr16:252162:A:G	2E-12	rs151330263-	bilirubin levels	A	G	0.611	1.842	0.275365142	-0.029	0.971	0.9646285	0.36614088	1	0.02465	1
ALT/AST	chr10:88047609:T:C	0.000000006	rs10887777-C	alanine aminotransferase levels	T	C	0.069	1.071	0.310743242	-0.032	0.968	0.7476069	0.36620736	1	0.90618	1
ALT/AST	chr10:94842866:A:G	0.000000001	I331V	CPIC:CYP2C19	A	G	0.002	1.002	0.990071695	0.310	1.364	0.1299865	0.36678501	1	0.81914	1
ALT/AST	chr9:109315250:T:C	1E-10	rs68082079-T	alanine aminotransferase levels	T	C	0.126	1.135	0.062601708	-0.112	0.894	0.2603906	0.36702111	1	0.30154	1

© 2022 Yang W et al. *JAMA Network Open*.

ALT/AST	chr22:42129132:C:T	0.000000001	p.V136M	CPIC:CYP2D6	C	T	-0.519	0.595	0.223128665	0.067	1.069	0.8667837	0.36731779	1	0.28074	1
ALT/AST	chr6:32433162:G:C	7E-19	rs3129859-C	acute-on-chronic liver failure in hepatitis b	G	C	-0.064	0.938	0.335779793	0.002	1.002	0.9869761	0.36734714	1	0.97569	1
ALT/AST	chr1:97082391:T:A	0.000000001	p.D949V	CPIC:DPYD	T	A	-0.423	0.655	0.406731457	-0.080	0.924	0.9085941	0.36863716	1	0.82727	1
ALT/AST	chrX:154536002:C:T	0.000000001	p.V68M	CPIC:G6PD	C	T	0.211	1.235	0.418601831	0.187	1.205	0.4854983	0.36915261	1	0.59147	1
ALT/AST	chr10:94762706:A:G	0.000000001	M1V	CPIC:CYP2C19	A	G	-0.629	0.533	0.341026034	0.079	1.083	0.9310821	0.3692298	1	0.38062	1
ALT/AST	chr2:11641492:T:C	0.00000002	rs149940960-	alanine aminotransferase (alt) levels after remission induction therapy in actute lymphoblastic leukemia (all)	T	C	0.051	1.052	0.921206009	-14.178	0.000	0.9730437	0.36994786	1	0.93700	1
ALT/AST	chr1:88680551:C:A	4E-11	rs12145922-A	liver enzyme levels (gamma-glutamyl transferase)	C	A	-0.034	0.966	0.568384799	-0.047	0.954	0.5977108	0.37096352	1	0.14290	1
ALT/AST	chr2:168854097:G:A	5E-18	rs11678685-A	aspartate aminotransferase levels	G	A	-0.084	0.919	0.210838517	0.060	1.061	0.5515029	0.37196775	1	0.93708	1
ALT/AST	chr8:8804171:C:G	1E-23	rs12544992-G	aspartate aminotransferase levels	C	G	-0.076	0.927	0.230578958	-0.002	0.998	0.9862433	0.37417658	1	0.41947	1
ALT/AST	chr19:35547488:A:G	6E-12	rs7599-A	alanine aminotransferase levels	A	G	-0.008	0.992	0.890234219	-0.106	0.899	0.2330884	0.37662632	1	0.94497	1
ALT/AST	chr5:39424526:A:C	0.000000007	rs1818782-?	aspartate aminotransferase levels	A	C	-0.061	0.941	0.325449512	0.002	1.002	0.9860336	0.37774767	1	0.04384	1
ALT/AST	chr2:36922355:T:C	0.00000004	rs11124554-?	alanine transaminase levels	T	C	0.046	1.048	0.435688381	0.044	1.045	0.6313468	0.37956401	1	0.20684	1
ALT/AST	chr19:19434290:G:A	0.00000002	rs4808199-A	nonalcoholic fatty liver disease	G	A	0.025	1.025	0.725157267	0.066	1.069	0.5369968	0.38284809	1	0.87856	1

© 2022 Yang W et al. *JAMA Network Open*.

ALT/AST	chr5:65395057:T:C	0.000000005	rs76622665-?	aspartate aminotransferase levels in low alcohol consumption	T	C	-0.269	0.764	0.355721219	-0.029	0.971	0.9365844	0.38348485	1	0.87829	1
ALT/AST	chr2:233356202:C:G	3E-19	rs1550532-?	bilirubin levels	C	G	-0.088	0.916	0.17042366	0.092	1.096	0.3566347	0.38534262	1	0.89201	1
ALT/AST	chr1:150320113:C:T	0.00000002	rs11581793-?	alanine aminotransferase levels	C	T	-0.078	0.925	0.798389023	-0.530	0.589	0.3314842	0.38924652	1	0.88794	1
ALT/AST	chr7:56022948:T:A	3E-12	rs6973917-T	alanine aminotransferase levels	T	A	-0.053	0.948	0.439086328	-0.028	0.972	0.7866138	0.39343787	1	0.01246	1
ALT/AST	chr12:21196951:A:G	0.000000001	I245V	CPIC:SLCO1B1	A	G	0.075	1.077	0.888785505	-0.944	0.389	0.2138319	0.39574935	1	0.73920	1
ALT/AST	chr10:94981296:A:C	0.000000001	p.I359L	CPIC:CYP2C9	A	C	0.004	1.004	0.973462156	-0.304	0.738	0.1310517	0.39576199	1	0.83106	1
ALT/AST	chr1:97699535:T:C	0.000000001	p.M166V	CPIC:DPYD	T	C	-0.005	0.995	0.967363414	0.208	1.231	0.2038826	0.39916951	1	0.97670	1
ALT/AST	chr12:124824136:G:A	4E-11	rs10846742-?	total bilirubin levels	G	A	0.005	1.005	0.945123277	0.139	1.150	0.207266	0.3993137	1	0.36713	1
ALT/AST	chr10:17833858:T:G	6E-49	rs56278466-?	aspartate transaminase levels in high alcohol intake;aspartate aminotransferase platelet ratio index in high alcohol intake	T	G	0.069	1.071	0.271394241	-0.013	0.987	0.8913932	0.39967136	1	0.96282	1
ALT/AST	chr1:21495264:A:G	7E-15	rs1780324-T	liver enzyme levels	A	G	-0.089	0.915	0.138815565	0.060	1.062	0.5005515	0.39988889	1	0.46446	1
ALT/AST	chr14:77043529:G:A	0.000000003	rs11159247-G	aspartate aminotransferase levels	G	A	-0.040	0.961	0.535640663	-0.047	0.954	0.6255436	0.39994101	1	0.60523	1
ALT/AST	chr2:187346716:C:T	7E-10	rs10931283-T	aspartate aminotransferase levels	C	T	0.038	1.039	0.53479402	0.051	1.052	0.5772916	0.40197778	1	0.74829	1
ALT/AST	chr16:72120610:C:T	3E-16	rs4788460-T	alanine aminotransferase levels	C	T	0.007	1.007	0.923253074	0.109	1.115	0.2824048	0.40567055	1	0.72069	1
ALT/AST	chr22:42128181:A:T	0.000000001	p.M279K	CPIC:CYP2D6	A	T	0.575	1.778	0.563930328	0.419	1.520	0.780386	0.40605674	1	0.04368	1
ALT/AST	chr10:114026477:G:A	0.000000004	rs72823014-G	aspartate aminotransferase levels	G	A	0.106	1.112	0.281270014	-0.059	0.943	0.670815	0.40683386	1	0.20393	1

© 2022 Yang W et al. *JAMA Network Open*.

ALT/AST	chr15:72282873:A:T	0.00000005	rs8027647-T	alanine aminotransferase levels	A	T	-0.044	0.957	0.500069851	-0.016	0.984	0.8667758	0.40711185	1	0.61132	1
ALT/AST	chr7:35515178:C:T	0.00000003	rs343064-A	non-alcoholic fatty liver disease histology (other)	C	T	0.031	1.031	0.617222293	0.031	1.031	0.7409377	0.41423416	1	0.37943	1
ALT/AST	chr8:23016020:G:A	3E-12	rs876435-G	aspartate aminotransferase levels	G	A	0.028	1.029	0.638458893	0.068	1.070	0.465861	0.41750027	1	0.25102	1
ALT/AST	chr10:94775489:G:A	0.000000001	R144H	CPIC:CYP2C19	G	A	0.054	1.056	0.961444414	-0.863	0.422	0.3628582	0.41801034	1	0.08341	1
ALT/AST	chr22:42127608:C:T	0.000000001	p.V338M	CPIC:CYP2D6	C	T	-0.451	0.637	0.314654582	0.022	1.022	0.9569054	0.42005373	1	0.34277	1
ALT/AST	chr8:22059711:T:C	3E-12	rs73545546-C	alanine aminotransferase levels	T	C	0.030	1.030	0.696375251	0.065	1.067	0.5808576	0.42022527	1	0.37267	1
ALT/AST	chr17:7282460:C:A	2E-12	rs117643180-	alanine aminotransferase levels;aspartate aminotransferase levels	C	A	0.045	1.046	0.85828354	0.253	1.288	0.4042151	0.42177599	1	0.09044	1
ALT/AST	chr20:12992873:A:T	2E-14	rs686548-A	alanine aminotransferase levels	A	T	-0.049	0.952	0.423332377	-0.009	0.991	0.9237332	0.42302378	1	0.97898	1
ALT/AST	chr15:58431280:T:C	2E-10	rs1077834-T	alanine aminotransferase levels	T	C	0.053	1.055	0.43441993	-0.022	0.978	0.822826	0.42566091	1	0.77116	1
ALT/AST	chr22:42129770:G:A	0.000000001	p.T107I; p.T10	CPIC:CYP2D6	G	A	0.458	1.582	0.070255066	-0.396	0.673	0.2521811	0.42601363	1	0.04239	1
ALT/AST	chr11:2987174:G:A	2E-11	rs12806061-G	aspartate aminotransferase levels	G	A	-0.017	0.983	0.796286847	0.145	1.156	0.1461935	0.42733193	1	0.72157	1
ALT/AST	chr3:33056563:G:A	8E-12	rs11706136-A	aspartate aminotransferase levels	G	A	-0.060	0.942	0.33743267	0.010	1.010	0.9131639	0.42813383	1	0.65666	1
ALT/AST	chr7:106687688:G:T	0.00000003	rs342221-?	aspartate aminotransferase platelet ratio index in high alcohol intake	G	T	-0.050	0.951	0.410545081	-0.015	0.985	0.8678449	0.43162286	1	0.77960	1

© 2022 Yang W et al. *JAMA Network Open*.

ALT/AST	chr13:48045806:G:A	0.000000001	3' UTR	CPIC:NUDT15	G	A	-0.071	0.932	0.512338593	-0.101	0.904	0.567333	0.43201865	1	0.09732	1
ALT/AST	chr12:25255530:A:G	0.00000003	rs12320328-A	alanine aminotransferase levels	A	G	-0.103	0.902	0.374984255	0.045	1.046	0.7749058	0.43314734	1	0.51997	1
ALT/AST	chr3:46130331:C:A	0.000000003	rs75182702-A	aspartate aminotransferase levels	C	A	0.133	1.142	0.249511484	-0.050	0.952	0.7656404	0.43423272	1	0.64967	1
ALT/AST	chr10:45443298:C:T	0.00000001	rs1051713-C	alanine aminotransferase levels	C	T	-0.032	0.969	0.69080811	-0.082	0.922	0.4991711	0.43630651	1	0.89009	1
ALT/AST	chr10:94762760:A:C	0.000000001	I19L	CPIC:CYP2C19	A	C	0.012	1.013	0.989543697	0.911	2.486	0.3259729	0.43715318	1	0.63626	1
ALT/AST	chr1:111194561:C:T	0.00000002	rs660173-C	aspartate aminotransferase levels	C	T	0.070	1.072	0.3069864	-0.033	0.968	0.7415043	0.44017658	1	0.70211	1
ALT/AST	chr3:170355551:A:C	0.000000001	rs12489967-C	aspartate aminotransferase levels	A	C	0.021	1.021	0.784450544	0.106	1.111	0.3598174	0.44140005	1	0.95792	1
ALT/AST	chr16:86063872:C:T	0.00000001	rs56360131-T	aspartate aminotransferase levels	C	T	-0.052	0.950	0.511186464	-0.021	0.979	0.8479451	0.44406041	1	0.44296	1
ALT/AST	chr22:42128945:C:T	0.000000001	Splicing defect	CPIC:CYP2D6	C	T	0.085	1.089	0.304334282	-0.052	0.950	0.6901109	0.44774509	1	0.08575	1
ALT/AST	chr15:58431740:G:A	1E-11	rs2070895-G	aspartate aminotransferase levels	G	A	0.050	1.052	0.45958794	-0.022	0.978	0.822826	0.44781718	1	0.78278	1
ALT/AST	chr14:103393869:G:A	1E-10	rs12889639-A	aspartate aminotransferase levels	G	A	0.079	1.082	0.210779501	-0.091	0.913	0.3445996	0.44845374	1	0.56934	1
ALT/AST	chr4:48041928:G:A	0.00000001	rs13145218-A	aspartate aminotransferase levels	G	A	0.031	1.031	0.673854562	-0.208	0.812	0.0652448	0.45098308	1	0.93774	1
ALT/AST	chr19:40991367:A:T	0.000000001	Q21L	CPIC:CYP2B6	A	T	-0.014	0.987	0.976319394	1.118	3.060	0.1161441	0.45187386	1	0.44811	1
ALT/AST	chr22:42126914:C:T	0.000000001	p.E418K	CPIC:CYP2D6	C	T	-0.339	0.712	0.75125877	-11.889	0.000	0.9680839	0.45217649	1	0.39499	1
ALT/AST	chr15:73689995:C:T	2E-16	rs8041181-T	aspartate aminotransferase levels	C	T	0.009	1.009	0.885666985	-0.144	0.866	0.1196709	0.45322206	1	0.71990	1

© 2022 Yang W et al. *JAMA Network Open*.

ALT/AST	chr2:48047453:A:T	5E-12	rs72816437-T	aspartate aminotransferase levels	A	T	0.000	1.000	0.995760648	0.130	1.139	0.1670469	0.4532725	1	0.12363	1
ALT/AST	chr5:157326276:T:C	5E-15	rs2289850-T	alanine aminotransferase levels	T	C	-0.054	0.947	0.657723196	-0.040	0.961	0.7975766	0.45510606	1	0.05262	1
ALT/AST	chr19:41012316:T:C	0.000000001	I328T	CPIC:CYP2B6	T	C	0.066	1.068	0.883848307	-0.478	0.620	0.3373111	0.45605384	1	0.90436	1
ALT/AST	chr2:233698748:A:C	0.000000005	rs12988520-C	serum bilirubin levels in metabolic syndrome	A	C	0.014	1.014	0.812431096	0.075	1.078	0.3928113	0.45675156	1	0.76250	1
ALT/AST	chr19:41016810:C:T	0.000000001	R487C; R487S	CPIC:CYP2B6	C	T	-0.043	0.958	0.681091278	-0.076	0.926	0.6056094	0.45720808	1	0.35463	1
ALT/AST	chr11:61930432:G:A	0.000000002	rs6591673-G	aspartate aminotransferase levels	G	A	0.006	1.006	0.923590441	0.092	1.097	0.3111802	0.4581321	1	0.66646	1
ALT/AST	chr8:9320758:A:T	2E-10	rs6984305-A	liver enzyme levels (alkaline phosphatase)	A	T	0.084	1.088	0.326187396	-0.016	0.984	0.905789	0.46137094	1	0.54327	1
ALT/AST	chr22:42130761:C:T	0.000000001	p.V11M	CPIC:CYP2D6	C	T	-0.156	0.855	0.266863986	0.117	1.124	0.6118407	0.46324886	1	0.83348	1
ALT/AST	chr12:21178691:C:T	0.000000001	F199=	CPIC:SLCO1B1	C	T	0.032	1.033	0.595107623	0.043	1.044	0.6370032	0.46683181	1	0.25377	1
ALT/AST	chr19:15879621:C:T	0.000000001	V433M	CPIC:CYP4F2	C	T	0.038	1.038	0.578024145	0.025	1.025	0.7991236	0.4688184	1	0.68666	1
ALT/AST	chr1:155154472:T:C	2E-15	rs10908458-T	liver enzyme levels (gamma-glutamyl transferase)	T	C	-0.005	0.995	0.930139293	-0.099	0.906	0.2717569	0.47181691	1	0.97387	1
ALT/AST	chr5:73099362:G:A	8E-10	rs628401-?	aspartate aminotransferase levels	G	A	0.068	1.071	0.254100325	-0.077	0.926	0.3927348	0.47189119	1	0.15823	1
ALT/AST	chr15:73692882:T:C	2E-19	rs2127015-C	alanine aminotransferase levels	T	C	0.030	1.030	0.617169356	-0.177	0.838	0.0461413	0.47206459	1	0.35662	1
ALT/AST	chr5:39555201:G:T	0.000000003	rs2939328-?	aspartate aminotransferase levels	G	T	0.058	1.059	0.341224056	-0.042	0.959	0.6449265	0.47681339	1	0.38186	1
ALT/AST	chr8:6719619:G:T	0.000000008	rs2911980-T	alanine aminotransferase levels	G	T	-0.052	0.949	0.4432821	-0.005	0.995	0.9615195	0.47713112	1	0.52537	1

© 2022 Yang W et al. *JAMA Network Open*.

ALT/AST	chr12:121102728:G:A	6E-12	rs1794898-G	alanine aminotransferase levels	G	A	-0.004	0.996	0.966069893	0.118	1.125	0.3505002	0.47718489	1	0.22141	1
ALT/AST	chr10:94988925:A:G	0.000000001	p.N457S	CPIC:CYP2C9	A	G	-12.622	0.000	0.95592489	0.286	1.331	0.8760947	0.47767444	1	0.32982	1
ALT/AST	chr1:89517625:G:A	0.00000002	rs359935-A	serum bilirubin levels in metabolic syndrome	G	A	-0.021	0.979	0.941190292	-0.369	0.692	0.3857438	0.48203197	1	0.16814	1
ALT/AST	chr19:41009350:C:A	0.000000001	S259R	CPIC:CYP2B6	C	A	0.291	1.337	0.640765182	0.360	1.433	0.6341322	0.4825201	1	0.35528	1
ALT/AST	chr8:144506797:G:C	3E-38	rs147998249-	alanine aminotransferase levels	G	C	0.284	1.328	0.806295725	0.540	1.716	0.5066255	0.48287627	1	0.14785	1
ALT/AST	chr12:21215788:T:C	0.00000005	rs4363657-?	bilirubin levels	T	C	-0.058	0.944	0.470525928	0.002	1.002	0.9853514	0.48442275	1	0.03973	1
ALT/AST	chr6:32338202:G:T	7E-25	rs3129900-?	lumiracoxib-related liver injury	G	T	-0.074	0.929	0.361711234	0.021	1.021	0.8670906	0.48549709	1	0.73512	1
ALT/AST	chr19:5831829:C:T	8E-24	rs17855739-C	aspartate aminotransferase levels	C	T	-0.093	0.911	0.453360478	0.004	1.004	0.9815823	0.48598221	1	0.05010	1
ALT/AST	chr5:140695889:C:T	0.00000002	rs17119056-T	aspartate aminotransferase levels	C	T	0.075	1.078	0.481133504	0.009	1.009	0.9547623	0.48615141	1	0.57523	1
ALT/AST	chr17:70468676:T:A	1E-10	rs717118-T	alanine aminotransferase levels	T	A	-0.060	0.942	0.31597978	0.042	1.043	0.6365545	0.48817411	1	0.82926	1
ALT/AST	chr2:233764076:T:G	9E-11	rs4148324-?	bilirubin levels in tenofovir-treated hiv infection	T	G	0.005	1.005	0.937411079	0.087	1.091	0.3503966	0.4910211	1	0.86887	1
ALT/AST	chr6:54059899:T:C	0.000000003	rs9296736-T	liver enzyme levels (gamma-glutamyl transferase)	T	C	0.056	1.057	0.388245608	0.003	1.003	0.9750516	0.49251015	1	0.32289	1
ALT/AST	chr6:32411959:G:A	1E-10	rs9405098-A	hepatitis c induced liver cirrhosis	G	A	0.216	1.241	0.190337247	-0.101	0.904	0.6561736	0.49260669	1	0.78277	1
ALT/AST	chr1:111141654:A:G	0.000000007	rs1335645-A	liver enzyme levels (gamma-glutamyl transferase)	A	G	-0.042	0.959	0.620418094	-0.076	0.927	0.5809996	0.49689024	1	0.97749	1

© 2022 Yang W et al. *JAMA Network Open*.

ALT/AST	chr18:57461443:G:A	4E-45	rs503581-G	aspartate aminotransferase levels	G	A	-0.023	0.977	0.806989269	-0.090	0.914	0.5349935	0.50079592	1	0.19251	1
ALT/AST	chr2:226248038:C:T	2E-20	rs2943654-T	alanine aminotransferase levels;aspartate aminotransferase levels	C	T	-0.022	0.978	0.742443102	-0.079	0.924	0.4035371	0.5024378	1	0.61154	1
ALT/AST	chr2:233764663:C:T	5E-93	rs4148325-?	bilirubin levels in extreme obesity;bilirubin levels;total bilirubin levels in hiv-1 infection;serum bilirubin levels in metabolic syndrome;bilirubin levels in tenofovir-treated hiv infection	C	T	0.000	1.000	0.995996604	0.096	1.100	0.3078502	0.50253482	1	0.82176	1
ALT/AST	chr2:233759924:C:T	1E-69	rs887829-T	bilirubin levels;total bilirubin levels in hiv-1 infection;bilirubin levels in tenofovir-treated hiv infection	C	T	0.001	1.001	0.991563032	0.095	1.100	0.3099065	0.50351529	1	0.81843	1

ALT/AST	chr2:233759924:C:T	0.000000001	nearGene-5	CPIC:UGT1A1	C	T	0.001	1.001	0.991563032	0.095	1.100	0.3099065	0.50351529	1	0.81843	1
ALT/AST	chr14:94318281:C:T	5E-12	rs61980636-T	alanine aminotransferase levels	C	T	-0.107	0.899	0.127860218	0.122	1.130	0.2725012	0.50847382	1	0.31049	1
ALT/AST	chr7:99665212:C:T	0.000000001	Splicing defect	CPIC:CYP3A5	C	T	-0.057	0.945	0.870873076	-0.184	0.832	0.6662141	0.51086408	1	0.13214	1
ALT/AST	chr1:97549713:G:A	0.000000001	p.N457N	CPIC:DPYD	G	A	-0.205	0.815	0.770287135	-0.334	0.716	0.6626374	0.51338412	1	0.40418	1
ALT/AST	chr22:36149089:A:T	6E-34	rs132642-T	alanine aminotransferase levels;aspartate aminotransferase levels	A	T	0.072	1.074	0.44876336	-0.015	0.985	0.9128698	0.51537761	1	0.68401	1

© 2022 Yang W et al. *JAMA Network Open*.

ALT/AST	chr6:135097880:T:C	0.000000001	rs9399137-?	aspartate aminotransferase levels	T	C	0.046	1.047	0.528803993	0.015	1.015	0.890705	0.51789828	1	0.42514	1
ALT/AST	chr7:7233809:G:A	2E-11	rs1008897-G	aspartate aminotransferase levels	G	A	0.107	1.113	0.101749067	-0.157	0.855	0.1201152	0.5206818	1	0.89569	1
ALT/AST	chr6:95634240:T:C	0.000000003	rs35064159-C	aspartate aminotransferase levels	T	C	0.088	1.092	0.447074378	-0.005	0.995	0.9796929	0.52435964	1	0.51732	1
ALT/AST	chr8:125495147:C:A	4E-20	rs2954038-?	alanine transaminase levels in high alcohol intake	C	A	0.017	1.017	0.80113887	0.081	1.084	0.4390194	0.52475075	1	0.59414	1
ALT/AST	chr2:36863090:T:C	0.00000003	rs2110944-C	alanine aminotransferase levels	T	C	-0.014	0.986	0.811902637	-0.082	0.921	0.3666397	0.52547441	1	0.23413	1
ALT/AST	chr19:39352092:C:T	2E-12	rs773561743-	alanine aminotransferase levels	C	T	1.366	3.918	0.419275591	-10.440	0.000	0.9743547	0.52672262	1	0.17071	1
ALT/AST	chr8:119175470:A:G	0.000000006	rs2468175-G	aspartate aminotransferase levels	A	G	-0.035	0.966	0.581382947	-0.013	0.987	0.8848357	0.52681947	1	0.67009	1
ALT/AST	chr6:116003979:C:T	6E-12	rs3756772-T	alanine aminotransferase levels	C	T	0.054	1.056	0.37214512	-0.019	0.981	0.8293119	0.53194934	1	0.79408	1
ALT/AST	chr2:233740656:G:A	0.000000006	rs17862875-?	bilirubin levels in tenofovir-treated hiv infection	G	A	0.010	1.011	0.875000865	0.067	1.069	0.4884203	0.53241851	1	0.79704	1
ALT/AST	chr14:77051443:G:A	0.00000003	rs75004998-G	alanine aminotransferase levels	G	A	-0.010	0.990	0.877369993	-0.077	0.926	0.4163997	0.53401764	1	0.74506	1
ALT/AST	chr9:133261662:G:A	5E-25	rs687621-G	alanine aminotransferase levels	G	A	-0.071	0.932	0.270777518	0.021	1.021	0.8245434	0.53516227	1	0.49593	1
ALT/AST	chr2:233741916:T:C	3E-10	rs34352510-?	bilirubin levels in tenofovir-treated hiv infection	T	C	0.003	1.003	0.964298643	0.084	1.087	0.3795389	0.53677629	1	0.78105	1

© 2022 Yang W et al. *JAMA Network Open*.

ALT/AST	chr8:18415371:G:A	7E-11	rs1495741-A	liver injury in anti-tuberculosis drug treatment	G	A	-0.012	0.988	0.851342416	-0.068	0.935	0.5062426	0.53740926	1	0.93534	1
ALT/AST	chr20:34545380:A:C	1E-18	rs6088521-C	alanine aminotransferase levels	A	C	-0.022	0.978	0.722529648	-0.013	0.987	0.8857619	0.53776354	1	0.92822	1
ALT/AST	chr6:139522446:T:C	0.000000008	rs636202-T	alanine aminotransferase levels	T	C	0.003	1.003	0.96586445	0.050	1.051	0.5774541	0.53874376	1	0.59468	1
ALT/AST	chr22:17957192:C:T	1E-16	rs1076540-C	liver enzyme levels (gamma-glutamyl transferase)	C	T	-0.043	0.957	0.56356641	0.207	1.230	0.0570641	0.54102975	1	0.31519	1
ALT/AST	chr19:41012465:C:T	0.000000001	R378X	CPIC:CYP2B6	C	T	0.842	2.322	0.645328698	0.356	1.428	0.8457362	0.54137748	1	0.32827	1
ALT/AST	chr9:93130444:T:C	1E-22	rs7018885-T	aspartate aminotransferase levels;alanine aminotransferase levels	T	C	-0.005	0.995	0.937077622	-0.094	0.911	0.3653715	0.54143531	1	0.88488	1
ALT/AST	chr10:100080295:T:C	0.00000003	rs7900372-?	alanine transaminase levels in high alcohol intake	T	C	-0.041	0.960	0.513121655	-0.013	0.987	0.891839	0.54324366	1	0.07752	1
ALT/AST	chr10:96706525:T:A	1E-14	rs61856806-A	alanine aminotransferase levels	T	A	-0.073	0.930	0.390059404	0.047	1.048	0.6957529	0.54508559	1	0.77739	1
ALT/AST	chr6:18139710:G:A	0.000000001	S125L	CPIC:TPMT	G	A	-12.081	0.000	0.957811454	0.094	1.099	0.9439173	0.54755844	1	0.60545	1
ALT/AST	chr6:115996281:T:C	0.000000002	rs3798232-C	aspartate aminotransferase levels	T	C	0.056	1.058	0.357715545	-0.027	0.973	0.7644972	0.54852532	1	0.66259	1
ALT/AST	chr8:125473052:T:A	3E-23	rs2954027-T	aspartate aminotransferase levels	T	A	-0.024	0.976	0.68286944	-0.021	0.980	0.8183641	0.5485712	1	0.58178	1
ALT/AST	chr22:28333779:A:T	0.00000002	rs134489-A	aspartate aminotransferase levels	A	T	-0.050	0.951	0.473125615	0.030	1.031	0.7713708	0.54919887	1	0.67778	1

© 2022 Yang W et al. *JAMA Network Open*.

ALT/AST	chr4:78698045:C:A	0.00000002	rs75759936-?	aspartate aminotransferase levels	C	A	0.062	1.064	0.811657629	0.190	1.210	0.7384694	0.55047325	1	0.74785	1
ALT/AST	chr6:54038354:G:A	4E-10	rs9637973-G	alanine aminotransferase levels	G	A	0.053	1.054	0.412079733	-0.009	0.991	0.926833	0.55122156	1	0.29486	1
ALT/AST	chr5:56792072:C:T	1E-10	rs144983009-	alanine aminotransferase levels;alanine transaminase levels	C	T	0.009	1.009	0.938588531	-0.209	0.811	0.266548	0.55421764	1	0.63040	1
ALT/AST	chr2:233747994:T:C	0.000000006	rs11888459-?	bilirubin levels in tenofovir-treated hiv infection	T	C	-0.011	0.989	0.866978329	0.106	1.112	0.2487348	0.55499249	1	0.57889	1
ALT/AST	chr2:233749231:T:A	0.000000006	rs10178992-?	bilirubin levels in tenofovir-treated hiv infection	T	A	-0.011	0.989	0.866978329	0.106	1.112	0.2487348	0.55499249	1	0.57889	1
ALT/AST	chr2:233749470:C:T	0.000000006	rs7604115-?	bilirubin levels in tenofovir-treated hiv infection	C	T	-0.011	0.989	0.866978329	0.106	1.112	0.2487348	0.55499249	1	0.57889	1
ALT/AST	chr9:114367105:C:G	7E-10	rs2787352-?	alanine transaminase levels in high alcohol intake	C	G	0.013	1.013	0.828714728	0.053	1.054	0.5550887	0.55553186	1	0.75526	1
ALT/AST	chr15:89802996:A:G	2E-10	rs7168849-A	alanine aminotransferase levels	A	G	0.009	1.009	0.909081286	0.039	1.039	0.7302976	0.55673099	1	0.13153	1
ALT/AST	chr19:806256:A:G	0.00000003	rs351978-G	alanine aminotransferase levels	A	G	0.009	1.009	0.888105149	-0.112	0.894	0.2148611	0.55721707	1	0.36300	1
ALT/AST	chr10:63374062:A:G	6E-23	rs7923609-G	liver enzyme levels (alkaline phosphatase)	A	G	0.052	1.053	0.39951277	-0.006	0.994	0.9505043	0.55956901	1	0.04071	1
ALT/AST	chr19:48702915:C:T	8E-10	rs516246-T	liver enzyme levels (gamma-glutamyl transferase)	C	T	0.054	1.056	0.381172055	-0.008	0.992	0.9286055	0.55957251	1	0.47573	1

© 2022 Yang W et al. *JAMA Network Open*.

ALT/AST	chr2:111932403:A:G	0.00000003	rs6755970-A	alanine aminotransferase levels	A	G	-0.054	0.948	0.385452567	0.034	1.035	0.7070559	0.56029104	1	0.00724	0.948718
ALT/AST	chr1:112574591:A:G	6E-10	rs12406530-A	aspartate aminotransferase levels	A	G	-0.091	0.913	0.194730372	0.062	1.064	0.561027	0.56312833	1	0.73455	1
ALT/AST	chr1:247439666:A:G	2E-14	rs4925546-A	alanine aminotransferase levels	A	G	-0.061	0.941	0.319725613	0.040	1.041	0.6687469	0.56403865	1	0.21885	1
ALT/AST	chr2:233764593:G:A	1E-10	rs3771341-?	bilirubin levels in tenofovir-treated hiv infection	G	A	-0.009	0.991	0.895755408	0.099	1.104	0.2985675	0.56513396	1	0.68256	1
ALT/AST	chr2:233688675:A:T	5E-16	rs10173355-?	total bilirubin levels in hiv-1 infection	A	T	-0.078	0.925	0.264023012	0.090	1.094	0.367458	0.56611324	1	0.88887	1
ALT/AST	chr2:169013785:C:A	9E-31	rs2241339-?	alanine aminotransferase levels;alanine transaminase levels;aspartate aminotransferase levels	C	A	0.042	1.043	0.678188507	-0.223	0.800	0.1273772	0.56788636	1	0.58268	1
ALT/AST	chr10:69225511:G:C	8E-27	rs2394529-C	alanine aminotransferase levels;aspartate aminotransferase levels	G	C	-0.034	0.967	0.585192969	-0.014	0.986	0.8752117	0.56936228	1	0.02715	1
ALT/AST	chr2:233600317:C:T	0.000000002	rs2741012-?	total bilirubin levels in hiv-1 infection	C	T	-0.076	0.927	0.291349501	0.078	1.081	0.4466021	0.56978161	1	0.60627	1
ALT/AST	chr9:129804387:G:A	0.00000002	rs7029757-G	alanine aminotransferase levels	G	A	0.149	1.161	0.189161294	-0.171	0.843	0.2877158	0.57101251	1	0.32754	1
ALT/AST	chr2:232648465:C:A	0.000000001	rs2140773-A	liver enzyme levels (gamma-glutamyl transferase)	C	A	-0.032	0.968	0.599159603	-0.009	0.991	0.9192373	0.57161526	1	0.86781	1
ALT/AST	chr2:233385215:C:T	0.00000003	rs4663580-T	bilirubin levels	C	T	0.044	1.045	0.750772776	-0.107	0.899	0.7289915	0.5717265	1	0.05423	1

© 2022 Yang W et al. *JAMA Network Open*.

ALT/AST	chr2:233755414:G:T	0.000000004	rs11673726-?	bilirubin levels in tenofovir-treated hiv infection	G	T	-0.012	0.988	0.84914421	0.106	1.112	0.2514767	0.57321632	1	0.55308	1
ALT/AST	chr6:130024960:G:A	6E-12	rs7740188-A	alanine aminotransferase levels;aspartate aminotransferase levels	G	A	0.015	1.015	0.818920533	-0.117	0.890	0.2520204	0.57415633	1	0.60554	1
ALT/AST	chr2:232655544:A:T	4E-50	rs13395911-T	alanine aminotransferase levels;aspartate aminotransferase levels;alanine transaminase levels;alanine transaminase levels in high alcohol intake	A	T	-0.036	0.964	0.553306244	-0.002	0.998	0.9830023	0.57562623	1	0.91732	1
ALT/AST	chr12:122131417:G:T	5E-25	rs11061602-T	alanine aminotransferase levels	G	T	0.015	1.015	0.808585024	0.053	1.054	0.5557444	0.57681415	1	0.25654	1
ALT/AST	chr6:24441518:G:A	1E-11	rs9467160-A	liver enzyme levels	G	A	-0.084	0.919	0.224376527	0.070	1.073	0.4744602	0.58124653	1	0.81172	1
ALT/AST	chr3:132479151:T:G	3E-13	rs62292950-T	aspartate aminotransferase levels	T	G	-0.055	0.946	0.498549143	0.025	1.025	0.8347276	0.58262134	1	0.04291	1
ALT/AST	chr5:132461855:A:G	1E-12	rs6894249-A	aspartate aminotransferase levels	A	G	0.042	1.043	0.495246867	-0.038	0.963	0.6722835	0.58274023	1	0.37088	1
ALT/AST	chr11:126355981:C:G	2E-14	rs112771035-	aspartate aminotransferase levels	C	G	-0.031	0.969	0.783439147	-0.070	0.933	0.6824383	0.58559712	1	0.62408	1
ALT/AST	chr9:114386047:T:C	1E-16	rs10739409-?	aspartate aminotransferase levels	T	C	-0.064	0.938	0.330822467	0.033	1.034	0.7494339	0.58649028	1	0.28481	1
ALT/AST	chr10:94942290:C:T	0.000000001	p.R144C	CPIC:CYP2C9	C	T	0.021	1.021	0.834987941	0.073	1.076	0.6333556	0.59263167	1	0.55162	1
ALT/AST	chr2:233693545:A:G	2E-14	rs2070959-G	serum bilirubin levels in metabolic syndrome	A	G	-0.074	0.929	0.285418834	0.090	1.094	0.3692658	0.59379672	1	0.88714	1

© 2022 Yang W et al. *JAMA Network Open*.

ALT/AST	chr10:63361805:C:A	5E-10	rs12355784-A	liver enzyme levels	C	A	0.049	1.050	0.430245762	-0.006	0.994	0.9505043	0.59531832	1	0.03617	1
ALT/AST	chr22:50656498:C:T	2E-14	rs9616810-T	aspartate aminotransferase levels	C	T	0.009	1.009	0.903545621	0.058	1.060	0.596365	0.59590593	1	0.84404	1
ALT/AST	chr2:233736540:G:T	9E-18	rs7564935-G	bilirubin levels	G	T	-0.012	0.988	0.849709155	0.102	1.108	0.2685215	0.59618736	1	0.62145	1
ALT/AST	chr21:39183635:T:C	6E-11	rs117143374-	alanine aminotransferase levels	T	C	0.073	1.076	0.439595828	-0.037	0.964	0.7823673	0.59671503	1	0.45642	1
ALT/AST	chr3:11354347:C:G	0.00000002	rs2594973-C	aspartate aminotransferase levels	C	G	-0.055	0.947	0.370196963	0.051	1.052	0.5652213	0.59809459	1	0.51578	1
ALT/AST	chr8:10713981:C:A	2E-19	rs4484649-C	alanine aminotransferase levels	C	A	-0.002	0.998	0.97056411	0.095	1.100	0.2846433	0.60062204	1	0.18417	1
ALT/AST	chr19:40991381:A:T	0.000000001	T26S	CPIC:CYP2B6	A	T	-0.639	0.528	0.471283526	0.174	1.190	0.841085	0.60111531	1	0.22413	1
ALT/AST	chr19:40991388:A:G	0.000000001	D28G	CPIC:CYP2B6	A	G	-0.639	0.528	0.471283526	0.174	1.190	0.841085	0.60111531	1	0.22413	1
ALT/AST	chr19:40991390:C:A	0.000000001	p.R29S	CPIC:CYP2B6	C	A	-0.639	0.528	0.471283526	0.174	1.190	0.841085	0.60111531	1	0.22413	1
ALT/AST	chr19:40991391:G:C	0.000000001	p.R29P	CPIC:CYP2B6	G	C	-0.639	0.528	0.471283526	0.174	1.190	0.841085	0.60111531	1	0.22413	1
ALT/AST	chr7:80545045:T:C	3E-14	rs139761834-	aspartate aminotransferase levels	T	C	0.234	1.263	0.826785031	-13.428	0.000	0.9685109	0.60387177	1	0.23221	1
ALT/AST	chr19:55312964:T:G	0.00000003	rs7246479-G	aspartate aminotransferase levels	T	G	-0.010	0.990	0.869133345	-0.064	0.938	0.4984203	0.60563089	1	0.27565	1
ALT/AST	chr19:41004125:G:A	0.000000001	G99E	CPIC:CYP2B6	G	A	0.281	1.325	0.877076704	-12.938	0.000	0.9721622	0.60933857	1	0.13985	1
ALT/AST	chr2:233744071:C:T	0.000000005	rs17864701-?	bilirubin levels in tenofovir-treated hiv infection	C	T	0.010	1.010	0.877458136	0.049	1.050	0.6085591	0.61198255	1	0.75619	1
ALT/AST	chr2:233750415:A:C	0.000000002	rs2885296-?	bilirubin levels in tenofovir-treated hiv infection	A	C	0.004	1.004	0.949117074	0.061	1.063	0.5257616	0.61275997	1	0.72786	1
ALT/AST	chr17:17280660:A:C	0.00000002	rs117743490-	alanine aminotransferase levels	A	C	-0.043	0.958	0.793344011	-0.073	0.930	0.7420978	0.61421516	1	0.48893	1
ALT/AST	chr1:97573863:C:T	0.000000001	p.E412E	CPIC:DPYD	C	T	-0.073	0.930	0.772582465	-0.178	0.837	0.7302745	0.61584226	1	0.83914	1
ALT/AST	chr1:97579893:G:C	0.000000001	NA	CPIC:DPYD	G	C	-0.073	0.930	0.772582465	-0.178	0.837	0.7302745	0.61584226	1	0.83914	1

© 2022 Yang W et al. *JAMA Network Open*.

ALT/AST	chr12:70719307:T:C	4E-27	rs2137537-T	aspartate aminotransferase levels	T	C	0.064	1.066	0.292085368	-0.047	0.954	0.5932269	0.61636506	1	0.70988	1
ALT/AST	chr2:233609365:G:A	0.000000001	rs2741027-A	serum bilirubin levels in metabolic syndrome	G	A	-0.061	0.941	0.39821923	0.061	1.063	0.5580036	0.61956609	1	0.55941	1
ALT/AST	chr5:73110201:A:G	3E-11	rs554327-?	aspartate aminotransferase levels	A	G	0.055	1.057	0.3549516	-0.085	0.919	0.3485224	0.62115215	1	0.07596	1
ALT/AST	chr5:31020414:A:G	0.000000001	rs6888304-A	liver enzyme levels (gamma-glutamyl transferase)	A	G	0.136	1.146	0.052973081	-0.195	0.823	0.0592412	0.6225111	1	0.16164	1
ALT/AST	chr19:10236408:T:C	2E-10	rs8108722-?	alanine aminotransferase levels;alanine transaminase levels	T	C	-0.003	0.997	0.963305837	0.104	1.110	0.3430438	0.62378204	1	0.57544	1
ALT/AST	chr7:116777794:G:C	0.000000001	rs11770163-C	alanine aminotransferase levels	G	C	0.007	1.007	0.918941069	-0.076	0.927	0.4198809	0.62596567	1	0.78504	1
ALT/AST	chr11:94180526:T:C	6E-18	rs74913549-?	alanine transaminase levels;aspartate aminotransferase levels	T	C	0.059	1.060	0.567294311	-0.013	0.987	0.9330949	0.62617074	1	0.99943	1
ALT/AST	chr2:233720033:T:G	0	rs13401281-?	total bilirubin levels	T	G	-0.010	0.990	0.879894542	0.097	1.102	0.2940914	0.6288948	1	0.48537	1
ALT/AST	chr11:16228637:C:T	1E-10	rs1401454-C	aspartate aminotransferase levels	C	T	-0.028	0.972	0.642410529	-0.019	0.981	0.8322286	0.62940477	1	0.02020	1
ALT/AST	chr17:78406035:G:A	7E-13	rs2376584-A	aspartate aminotransferase levels	G	A	0.053	1.054	0.376219697	-0.074	0.929	0.4379416	0.63023763	1	0.25841	1
ALT/AST	chr15:73685996:C:T	0.000000001	rs8038465-T	liver enzyme levels (gamma-glutamyl transferase)	C	T	0.014	1.014	0.823236037	-0.124	0.883	0.1980275	0.63212199	1	0.73595	1
ALT/AST	chr4:154468095:T:C	6E-11	rs17031671-?	bilirubin levels	T	C	0.519	1.680	0.216788302	-1.636	0.195	0.0455283	0.63335354	1	0.15145	1
ALT/AST	chr18:66962261:A:G	0.000000007	rs116561224-	drug-induced liver injury (statins)	A	G	-0.149	0.861	0.363316957	0.118	1.125	0.5649055	0.63344184	1	0.40579	1

© 2022 Yang W et al. *JAMA Network Open*.

ALT/AST	chr6:29860883:A:G	2E-10	rs2523822-?	drug-induced liver injury (amoxicillin-clavulanate)	A	G	-0.042	0.959	0.527848665	0.150	1.161	0.1236057	0.63522715	1	0.87780	1
ALT/AST	chr7:50218883:A:T	0.00000003	rs4598207-?	aspartate aminotransferase levels	A	T	-0.043	0.958	0.496511567	-0.007	0.993	0.9431844	0.63547169	1	0.18357	1
ALT/AST	chr22:28712241:A:G	7E-18	rs5752776-G	alanine aminotransferase levels	A	G	0.031	1.031	0.635958866	0.006	1.006	0.9501505	0.63685709	1	0.69397	1
ALT/AST	chr22:29381515:C:T	5E-15	rs11704551-C	aspartate aminotransferase levels	C	T	0.013	1.013	0.859615215	0.061	1.063	0.5622117	0.63692351	1	0.35792	1
ALT/AST	chr2:233745803:A:G	0.000000003	rs11695484-?	bilirubin levels in tenofovir-treated hiv infection	A	G	0.009	1.009	0.898250145	0.047	1.048	0.6251294	0.63734408	1	0.75446	1
ALT/AST	chr2:233702448:T:G	1E-22	rs17863787-?	total bilirubin levels in hiv-1 infection;serum bilirubin levels in metabolic syndrome	T	G	-0.018	0.982	0.79166331	0.111	1.118	0.2569452	0.63757041	1	0.88167	1
ALT/AST	chr4:145873469:T:C	3E-27	rs4547811-C	liver enzyme levels (gamma-glutamyl transferase)	T	C	0.012	1.012	0.861526438	0.005	1.005	0.9635152	0.63806247	1	0.06486	1
ALT/AST	chr11:47893747:A:G	2E-15	rs34467936-A	aspartate aminotransferase levels	A	G	-0.010	0.990	0.873011752	-0.048	0.953	0.6098576	0.64000112	1	0.56281	1
ALT/AST	chr9:101460951:C:G	0.000000001	rs10819937-C	liver enzyme levels (alkaline phosphatase)	C	G	0.001	1.001	0.986621666	0.108	1.114	0.3230544	0.64039679	1	0.39463	1
ALT/AST	chr16:31096368:C:T	0.000000001	5' region	CPIC:VKORC1	C	T	0.002	1.002	0.971918637	0.045	1.046	0.6302751	0.64099842	1	0.60887	1
ALT/AST	chr4:102789773:G:A	0.000000006	rs223454-G	alanine aminotransferase levels	G	A	0.006	1.006	0.921811496	-0.078	0.925	0.3687556	0.64161224	1	0.37486	1
ALT/AST	chr10:63515167:A:G	7E-10	rs10761779-G	liver enzyme levels	A	G	0.046	1.047	0.452160861	-0.013	0.987	0.8874631	0.64525499	1	0.04624	1

© 2022 Yang W et al. *JAMA Network Open*.

ALT/AST	chr3:171014810:G:A	0.000000002	rs7356034-A	alanine aminotransferase levels	G	A	0.046	1.048	0.477659126	-0.169	0.844	0.0831139	0.64544811	1	0.82942	1
ALT/AST	chr6:32223562:C:T	3E-16	rs115695709-	aspartate aminotransferase levels	C	T	0.031	1.032	0.7378501	0.023	1.023	0.869603	0.64806302	1	0.79527	1
ALT/AST	chr10:98480617:C:T	3E-10	rs72838936-?	aspartate aminotransferase levels	C	T	0.057	1.059	0.530857519	-0.043	0.958	0.7560951	0.64855726	1	0.53784	1
ALT/AST	chr22:42130692:G:A	0.000000001	p.P34S	CPIC:CYP2D6	G	A	-0.005	0.995	0.946876518	-0.064	0.938	0.5796309	0.65242983	1	0.13601	1
ALT/AST	chr12:120878449:A:G	1E-15	rs656933-A	aspartate aminotransferase levels	A	G	0.003	1.003	0.96397719	-0.053	0.948	0.5928709	0.6534952	1	0.76651	1
ALT/AST	chr6:31464003:T:G	6E-97	rs2395029-?	drug-induced liver injury (flucloxacillin)	T	G	0.150	1.161	0.415340381	-0.099	0.906	0.713278	0.65368385	1	0.83916	1
ALT/AST	chr2:233772999:G:C	4E-20	rs8330-?	total bilirubin levels	G	C	0.024	1.024	0.733924085	0.018	1.018	0.8631786	0.65474071	1	0.55971	1
ALT/AST	chr11:16227964:A:G	6E-10	rs10832570-A	alanine aminotransferase levels	A	G	-0.026	0.975	0.671035029	-0.019	0.981	0.8322286	0.65513391	1	0.01866	1
ALT/AST	chr14:103106478:A:G	6E-29	rs944002-G	liver enzyme levels (gamma-glutamyl transferase)	A	G	-0.014	0.986	0.843617401	-0.031	0.969	0.7616775	0.65555768	1	0.36138	1
ALT/AST	chr10:77900367:T:C	5E-27	rs10430531-C	alanine aminotransferase levels	T	C	0.020	1.021	0.773138341	0.024	1.024	0.8199432	0.65635909	1	0.39176	1
ALT/AST	chr4:145900258:C:A	2E-62	rs4835265-A	alanine aminotransferase levels;aspartate aminotransferase levels;alanine transaminase levels	C	A	0.020	1.021	0.77879332	-0.021	0.979	0.8599075	0.65654201	1	0.09889	1
ALT/AST	chr2:232639279:G:A	0.000000006	rs10165093-?	aspartate aminotransferase levels	G	A	-0.032	0.969	0.60621745	0.134	1.143	0.1322059	0.65758412	1	0.57892	1
ALT/AST	chr1:97573881:C:T	0.000000001	p.M406I	CPIC:DPYD	C	T	-0.493	0.611	0.28332064	0.821	2.272	0.0679724	0.65903353	1	0.96800	1

© 2022 Yang W et al. *JAMA Network Open*.

ALT/AST	chr2:100962027:G:A	0.000000005	rs6747874-A	aspartate aminotransferase levels	G	A	-0.031	0.970	0.647264692	-0.030	0.970	0.7636854	0.66282286	1	0.93747	1
ALT/AST	chr2:233730664:G:A	1E-41	rs11891311-?	bilirubin levels	G	A	-0.017	0.984	0.796377627	0.104	1.110	0.261556	0.66417644	1	0.65646	1
ALT/AST	chr12:21008244:A:G	3E-18	rs76300959-?	total bilirubin levels	A	G	0.016	1.016	0.801910231	0.045	1.046	0.6364213	0.66418595	1	0.84991	1
ALT/AST	chr16:58037060:T:A	1E-10	rs12928392-T	alanine aminotransferase levels	T	A	-0.018	0.982	0.789499282	-0.030	0.971	0.7669255	0.66519296	1	0.16534	1

ALT/AST	chr2:233755708:G:A	0.000000002	rs6747843-?	bilirubin levels in tenofovir-treated hiv infection	G	A	-0.005	0.995	0.94123246	0.069	1.071	0.4742571	0.6671998	1	0.63484	1
ALT/AST	chr19:54255708:C:T	3E-11	rs380731-T	aspartate aminotransferase levels	C	T	0.051	1.052	0.482451942	-0.039	0.962	0.7108349	0.66839372	1	0.33740	1
ALT/AST	chr10:94775507:G:A	0.000000001	R150H	CPIC:CYP2C19	G	A	-0.276	0.759	0.641571493	0.018	1.019	0.9778065	0.66908231	1	0.31961	1
ALT/AST	chr12:21205999:G:C	0.000000001	G488A	CPIC:SLCO1B1	G	C	0.337	1.401	0.551761672	-1.354	0.258	0.2236481	0.66960607	1	0.27311	1
ALT/AST	chr6:18130762:C:T	0.000000001	R215H	CPIC:TPMT	C	T	0.431	1.539	0.530404698	-0.305	0.737	0.8012734	0.67114312	1	0.28304	1
ALT/AST	chr2:233765830:A:G	3E-24	rs929596-?	total bilirubin levels in hiv-1 infection;serum bilirubin levels in metabolic syndrome;bilirubin levels in tenofovir-treated hiv infection	A	G	-0.008	0.992	0.909437607	0.076	1.079	0.4387174	0.67228161	1	0.46991	1
ALT/AST	chr3:149494481:G:A	3E-15	rs62269283-?	alanine aminotransferase levels;alanine transaminase levels	G	A	0.059	1.060	0.86824786	0.250	1.284	0.5935021	0.67360863	1	0.01234	1
ALT/AST	chr19:41006936:G:T	0.000000001	Q172H	CPIC:CYP2B6	G	T	0.021	1.022	0.745644563	0.012	1.012	0.9024894	0.67413386	1	0.82624	1
ALT/AST	chr16:58736993:T:G	9E-41	rs11643959-T	aspartate aminotransferase levels	T	G	0.072	1.074	0.564609202	-0.021	0.979	0.9062602	0.67511366	1	0.96286	1
ALT/AST	chr22:41830391:C:T	0.00000001	rs5758487-C	alanine aminotransferase levels	C	T	-0.004	0.996	0.943800401	-0.075	0.927	0.3932671	0.67547778	1	0.64994	1

© 2022 Yang W et al. *JAMA Network Open*.

ALT/AST	chr2:233757136:G:A	3E-64	rs10929302-A	bilirubin levels;bilirubin levels in tenofovir-treated hiv infection	G	A	0.000	1.000	0.996638208	0.056	1.057	0.5614499	0.67665782	1	0.65125	1
ALT/AST	chr3:12288912:G:A	0.00000002	rs17036170-?	drug-induced liver injury	G	A	0.010	1.010	0.971687237	-0.286	0.751	0.4945453	0.67736552	1	0.10751	1
ALT/AST	chr11:78229029:G:A	5E-10	rs2450128-G	alanine aminotransferase levels	G	A	0.027	1.028	0.707709371	-0.141	0.869	0.2028505	0.67778826	1	0.45994	1

ALT/AST	chr9:100569123:T:C	4E-12	rs2800290-C	aspartate aminotransferase levels	T	C	-0.079	0.924	0.196414042	0.106	1.111	0.2530698	0.67969688	1	0.34902	1
ALT/AST	chr22:42127852:C:T	0.000000001	Splicing defect	CPIC:CYP2D6	C	T	0.142	1.152	0.793269062	0.156	1.169	0.8346473	0.67970396	1	0.79884	1
ALT/AST	chr2:233756119:T:C	0.000000002	rs6714634-?	bilirubin levels in tenofovir-treated hiv infection	T	C	0.000	1.000	0.996638208	0.054	1.056	0.571559	0.68167325	1	0.65257	1
ALT/AST	chr4:76495474:A:G	1E-22	rs12500824-A	alanine aminotransferase levels;aspartate aminotransferase levels;alanine transaminase levels	A	G	-0.041	0.959	0.498609705	0.036	1.037	0.698265	0.68327103	1	0.72361	1
ALT/AST	chr2:160010548:G:A	2E-13	rs2667011-?	bilirubin levels	G	A	0.007	1.007	0.926018621	-0.101	0.904	0.372751	0.6835033	1	0.06957	1
ALT/AST	chr9:130276442:A:G	0.00000003	rs9657643-A	aspartate aminotransferase levels	A	G	-0.008	0.992	0.915035304	-0.046	0.955	0.662869	0.68488662	1	0.50832	1
ALT/AST	chr6:21939097:G:A	2E-11	rs6938097-G	alanine aminotransferase levels;aspartate aminotransferase levels	G	A	-0.005	0.995	0.946952475	0.087	1.091	0.4128105	0.68528859	1	0.04653	1
ALT/AST	chr12:3231491:C:T	0.00000004	rs7976853-T	aspartate aminotransferase levels	C	T	-0.006	0.994	0.955848748	-0.079	0.924	0.6133722	0.68763086	1	0.43220	1

© 2022 Yang W et al. *JAMA Network Open*.

ALT/AST	chr4:17878793:A:C	0.000000003	rs7700107-C	alanine aminotransferase levels	A	C	0.072	1.074	0.390666488	-0.093	0.912	0.4633897	0.69050016	1	0.48426	1
ALT/AST	chr9:19469848:G:T	0.000000009	rs11790131-?	liver fibrosis severity in hiv/hepatitis c co-infection	G	T	0.054	1.056	0.474521453	-0.042	0.959	0.7138693	0.69057348	1	0.41699	1
ALT/AST	chr13:21776998:T:C	6E-11	rs500802-T	aspartate aminotransferase levels	T	C	-0.046	0.955	0.485557769	0.034	1.035	0.7197893	0.69072192	1	0.76737	1
ALT/AST	chr8:143560999:C:T	3E-10	rs1545536-T	alanine aminotransferase levels	C	T	-0.053	0.949	0.441493719	0.040	1.041	0.6977382	0.69420616	1	0.64752	1

ALT/AST	chr12:6384185:A:G	0.000000001	rs10849448-A	aspartate aminotransferase levels	A	G	0.090	1.094	0.217364319	-0.155	0.857	0.1616306	0.69474407	1	0.91857	1
ALT/AST	chrX:154535277:T:C	0.000000001	p.N126D	CPIC:G6PD	T	C	-0.050	0.952	0.787968891	0.017	1.017	0.9339264	0.69598846	1	0.98360	1
ALT/AST	chr19:15897578:A:C	0.000000001	W12G	CPIC:CYP4F2	A	C	-0.029	0.971	0.72337705	0.149	1.161	0.1947154	0.69796157	1	0.03871	1
ALT/AST	chr16:80464197:T:C	2E-15	rs7189522-T	aspartate aminotransferase levels	T	C	-0.023	0.978	0.739891735	0.091	1.096	0.3496064	0.69953234	1	0.38937	1
ALT/AST	chr9:91218276:T:A	2E-12	rs56049491-?	total bilirubin levels	T	A	-0.049	0.952	0.53654216	0.204	1.226	0.0974125	0.70196683	1	0.99817	1
ALT/AST	chr10:94780574:G:C	0.000000001	R186P	CPIC:CYP2C19	G	C	0.808	2.244	0.561214337	-9.548	0.000	0.9765436	0.70281801	1	0.66473	1
ALT/AST	chr13:110647653:T:C	3E-11	rs421491-T	aspartate aminotransferase levels	T	C	-0.004	0.996	0.97081073	0.099	1.104	0.5418457	0.70451921	1	0.30026	1
ALT/AST	chr12:56639569:C:T	4E-12	rs2950388-C	alanine aminotransferase levels	C	T	0.003	1.003	0.959968977	-0.071	0.931	0.4870762	0.70520505	1	0.89313	1
ALT/AST	chr10:63405424:G:A	3E-11	rs10822168-?	total bilirubin levels	G	A	0.047	1.049	0.441510103	-0.026	0.975	0.7763339	0.70831503	1	0.07861	1
ALT/AST	chr12:20921188:C:T	3E-14	rs2117032-C	bilirubin levels	C	T	0.010	1.010	0.866566071	0.025	1.026	0.7867161	0.7088801	1	0.84450	1
ALT/AST	chr10:94775416:T:C	0.000000001	W120R	CPIC:CYP2C19	T	C	0.157	1.170	0.808671533	0.198	1.219	0.8564045	0.70933552	1	0.35619	1
ALT/AST	chr2:233749337:T:C	0.000000001	rs10179091-?	bilirubin levels in tenofovir-treated hiv infection	T	C	-0.005	0.995	0.939323373	0.060	1.062	0.5017148	0.71010286	1	0.17798	1

© 2022 Yang W et al. *JAMA Network Open*.

ALT/AST	chr2:233755003:C:G	0.000000007	rs10929301-?	bilirubin levels in tenofovir-treated hiv infection	C	G	-0.003	0.997	0.96206578	0.062	1.064	0.4969267	0.71039064	1	0.25415	1
ALT/AST	chr2:233758936:A:C	9E-20	rs3755319-?	total bilirubin levels in hiv-1 infection	A	C	0.000	1.000	0.998436579	0.055	1.056	0.5479459	0.71357949	1	0.25663	1
ALT/AST	chr8:102650624:T:C	6E-17	rs13275089-T	alanine aminotransferase levels	T	C	-0.023	0.978	0.724237892	-0.013	0.987	0.8881905	0.71393336	1	0.02242	1
ALT/AST	chr6:18143724:C:G	0.000000001	A80P	CPIC:TPMT	C	G	0.290	1.337	0.67379803	-0.032	0.969	0.9820259	0.71435145	1	0.75751	1
ALT/AST	chr5:77438259:C:T	0.00000003	rs33204-C	aspartate aminotransferase levels	C	T	-0.002	0.998	0.976654021	0.038	1.039	0.6764984	0.7158285	1	0.56002	1
ALT/AST	chr11:95450772:T:C	0.00000001	rs12799680-T	aspartate aminotransferase levels	T	C	-0.057	0.944	0.577108572	0.031	1.031	0.8343454	0.71629645	1	0.93338	1
ALT/AST	chr1:150770224:T:C	2E-18	rs11204725-C	aspartate aminotransferase levels	T	C	0.028	1.028	0.639920852	-0.011	0.990	0.907723	0.71675423	1	0.79307	1
ALT/AST	chr10:100152437:C:T	1E-16	rs1408579-?	alanine aminotransferase levels	C	T	0.019	1.019	0.766345029	-0.095	0.909	0.3240075	0.71698486	1	0.02692	1
ALT/AST	chr11:61806212:T:C	0.000000006	rs174551-?	alanine transaminase levels	T	C	-0.036	0.965	0.569785629	0.004	1.004	0.9676284	0.71970554	1	0.13953	1
ALT/AST	chr5:56508725:A:C	2E-14	rs40270-C	alanine aminotransferase levels	A	C	-0.089	0.915	0.192964355	0.113	1.120	0.253322	0.7203474	1	0.30588	1
ALT/AST	chr11:1758975:A:G	9E-13	rs11555039-G	aspartate aminotransferase levels	A	G	0.066	1.068	0.559150914	-0.210	0.810	0.2015668	0.72420917	1	0.29399	1
ALT/AST	chr2:233759379:A:G	0.00000005	rs6723506-G	bilirubin levels	A	G	0.079	1.083	0.696874469	-0.836	0.433	0.0675818	0.72467795	1	0.13197	1
ALT/AST	chr14:54771591:G:A	4E-16	rs7155922-G	aspartate aminotransferase levels	G	A	-0.001	0.999	0.982025843	-0.065	0.937	0.4697296	0.72722986	1	0.04370	1
ALT/AST	chr15:72211718:G:A	0.000000004	rs8023703-A	aspartate aminotransferase levels	G	A	0.005	1.005	0.943255276	-0.043	0.958	0.658152	0.730097	1	0.57911	1

© 2022 Yang W et al. *JAMA Network Open*.

ALT/AST	chr10:96700824:G:A	2E-11	rs45587331-?	alanine aminotransferase levels;aspartate aminotransferase levels;alanine transaminase levels	G	A	-0.044	0.957	0.594256113	0.034	1.035	0.7710254	0.7300994	1	0.80327	1
ALT/AST	chr8:125469835:A:G	2E-75	rs2954021-A	alanine aminotransferase levels;liver enzyme levels (alkaline phosphatase);liver enzyme levels (alanine transaminase)	A	G	-0.015	0.985	0.792286311	-0.004	0.996	0.9654153	0.73071498	1	0.90097	1
ALT/AST	chr10:94938683:A:G	0.000000001	p.M1V	CPIC:CYP2C9	A	G	-12.607	0.000	0.955977818	0.478	1.612	0.7273453	0.73142081	1	0.46482	1

ALT/AST	chr9:4763176:T:C	2E-13	rs385893-?	aspartate aminotransferase platelet ratio index in high alcohol intake	T	C	-0.056	0.946	0.36556579	0.074	1.077	0.4145258	0.73357294	1	0.30742	1
ALT/AST	chr16:80463704:C:A	0.000000003	rs4581712-A	liver enzyme levels (gamma-glutamyl transferase)	C	A	0.030	1.030	0.668150656	-0.104	0.901	0.3003188	0.73669287	1	0.33174	1
ALT/AST	chr1:97450068:A:G	0.000000001	p.F632F	CPIC:DPYD	A	G	-0.011	0.989	0.928423456	-0.067	0.935	0.7358554	0.73685237	1	0.32364	1
ALT/AST	chr6:43789345:T:A	4E-14	rs4711750-A	alanine aminotransferase levels	T	A	-0.011	0.989	0.858577781	-0.024	0.977	0.7984561	0.7369245	1	0.99355	1
ALT/AST	chr12:123409363:T:C	2E-15	rs28642812-C	alanine aminotransferase levels	T	C	-0.067	0.935	0.295208011	0.186	1.205	0.055624	0.73756628	1	0.93177	1
ALT/AST	chr1:209942767:G:A	0.00000002	rs2205986-?	drug-induced liver injury in interferon-beta-treated multiple sclerosis	G	A	-0.092	0.912	0.410076432	0.132	1.141	0.456853	0.73882258	1	0.85523	1

© 2022 Yang W et al. *JAMA Network Open*.

ALT/AST	chr10:100152307:T:C	3E-114	rs2862954-T	alanine aminotransferase levels;aspartate aminotransferase levels	T	C	0.019	1.019	0.766345029	-0.091	0.913	0.3459255	0.7400306	1	0.02640	1
ALT/AST	chr12:21176804:A:G	0.000000001	N130D	CPIC:SLCO1B1	A	G	-0.006	0.994	0.925969197	-0.017	0.983	0.8548208	0.7456692	1	0.69349	1
ALT/AST	chr16:72186474:G:C	0.000000005	rs7186908-C	liver enzyme levels (alkaline phosphatase)	G	C	0.025	1.025	0.731347879	-0.085	0.919	0.4317828	0.74667861	1	0.28586	1
ALT/AST	chr9:133263862:A:C	2E-30	rs657152-T	liver enzyme levels	A	C	-0.043	0.958	0.496445844	0.005	1.005	0.9547146	0.74908626	1	0.84023	1
ALT/AST	chr2:168984064:T:G	0.000000002	rs16856332-T	liver enzyme levels (alkaline phosphatase)	T	G	0.012	1.012	0.941299919	-0.184	0.832	0.4511212	0.74928633	1	0.62610	1
ALT/AST	chr11:62432704:C:T	8E-12	rs17145884-C	alanine aminotransferase levels	C	T	0.032	1.032	0.663448438	-0.141	0.869	0.2226141	0.75067596	1	0.23296	1
ALT/AST	chrX:154532439:A:G	0.000000001	p.Y437Y	CPIC:G6PD	A	G	0.015	1.015	0.832693405	0.017	1.017	0.8609845	0.75723754	1	0.75153	1

© 2022 Yang W et al. *JAMA Network Open*.

ALT/AST	chr1:88681169:A:G	1E-20	rs1002436-G	aspartate aminotransferase levels;alanine aminotransferase levels	A	G	-0.010	0.990	0.864457293	-0.017	0.983	0.8509235	0.76257915	1	0.27374	1
ALT/AST	chr1:198665496:T:C	0.000000003	rs1052238-T	aspartate aminotransferase levels	T	C	0.028	1.028	0.645099194	-0.104	0.901	0.2457639	0.76351903	1	0.49956	1
ALT/AST	chr12:111803962:G:A	5E-13	rs671-A	aspartate aminotransferase levels	G	A	-0.022	0.978	0.959683365	-0.145	0.865	0.7720419	0.76415718	1	0.48750	1
ALT/AST	chr10:119711739:C:G	4E-22	rs196210-G	aspartate aminotransferase levels	C	G	0.074	1.077	0.231903513	-0.117	0.890	0.2094225	0.7644392	1	0.54420	1
ALT/AST	chr2:233735091:A:T	5E-16	rs4477910-?	total bilirubin levels in hiv-1 infection	A	T	-0.020	0.980	0.749203343	0.095	1.100	0.3062016	0.76739572	1	0.38924	1
ALT/AST	chr19:33420799:T:C	2E-16	rs57457691-T	aspartate aminotransferase levels	T	C	0.052	1.054	0.38394744	-0.069	0.933	0.4430587	0.76772166	1	0.51094	1
ALT/AST	chr6:36680587:T:C	0.00000001	rs3176334-T	alanine aminotransferase levels	T	C	0.011	1.011	0.857994859	0.033	1.034	0.7304636	0.768655	1	0.04744	1
ALT/AST	chr17:37713312:C:A	2E-15	rs17138478-C	alanine aminotransferase levels;aspartate aminotransferase levels	C	A	0.057	1.059	0.534525112	-0.112	0.894	0.4034257	0.76901004	1	0.90533	1
ALT/AST	chr6:32347950:T:C	9E-11	rs910049-A	hepatitis c induced liver cirrhosis	T	C	-0.058	0.943	0.398090546	0.091	1.095	0.3917222	0.77042514	1	0.55494	1
ALT/AST	chr9:114371911:C:T	2E-18	rs7043196-?	aspartate aminotransferase levels	C	T	-0.024	0.976	0.681516317	0.006	1.006	0.9475428	0.77401561	1	0.78515	1
ALT/AST	chr10:17219763:G:A	3E-21	rs12413013-G	aspartate aminotransferase levels	G	A	-0.028	0.972	0.659405839	0.084	1.088	0.3865308	0.77898495	1	0.52091	1

© 2022 Yang W et al. *JAMA Network Open*.

ALT/AST	chr15:49544670:G:T	0.000000009	rs12593917-T	aspartate aminotransferase levels	G	T	-0.004	0.996	0.94916776	0.060	1.061	0.5502598	0.77993527	1	0.38576	1
ALT/AST	chr20:34962143:C:T	8E-15	rs11698868-T	aspartate aminotransferase levels	C	T	0.043	1.044	0.5749276	-0.099	0.906	0.3863507	0.77999834	1	0.78160	1
ALT/AST	chr18:57655270:C:T	9E-10	rs12968116-C	liver enzyme levels (gamma-glutamyl transferase)	C	T	-0.001	0.999	0.992430509	-0.060	0.941	0.6894855	0.78048056	1	0.81839	1
ALT/AST	chr10:94949217:A:G	0.000000001	p.H251R	CPIC:CYP2C9	A	G	-0.282	0.754	0.53721972	0.326	1.386	0.4150187	0.78052446	1	0.67530	1
ALT/AST	chr1:26811902:C:T	0.00000001	rs12748152-C	alanine aminotransferase (alt) levels after remission induction therapy in actute lymphoblastic leukemia (all)	C	T	0.017	1.017	0.895508865	-0.117	0.890	0.5487849	0.78243618	1	0.93083	1
ALT/AST	chr7:99652613:G:T	0.000000001	T398N	CPIC:CYP3A5	G	T	0.270	1.310	0.656567126	-0.772	0.462	0.3598095	0.7825243	1	0.78348	1
ALT/AST	chr9:34107507:C:T	0.000000003	rs11557154-T	alanine aminotransferase levels	C	T	0.016	1.017	0.839368154	0.007	1.008	0.9515502	0.78345315	1	0.74749	1
ALT/AST	chr17:80390047:G:A	5E-10	rs3185057-G	aspartate aminotransferase levels	G	A	0.019	1.019	0.869382445	-0.110	0.896	0.5312068	0.7884744	1	0.56840	1
ALT/AST	chr2:168977860:G:A	9E-27	rs72623176-A	alanine aminotransferase levels	G	A	0.028	1.029	0.817509136	-0.135	0.873	0.4228703	0.79545233	1	0.74307	1
ALT/AST	chr1:93392732:G:A	2E-10	rs1365298-A	alanine aminotransferase levels	G	A	-0.026	0.975	0.712853132	0.109	1.115	0.2995656	0.79729716	1	0.44759	1
ALT/AST	chr6:135097778:A:G	6E-20	rs7776054-A	aspartate aminotransferase levels	A	G	0.018	1.018	0.804637141	0.010	1.010	0.9248978	0.8027896	1	0.35115	1

© 2022 Yang W et al. *JAMA Network Open*.

ALT/AST	chr6:135097901:C:A	1E-14	rs56293029-?	aspartate aminotransferase platelet ratio index in high alcohol intake	C	A	0.017	1.018	0.806221351	0.010	1.010	0.9248978	0.80476097	1	0.35441	1
ALT/AST	chr2:233746667:C:A	4E-16	rs4663969-?	total bilirubin levels in hiv-1 infection	C	A	-0.008	0.992	0.899138436	0.048	1.049	0.5885981	0.80481158	1	0.17565	1

ALT/AST	chr12:111280427:C:A	1E-30	rs79105258-?	alanine transaminase levels;alanine aminotransferase levels;aspartate aminotransferase levels	C	A	0.271	1.311	0.516315517	-0.479	0.619	0.3162808	0.80563519	1	0.60317	1
ALT/AST	chr1:150507425:C:T	1E-12	rs1815544-C	alanine aminotransferase levels	C	T	0.010	1.010	0.87160462	0.026	1.026	0.7856879	0.80752568	1	0.12603	1
ALT/AST	chr2:201196097:C:T	0.00000001	rs3731714-C	alanine aminotransferase levels	C	T	-0.065	0.937	0.351612815	0.101	1.106	0.3273983	0.80872887	1	0.61694	1
ALT/AST	chr1:97883329:A:G	0.000000001	p.C29R	CPIC:DPYD	A	G	-0.023	0.977	0.749756286	0.012	1.012	0.9124986	0.80991027	1	0.11845	1
ALT/AST	chr12:20426458:G:C	0.00000001	rs7488780-G	aspartate aminotransferase levels	G	C	-0.028	0.973	0.71682137	0.092	1.096	0.4198414	0.81092898	1	0.06711	1
ALT/AST	chr2:233760498:G:A	3E-139	rs4148323-?	bilirubin levels	G	A	0.243	1.275	0.399001532	-0.558	0.572	0.2959212	0.81216024	1	0.33360	1
ALT/AST	chr2:233760498:G:A	0.000000001	G71R	CPIC:UGT1A1	G	A	0.243	1.275	0.399001532	-0.558	0.572	0.2959212	0.81216024	1	0.33360	1
ALT/AST	chr2:85188306:A:T	1E-13	rs2568207-T	aspartate aminotransferase levels	A	T	-0.005	0.995	0.937776429	-0.002	0.998	0.9830107	0.81880443	1	0.46250	1
ALT/AST	chr12:89147879:G:C	0.00000001	rs10858828-G	aspartate aminotransferase levels	G	C	0.012	1.012	0.872780106	-0.058	0.944	0.579392	0.81914852	1	0.09470	1
ALT/AST	chr6:26116754:C:T	0.00000002	rs12206204-T	bilirubin levels	C	T	-0.237	0.789	0.359726139	0.250	1.285	0.4673706	0.8200613	1	0.11559	1
ALT/AST	chr17:48065680:T:C	9E-10	rs7209484-C	aspartate aminotransferase levels	T	C	0.023	1.024	0.728078108	-0.030	0.970	0.7766322	0.82086292	1	0.49041	1

© 2022 Yang W et al. *JAMA Network Open*.

ALT/AST	chr22:38203357:G:A	6E-12	rs4821764-A	alanine aminotransferase levels	G	A	0.028	1.028	0.646370866	-0.031	0.969	0.7177323	0.82092514	1	0.40989	1
ALT/AST	chr6:135098493:A:G	0.000000004	rs9389268-?	aspartate aminotransferase levels	A	G	0.017	1.017	0.816667081	0.011	1.011	0.9204552	0.82205549	1	0.35915	1
ALT/AST	chr22:42127803:C:T	0.000000001	Splicing defect	CPIC:CYP2D6	C	T	0.088	1.092	0.400455886	-0.140	0.869	0.3980361	0.8237772	1	0.58558	1
ALT/AST	chr10:18221519:G:A	3E-14	rs2489201-?	aspartate aminotransferase levels	G	A	0.060	1.062	0.360824151	-0.110	0.896	0.2401777	0.82516948	1	0.52823	1
ALT/AST	chr5:161257724:T:C	0.000000003	rs114811931-	drug-induced liver injury (diclofenac)	T	C	-0.093	0.911	0.537548981	0.143	1.153	0.6265092	0.82521667	1	0.83808	1
ALT/AST	chr17:72110287:T:C	1E-12	rs1477066-C	alanine aminotransferase levels	T	C	-0.013	0.987	0.822446132	0.013	1.013	0.8880599	0.82681154	1	0.49059	1
ALT/AST	chr10:94344908:T:G	0.00000002	rs3758526-G	aspartate aminotransferase levels	T	G	-0.018	0.982	0.839930774	-0.003	0.997	0.9788447	0.82719099	1	0.10207	1
ALT/AST	chr10:17974944:C:T	4E-159	rs508196-C	aspartate aminotransferase levels	C	T	0.018	1.018	0.76783906	-0.019	0.981	0.8340365	0.82730715	1	0.61778	1
ALT/AST	chr1:201047168:G:A	1E-10	rs3850625-G	aspartate aminotransferase levels	G	A	0.040	1.040	0.68481841	-0.021	0.979	0.8869061	0.82866099	1	0.48752	1
ALT/AST	chr17:72102020:G:C	0.000000001	rs9913711-C	liver enzyme levels (gamma-glutamyl transferase)	G	C	-0.026	0.975	0.685218625	0.089	1.093	0.3577803	0.83191835	1	0.16658	1
ALT/AST	chr1:28245806:G:C	2E-13	rs12129745-G	aspartate aminotransferase levels	G	C	-0.057	0.945	0.387486889	0.069	1.071	0.4741429	0.83394203	1	0.86476	1
ALT/AST	chr12:21130388:G:A	0.000000001	NA	CPIC:SLCO1B1	G	A	-0.043	0.958	0.756947138	0.197	1.218	0.2926573	0.8350689	1	0.15718	1
ALT/AST	chr2:233260305:C:T	0.00000003	rs6758317-T	bilirubin levels	C	T	0.058	1.060	0.424192779	-0.118	0.889	0.28302	0.83576971	1	0.35406	1
ALT/AST	chr19:40991369:C:T	0.000000001	R22C	CPIC:CYP2B6	C	T	0.078	1.081	0.583116549	-0.119	0.888	0.610377	0.84242295	1	0.79507	1
ALT/AST	chr11:61855668:C:T	0.000000003	rs174601-T	liver enzyme levels (alkaline phosphatase)	C	T	0.007	1.007	0.908285733	-0.002	0.998	0.9841911	0.84253472	1	0.37930	1

© 2022 Yang W et al. *JAMA Network Open*.

ALT/AST	chr2:113083453:A:G	5E-16	rs6734238-A	aspartate aminotransferase levels	A	G	-0.010	0.990	0.868138213	0.009	1.009	0.9190975	0.84345071	1	0.62009	1
ALT/AST	chr2:71335373:T:C	2E-10	rs17743415-C	aspartate aminotransferase levels	T	C	-0.005	0.995	0.929937813	0.012	1.013	0.8955795	0.84475264	1	0.21550	1
ALT/AST	chr9:4774830:G:C	9E-15	rs35954307-?	aspartate aminotransferase platelet ratio index in high alcohol intake	G	C	-0.024	0.976	0.753134356	0.081	1.084	0.4905459	0.84479753	1	0.79412	1
ALT/AST	chr9:6665010:C:T	2E-11	rs1658972-C	alanine aminotransferase levels;alanine transaminase levels	C	T	-0.043	0.958	0.637885456	0.120	1.128	0.3095312	0.84517419	1	0.55436	1
ALT/AST	chr11:125582185:C:T	0.00000002	rs11220136-T	alanine aminotransferase levels	C	T	0.069	1.072	0.559265643	-0.086	0.917	0.6317123	0.84579106	1	0.29312	1
ALT/AST	chr7:56005148:C:T	0.00000003	rs4948100-?	alanine aminotransferase levels	C	T	-0.036	0.964	0.576828927	0.106	1.111	0.2826917	0.84631748	1	0.04404	1
ALT/AST	chr17:75397378:T:A	2E-10	rs2053158-A	aspartate aminotransferase levels	T	A	-0.060	0.942	0.445033164	0.127	1.135	0.2715786	0.84691587	1	0.67117	1
ALT/AST	chr17:81513660:T:C	7E-25	rs11657440-C	alanine aminotransferase levels	T	C	-0.012	0.989	0.85547047	-0.005	0.995	0.954407	0.84808937	1	0.26269	1
ALT/AST	chr19:41012339:C:T	0.000000001	R336C	CPIC:CYP2B6	C	T	-10.910	0.000	0.96189943	1.085	2.958	0.5562385	0.84868876	1	0.74495	1
ALT/AST	chr7:17872129:G:T	4E-25	rs1917368-G	aspartate aminotransferase levels	G	T	-0.027	0.973	0.652193198	0.025	1.026	0.7814902	0.8496177	1	0.70987	1

© 2022 Yang W et al. *JAMA Network Open*.

ALT/AST	chr16:58804727:G:A	0.00000002	rs35317979-?	aspartate transaminase levels in high alcohol intake;aspartate aminotransferase platelet ratio index in high alcohol intake	G	A	0.055	1.057	0.662939501	-0.056	0.945	0.761403	0.85164862	1	0.98483	1
ALT/AST	chr16:80463444:G:C	7E-22	rs28650012-G	alanine aminotransferase levels	G	C	-0.026	0.974	0.696346894	0.069	1.072	0.4749662	0.85632382	1	0.39180	1
ALT/AST	chr22:17168884:T:C	0.00000004	rs5748926-T	non-alcoholic fatty liver disease activity score in non-alcoholic fatty liver disease	T	C	0.025	1.026	0.693811296	-0.038	0.963	0.6994337	0.85758699	1	0.85604	1
ALT/AST	chr6:32400310:T:C	0.00000001	rs3817963-A	hepatitis c induced liver cirrhosis	T	C	0.004	1.004	0.946159303	-0.041	0.960	0.6712786	0.85818651	1	0.96739	1
ALT/AST	chr1:65675819:G:T	9E-13	rs1938500-T	aspartate aminotransferase levels;alanine aminotransferase levels	G	T	0.093	1.098	0.187021029	-0.181	0.834	0.0908647	0.86166266	1	0.07149	1
ALT/AST	chr6:20404189:T:C	7E-10	rs73382439-C	alanine aminotransferase levels	T	C	0.004	1.004	0.95551363	-0.032	0.968	0.7805497	0.86437703	1	0.68178	1
ALT/AST	chr8:144467535:G:A	0.000000003	rs35968570-?	alanine transaminase levels in high alcohol intake	G	A	-0.133	0.876	0.728340166	0.183	1.201	0.7509719	0.86528245	1	0.03097	1
ALT/AST	chr13:113849020:T:C	7E-16	rs6602909-C	aspartate aminotransferase levels	T	C	0.057	1.058	0.398978262	-0.086	0.918	0.3628488	0.86604129	1	0.75661	1
ALT/AST	chr7:135197671:T:A	6E-14	rs292580-A	aspartate aminotransferase levels	T	A	0.007	1.007	0.916979346	-0.015	0.985	0.8810451	0.86922035	1	0.50496	1
ALT/AST	chr22:42130710:G:A	0.000000001	p.R28C	CPIC:CYP2D6	G	A	-0.104	0.901	0.859124906	0.124	1.132	0.8921843	0.87053114	1	0.18375	1

© 2022 Yang W et al. *JAMA Network Open*.

ALT/AST	chr2:135839297:G:A	0.00000002	rs6742283-G	aspartate aminotransferase levels	G	A	0.021	1.021	0.738351205	-0.036	0.965	0.7000496	0.87303795	1	0.90820	1
ALT/AST	chr19:38268463:T:C	0.00000001	rs3786876-C	alanine aminotransferase levels	T	C	0.002	1.002	0.983578302	-0.026	0.974	0.8454713	0.87391987	1	0.18276	1
ALT/AST	chr9:114377802:T:C	1E-40	rs4979372-?	alanine aminotransferase levels;alanine transaminase levels	T	C	-0.003	0.997	0.956188146	0.022	1.022	0.8127045	0.87427455	1	0.47206	1
ALT/AST	chr10:94972119:C:A	0.000000001	p.P279T	CPIC:CYP2C9	C	A	0.206	1.229	0.872739835	-12.429	0.000	0.9769593	0.8745373	1	0.36769	1
ALT/AST	chrX:154326058:C:G	0.000000009	rs766420-G	bilirubin levels	C	G	-0.023	0.977	0.690081009	0.079	1.082	0.3149618	0.87919019	1	0.98650	1
ALT/AST	chr19:41307470:T:C	7E-10	rs15052-?	aspartate aminotransferase platelet ratio index in high alcohol intake	T	C	0.043	1.044	0.638425376	-0.063	0.939	0.630314	0.87957675	1	0.84033	1

© 2022 Yang W et al. *JAMA Network Open*.

ALT/AST	chr11:22250324:A:T	8E-26	rs7481951-T	aspartate aminotransferase levels;alanine aminotransferase levels	A	T	0.001	1.001	0.982879813	0.004	1.004	0.9633199	0.88146666	1	0.51457	1
ALT/AST	chr4:23924364:G:A	2E-10	rs11932940-A	aspartate aminotransferase levels	G	A	-0.063	0.939	0.323675442	0.186	1.205	0.053338	0.8816742	1	0.77168	1
ALT/AST	chr12:111286895:T:C	2E-18	rs4766568-T	aspartate aminotransferase levels	T	C	-0.024	0.976	0.764713135	0.067	1.070	0.5568511	0.88285503	1	0.93953	1
ALT/AST	chr19:15864286:G:T	0.00000003	rs11086005-G	aspartate aminotransferase levels	G	T	0.016	1.016	0.795094286	-0.079	0.924	0.3832689	0.88332338	1	0.26635	1
ALT/AST	chr17:70379805:T:C	4E-10	rs4485410-T	aspartate aminotransferase levels	T	C	-0.043	0.958	0.545586768	0.062	1.064	0.5717865	0.88405121	1	0.15173	1
ALT/AST	chr11:116778201:G:C	2E-19	rs964184-G	aspartate aminotransferase levels	G	C	0.013	1.013	0.864248102	0.041	1.041	0.7258503	0.88542709	1	0.36912	1
ALT/AST	chr7:135199435:C:T	2E-10	rs292585-T	alanine aminotransferase levels	C	T	0.006	1.006	0.932174597	-0.055	0.946	0.5672383	0.88768551	1	0.51198	1
ALT/AST	chr19:48703346:C:T	1E-15	rs281377-T	liver enzyme levels (alkaline phosphatase)	C	T	-0.022	0.978	0.70944434	0.021	1.021	0.8157829	0.8900688	1	0.77123	1
ALT/AST	chr10:73021160:A:G	0.000000002	rs79188145-A	alanine aminotransferase levels	A	G	0.008	1.008	0.935165879	0.027	1.027	0.8443076	0.89100624	1	0.03063	1
ALT/AST	chr22:42129075:C:T	0.000000001	p.E155K	CPIC:CYP2D6	C	T	-0.132	0.876	0.845616558	0.165	1.179	0.7688997	0.89118559	1	0.24316	1
ALT/AST	chr20:39913927:C:T	0.00000001	rs17803745-C	aspartate aminotransferase levels	C	T	0.047	1.048	0.447957078	-0.146	0.865	0.1316559	0.89158538	1	0.81525	1
ALT/AST	chr12:21176879:C:A	0.000000001	P155T	CPIC:SLCO1B1	C	A	0.075	1.078	0.377261692	-0.164	0.849	0.2190027	0.89279387	1	0.19477	1
ALT/AST	chr17:7188331:T:C	8E-12	rs314253-C	liver enzyme levels (alkaline phosphatase)	T	C	-0.006	0.994	0.918659601	-0.011	0.989	0.9034752	0.89366557	1	0.43194	1

© 2022 Yang W et al. *JAMA Network Open*.

ALT/AST	chr16:72072066:T:C	7E-17	rs217184-T	aspartate aminotransferase levels	T	C	-0.004	0.996	0.958239804	0.007	1.007	0.9471788	0.89616431	1	0.68678	1
ALT/AST	chr8:60613221:G:A	9E-16	rs671275-A	aspartate aminotransferase levels	G	A	0.013	1.013	0.850449112	-0.042	0.959	0.6752502	0.89725944	1	0.35936	1
ALT/AST	chr19:33398687:A:G	5E-16	rs7256564-A	alanine aminotransferase levels	A	G	0.059	1.061	0.350966247	-0.092	0.912	0.3249446	0.89774164	1	0.75298	1
ALT/AST	chr6:24491247:A:C	6E-26	rs1883415-C	liver enzyme levels (alkaline phosphatase)	A	C	-0.066	0.936	0.303715485	0.169	1.184	0.0778994	0.89871758	1	0.46280	1
ALT/AST	chr3:12355089:G:A	6E-22	rs4135247-G	alanine aminotransferase levels;aspartate aminotransferase levels	G	A	0.049	1.050	0.419000233	-0.129	0.879	0.1445388	0.90034051	1	0.18078	1
ALT/AST	chr11:94198621:T:C	1E-24	rs1857682-?	alanine aminotransferase levels	T	C	0.049	1.050	0.48252641	-0.134	0.875	0.2044924	0.90198811	1	0.81019	1
ALT/AST	chr1:59214574:T:G	2E-10	rs6678642-T	aspartate aminotransferase levels	T	G	0.049	1.050	0.472584651	-0.056	0.946	0.5776893	0.90273192	1	0.56641	1
ALT/AST	chr5:52897294:A:G	3E-13	rs4074793-G	alanine aminotransferase levels;liver enzyme levels (gamma-glutamyl transferase)	A	G	0.042	1.043	0.700524731	-0.070	0.932	0.6930511	0.90388098	1	0.77793	1
ALT/AST	chr1:196729333:A:G	4E-10	rs6689009-A	aspartate aminotransferase levels	A	G	0.039	1.040	0.62424064	-0.045	0.956	0.6977294	0.90400629	1	0.50757	1
ALT/AST	chr6:36679903:T:C	1E-10	rs4135240-T	aspartate aminotransferase levels	T	C	0.000	1.000	0.996756968	-0.006	0.994	0.9467061	0.90581929	1	0.05601	1
ALT/AST	chr10:77920676:A:T	6E-10	rs754466-T	liver enzyme levels (gamma-glutamyl transferase)	A	T	-0.016	0.984	0.819527339	0.012	1.012	0.9088993	0.9082411	1	0.42830	1

© 2022 Yang W et al. *JAMA Network Open*.

ALT/AST	chr7:87430383:C:T	3E-12	rs31672-T	aspartate aminotransferase levels	C	T	-0.007	0.993	0.921176961	0.026	1.026	0.7996216	0.90885985	1	0.33943	1
ALT/AST	chr2:112957399:T:G	0.000000006	rs12617864-G	alanine aminotransferase levels	T	G	-0.062	0.940	0.33733808	0.113	1.120	0.2619665	0.91106024	1	0.26194	1
ALT/AST	chr5:31021251:A:G	6E-25	rs10075805-A	alanine aminotransferase levels;aspartate aminotransferase levels	A	G	0.119	1.127	0.100747229	-0.212	0.809	0.0473129	0.91365388	1	0.21273	1
ALT/AST	chr12:21178615:T:C	7E-13	rs4149056-C	bilirubin levels	T	C	-0.050	0.951	0.566640517	0.133	1.142	0.304859	0.91578958	1	0.01117	1
ALT/AST	chr12:21178615:T:C	0.000000001	V174A	CPIC:SLCO1B1	T	C	-0.050	0.951	0.566640517	0.133	1.142	0.304859	0.91578958	1	0.01117	1
ALT/AST	chr17:43058379:A:C	1E-12	rs8176279-C	aspartate aminotransferase levels	A	C	-0.035	0.965	0.575271289	0.109	1.115	0.2475922	0.91728149	1	0.25477	1
ALT/AST	chr7:117587806:G:A	0.000000001	G551D	CPIC:CFTR	G	A	0.268	1.308	0.882765841	-10.658	0.000	0.9738187	0.92190062	1	0.25367	1
ALT/AST	chr4:99527667:C:T	0.00000002	rs10007975-C	alanine aminotransferase levels	C	T	0.006	1.006	0.932030877	-0.040	0.961	0.695096	0.92237552	1	0.88224	1
ALT/AST	chr1:97679170:T:C	0.000000001	p.K259E	CPIC:DPYD	T	C	0.172	1.188	0.693769526	-0.427	0.652	0.5485531	0.92266108	1	0.91523	1
ALT/AST	chr4:3442204:A:G	3E-12	rs13108218-A	alanine aminotransferase levels;aspartate aminotransferase levels	A	G	0.048	1.049	0.418170541	-0.088	0.916	0.3127647	0.92350925	1	0.17945	1
ALT/AST	chr22:42130715:C:T	0.000000001	p.R26H	CPIC:CYP2D6	C	T	-0.386	0.680	0.603520347	0.636	1.889	0.4631423	0.92483293	1	0.52247	1
ALT/AST	chr12:26321934:G:A	0.00000002	rs10842708-G	alanine aminotransferase levels	G	A	-0.034	0.966	0.602705613	0.090	1.094	0.3583216	0.92939603	1	0.18550	1
ALT/AST	chr1:97721542:T:C	0.000000001	p.N151D	CPIC:DPYD	T	C	-0.289	0.749	0.65215445	0.384	1.469	0.6076721	0.93085172	1	0.28437	1
ALT/AST	chr9:114383763:C:G	8E-124	rs7041363-C	alanine aminotransferase levels	C	G	-0.007	0.993	0.91070866	0.020	1.020	0.82869	0.93300199	1	0.40773	1

© 2022 Yang W et al. *JAMA Network Open*.

ALT/AST	chr6:135106021:T:C	5E-14	rs9389269-?	aspartate aminotransferase platelet ratio index in high alcohol intake	T	C	0.024	1.024	0.734140184	-0.037	0.964	0.7315887	0.93990084	1	0.35871	1
ALT/AST	chr12:21176827:G:A	0.000000001	S137=	CPIC:SLCO1B1	G	A	0.065	1.068	0.449130203	-0.160	0.852	0.2373332	0.94979892	1	0.22053	1
ALT/AST	chr11:61824890:A:G	0.00000002	rs174566-?	aspartate aminotransferase platelet ratio index in high alcohol intake	A	G	0.001	1.001	0.983675198	-0.029	0.971	0.757992	0.95002645	1	0.11389	1
ALT/AST	chr12:112136812:C:T	3E-13	rs12231737-T	aspartate aminotransferase levels;alanine aminotransferase levels	C	T	0.157	1.170	0.706837228	-0.145	0.865	0.7720419	0.95126009	1	0.28366	1
ALT/AST	chr1:26700496:C:G	0.00000003	rs4579782-G	alanine aminotransferase levels	C	G	0.042	1.043	0.745700629	-0.107	0.899	0.5849683	0.95143894	1	0.96090	1
ALT/AST	chr22:42128308:C:A	0.000000001	p.A237S	CPIC:CYP2D6	C	A	-0.206	0.813	0.566081124	0.321	1.379	0.5053122	0.95395536	1	0.50853	1
ALT/AST	chr10:94761900:C:T	0.000000001	5' region	CPIC:CYP2C19	C	T	-0.024	0.976	0.761231333	0.069	1.072	0.5480777	0.9543611	1	0.47116	1
ALT/AST	chr9:114382224:C:T	1E-51	rs4979373-C	aspartate aminotransferase levels	C	T	-0.010	0.990	0.859795597	0.006	1.006	0.945361	0.95823971	1	0.37135	1
ALT/AST	chr12:112379979:T:A	2E-11	rs11066280-?	aspartate aminotransferase levels;alanine aminotransferase levels	T	A	0.112	1.119	0.786358156	-0.084	0.919	0.8617914	0.95926799	1	0.28772	1
ALT/AST	chr3:50080087:A:G	0.000000007	rs2624817-A	alanine aminotransferase levels	A	G	-0.081	0.922	0.317141081	0.186	1.205	0.1530672	0.95975823	1	0.04947	1
ALT/AST	chr6:32421871:A:G	1E-10	rs3135363-C	hepatitis c induced liver cirrhosis	A	G	-0.039	0.962	0.566615935	0.100	1.105	0.3394282	0.96135819	1	0.09947	1
ALT/AST	chr12:20904347:C:T	7E-13	rs7953767-T	alanine aminotransferase levels	C	T	0.008	1.008	0.921209942	-0.023	0.977	0.8472151	0.96140435	1	0.17337	1
ALT/AST	chr1:97515839:T:C	0.000000001	p.I543V	CPIC:DPYD	T	C	0.021	1.021	0.767876577	-0.025	0.975	0.8157469	0.9642078	1	0.79765	1

© 2022 Yang W et al. *JAMA Network Open*.

ALT/AST	chr7:55869748:T:A	0.000000004	rs7803882-?	alanine transaminase levels	T	A	0.040	1.041	0.497784145	-0.090	0.914	0.3180269	0.96425092	1	0.19004	1

© 2022 Yang W et al. *JAMA Network Open*.

ALT/AST	chr1:65675819:G:GT	9E-13	rs1938500-T	aspartate aminotransferase levels;alanine aminotransferase levels	G	GT	0.464	1.590	0.798709084	-10.448	0.000	0.9743336	0.96462088	1	0.41917	1
ALT/AST	chr6:43790159:C:A	1E-16	rs998584-A	aspartate aminotransferase levels	C	A	-0.001	0.999	0.988923346	-0.002	0.998	0.9857684	0.97973665	1	0.99693	1
ALT/AST	chr4:145893489:G:A	8E-13	rs7682289-?	alanine aminotransferase levels	G	A	0.008	1.008	0.909316118	-0.071	0.931	0.5339184	0.98063897	1	0.11304	1
ALT/AST	chr17:66212167:C:G	8E-17	rs1801690-G	aspartate aminotransferase levels	C	G	0.068	1.070	0.642145572	-0.184	0.832	0.4232325	0.98246837	1	0.17287	1
ALT/AST	chr13:48045719:C:T	0.000000001	R139C	CPIC:NUDT15	C	T	-0.053	0.948	0.816690269	0.087	1.091	0.813758	0.98394639	1	0.85760	1
ALT/AST	chr7:87445648:T:C	4E-42	rs1468615-T	alanine aminotransferase levels	T	C	-0.043	0.958	0.572778539	0.094	1.098	0.3956263	0.98485147	1	0.79855	1
ALT/AST	chr2:233716976:C:T	7E-12	rs1875263-?	total bilirubin levels in hiv-1 infection	C	T	-0.036	0.965	0.579345778	0.079	1.082	0.3982122	0.98916963	1	0.31501	1
ALT/AST	chr2:233768740:G:A	3E-81	rs6431630-?	total bilirubin levels	G	A	-0.010	0.990	0.923025319	0.036	1.037	0.7968497	0.99169867	1	0.07568	1
ALT/AST	chr11:61836038:C:A	0.000000001	rs174576-?	alanine aminotransferase levels	C	A	-0.010	0.990	0.872247407	-0.002	0.998	0.9831364	0.99680708	1	0.48578	1
ALT/AST	chr2:233760973:C:A	0.000000001	P229Q	CPIC:UGT1A1	C	A	0.107	1.113	0.896211389	-0.165	0.848	0.8705396	1	1	0.06215	1
ALT/AST	chr8:144729622:T:C	5E-25	rs2467663-T	alanine aminotransferase levels	T	C	-0.004	0.996	0.955409706	0.027	1.027	0.7680315	1	1	0.26015	1
ALT/AST	chr2:210675783:C:A	6E-18	rs1047891-?	alanine transaminase levels;alanine aminotransferase levels;protein quantitative trait loci (liver)	C	A	0.019	1.020	0.760840192	-0.025	0.975	0.7920004	1	1	0.33031	1
ALT/AST	chr11:65408899:C:T	3E-13	rs12799896-C	alanine aminotransferase levels	C	T	-0.015	0.985	0.84638557	-0.001	0.999	0.9950218	1	1	0.35711	1

© 2022 Yang W et al. *JAMA Network Open*.

ALT/AST	chr8:125478730:A:T	2E-16	rs2954029-?	alanine transaminase levels	A	T	-0.015	0.985	0.805450435	0.037	1.037	0.6847412	1	1	0.51473	1
ALT/AST	chr15:78944951:C:T	4E-11	rs2289702-T	aspartate aminotransferase levels	C	T	0.040	1.041	0.727628478	-0.085	0.919	0.6023107	1	1	0.57314	1
ALT/AST	chr16:83946924:C:G	1E-19	rs4782568-C	alanine aminotransferase levels	C	G	0.015	1.015	0.810045041	-0.034	0.967	0.7052975	1	1	0.99268	1
ALT/AST	chr3:142940519:AT:A	3E-11	rs6440123-A	alanine aminotransferase levels	AT	A	NA	NA	NA	NA	NA	NA	NA	NA	NA	NA
ALT/AST	chr19:46932214:G:A	0.00000002	rs547445211-	aspartate aminotransferase levels	G	A	NA	NA	NA	NA	NA	NA	NA	NA	NA	NA
ALT/AST	chr7:117530974:C:G	0.000000001	R117C	CPIC:CFTR	C	G	NA	NA	NA	NA	NA	NA	NA	NA	NA	NA
ALT/AST	chr7:117548795:C:T	0.000000001	A455E	CPIC:CFTR	C	T	NA	NA	NA	NA	NA	NA	NA	NA	NA	NA
ALT/AST	chr7:117611595:T:G	0.000000001	F1052V	CPIC:CFTR	T	G	NA	NA	NA	NA	NA	NA	NA	NA	NA	NA
ALT/AST	chr19:41010088:C:G	0.000000001	T306S	CPIC:CYP2B6	C	G	NA	NA	NA	NA	NA	NA	NA	NA	NA	NA
ALT/AST	chr10:94780574:G:A	0.000000001	R186P	CPIC:CYP2C19	G	A	NA	NA	NA	NA	NA	NA	NA	NA	NA	NA
ALT/AST	chr10:94780579:G:A	0.000000001	D188N	CPIC:CYP2C19	G	A	NA	NA	NA	NA	NA	NA	NA	NA	NA	NA
ALT/AST	chr10:94781858:C:T	0.000000001	P227L	CPIC:CYP2C19	C	T	NA	NA	NA	NA	NA	NA	NA	NA	NA	NA
ALT/AST	chr10:94852765:C:T	0.000000001	R442C	CPIC:CYP2C19	C	T	NA	NA	NA	NA	NA	NA	NA	NA	NA	NA
ALT/AST	chr10:94938771:C:T	0.000000001	p.P30L	CPIC:CYP2C9	C	T	NA	NA	NA	NA	NA	NA	NA	NA	NA	NA
ALT/AST	chr10:94942231:G:A	0.000000001	p.R124Q	CPIC:CYP2C9	G	A	NA	NA	NA	NA	NA	NA	NA	NA	NA	NA
ALT/AST	chr10:94981201:T:C	0.000000001	p.I327T	CPIC:CYP2C9	T	C	NA	NA	NA	NA	NA	NA	NA	NA	NA	NA
ALT/AST	chr22:42126605:G:A	0.000000001	p.S488F	CPIC:CYP2D6	G	A	NA	NA	NA	NA	NA	NA	NA	NA	NA	NA
ALT/AST	chr22:42126647:C:T	0.000000001	p.R474Q	CPIC:CYP2D6	C	T	NA	NA	NA	NA	NA	NA	NA	NA	NA	NA
ALT/AST	chr22:42127457:C:G	0.000000001	p.R388H	CPIC:CYP2D6	C	G	NA	NA	NA	NA	NA	NA	NA	NA	NA	NA
ALT/AST	chr22:42127846:C:T	0.000000001	p.321fs	CPIC:CYP2D6	C	T	NA	NA	NA	NA	NA	NA	NA	NA	NA	NA
ALT/AST	chr22:42128329:G:A	0.000000001	p.L230F	CPIC:CYP2D6	G	A	NA	NA	NA	NA	NA	NA	NA	NA	NA	NA
ALT/AST	chr1:97450168:A:G	0.000000001	p.M599T	CPIC:DPYD	A	G	NA	NA	NA	NA	NA	NA	NA	NA	NA	NA
ALT/AST	chr1:97549735:G:A	0.000000001	p.A450V	CPIC:DPYD	G	A	NA	NA	NA	NA	NA	NA	NA	NA	NA	NA
ALT/AST	chrX:154533122:C:T	0.000000001	p.V291M	CPIC:G6PD	C	T	NA	NA	NA	NA	NA	NA	NA	NA	NA	NA
ALT/AST	chr12:21176868:A:G	0.000000001	N151S	CPIC:SLCO1B1	A	G	NA	NA	NA	NA	NA	NA	NA	NA	NA	NA
ALT/AST	chr6:18130729:C:T	0.000000001	R226Q	CPIC:TPMT	C	T	NA	NA	NA	NA	NA	NA	NA	NA	NA	NA
ALT/AST	chr6:18133845:T:A	0.000000001	Y180F	CPIC:TPMT	T	A	NA	NA	NA	NA	NA	NA	NA	NA	NA	NA
ALT/AST	chr6:18138970:G:A	0.000000001	R163C	CPIC:TPMT	G	A	NA	NA	NA	NA	NA	NA	NA	NA	NA	NA

© 2022 Yang W et al. *JAMA Network Open*.

ALT/AST	chr6:18147851:G:C	0.000000001	L69V	CPIC:TPMT	G	C	NA	NA	NA	NA	NA	NA	NA	NA	NA	NA

ALT/AST	chr12:20845519:T:C	4E-13	rs137858877-	total bilirubin levels	T	C	NA	NA	NA	NA	NA	NA	NA	NA	NA	NA

© 2022 Yang W et al. *JAMA Network Open*.

eTable 3. Top PRS from PRS catalog. Shown are statistics across treatment phases based on mixed-effects logistic model. Odds ratio estimates correspond to the increase of PRS score by one standard deviation in PRS score. PGS000687 (3,105 SNPs) and PGS 000668 (12,077 SNPs) were obtained from PGS catalog (http://www.pgscatalog.org/).

			AALL0232 (n=2283)		AALL0434 (n=1274)		Meta Analysis	
All ancestries								
Toxicity	PGS	Adjusting for GWAS top SNP	Odds Ratio (CI)	p-value	Odds Ratio (CI)	p-value	p-value	p-value_interaction
Hyperbilirubinemia	direct_bili (PGS000681)	No	1.16 (1.12-1.2)	3.6e-17	1.09 (1.06-1.12)	5.6e-9	5.4e-23	0.114
	direct_bili (PGS000681)	Yes	1.01 (0.90-1.12)	0.90	1.05 (0.953-	0.324	0.4	0.61
Elevated ALT	ALT (PGS000668)	No	1.09 (1.04-1.14)	0.00045	1.08 (1.02-	0.0088	1.2e-5	0.254
	ALT (PGS000668)	Yes	1.06 (1.01-1.12)	0.019	1.07 (1.01-	0.027	0.0013	0.28
European ancestry only								
Toxicity	PGS	Adjusting for GWAS top SNP	Odds Ratio (CI)	p-value	Odds Ratio (CI)	p-value	p-value	p-value_interaction
Hyperbilirubinemia	direct_bili (PGS000681)	No	1.14 (1.1-1.19)	1.1e-9	1.1 (1.06-1.14)	1.42e-6	2.4e-14	0.20
Elevated Liver Enzymes	ALT (PGS000668)	No	1.08 (1.02-1.14)	0.013	1.08 (1.01-	0.03	0.0010	0.31

© 2022 Yang W et al. *JAMA Network Open*.

eTable 4. Association between liver function related PRS with hyperbilirubinemia or elevated ALT/AST in combined cohort or European Ancestry only cohort.

cohort	toxicity	term	pgs.trait	estimate AALL0232	OR AALL0232	p.value AALL0232	estimate AALL0434	OR AALL0434	p.value AALL0434	meta.p	adj.meta.p	adjusting for GWAS SNPs
All patients	Hyperbilirubinemia	PGS000681	Direct bilirubin [umol/L]	0.475	1.608	3.57E-17	0.556	1.744	0.000000	5.07E-23	2.33E-21	no
All patients	Hyperbilirubinemia	PGS000697	Total bilirubin [umol/L]	0.467	1.596	3.15E-17	0.545	1.724	0.000000	1.33E-22	6.11E-21	no
All patients	Elevated ALT/AST	PGS000668	Alanine aminotransferase [U/L]	0.136	1.145	4.51E-04	0.142	1.153	0.008783	1.20E-05	5.52E-04	no
All patients	Elevated ALT/AST	PGS000673	Aspartate aminotransferase [U/L]	0.094	1.099	2.26E-03	0.082	1.086	0.067940	0.000439	0.02	no
All patients	Elevated ALT/AST	PGS000668	Alanine aminotransferase [U/L]	0.092	1.096	1.85E-02	0.129	1.138	0.026141	0.001244	0.06	adjusted
All patients	Hyperbilirubinemia	PGS000683	Gamma glutamyltransferase [U/L]	0.207	1.230	6.00E-03	0.184	1.203	0.160103	0.004522	0.21	adjusted
All patients	Hyperbilirubinemia	PGS000683	Gamma glutamyltransferase [U/L]	0.232	1.262	2.44E-03	0.131	1.140	0.243117	0.004615	0.21	no
All patients	Elevated ALT/AST	PGS000694	SHBG [nmol/L]	0.061	1.063	4.91E-02	0.128	1.136	0.065100	0.007333	0.34	no
All patients	Hyperbilirubinemia	PGS000668	Alanine aminotransferase [U/L]	0.221	1.247	1.41E-03	0.108	1.114	0.447702	0.007923	0.36	no
All patients	Elevated ALT/AST	PGS000674	AST to ALT ratio	-0.071	0.932	1.07E-01	-0.117	0.889	0.053847	0.01404	0.65	no
All patients	Hyperbilirubinemia	PGS000674	AST to ALT ratio	-0.124	0.884	2.91E-02	-0.218	0.804	0.237449	0.020482	0.94	no
All patients	Hyperbilirubinemia	PGS000706	Hypertension	0.130	1.139	1.21E-01	0.093	1.098	0.086117	0.02077	0.96	no
All patients	Elevated ALT/AST	PGS000694	SHBG [nmol/L]	0.050	1.051	9.94E-02	0.126	1.135	0.104328	0.021831	1.00	adjusted
All patients	Elevated ALT/AST	PGS000705	Gallstones	0.041	1.042	9.26E-02	0.085	1.089	0.131074	0.024325	1.00	adjusted
All patients	Hyperbilirubinemia	PGS000707	Cholecystitis	0.232	1.261	2.68E-03	0.066	1.069	0.723766	0.025697	1.00	no
All patients	Elevated ALT/AST	PGS000673	Aspartate aminotransferase [U/L]	0.050	1.051	4.51E-02	0.041	1.042	0.283948	0.026116	1.00	adjusted
All patients	Hyperbilirubinemia	PGS000702	Vitamin D [nmol/L]	-0.026	0.974	2.24E-01	-0.309	0.734	0.063376	0.02761	1.00	no
All patients	Hyperbilirubinemia	PGS000713	T2D	0.179	1.196	4.03E-02	0.083	1.087	0.242572	0.027818	1.00	no
All patients	Hyperbilirubinemia	PGS000706	Hypertension	0.112	1.118	1.88E-01	0.048	1.050	0.081511	0.029298	1.00	adjusted
All patients	Hyperbilirubinemia	PGS000704	Alcoholic cirrhosis	0.211	1.235	1.48E-02	0.110	1.117	0.499862	0.03762	1.00	no
All patients	Elevated ALT/AST	PGS000705	Gallstones	0.033	1.033	1.49E-01	0.089	1.093	0.155360	0.0446	1.00	no
All patients	Elevated ALT/AST	PGS000674	AST to ALT ratio	-0.048	0.953	3.21E-01	-0.115	0.892	0.055291	0.049053	1.00	adjusted
All patients	Hyperbilirubinemia	PGS000702	Vitamin D [nmol/L]	-0.006	0.994	3.20E-01	-0.273	0.761	0.100784	0.058117	1.00	adjusted
All patients	Hyperbilirubinemia	PGS000668	Alanine aminotransferase [U/L]	0.137	1.146	7.67E-02	0.150	1.162	0.345212	0.062416	1.00	adjusted
All patients	Elevated ALT/AST	PGS000709	Heart failure	-0.066	0.936	1.07E-01	-0.077	0.926	0.357078	0.066004	1.00	no
All patients	Hyperbilirubinemia	PGS000712	T2D (cases vs HbA1c filtered controls)	0.145	1.156	1.30E-01	0.097	1.101	0.274901	0.070925	1.00	no
All patients	Hyperbilirubinemia	PGS000671	Apolipoprotein A [g/L]	0.079	1.082	8.58E-02	0.004	1.004	0.397283	0.077471	1.00	adjusted
All patients	Elevated ALT/AST	PGS000707	Cholecystitis	0.007	1.007	3.47E-01	0.112	1.118	0.096225	0.077722	1.00	adjusted

© 2022 Yang W et al. *JAMA Network Open*.

All patients	Hyperbilirubinemia	PGS000671	Apolipoprotein A [g/L]	0.080	1.083	1.00E-01	0.004	1.004	0.379917	0.082238	1.00	no

All patients	Elevated ALT/AST	PGS000688	LDL cholesterol [mmol/L] (statin adjusted)	-0.023	0.977	4.35E-01	-0.097	0.908	0.075442	0.082357	1.00	no
All patients	Elevated ALT/AST	PGS000709	Heart failure	-0.062	0.940	1.27E-01	-0.070	0.932	0.387878	0.082577	1.00	adjusted
All patients	Hyperbilirubinemia	PGS000674	AST to ALT ratio	-0.065	0.937	2.03E-01	-0.227	0.797	0.238446	0.085015	1.00	adjusted
All patients	Elevated ALT/AST	PGS000672	Apolipoprotein B [g/L] (statin adjusted)	-0.027	0.973	3.27E-01	-0.083	0.920	0.133676	0.086786	1.00	no
All patients	Elevated ALT/AST	PGS000699	Triglycerides [mmol/L]	-0.043	0.958	3.78E-01	-0.135	0.874	0.109905	0.093525	1.00	adjusted
All patients	Elevated ALT/AST	PGS000699	Triglycerides [mmol/L]	-0.049	0.952	3.06E-01	-0.110	0.896	0.190161	0.107243	1.00	no
All patients	Hyperbilirubinemia	PGS000672	Apolipoprotein B [g/L] (statin adjusted)	-0.161	0.851	6.21E-02	0.053	1.055	0.575056	0.108185	1.00	no
All patients	Hyperbilirubinemia	PGS000704	Alcoholic cirrhosis	0.168	1.183	8.33E-02	0.118	1.126	0.556587	0.118405	1.00	adjusted
All patients	Elevated ALT/AST	PGS000672	Apolipoprotein B [g/L] (statin adjusted)	-0.026	0.974	3.40E-01	-0.069	0.933	0.200384	0.120541	1.00	adjusted
All patients	Elevated ALT/AST	PGS000688	LDL cholesterol [mmol/L] (statin adjusted)	-0.020	0.980	4.67E-01	-0.085	0.918	0.122355	0.121556	1.00	adjusted
All patients	Hyperbilirubinemia	PGS000692	Phosphate [mmol/L]	0.135	1.145	4.36E-01	0.126	1.135	0.171525	0.122801	1.00	adjusted
All patients	Elevated ALT/AST	PGS000677	Cholesterol [mmol/L] (statin adjusted)	-0.017	0.983	6.13E-01	-0.104	0.902	0.072901	0.126721	1.00	no
All patients	Hyperbilirubinemia	PGS000713	T2D	0.151	1.163	1.50E-01	0.033	1.034	0.428708	0.127603	1.00	adjusted
All patients	Elevated ALT/AST	PGS000707	Cholecystitis	0.002	1.002	4.56E-01	0.107	1.113	0.133482	0.13145	1.00	no
All patients	Hyperbilirubinemia	PGS000692	Phosphate [mmol/L]	0.139	1.149	3.01E-01	0.060	1.062	0.289702	0.139645	1.00	no
All patients	Hyperbilirubinemia	PGS000707	Cholecystitis	0.177	1.194	1.94E-02	0.021	1.022	0.900346	0.149995	1.00	adjusted
All patients	Hyperbilirubinemia	PGS000696	Testosterone [nmol/L]	-0.157	0.855	1.28E-01	-0.102	0.903	0.613757	0.162032	1.00	adjusted
All patients	Hyperbilirubinemia	PGS000705	Gallstones	0.126	1.134	2.68E-02	-0.025	0.976	0.905450	0.166683	1.00	no
All patients	Hyperbilirubinemia	PGS000700	Urate [umol/L]	-0.066	0.936	4.17E-01	-0.084	0.919	0.270312	0.172557	1.00	no
All patients	Elevated ALT/AST	PGS000702	Vitamin D [nmol/L]	0.002	1.002	5.24E-01	-0.059	0.942	0.169520	0.175973	1.00	no
All patients	Hyperbilirubinemia	PGS000673	Aspartate aminotransferase [U/L]	0.201	1.223	3.14E-03	-0.147	0.863	0.477104	0.195703	1.00	no
All patients	Hyperbilirubinemia	PGS000695	Sodium in urine [mmol/L]	0.155	1.167	7.69E-01	0.026	1.026	0.148570	0.196378	1.00	adjusted
All patients	Hyperbilirubinemia	PGS000672	Apolipoprotein B [g/L] (statin adjusted)	-0.138	0.871	1.22E-01	0.054	1.056	0.685912	0.197816	1.00	adjusted
All patients	Hyperbilirubinemia	PGS000695	Sodium in urine [mmol/L]	0.125	1.133	9.20E-01	0.067	1.069	0.110197	0.198823	1.00	no
All patients	Hyperbilirubinemia	PGS000700	Urate [umol/L]	-0.073	0.930	4.59E-01	-0.111	0.895	0.306531	0.2089	1.00	adjusted
All patients	Elevated ALT/AST	PGS000693	Potassium in urine [mmol/L]	-0.051	0.950	9.28E-02	0.036	1.036	0.927637	0.209891	1.00	no
All patients	Hyperbilirubinemia	PGS000679	Creatinine in urine [umol/L]	0.096	1.101	5.47E-01	0.071	1.073	0.256898	0.210634	1.00	adjusted
All patients	Elevated ALT/AST	PGS000693	Potassium in urine [mmol/L]	-0.041	0.960	1.39E-01	0.027	1.027	0.886205	0.214443	1.00	adjusted
All patients	Elevated ALT/AST	PGS000677	Cholesterol [mmol/L] (statin adjusted)	-0.009	0.991	7.23E-01	-0.093	0.911	0.137227	0.223154	1.00	adjusted
All patients	Hyperbilirubinemia	PGS000679	Creatinine in urine [umol/L]	0.074	1.077	7.18E-01	0.115	1.122	0.203215	0.229704	1.00	no
All patients	Elevated ALT/AST	PGS000702	Vitamin D [nmol/L]	0.005	1.005	6.60E-01	-0.060	0.941	0.177726	0.236343	1.00	adjusted

© 2022 Yang W et al. *JAMA Network Open*.

All patients	Elevated ALT/AST	PGS000671	Apolipoprotein A [g/L]	0.035	1.035	1.97E-01	-0.013	0.987	0.795724	0.239549	1.00	adjusted
All patients	Elevated ALT/AST	PGS000671	Apolipoprotein A [g/L]	0.032	1.033	2.00E-01	-0.011	0.989	0.831499	0.253409	1.00	no
All patients	Hyperbilirubinemia	PGS000696	Testosterone [nmol/L]	-0.144	0.866	2.73E-01	-0.114	0.892	0.584682	0.253753	1.00	no

All patients	Hyperbilirubinemia	PGS000676	Calcium [mmol/L]	0.077	1.080	3.23E-01	0.125	1.133	0.518941	0.256724	1.00	adjusted
All patients	Hyperbilirubinemia	PGS000712	T2D (cases vs HbA1c filtered controls)	0.119	1.126	3.54E-01	0.045	1.046	0.498615	0.266151	1.00	adjusted
All patients	Hyperbilirubinemia	PGS000688	LDL cholesterol [mmol/L] (statin adjusted)	-0.148	0.863	1.69E-01	0.064	1.066	0.772675	0.276871	1.00	no
All patients	Hyperbilirubinemia	PGS000680	Cystatin C [mg/L]	0.096	1.100	7.27E-02	0.055	1.057	0.884580	0.291588	1.00	no
All patients	Elevated ALT/AST	PGS000696	Testosterone [nmol/L]	-0.003	0.997	5.45E-01	0.063	1.065	0.348000	0.296438	1.00	no
All patients	Hyperbilirubinemia	PGS000676	Calcium [mmol/L]	0.075	1.078	4.89E-01	0.118	1.125	0.446906	0.303978	1.00	no
All patients	Elevated ALT/AST	PGS000698	Total protein [g/L]	-0.006	0.995	7.55E-01	0.132	1.142	0.050123	0.33538	1.00	adjusted
All patients	Hyperbilirubinemia	PGS000680	Cystatin C [mg/L]	0.079	1.082	7.23E-02	0.028	1.029	0.759622	0.347528	1.00	adjusted
All patients	Elevated ALT/AST	PGS000710	Myocardial infarction	-0.044	0.957	5.68E-01	-0.053	0.948	0.430658	0.348799	1.00	no
All patients	Hyperbilirubinemia	PGS000689	Lipoprotein A [nmol/L]	0.042	1.043	6.47E-01	0.222	1.248	0.408162	0.353844	1.00	no
All patients	Elevated ALT/AST	PGS000698	Total protein [g/L]	-0.005	0.995	8.38E-01	0.124	1.132	0.077146	0.354478	1.00	no
All patients	Elevated ALT/AST	PGS000703	Angina	-0.071	0.931	2.28E-01	0.019	1.019	0.979452	0.354602	1.00	no
All patients	Elevated ALT/AST	PGS000704	Alcoholic cirrhosis	0.008	1.008	3.39E-01	0.028	1.028	0.792883	0.36255	1.00	no
All patients	Hyperbilirubinemia	PGS000684	Glucose [mmol/L]	-0.015	0.985	8.25E-01	-0.094	0.910	0.319840	0.372763	1.00	adjusted
All patients	Hyperbilirubinemia	PGS000675	C-reactive protein [mg/L]	0.185	1.204	1.20E-01	0.048	1.049	0.891590	0.376592	1.00	no
All patients	Hyperbilirubinemia	PGS000689	Lipoprotein A [nmol/L]	0.047	1.048	5.27E-01	0.222	1.249	0.557679	0.391536	1.00	adjusted
All patients	Elevated ALT/AST	PGS000687	IGF-1 [nmol/L]	-0.010	0.990	6.56E-01	0.107	1.113	0.055520	0.391779	1.00	adjusted
All patients	Elevated ALT/AST	PGS000680	Cystatin C [mg/L]	-0.023	0.978	6.08E-01	0.134	1.144	0.042025	0.392262	1.00	adjusted
All patients	Hyperbilirubinemia	PGS000669	Albumin [g/L]	-0.090	0.914	6.29E-01	-0.122	0.885	0.476058	0.392428	1.00	no
All patients	Hyperbilirubinemia	PGS000705	Gallstones	0.092	1.096	6.39E-02	-0.076	0.927	0.592540	0.402318	1.00	adjusted
All patients	Elevated ALT/AST	PGS000710	Myocardial infarction	-0.039	0.961	6.59E-01	-0.058	0.944	0.437255	0.407307	1.00	adjusted
All patients	Hyperbilirubinemia	PGS000681	Direct bilirubin [umol/L]	0.011	1.011	9.03E-01	0.546	1.726	0.324220	0.407456	1.00	adjusted
All patients	Elevated ALT/AST	PGS000669	Albumin [g/L]	0.018	1.018	4.88E-01	0.045	1.046	0.653293	0.410313	1.00	no
All patients	Elevated ALT/AST	PGS000680	Cystatin C [mg/L]	-0.028	0.972	4.71E-01	0.152	1.164	0.025856	0.417735	1.00	no
All patients	Elevated ALT/AST	PGS000696	Testosterone [nmol/L]	-0.008	0.992	6.80E-01	0.068	1.071	0.422326	0.418543	1.00	adjusted
All patients	Elevated ALT/AST	PGS000687	IGF-1 [nmol/L]	-0.007	0.993	6.69E-01	0.099	1.104	0.068800	0.420782	1.00	no
All patients	Elevated ALT/AST	PGS000669	Albumin [g/L]	0.014	1.014	5.74E-01	0.048	1.049	0.560897	0.423325	1.00	adjusted
All patients	Hyperbilirubinemia	PGS000709	Heart failure	0.149	1.161	2.23E-01	0.079	1.083	0.989655	0.432238	1.00	no
All patients	Hyperbilirubinemia	PGS000699	Triglycerides [mmol/L]	0.104	1.110	8.96E-02	-0.109	0.897	0.650492	0.439695	1.00	adjusted

© 2022 Yang W et al. *JAMA Network Open*.

All patients	Hyperbilirubinemia	PGS000709	Heart failure	0.132	1.142	2.19E-01	0.083	1.086	0.953580	0.446416	1.00	adjusted
All patients	Elevated ALT/AST	PGS000692	Phosphate [mmol/L]	-0.041	0.960	4.98E-01	-0.008	0.992	0.732246	0.456412	1.00	adjusted
All patients	Elevated ALT/AST	PGS000670	Alkaline phosphatase [U/L]	-0.071	0.932	1.42E-01	0.083	1.086	0.496767	0.471137	1.00	no
All patients	Hyperbilirubinemia	PGS000684	Glucose [mmol/L]	-0.029	0.972	7.64E-01	-0.057	0.944	0.494889	0.476758	1.00	no
All patients	Hyperbilirubinemia	PGS000685	HbA1c [mmol/mol]	0.091	1.095	3.46E-01	-0.015	0.985	0.905007	0.483218	1.00	no
All patients	Hyperbilirubinemia	PGS000688	LDL cholesterol [mmol/L] (statin adjusted)	-0.119	0.888	3.26E-01	0.068	1.070	0.919350	0.486322	1.00	adjusted
All patients	Hyperbilirubinemia	PGS000673	Aspartate aminotransferase [U/L]	0.129	1.138	6.98E-02	-0.120	0.887	0.565825	0.489459	1.00	adjusted
All patients	Elevated ALT/AST	PGS000700	Urate [umol/L]	-0.030	0.971	4.28E-01	0.008	1.008	0.923078	0.490177	1.00	no
All patients	Elevated ALT/AST	PGS000681	Direct bilirubin [umol/L]	-0.022	0.978	8.29E-01	0.068	1.071	0.174387	0.496239	1.00	no
All patients	Hyperbilirubinemia	PGS000698	Total protein [g/L]	0.006	1.006	9.12E-01	-0.082	0.922	0.424463	0.496973	1.00	no
All patients	Hyperbilirubinemia	PGS000677	Cholesterol [mmol/L] (statin adjusted)	-0.101	0.904	5.44E-01	-0.007	0.993	0.707191	0.499787	1.00	no
All patients	Hyperbilirubinemia	PGS000708	Kidney failure	0.136	1.146	7.28E-02	0.001	1.001	0.505767	0.512224	1.00	no
All patients	Elevated ALT/AST	PGS000703	Angina	-0.055	0.946	3.90E-01	0.016	1.016	0.981110	0.512647	1.00	adjusted
All patients	Elevated ALT/AST	PGS000678	Creatinine [umol/L]	-0.008	0.992	3.81E-01	0.122	1.130	0.027834	0.523458	1.00	adjusted
All patients	Hyperbilirubinemia	PGS000675	C-reactive protein [mg/L]	0.166	1.181	1.39E-01	0.033	1.034	0.683847	0.528153	1.00	adjusted
All patients	Elevated ALT/AST	PGS000691	Non-albumin protein [g/L]	-0.006	0.994	6.44E-01	0.118	1.126	0.099381	0.529328	1.00	adjusted
All patients	Elevated ALT/AST	PGS000691	Non-albumin protein [g/L]	-0.003	0.997	7.29E-01	0.103	1.109	0.134584	0.529829	1.00	no
All patients	Elevated ALT/AST	PGS000686	HDL cholesterol [mmol/L]	0.043	1.043	2.37E-01	-0.056	0.945	0.612906	0.540031	1.00	adjusted
All patients	Elevated ALT/AST	PGS000670	Alkaline phosphatase [U/L]	-0.064	0.938	1.93E-01	0.070	1.073	0.523975	0.540265	1.00	adjusted
All patients	Hyperbilirubinemia	PGS000669	Albumin [g/L]	-0.122	0.885	5.64E-01	-0.032	0.969	0.759056	0.542439	1.00	adjusted
All patients	Elevated ALT/AST	PGS000686	HDL cholesterol [mmol/L]	0.041	1.042	2.24E-01	-0.058	0.944	0.568060	0.548953	1.00	no
All patients	Hyperbilirubinemia	PGS000698	Total protein [g/L]	0.015	1.016	9.99E-01	-0.035	0.966	0.454699	0.571215	1.00	adjusted
All patients	Elevated ALT/AST	PGS000678	Creatinine [umol/L]	-0.012	0.988	3.16E-01	0.124	1.132	0.024305	0.571218	1.00	no
All patients	Elevated ALT/AST	PGS000697	Total bilirubin [umol/L]	-0.018	0.982	8.53E-01	0.054	1.055	0.252438	0.571447	1.00	no
All patients	Hyperbilirubinemia	PGS000686	HDL cholesterol [mmol/L]	0.033	1.033	4.78E-01	-0.051	0.950	0.899775	0.57881	1.00	no
All patients	Hyperbilirubinemia	PGS000710	Myocardial infarction	-0.040	0.961	9.81E-01	0.041	1.041	0.456486	0.580657	1.00	no
All patients	Elevated ALT/AST	PGS000700	Urate [umol/L]	-0.021	0.979	5.59E-01	0.008	1.008	0.887204	0.581354	1.00	adjusted
All patients	Hyperbilirubinemia	PGS000711	Gout	-0.045	0.956	9.58E-01	-0.061	0.941	0.494255	0.58228	1.00	no
All patients	Hyperbilirubinemia	PGS000708	Kidney failure	0.117	1.124	7.33E-02	-0.033	0.967	0.415052	0.585326	1.00	adjusted
All patients	Hyperbilirubinemia	PGS000670	Alkaline phosphatase [U/L]	-0.122	0.885	1.73E-01	0.069	1.072	0.667372	0.587683	1.00	no
All patients	Hyperbilirubinemia	PGS000690	Microalbumin in urine [mg/L]	0.013	1.013	7.29E-01	0.191	1.211	0.315104	0.591274	1.00	no
All patients	Hyperbilirubinemia	PGS000699	Triglycerides [mmol/L]	0.102	1.108	1.27E-01	-0.099	0.906	0.522537	0.607697	1.00	no

© 2022 Yang W et al. *JAMA Network Open*.

All patients	Hyperbilirubinemia	PGS000710	Myocardial infarction	-0.032	0.969	8.91E-01	-0.014	0.986	0.583111	0.612563	1.00	adjusted
All patients	Hyperbilirubinemia	PGS000703	Angina	0.012	1.012	3.34E-01	-0.027	0.973	0.879023	0.612794	1.00	adjusted
All patients	Elevated ALT/AST	PGS000675	C-reactive protein [mg/L]	0.004	1.004	6.92E-01	-0.027	0.973	0.758855	0.616386	1.00	no
All patients	Hyperbilirubinemia	PGS000686	HDL cholesterol [mmol/L]	0.033	1.034	4.68E-01	-0.050	0.951	0.976808	0.621923	1.00	adjusted
All patients	Elevated ALT/AST	PGS000692	Phosphate [mmol/L]	-0.036	0.965	6.00E-01	0.000	1.000	0.908361	0.627584	1.00	no
All patients	Elevated ALT/AST	PGS000713	T2D	0.027	1.027	5.97E-01	-0.045	0.956	0.952259	0.654658	1.00	no
All patients	Elevated ALT/AST	PGS000685	HbA1c [mmol/mol]	0.009	1.009	9.24E-01	-0.115	0.891	0.419687	0.671395	1.00	no
All patients	Elevated ALT/AST	PGS000683	Gamma glutamyltransferase [U/L]	-0.004	0.996	9.40E-01	0.063	1.065	0.561331	0.672267	1.00	adjusted
All patients	Hyperbilirubinemia	PGS000693	Potassium in urine [mmol/L]	-0.012	0.988	4.17E-01	-0.113	0.893	0.861028	0.697885	1.00	adjusted
All patients	Elevated ALT/AST	PGS000697	Total bilirubin [umol/L]	-0.145	0.865	6.22E-01	0.145	1.156	0.984630	0.709179	1.00	adjusted
All patients	Hyperbilirubinemia	PGS000685	HbA1c [mmol/mol]	0.100	1.105	4.32E-01	-0.010	0.990	0.833968	0.725006	1.00	adjusted
All patients	Elevated ALT/AST	PGS000683	Gamma glutamyltransferase [U/L]	0.000	1.000	8.01E-01	0.043	1.044	0.807323	0.72698	1.00	no
All patients	Hyperbilirubinemia	PGS000690	Microalbumin in urine [mg/L]	-0.012	0.988	5.68E-01	0.182	1.199	0.339811	0.727007	1.00	adjusted
All patients	Hyperbilirubinemia	PGS000687	IGF-1 [nmol/L]	-0.084	0.919	3.69E-01	-0.045	0.956	0.765640	0.729012	1.00	no
All patients	Elevated ALT/AST	PGS000708	Kidney failure	-0.024	0.976	4.48E-01	0.092	1.096	0.129680	0.730922	1.00	adjusted
All patients	Elevated ALT/AST	PGS000713	T2D	0.018	1.019	7.64E-01	-0.034	0.967	0.864013	0.733728	1.00	adjusted
All patients	Hyperbilirubinemia	PGS000703	Angina	0.010	1.010	4.86E-01	-0.029	0.972	0.870532	0.747198	1.00	no
All patients	Hyperbilirubinemia	PGS000678	Creatinine [umol/L]	0.061	1.063	1.48E-01	0.004	1.004	0.391963	0.751624	1.00	adjusted
All patients	Hyperbilirubinemia	PGS000697	Total bilirubin [umol/L]	-0.056	0.945	9.48E-01	0.544	1.723	0.736979	0.765307	1.00	adjusted
All patients	Hyperbilirubinemia	PGS000711	Gout	-0.055	0.947	8.90E-01	-0.068	0.935	0.610410	0.76984	1.00	adjusted
All patients	Hyperbilirubinemia	PGS000693	Potassium in urine [mmol/L]	-0.038	0.963	3.28E-01	-0.024	0.977	0.664313	0.770329	1.00	no
All patients	Elevated ALT/AST	PGS000675	C-reactive protein [mg/L]	0.006	1.006	7.93E-01	-0.013	0.987	0.899957	0.776895	1.00	adjusted
All patients	Elevated ALT/AST	PGS000679	Creatinine in urine [umol/L]	-0.029	0.971	3.17E-01	0.071	1.073	0.408638	0.777092	1.00	no
All patients	Hyperbilirubinemia	PGS000670	Alkaline phosphatase [U/L]	-0.105	0.900	3.47E-01	0.050	1.051	0.667246	0.78392	1.00	adjusted
All patients	Elevated ALT/AST	PGS000682	eGFR [ml/min/1.73m2]	0.020	1.020	2.80E-01	-0.109	0.897	0.062464	0.790394	1.00	adjusted
All patients	Hyperbilirubinemia	PGS000678	Creatinine [umol/L]	0.079	1.082	1.32E-01	-0.002	0.998	0.330868	0.793614	1.00	no
All patients	Hyperbilirubinemia	PGS000701	Urea [mmol/L]	0.053	1.055	4.45E-01	-0.192	0.825	0.320748	0.794078	1.00	no
All patients	Elevated ALT/AST	PGS000704	Alcoholic cirrhosis	-0.016	0.985	7.15E-01	0.012	1.012	0.949593	0.807266	1.00	adjusted
All patients	Elevated ALT/AST	PGS000689	Lipoprotein A [nmol/L]	0.000	1.000	8.99E-01	0.017	1.017	0.817943	0.808497	1.00	no
All patients	Hyperbilirubinemia	PGS000682	eGFR [ml/min/1.73m2]	-0.067	0.935	2.55E-01	0.061	1.063	0.184121	0.8087	1.00	no
All patients	Elevated ALT/AST	PGS000679	Creatinine in urine [umol/L]	-0.023	0.977	4.03E-01	0.066	1.068	0.485054	0.818281	1.00	adjusted
All patients	Hyperbilirubinemia	PGS000687	IGF-1 [nmol/L]	-0.079	0.924	4.91E-01	-0.058	0.944	0.777447	0.821389	1.00	adjusted

© 2022 Yang W et al. *JAMA Network Open*.

All patients	Elevated ALT/AST	PGS000685	HbA1c [mmol/mol]	0.015	1.015	8.46E-01	-0.108	0.898	0.544546	0.824095	1.00	adjusted
All patients	Elevated ALT/AST	PGS000712	T2D (cases vs HbA1c filtered controls)	0.008	1.008	9.09E-01	-0.031	0.969	0.833428	0.825135	1.00	no
All patients	Hyperbilirubinemia	PGS000701	Urea [mmol/L]	0.066	1.069	3.20E-01	-0.237	0.789	0.255967	0.829333	1.00	adjusted
All patients	Hyperbilirubinemia	PGS000691	Non-albumin protein [g/L]	0.128	1.137	4.29E-01	-0.010	0.990	0.325049	0.834013	1.00	adjusted
All patients	Hyperbilirubinemia	PGS000682	eGFR [ml/min/1.73m2]	-0.045	0.956	2.88E-01	0.056	1.057	0.224501	0.840452	1.00	adjusted
All patients	Elevated ALT/AST	PGS000711	Gout	-0.004	0.996	8.89E-01	0.056	1.057	0.639122	0.860011	1.00	adjusted
All patients	Elevated ALT/AST	PGS000708	Kidney failure	-0.032	0.968	3.16E-01	0.098	1.103	0.119303	0.861006	1.00	no
All patients	Elevated ALT/AST	PGS000689	Lipoprotein A [nmol/L]	-0.001	0.999	9.66E-01	0.026	1.026	0.824760	0.864592	1.00	adjusted
All patients	Elevated ALT/AST	PGS000695	Sodium in urine [mmol/L]	-0.016	0.984	3.27E-01	0.034	1.035	0.133608	0.866904	1.00	adjusted

All patients	Elevated ALT/AST	PGS000682	eGFR [ml/min/1.73m2]	0.024	1.025	2.21E-01	-0.108	0.897	0.060224	0.873734	1.00	no
All patients	Elevated ALT/AST	PGS000711	Gout	-0.018	0.982	6.09E-01	0.052	1.054	0.681308	0.876241	1.00	no
All patients	Elevated ALT/AST	PGS000681	Direct bilirubin [umol/L]	-0.183	0.832	4.66E-01	0.269	1.309	0.502603	0.876336	1.00	adjusted
All patients	Elevated ALT/AST	PGS000712	T2D (cases vs HbA1c filtered controls)	0.001	1.001	9.36E-01	-0.020	0.981	0.738764	0.882288	1.00	adjusted
All patients	Elevated ALT/AST	PGS000676	Calcium [mmol/L]	-0.036	0.964	3.55E-01	0.051	1.053	0.344771	0.883664	1.00	no
All patients	Hyperbilirubinemia	PGS000691	Non-albumin protein [g/L]	0.108	1.115	4.81E-01	0.012	1.012	0.414146	0.884359	1.00	no
All patients	Elevated ALT/AST	PGS000690	Microalbumin in urine [mg/L]	-0.006	0.994	8.64E-01	0.030	1.031	0.990160	0.886921	1.00	adjusted
All patients	Elevated ALT/AST	PGS000690	Microalbumin in urine [mg/L]	-0.005	0.995	9.16E-01	0.028	1.029	0.945460	0.900707	1.00	no
All patients	Elevated ALT/AST	PGS000701	Urea [mmol/L]	0.033	1.033	5.48E-01	-0.066	0.936	0.343464	0.911362	1.00	no
All patients	Elevated ALT/AST	PGS000684	Glucose [mmol/L]	-0.003	0.997	7.98E-01	-0.061	0.941	0.617726	0.920867	1.00	no
All patients	Elevated ALT/AST	PGS000701	Urea [mmol/L]	0.039	1.040	4.62E-01	-0.075	0.927	0.289155	0.93775	1.00	adjusted
All patients	Hyperbilirubinemia	PGS000677	Cholesterol [mmol/L] (statin adjusted)	-0.060	0.941	9.32E-01	0.005	1.005	0.984060	0.944769	1.00	adjusted
All patients	Hyperbilirubinemia	PGS000694	SHBG [nmol/L]	-0.023	0.978	7.07E-01	-0.087	0.917	0.686727	0.960838	1.00	adjusted
All patients	Elevated ALT/AST	PGS000706	Hypertension	0.011	1.011	7.16E-01	-0.014	0.986	0.588248	0.961641	1.00	no
All patients	Elevated ALT/AST	PGS000676	Calcium [mmol/L]	-0.030	0.971	4.16E-01	0.056	1.057	0.333496	0.96684	1.00	adjusted
All patients	Elevated ALT/AST	PGS000706	Hypertension	0.006	1.006	7.52E-01	-0.018	0.982	0.644581	0.970348	1.00	adjusted
All patients	Hyperbilirubinemia	PGS000694	SHBG [nmol/L]	-0.004	0.996	5.25E-01	-0.103	0.902	0.553528	0.983291	1.00	no
All patients	Elevated ALT/AST	PGS000695	Sodium in urine [mmol/L]	-0.021	0.979	2.52E-01	0.037	1.038	0.139929	0.986139	1.00	no
All patients	Elevated ALT/AST	PGS000684	Glucose [mmol/L]	-0.005	0.995	8.41E-01	-0.054	0.947	0.793574	0.999526	1.00	adjusted
European Only	Hyperbilirubinemia	PGS000697	Total bilirubin [umol/L]	0.394	1.483	0.000	0.587	1.798	8.2E-07	1.3E-14	5.98E-13	no
European Only	Hyperbilirubinemia	PGS000681	Direct bilirubin [umol/L]	0.403	1.496	0.000	0.575	1.778	1.4E-06	2.4E-14	1.11E-12	no
European Only	Elevated ALT/AST	PGS000668	Alanine aminotransferase [U/L]	0.090	1.095	0.013	0.133	1.142	3.0E-02	1.0E-03	0.05	no
European Only	Elevated ALT/AST	PGS000673	Aspartate aminotransferase [U/L]	0.067	1.069	0.028	0.090	1.094	6.7E-02	4.2E-03	0.19	no

© 2022 Yang W et al. *JAMA Network Open*.

European Only	Elevated ALT/AST	PGS000669	Albumin [g/L]	0.075	1.078	0.037	0.101	1.107	2.5E-01	1.9E-02	0.88	no
European Only	Hyperbilirubinemia	PGS000683	Gamma glutamyltransferase [U/L]	0.214	1.239	0.018	0.176	1.193	3.4E-01	2.3E-02	1.00	no
European Only	Elevated ALT/AST	PGS000705	Gallstones	0.057	1.058	0.150	0.123	1.131	9.1E-02	2.9E-02	1.00	no
European Only	Elevated ALT/AST	PGS000687	IGF-1 [nmol/L]	0.085	1.088	0.074	0.048	1.049	3.5E-01	4.7E-02	1.00	no
European Only	Hyperbilirubinemia	PGS000668	Alanine aminotransferase [U/L]	0.130	1.139	0.010	0.159	1.172	8.7E-01	6.9E-02	1.00	no
European Only	Hyperbilirubinemia	PGS000707	Cholecystitis	0.172	1.187	0.035	0.095	1.100	5.5E-01	6.9E-02	1.00	no
European Only	Elevated ALT/AST	PGS000707	Cholecystitis	0.008	1.008	0.505	0.152	1.164	4.5E-02	7.4E-02	1.00	no
European Only	Hyperbilirubinemia	PGS000713	T2D	-0.014	0.987	0.685	0.226	1.254	4.9E-02	7.6E-02	1.00	no
European Only	Elevated ALT/AST	PGS000680	Cystatin C [mg/L]	-0.007	0.993	0.761	0.214	1.239	2.4E-03	8.8E-02	1.00	no
European Only	Hyperbilirubinemia	PGS000699	Triglycerides [mmol/L]	0.088	1.092	0.054	0.011	1.011	6.3E-01	1.0E-01	1.00	no
European Only	Hyperbilirubinemia	PGS000692	Phosphate [mmol/L]	0.109	1.115	0.474	0.217	1.242	1.3E-01	1.1E-01	1.00	no
European Only	Elevated ALT/AST	PGS000709	Heart failure	-0.078	0.925	0.081	-0.065	0.937	7.1E-01	1.1E-01	1.00	no
European Only	Hyperbilirubinemia	PGS000674	AST to ALT ratio	-0.104	0.901	0.083	-0.263	0.769	5.5E-01	1.1E-01	1.00	no
European Only	Hyperbilirubinemia	PGS000702	Vitamin D [nmol/L]	-0.026	0.974	0.360	-0.185	0.831	2.5E-01	1.4E-01	1.00	no
European Only	Elevated ALT/AST	PGS000706	Hypertension	-0.055	0.947	0.300	-0.069	0.933	3.1E-01	1.5E-01	1.00	no
European Only	Elevated ALT/AST	PGS000676	Calcium [mmol/L]	0.019	1.020	0.679	0.119	1.127	7.4E-02	1.5E-01	1.00	no
European Only	Hyperbilirubinemia	PGS000712	T2D (cases vs HbA1c filtered controls)	-0.042	0.959	0.993	0.228	1.256	6.5E-02	1.6E-01	1.00	no
European Only	Elevated ALT/AST	PGS000698	Total protein [g/L]	0.018	1.018	0.650	0.161	1.174	9.0E-02	1.6E-01	1.00	no
European Only	Hyperbilirubinemia	PGS000700	Urate [umol/L]	-0.013	0.987	0.507	-0.208	0.812	2.2E-01	1.7E-01	1.00	no
European Only	Elevated ALT/AST	PGS000674	AST to ALT ratio	-0.053	0.948	0.268	-0.060	0.942	4.5E-01	1.8E-01	1.00	no
European Only	Elevated ALT/AST	PGS000678	Creatinine [umol/L]	0.017	1.017	0.684	0.177	1.193	7.2E-03	1.8E-01	1.00	no
European Only	Elevated ALT/AST	PGS000701	Urea [mmol/L]	0.058	1.060	0.242	0.047	1.048	5.3E-01	1.9E-01	1.00	no
European Only	Hyperbilirubinemia	PGS000680	Cystatin C [mg/L]	0.128	1.136	0.194	0.063	1.065	5.7E-01	2.0E-01	1.00	no
European Only	Hyperbilirubinemia	PGS000678	Creatinine [umol/L]	0.116	1.123	0.155	0.106	1.111	7.2E-01	2.2E-01	1.00	no
European Only	Elevated ALT/AST	PGS000693	Potassium in urine [mmol/L]	-0.059	0.943	0.197	0.031	1.031	7.8E-01	2.5E-01	1.00	no
European Only	Hyperbilirubinemia	PGS000684	Glucose [mmol/L]	-0.112	0.894	0.304	-0.089	0.915	5.3E-01	2.5E-01	1.00	no
European Only	Elevated ALT/AST	PGS000702	Vitamin D [nmol/L]	-0.002	0.998	0.664	-0.049	0.952	2.0E-01	2.5E-01	1.00	no
European Only	Elevated ALT/AST	PGS000708	Kidney failure	-0.019	0.981	0.480	0.159	1.173	8.9E-03	2.7E-01	1.00	no
European Only	Elevated ALT/AST	PGS000694	SHBG [nmol/L]	0.084	1.087	0.182	0.023	1.023	9.4E-01	2.7E-01	1.00	no
European Only	Elevated ALT/AST	PGS000713	T2D	0.041	1.042	0.456	-0.007	0.993	4.3E-01	2.8E-01	1.00	no
European Only	Hyperbilirubinemia	PGS000679	Creatinine in urine [umol/L]	0.007	1.007	0.831	0.183	1.201	1.1E-01	2.8E-01	1.00	no
European Only	Hyperbilirubinemia	PGS000706	Hypertension	0.087	1.090	0.491	0.068	1.070	4.3E-01	2.9E-01	1.00	no

© 2022 Yang W et al. *JAMA Network Open*.

European Only	Hyperbilirubinemia	PGS000709	Heart failure	0.256	1.291	0.292	0.018	1.019	6.8E-01	3.2E-01	1.00	no
European Only	Elevated ALT/AST	PGS000675	C-reactive protein [mg/L]	-0.017	0.984	0.441	-0.039	0.962	5.2E-01	3.2E-01	1.00	no
European Only	Hyperbilirubinemia	PGS000704	Alcoholic cirrhosis	0.184	1.202	0.035	-0.012	0.988	5.9E-01	3.3E-01	1.00	no
European Only	Elevated ALT/AST	PGS000670	Alkaline phosphatase [U/L]	-0.019	0.981	0.697	0.142	1.152	2.8E-01	3.3E-01	1.00	no
European Only	Elevated ALT/AST	PGS000696	Testosterone [nmol/L]	0.039	1.040	0.336	0.037	1.037	7.7E-01	3.5E-01	1.00	no
European Only	Hyperbilirubinemia	PGS000708	Kidney failure	0.143	1.153	0.273	0.028	1.028	7.7E-01	3.5E-01	1.00	no
European Only	Elevated ALT/AST	PGS000682	eGFR [ml/min/1.73m2]	-0.002	0.998	0.504	-0.147	0.864	1.9E-02	3.6E-01	1.00	no
European Only	Hyperbilirubinemia	PGS000696	Testosterone [nmol/L]	-0.168	0.846	0.394	-0.077	0.926	6.9E-01	3.9E-01	1.00	no
European Only	Hyperbilirubinemia	PGS000670	Alkaline phosphatase [U/L]	-0.109	0.897	0.514	0.001	1.001	5.7E-01	3.9E-01	1.00	no
European Only	Elevated ALT/AST	PGS000712	T2D (cases vs HbA1c filtered controls)	0.007	1.007	0.874	0.023	1.023	2.6E-01	4.1E-01	1.00	no
European Only	Hyperbilirubinemia	PGS000675	C-reactive protein [mg/L]	0.202	1.224	0.223	0.066	1.068	9.6E-01	4.2E-01	1.00	no
European Only	Hyperbilirubinemia	PGS000682	eGFR [ml/min/1.73m2]	-0.108	0.898	0.290	-0.038	0.962	9.5E-01	4.5E-01	1.00	no
European Only	Hyperbilirubinemia	PGS000711	Gout	0.003	1.003	0.869	-0.163	0.849	4.1E-01	4.6E-01	1.00	no

European Only	Hyperbilirubinemia	PGS000671	Apolipoprotein A [g/L]	0.082	1.085	0.324	0.080	1.083	9.3E-01	4.7E-01	1.00	no
European Only	Elevated ALT/AST	PGS000681	Direct bilirubin [umol/L]	-0.103	0.902	0.094	0.086	1.090	3.4E-01	4.9E-01	1.00	no
European Only	Elevated ALT/AST	PGS000697	Total bilirubin [umol/L]	-0.095	0.909	0.112	0.083	1.086	3.7E-01	5.0E-01	1.00	no
European Only	Elevated ALT/AST	PGS000700	Urate [umol/L]	-0.033	0.967	0.370	0.008	1.008	9.7E-01	5.0E-01	1.00	no
European Only	Elevated ALT/AST	PGS000703	Angina	-0.067	0.936	0.482	-0.032	0.969	8.9E-01	5.3E-01	1.00	no
European Only	Hyperbilirubinemia	PGS000673	Aspartate aminotransferase [U/L]	0.136	1.146	0.011	-0.194	0.824	2.2E-01	5.4E-01	1.00	no
European Only	Hyperbilirubinemia	PGS000689	Lipoprotein A [nmol/L]	0.088	1.091	0.659	0.191	1.210	6.7E-01	5.4E-01	1.00	no
European Only	Hyperbilirubinemia	PGS000705	Gallstones	0.012	1.012	0.365	-0.071	0.931	9.9E-01	5.5E-01	1.00	no
European Only	Hyperbilirubinemia	PGS000676	Calcium [mmol/L]	0.054	1.055	0.476	0.201	1.222	8.7E-01	5.5E-01	1.00	no
European Only	Elevated ALT/AST	PGS000695	Sodium in urine [mmol/L]	-0.028	0.973	0.202	-0.010	0.990	5.1E-01	5.6E-01	1.00	no
European Only	Elevated ALT/AST	PGS000710	Myocardial infarction	-0.052	0.949	0.711	-0.061	0.941	6.5E-01	5.7E-01	1.00	no
European Only	Elevated ALT/AST	PGS000704	Alcoholic cirrhosis	-0.077	0.926	0.316	0.007	1.007	6.9E-01	5.9E-01	1.00	no
European Only	Elevated ALT/AST	PGS000686	HDL cholesterol [mmol/L]	0.014	1.015	0.615	-0.109	0.896	1.8E-01	6.6E-01	1.00	no
European Only	Elevated ALT/AST	PGS000692	Phosphate [mmol/L]	-0.032	0.968	0.940	0.027	1.027	4.4E-01	6.6E-01	1.00	no
European Only	Hyperbilirubinemia	PGS000672	Apolipoprotein B [g/L] (statin adjusted)	-0.151	0.860	0.387	0.062	1.063	8.8E-01	6.6E-01	1.00	no
European Only	Elevated ALT/AST	PGS000677	Cholesterol [mmol/L] (statin adjusted)	0.007	1.007	0.814	-0.054	0.947	3.6E-01	7.0E-01	1.00	no
European Only	Elevated ALT/AST	PGS000699	Triglycerides [mmol/L]	-0.016	0.984	0.983	-0.070	0.933	5.5E-01	7.0E-01	1.00	no
European Only	Elevated ALT/AST	PGS000684	Glucose [mmol/L]	0.004	1.004	0.639	-0.044	0.957	9.8E-01	7.3E-01	1.00	no
European Only	Elevated ALT/AST	PGS000690	Microalbumin in urine [mg/L]	0.017	1.017	0.594	0.033	1.033	9.2E-01	7.3E-01	1.00	no

© 2022 Yang W et al. *JAMA Network Open*.

European Only	Elevated ALT/AST	PGS000691	Non-albumin protein [g/L]	-0.023	0.978	0.312	0.164	1.178	6.9E-02	7.4E-01	1.00	no
European Only	Elevated ALT/AST	PGS000683	Gamma glutamyltransferase [U/L]	-0.037	0.964	0.598	0.002	1.002	8.4E-01	7.8E-01	1.00	no
European Only	Hyperbilirubinemia	PGS000698	Total protein [g/L]	0.051	1.052	0.308	-0.080	0.923	5.7E-01	8.1E-01	1.00	no
European Only	Hyperbilirubinemia	PGS000687	IGF-1 [nmol/L]	-0.098	0.906	0.572	-0.027	0.974	4.4E-01	8.3E-01	1.00	no
European Only	Hyperbilirubinemia	PGS000691	Non-albumin protein [g/L]	0.052	1.053	0.520	-0.117	0.890	4.0E-01	8.3E-01	1.00	no
European Only	Elevated ALT/AST	PGS000711	Gout	-0.021	0.979	0.527	0.040	1.041	6.5E-01	8.3E-01	1.00	no
European Only	Hyperbilirubinemia	PGS000703	Angina	-0.086	0.917	0.753	0.043	1.044	1.0E+00	8.4E-01	1.00	no
European Only	Elevated ALT/AST	PGS000685	HbA1c [mmol/mol]	0.026	1.026	0.581	-0.100	0.905	6.7E-01	8.7E-01	1.00	no
European Only	Hyperbilirubinemia	PGS000710	Myocardial infarction	-0.034	0.966	0.871	0.014	1.014	7.3E-01	8.7E-01	1.00	no
European Only	Hyperbilirubinemia	PGS000685	HbA1c [mmol/mol]	-0.077	0.926	0.643	0.126	1.134	5.5E-01	8.7E-01	1.00	no
European Only	Hyperbilirubinemia	PGS000701	Urea [mmol/L]	0.109	1.115	0.815	-0.141	0.869	6.8E-01	8.8E-01	1.00	no
European Only	Elevated ALT/AST	PGS000679	Creatinine in urine [umol/L]	-0.045	0.956	0.192	0.090	1.095	1.7E-01	8.8E-01	1.00	no
European Only	Elevated ALT/AST	PGS000689	Lipoprotein A [nmol/L]	-0.021	0.979	0.908	0.029	1.029	7.1E-01	8.9E-01	1.00	no
European Only	Hyperbilirubinemia	PGS000695	Sodium in urine [mmol/L]	0.117	1.124	0.319	-0.050	0.951	4.9E-01	9.0E-01	1.00	no
European Only	Hyperbilirubinemia	PGS000686	HDL cholesterol [mmol/L]	0.046	1.047	0.823	0.000	1.000	7.2E-01	9.0E-01	1.00	no
European Only	Hyperbilirubinemia	PGS000694	SHBG [nmol/L]	0.067	1.069	0.176	-0.177	0.838	1.9E-01	9.3E-01	1.00	no
European Only	Elevated ALT/AST	PGS000688	LDL cholesterol [mmol/L] (statin adjusted)	0.016	1.016	0.726	-0.023	0.978	5.8E-01	9.3E-01	1.00	no
European Only	Hyperbilirubinemia	PGS000677	Cholesterol [mmol/L] (statin adjusted)	-0.097	0.907	0.888	-0.033	0.968	9.8E-01	9.4E-01	1.00	no
European Only	Hyperbilirubinemia	PGS000669	Albumin [g/L]	-0.039	0.962	0.701	0.106	1.112	6.8E-01	9.5E-01	1.00	no
European Only	Elevated ALT/AST	PGS000672	Apolipoprotein B [g/L] (statin adjusted)	0.003	1.003	0.970	0.000	1.000	8.9E-01	9.6E-01	1.00	no
European Only	Elevated ALT/AST	PGS000671	Apolipoprotein A [g/L]	-0.012	0.988	0.746	-0.049	0.953	7.2E-01	9.8E-01	1.00	no
European Only	Hyperbilirubinemia	PGS000690	Microalbumin in urine [mg/L]	0.028	1.028	0.802	0.172	1.187	8.6E-01	9.8E-01	1.00	no
European Only	Hyperbilirubinemia	PGS000688	LDL cholesterol [mmol/L] (statin adjusted)	-0.126	0.881	0.618	0.072	1.075	7.0E-01	9.9E-01	1.00	no
European Only	Hyperbilirubinemia	PGS000693	Potassium in urine [mmol/L]	-0.030	0.971	0.435	-0.055	0.947	5.3E-01	1.0E+00	1.00	no

© 2022 Yang W et al. *JAMA Network Open*.

eTable 5. Multivariable analysis including both ancestral group and genotypes.

AALL0232, Hyperbilirubinemia		Without adjusting for UGT1A1		After adjusting for UGT1A1	
		Odds Ratio (CI)	p-value	Odds Ratio (CI)	p-value
Genetic Ancestral Group	European	ref			
	African	0.41 (0.17-0.93)	0.032	0.30 (0.13-0.71)	0.0058
	Admixed American/Latino	1.61 (1.19-2.16)	0.0017	1.36 (0.99-1.86)	0.051
	Asian	1.8 (0.92-3.47)	0.088	2.16 (1.13-4.13)	0.02
	Other	0.84 (0.51-1.4)	0.51	0.84 (0.51-1.39)	0.50
AALL0434, Hyperbilirubinemia					
Genetic Ancestral Group	European	ref			
	African	0.69 (0.36-1.32)	0.26	0.55 (0.29-1.07)	0.079
	Admixed American/Latino	1.30 (0.79-2.13)	0.3	1.2 (0.72-2.01)	0.49
	Asian	1.75 (0.88-3.48)	0.11	2.1 (1.06-4.14)	0.033
	Other	1.12 (0.48-2.58)	0.8	1.04 (0.43-2.54)	0.93
AALL0232 elevated ALT/AST		Without adjusting for PNPLA3 and TPMT		After adjusting for PNPLA3 and TPMT	
		Odds Ratio (CI)	p-value	Odds Ratio (CI)	p-value
Genetic Ancestral Group	European	ref			
	African	0.34 (0.22-0.54)	6.40E-06	0.36 (0.23-0.575)	1.6e-5
	Admixed American/Latino	1.57 (1.29-1.91)	6.90E-06	1.35 (1.1-1.66)	0.004
	Asian	1.58 (0.98-2.54)	0.056	1.52 (0.95-2.43)	0.078
	Other	1.11 (0.8-1.47)	0.49	1.09 (0.83-1.45)	0.53
AALL0434 elevated ALT/AST					
Genetic Ancestral Group	European	ref			
	African	0.40 (0.26-0.63)	5.50E-05	0.44 (0.28-0.69)	0.00032
	Admixed American/Latino	1.19 (0.84-1.67)	0.33	0.97 (0.67-1.39)	0.85
	Asian	1.43 (0.85-2.43)	0.18	1.38 (0.82-2.32)	0.23
	Other	1.71 (0.99-2.93)	0.052	1.72 (1.0-2.94)	0.050

© 2022 Yang W et al. *JAMA Network Open*.

eFigure 1. Cumulative incidence of hepatotoxicities in AALL0232 and AALL0434. Blue: age >=10 yrs; Green: age < 10 yrs; Gray: all patients.

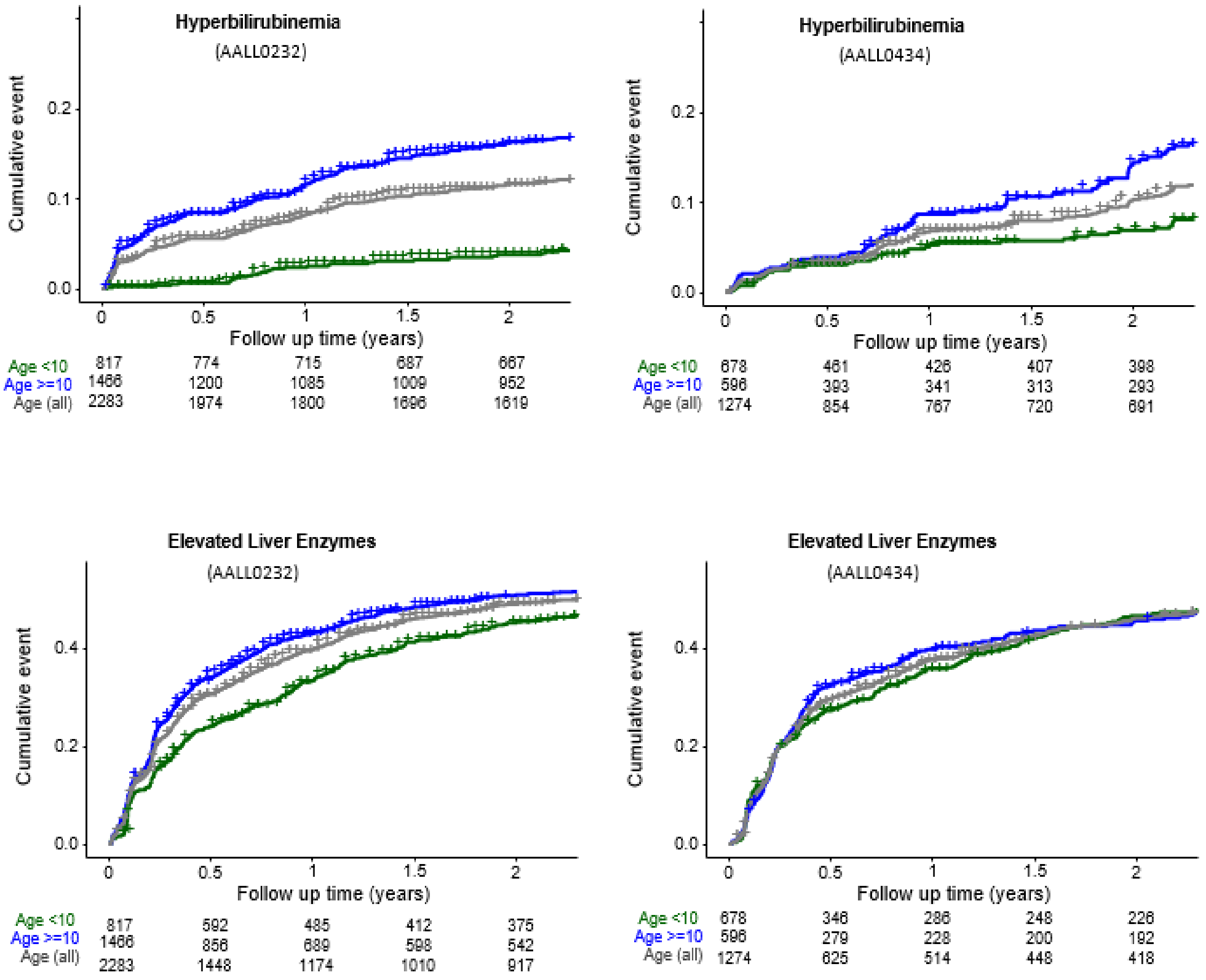

© 2022 Yang W et al. *JAMA Network Open*.

eFigure 2. Proportion with toxicities by age. Dark bar indicates older patients (age >= 10 yrs) and light gray indicates younger patients (age < 10 yrs).

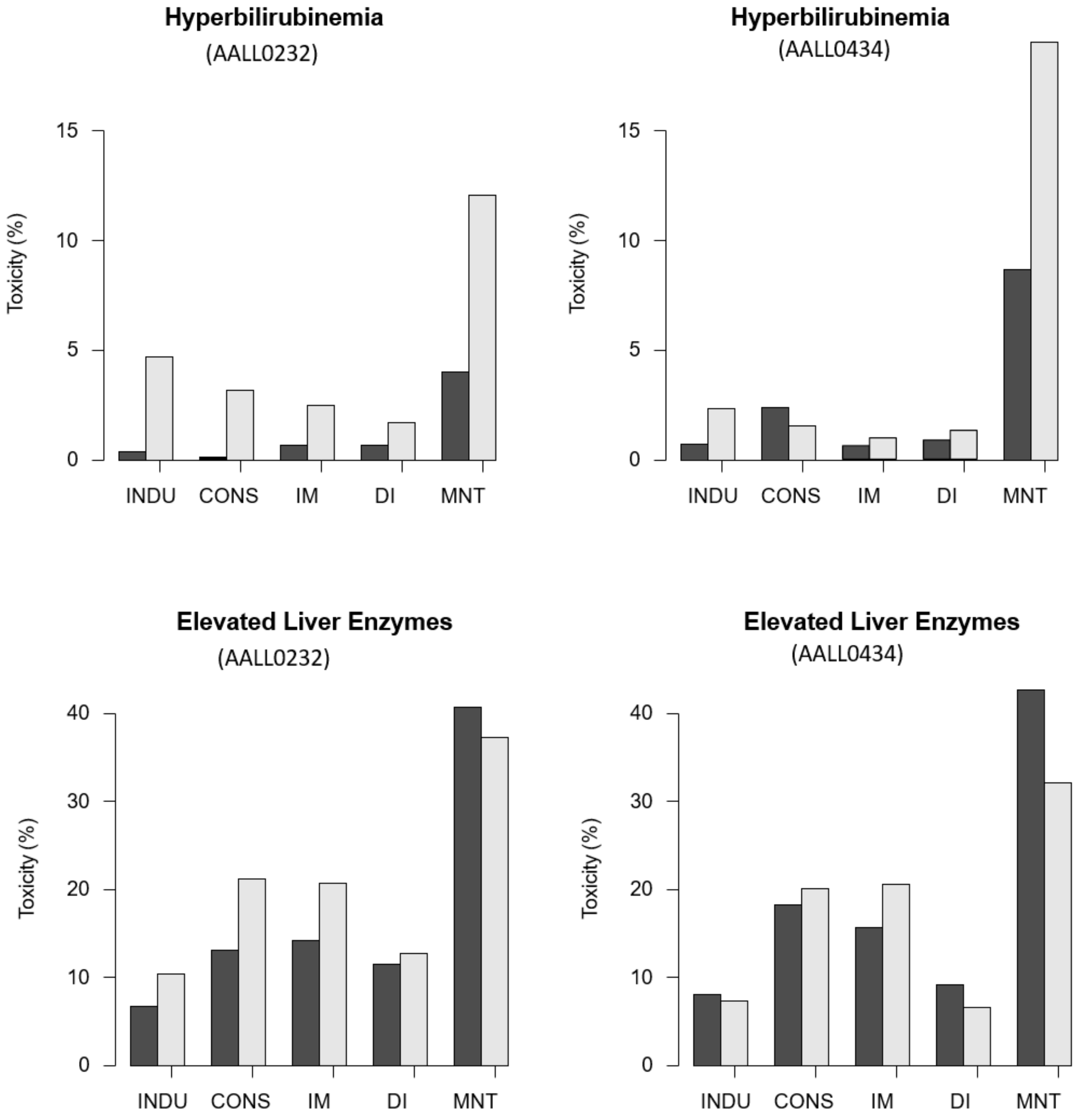

© 2022 Yang W et al. *JAMA Network Open*.

eFigure 3. Forest plot of the association between top PRS with Hyperbilirubinemia (A) and elevated ALT/AST (B). Odds ratios indicate the change of one standard deviation in PRS score. PGS000687 (3,105 SNPs) and PGS000668 (12,077 SNPs) were obtained from PGS catalog.

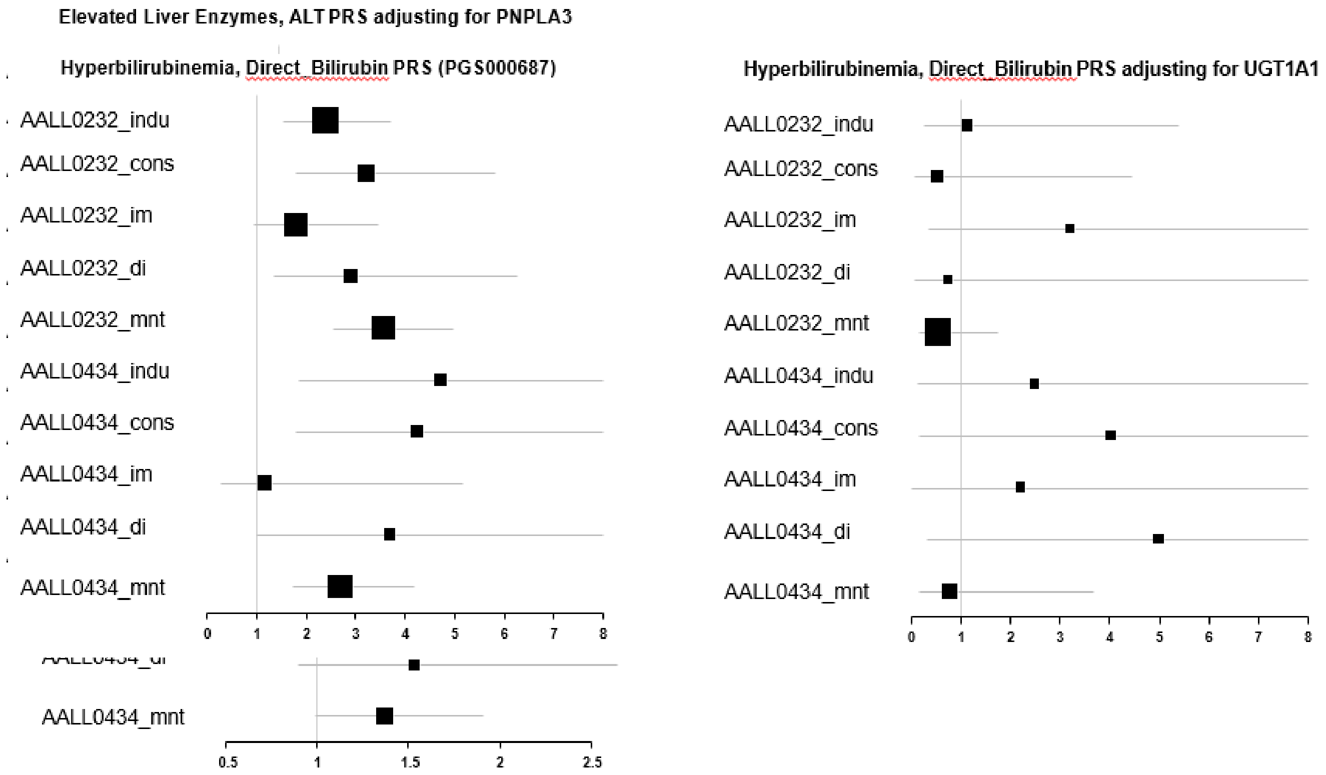

© 2022 Yang W et al. *JAMA Network Open*.

eFigure 4. GWAS of A) hyperbilirubinemia and B) elevated ALT/AST. Mixed-effects logistic regression model across all treatment phases.

A

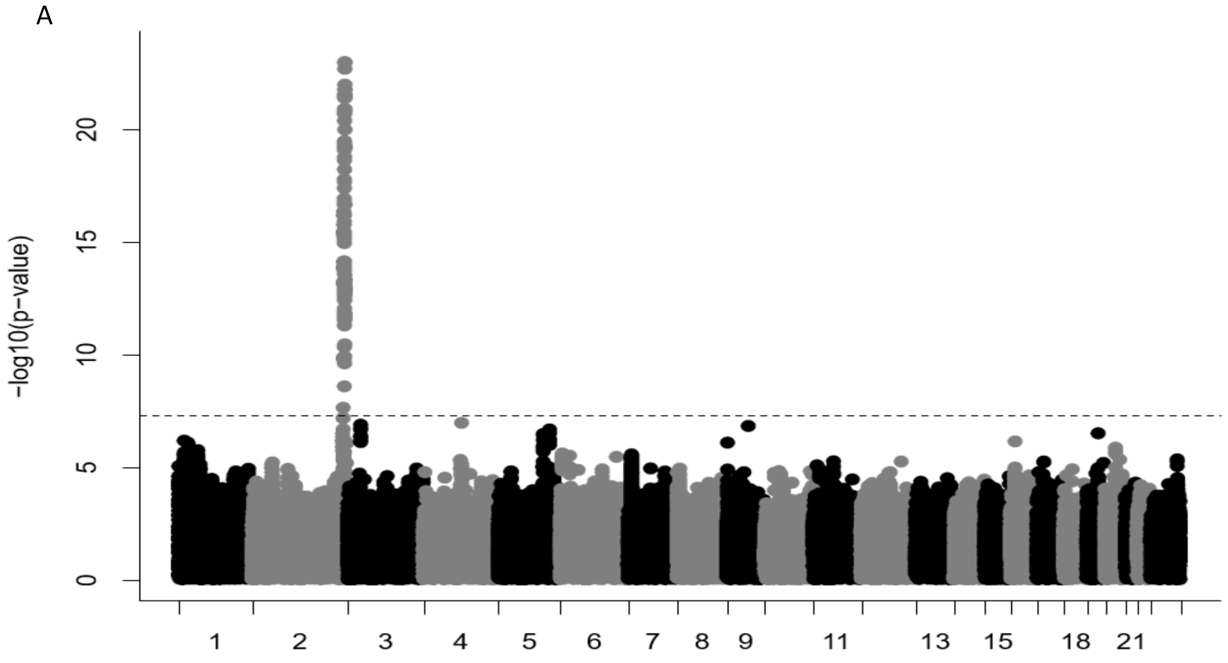

B

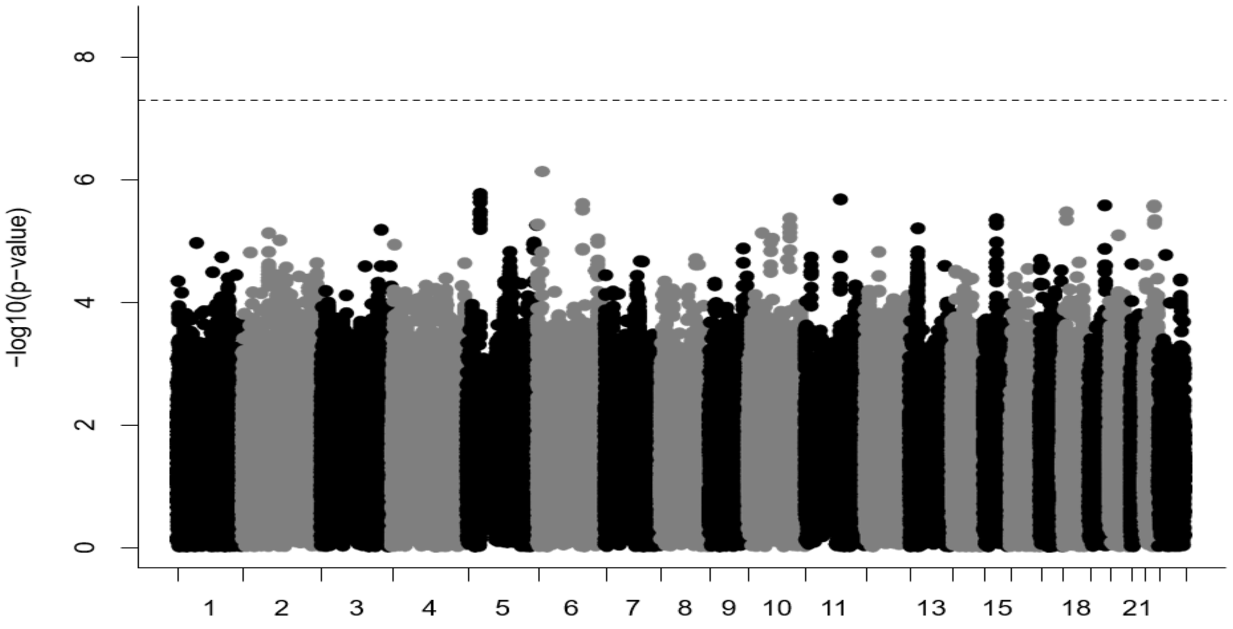

© 2022 Yang W et al. *JAMA Network Open*.

eFigure 5. Locus Zoom for *UGT1A1* and hyperbilirubinemia based on results from meta-analysis combining both AALL0232 and AALL0434. Mixed effects logistic regression model across all treatment phases.

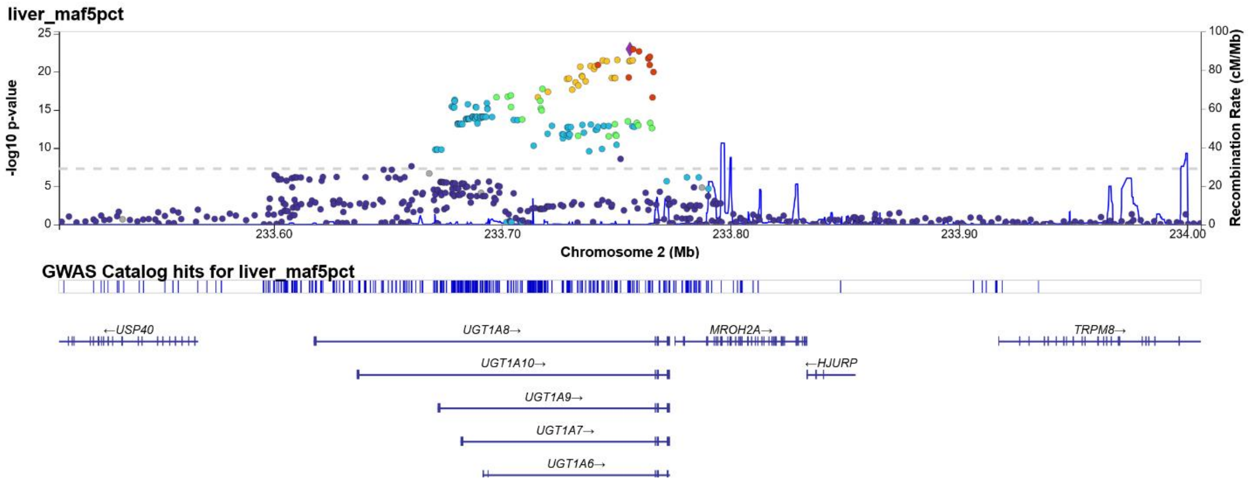

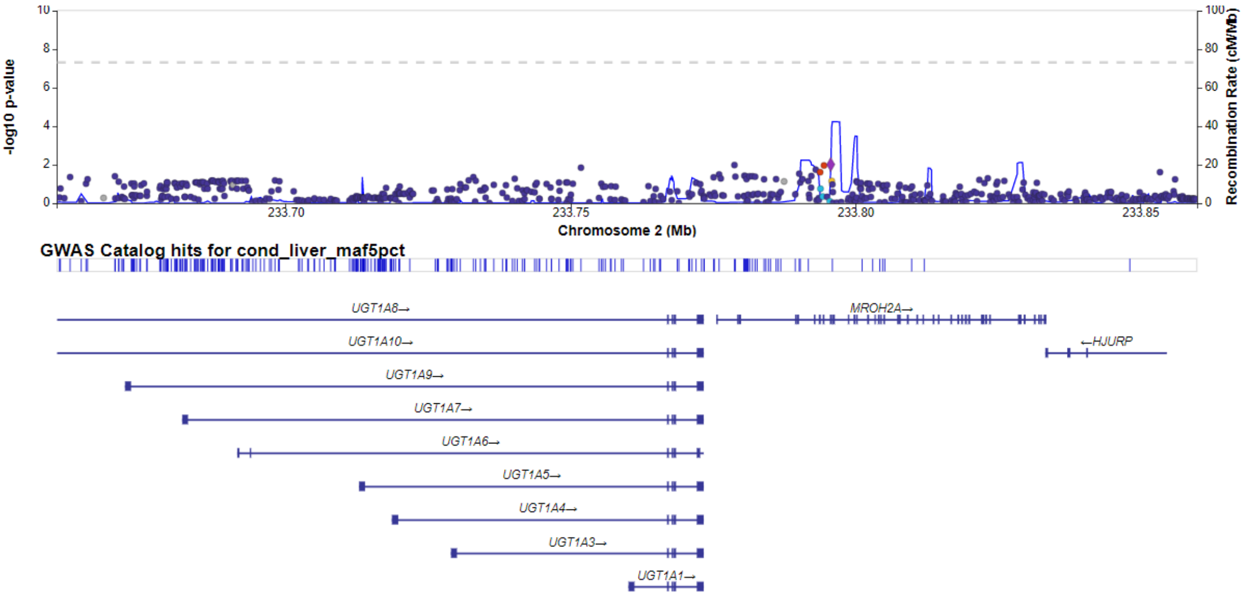

© 2022 Yang W et al. *JAMA Network Open*.

eFigure 6. Association of rs12283870 genotypes and elevated ALT/AST by age and treatment phase. Y-axis indicates the percentage of patients who experienced elevated enzyme toxicity within each treatment phase.

Age < 10

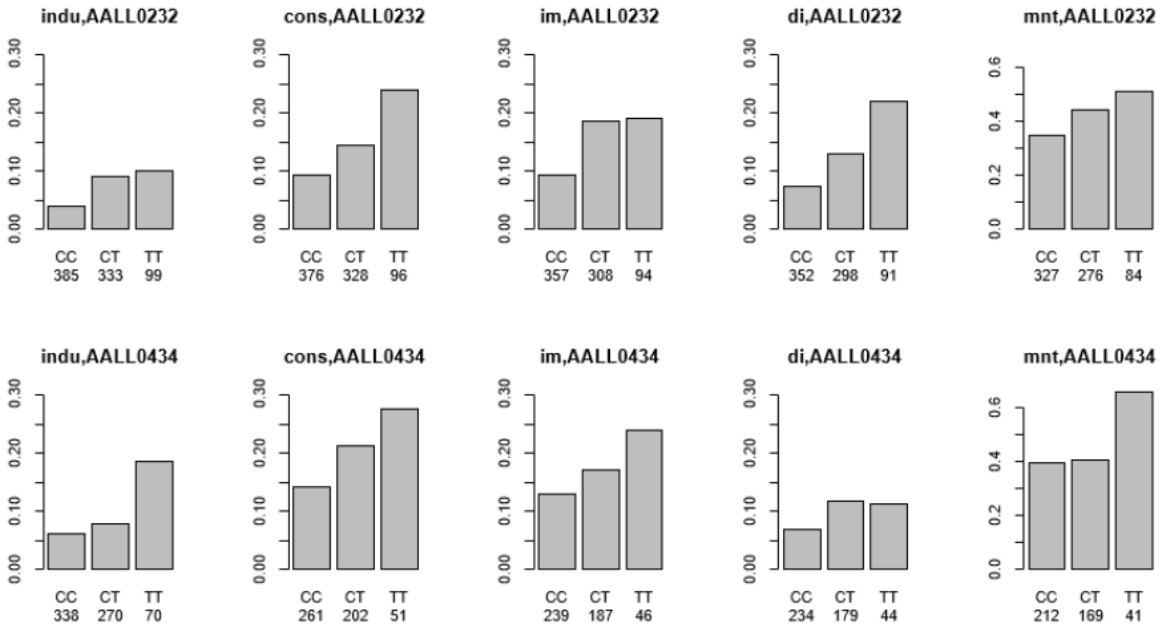

Age >= 10

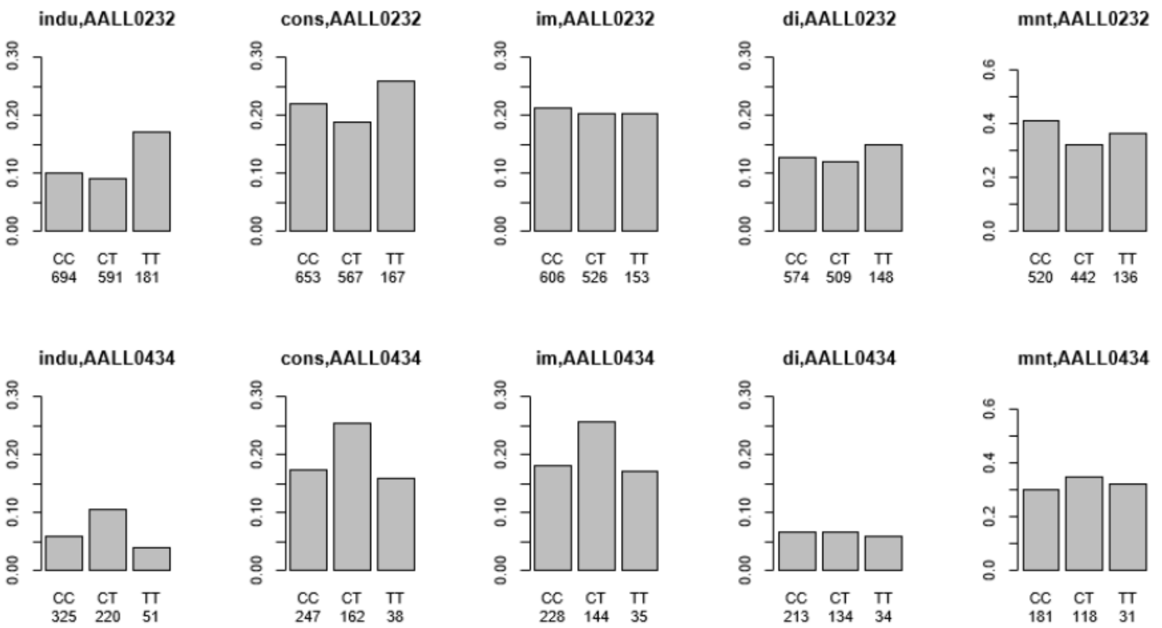

© 2022 Yang W et al. *JAMA Network Open*.

eFigure 7. Locus Zoom for *PNPLA3* and elevated ALT/AST based on results from meta-analysis combining both AALL0232 and AALL0434

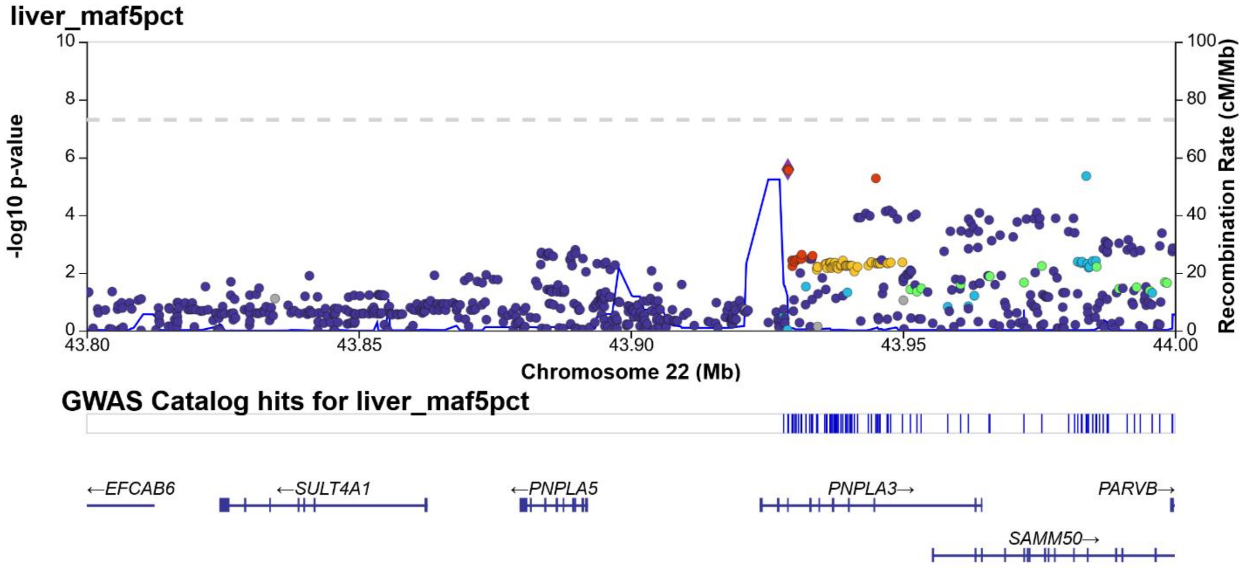

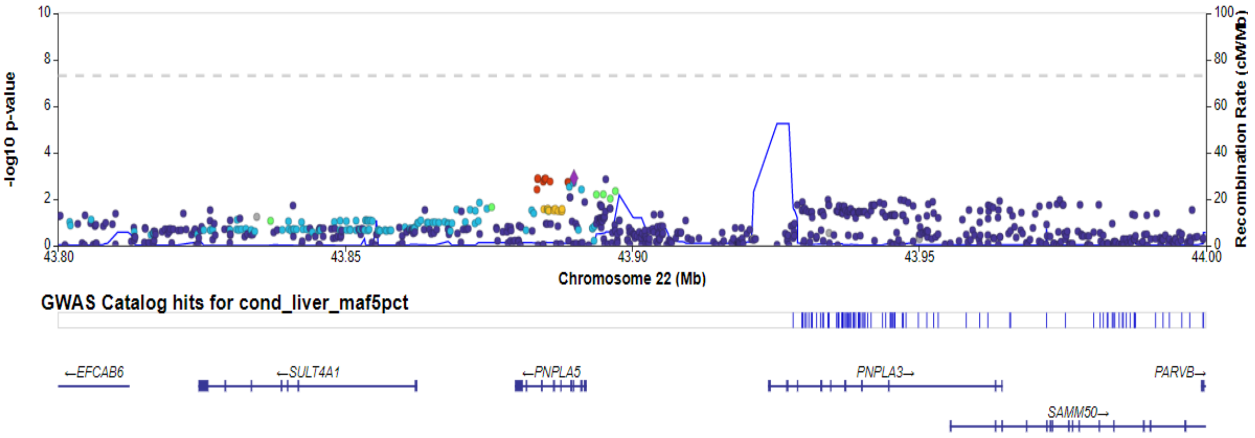

© 2022 Yang W et al. *JAMA Network Open*.

eFigure 8. (A). Locus Zoom for rs12283870 and elevated ALT/AST (age < 10) based on results from meta-analysis combining both AALL0232 and AALL0434; (B). GTEx portal (https://gtexportal.org/home/snp/rs12283870, retrieved on 2022-02-07) showed that rs12283870 is an eQTL for multiple genes including *IGHMBP2* across multiple tissue types; (C). GTEx data showed that rs12283870 is a splice-QTL for *CPT1A*.

A

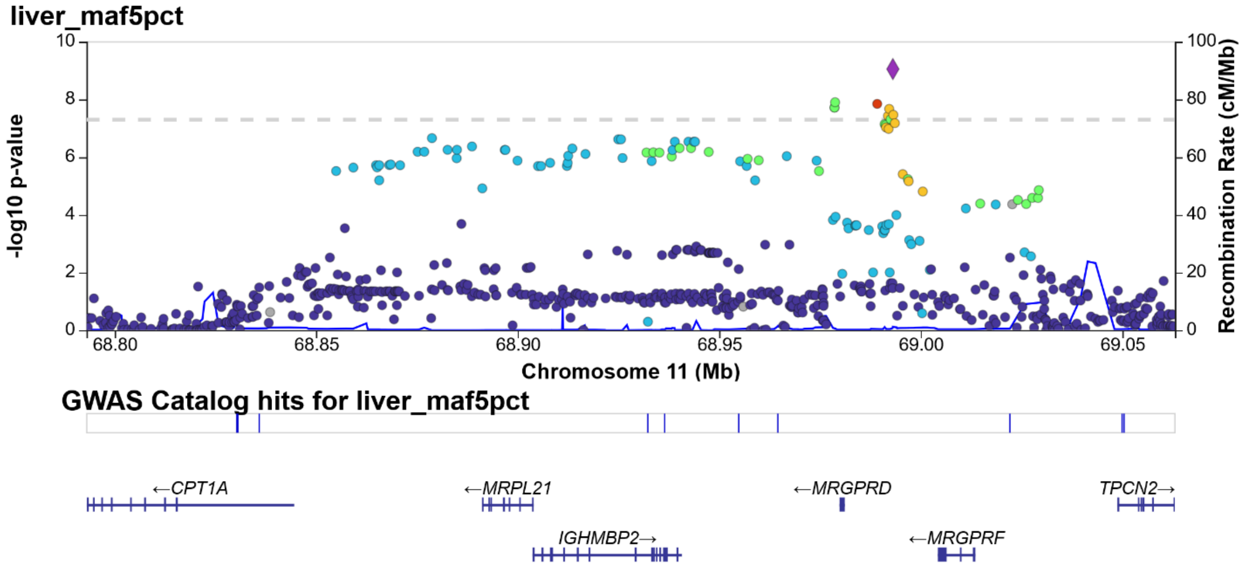

B

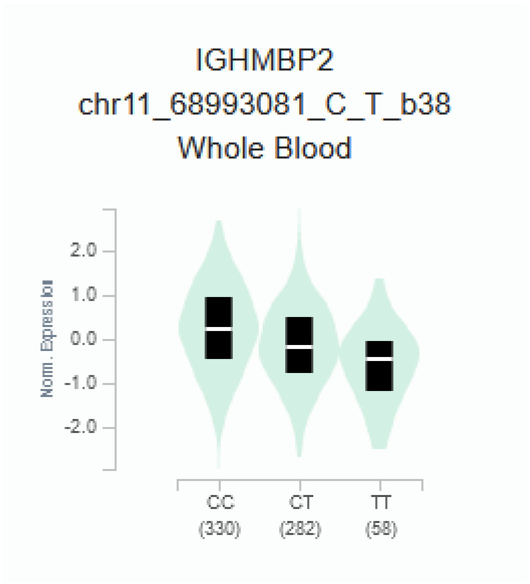

C

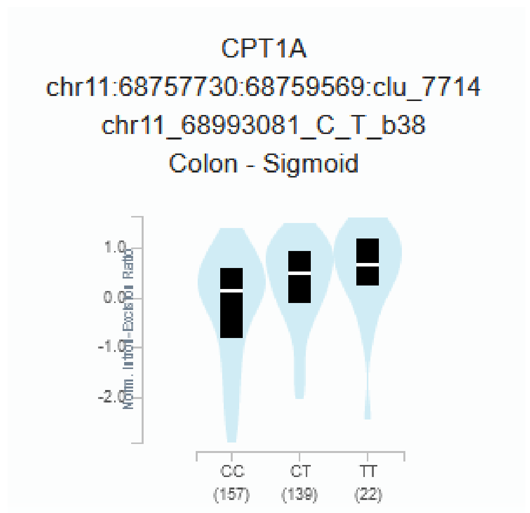

© 2022 Yang W et al. *JAMA Network Open*.

eReferences

1. Das S, Forer L, Schonherr S, et al. Next-generation genotype imputation service and methods. *Nat Genet*. Oct 2016;48(10):1284-1287. doi:10.1038/ng.3656
2. Bansal V, Libiger O. Fast individual ancestry inference from DNA sequence data leveraging allele frequencies for multiple populations. *BMC Bioinformatics*. Jan 16 2015;16:4. doi:10.1186/s12859-014-0418-7
3. Lee SHR, Antillon-Klussmann F, Pei D, et al. Association of Genetic Ancestry With the Molecular Subtypes and Prognosis of Childhood Acute Lymphoblastic Leukemia. *JAMA Oncol*. Mar 1 2022;8(3):354-363. doi:10.1001/jamaoncol.2021.6826
4. Sinnott-Armstrong N, Tanigawa Y, Amar D, et al. Genetics of 35 blood and urine biomarkers in the UK Biobank. *Nat Genet*. Feb 2021;53(2):185-194. doi:10.1038/s41588-020-00757-z
5. Choi SW, O'Reilly PF. PRSice-2: Polygenic Risk Score software for biobank-scale data. *Gigascience*. Jul 1 2019;8(7)doi:10.1093/gigascience/giz082

© 2022 Yang W et al. *JAMA Network Open*.
