# Supplementary material for: Association of Inherited Genetic Factors With Drug-Induced Hepatic Damage Among Children With Acute Lymphoblastic Leukemia
Source: JAMA Netw Open. 2022 Dec 29;5(12):e2248803. doi: 10.1001/jamanetworkopen.2022.48803 (PMC9857512; doi:10.1001/jamanetworkopen.2022.48803)
Supplement: Supplement 2. — Data Sharing Statement [file jamanetwopen-e2248803-s002.pdf]

Data Sharing Statement

Yang. Association of Inherited Genetic Factors With Drug-Induced Hepatic Damage Among Children With Acute Lymphoblastic Leukemia. *JAMA Netw Open*. Published December 29, 2022. doi:10.1001/jamanetworkopen.2022.48803

Data
Data available: Yes
Data types: Deidentified participant data
How to access data: To request access to de-identified data used in the preparation of this manuscript, contact jun.yang@stjude.org.
When available: With publication

Supporting Documents
Document types: None

Additional Information
Who can access the data: Approved researchers with appropriate data use agreements and human protections approval may be granted data access.
Types of analyses: Data will be available for approved research purposes.
Mechanisms of data availability: Approved researchers with appropriate data use agreements and human protections approval may be granted data access.
